# Atroposelective Pd-Catalyzed C(*sp* ^2^)–P Coupling Enabling Modular Assembly of Axially Chiral QUINAPO Ligands

**DOI:** 10.1021/jacsau.5c01633

**Published:** 2026-01-22

**Authors:** Zhiping Yang, Jiangtao Cheng, Jun Wang

**Affiliations:** † Department of Chemistry, 26679 Hong Kong Baptist University, Kowloon, Hong Kong 999077, China

**Keywords:** Axial Chirality, C−P coupling, Pd-catalysis, QUINAP, DYKAT

## Abstract

QUINAP ligands have been widely employed in a broad range of synthetically valuable asymmetric transformations, and their oxidized analogues, QUINAPOs, serve as effective Lewis base catalysts. Yet, a general and modular synthesis of these scaffolds has remained elusive. Herein, we present a Pd-catalyzed asymmetric C­(*sp* ^2^)–P coupling reaction of racemic heterobiaryl triflates with secondary phosphine oxides that furnishes axially chiral QUINAPOs in 47–98% yields and up to 95% ee across a broad substrate scope. The platform is diversity-oriented, enabling rapid access to structurally varied QUINAPO frameworks; subsequent deoxygenation delivers the corresponding QUINAP ligands. Representative members exhibit high activity and enantioselectivity in the asymmetric allylic alkylation and alkynylation of chromones, highlighting how ligand substitution modulates catalytic performance.

## Graphical abstract

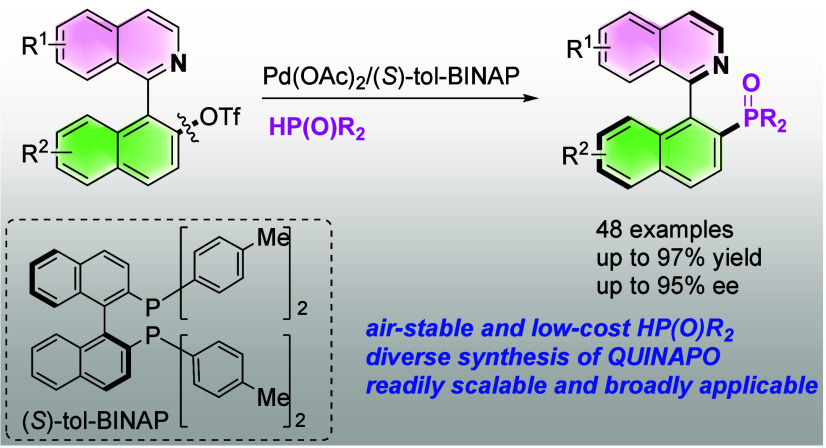

Structurally diverse chiral phosphine ligands are central to asymmetric catalysis because their steric and electronic properties can be finely tuned to control selectivity and reactivity in traditional metal-catalyzed processes.^1–7^ For instance, BINAP is a benchmark ligand with broad utility and exceptional efficacy, yet it has limitations in specific transformations. Systematic modification of substituents and phosphorus electronics has enabled control of dihedral angles and steric profiles, giving rise to a wide array of axially chiral phosphines that excel in asymmetric reactions, exemplified by DTBM-SegPhos and DifluorPhos.^1–3^

Axially chiral *P,N*-ligands, such as QUINAP, constitute another particularly powerful class, enabling key asymmetric transformations including hydroboration of alkenes, allylic alkylation, and 1,3-dipolar cycloaddition reaction. Their corresponding oxides (QUINAPOs) usually serve as effective Lewis base catalysts (Scheme 1a).^5^ However, the access of these ligands and organocatalysis often requires complex, multistep procedures and resolution protocols. This complexity hampers systematic tuning of their steric and electronic features and, thereby, limits their structural diversity and results in high cost (Scheme 1b).

**Scheme 1:**
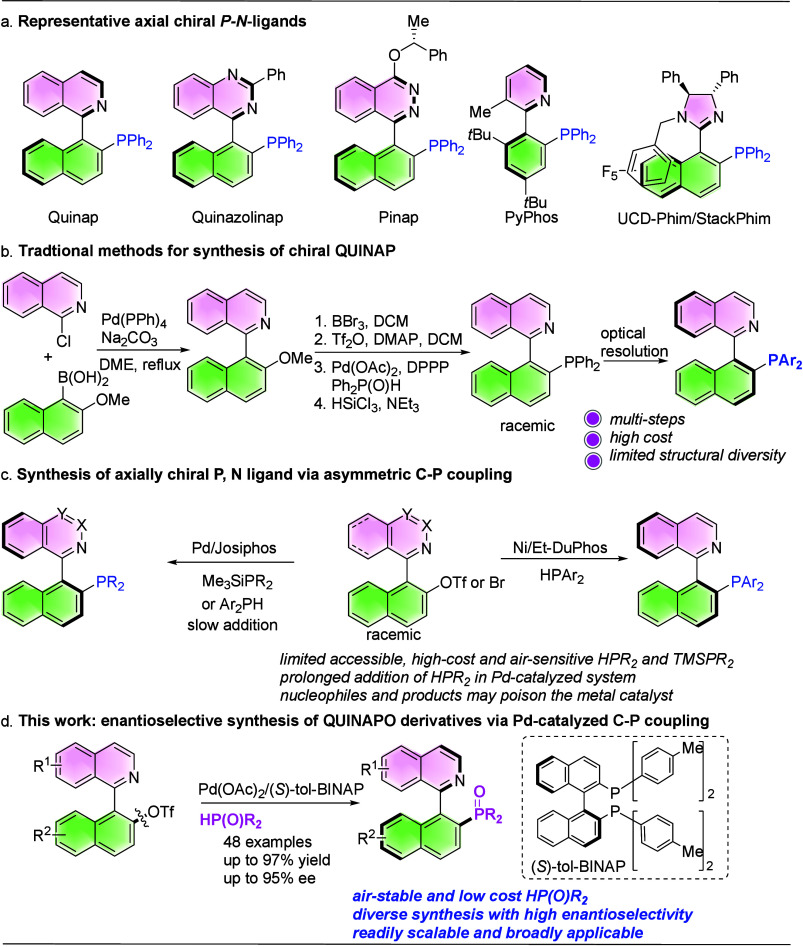
Pd-Catalyzed Asymmetric C­(*sp* ^*2*^)–P Coupling for Synthesis of Chiral QUINAPOs

Dynamic kinetic asymmetric transformation (DYKAT) of *N*-heterobiaryl derivatives, typically via metallacycle intermediates, has become a powerful platform for constructing axial chirality. Over the past decades, Pd,^8–20^ Rh,^21–25^ Ir,^26–29^ Ni,^30–33^ and Co^34–37^ -catalyzed DYKATs have demonstrated remarkable efficacy in synthesizing axially chiral heterobiaryls. Although chemists have developed various reactions for constructing C–P bonds in the synthesis of axially chiral phosphine compounds,^8–11,38–46^ transition metal-catalyzed C–P coupling for axially chiral *P,N*-ligands remains rare and limited in scope.^8–11,38^ This is mainly due to the potential for catalyst poisoning or deactivation caused by phosphorus nucleophiles and their products.

Pd-catalyzed C­(*sp* ^2^)–P coupling for the synthesis of chiral QUINAP was first disclosed in 2013, but it suffered from narrow scope and required slow addition of HPAr_2_ to suppress deleterious coordination between HPAr_2_ and metal catalysts and enable dynamic kinetic cross-coupling.^8,9^ Later, Lassaletta reported an expensive masked phosphorus nucleophile (trimethylsilylphosphines-TMSPR_2_) to realize dynamic kinetic C–P cross–coupling under mild condition without slow addition of nucleophiles.^10^ However, the aforementioned reports did not thoroughly investigate the effects of substituents on the *N*-heterobiaryl scaffold. Recently, our group developed a Ni-catalyzed DYKAT C­(*sp* ^2^)–P coupling of *N*-heterobiaryl triflates with HPAr_2_ that offered structurally diverse chiral QUINAPs with high yields and good enantioselectivities, systematically mapping the impact of substitution patterns (Scheme 1c).^38^ We also found that the coupling products can act as ligands in situ to some extent, thereby affecting both enantioselectivity and reactivity. This observation explains the challenges of achieving consistently high enantioselectivity in transition metal catalyzed C­(*sp* ^2^)–P coupling for construction of axially chiral phosphorus compounds. Because the phosphine products are easily air-oxidized, we typically isolated the corresponding oxides after rapid H_2_O_2_ oxidation. In general, both HPR_2_ and TMSPR_2_ nucleophiles are costly, air-sensitive, and strongly coordinating, which limits their general applicability in transition metal catalysis.

Secondary phosphine oxides (SPOs), as air-stable, inexpensive, low toxicity, and readily diversified phosphorus nucleophiles, offer an attractive alternative to the synthetic community. Additionally, their C–P coupling products usually do not coordinate strongly with metal catalysts. In recent decades, SPOs have been extensively employed in addition reactions to construct carbon-centered chirality^47^ and in C­(*sp* ^2^)–P coupling reactions to generate P-chirality.^48^ Nevertheless, the catalytic asymmetric synthesis of axially chiral phosphorus compounds, especially the atroposelective construction of *P*, *N* frameworks via C­(*sp* ^2^)–P bond formation, remains relatively underdeveloped. This difficulty likely arises from their tendency to coordinate with metals^49,50^ and their ability to act as reductants,^38^ both of which can adversely affect catalytic efficiency. Therefore, new catalytic systems that enable the efficient synthesis of axially chiral QUINAPOs from SPOs via asymmetric C­(*sp* ^2^)-P coupling are highly desirable. While this manuscript was in preparation, Liu reported a diastereo- and enantioselective Pd-catalyzed C­(*sp* ^2^)–P coupling that enables the synthesis of axially chiral and P-chiral phosphine oxides;^11^ however, substituent effects on the *N*-heterobiaryl scaffold were not comprehensively examined.

Herein, drawing on our prior work in chiral phosphorus chemistry^38,39,51–61^ and recent works about Pd-catalyzed asymmetric reactions,^62–67^ we report a simple and robust palladium catalytic system (Pd­(OAc)_2_ and (*S*)-tol-BINAP) that facilitates enantioconvergent C­(*sp* ^2^)-P coupling of *N*-heterobiaryl triflates with SPOs, producing a series of axially chiral QUINAPOs with excellent enantioselectivity (Scheme 1d). The method tolerates diverse substitution patterns on the *N*-heterobiaryl core and provides a modular entry to QUINAPO scaffolds.

We began our study using *N*-heterobiaryl triflate (**1a**) and HP­(O)­Ph_2_ (**2a**) as model substrates, along with Pd­(OAc)_2_ and a variety of chiral phosphine ligands (**L1–L8**) (Table 1). (*R*, *R*)-Et-DuPhos (**L1**) was not applicable in the Pd-catalysis system but has shown good results in Ni-catalysis^38^ (entry 1). (*R*, *R*)-QuinoxP (**L2**) gave an 83% yield and 66% ee (entry 2), whereas (*R*, *R*)-BenzP (**L3**) was ineffective (entry 3). Other skeleton ligands such as (*R, R*)-Ph-BPE and (*R, R, R*)-Ph-SKP (**L4-L5**) were also unable to afford the expected product (entries 4–5). Finally, (*S*)-tol-BINAP (**L7**) gave the best enantioselectivity (entry 8, 94% ee). Changing the base to LiOAc or KOAc from NaOAc stopped the reaction or gave poor enantioselectivity (entries 7, 9–10). Reducing the amount of NaOAc to 3 equiv and increasing the equivalent of HP­(O)­Ph_2_ (**2a**) to 1.3 equiv improved the yield to 95% yield with 94% ee (entries 11–12). lternative Pd sources (Pd­(TFA)_2_, Pd­(PPh_3_)_4_, Pd_2_(dba)_3_) gave diminished yields at similar ee values (entries 13–15). Toluene proved superior to 1,4-dioxane and THF (entries 12, 16–17). Reducing the loading of Pd­(OAc)_2_ to 5 mol % and that of (*S*)-tol-BINAP (**L7**) to 6 mol % led to a similar result (entries 12, 18, 19, 98% NMR yield, 83% isolated yield and 94% ee).

**Table 1:**
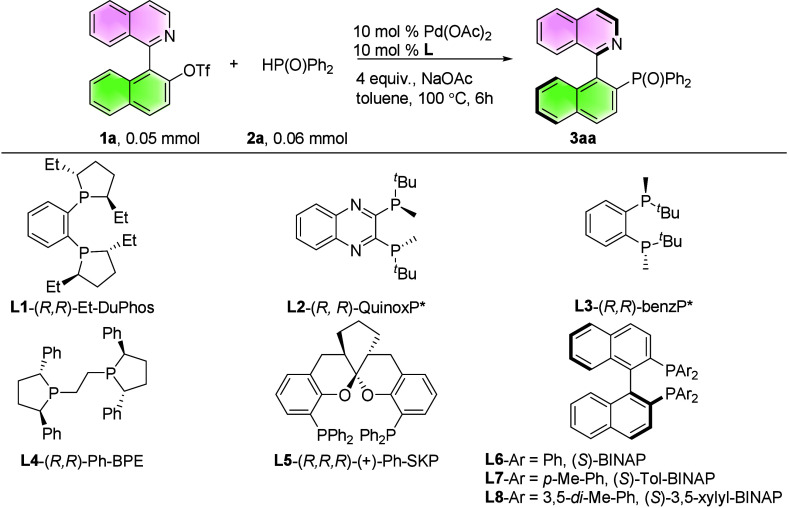
Optimization of the Reaction Conditions^a^

Entry	[Pd]	L	Base	Yield (%)	Ee (%)
1	Pd(OAc)_2_	**L1**	NaOAc	-	-
2	Pd(OAc)_2_	**L2**	NaOAc	83	66
3	Pd(OAc)_2_	**L3**	NaOAc	-	-
4	Pd(OAc)_2_	**L4**	NaOAc	-	-
5	Pd(OAc)_2_	**L5**	NaOAc	-	-
6	Pd(OAc)_2_	**L6**	NaOAc	55	84
7	Pd(OAc)_2_	**L7**	NaOAc	77	94
8	Pd(OAc)_2_	**L8**	NaOAc	37	94
9	Pd(OAc)_2_	**L7**	LiOAc	-	-
10	Pd(OAc)_2_	**L7**	KOAc	47	0
11^b^	Pd(OAc)_2_	**L7**	NaOAc	82	94
12^b^ ^c^	Pd(OAc)_2_	**L7**	NaOAc	95	94
13^b^ ^c^	Pd(TFA)_2_	**L7**	NaOAc	44	94
14^b^ ^c^	Pd(PPh_3_)_4_	**L7**	NaOAc	20	94
15^b^ ^c^	Pd_2_(dba)_3_	**L7**	NaOAc	36	94
16^b^ ^c^ ^d^	Pd(OAc)_2_	**L7**	NaOAc	89	92
17^b^ ^c^ ^e^	Pd(OAc)_2_	**L7**	NaOAc	93	94
18^b^ ^c^ ^f^	Pd(OAc)_2_	**L7**	NaOAc	98	94
19^b^ ^c^ ^g^	Pd(OAc)_2_	**L7**	NaOAc	98(83)	94

a Reaction conditions: **1a** (0.05 mmol), **2a** (0.06 mmol), 10 mol % Pd­(OAc)_2_, 10 mol % **L**, in 1 mL toluene, 100 °C, 6 h. Yield (**3aa**) was based on ^31^P NMR analysis with PPh_3_ as internal standard. Ee was determined by HPLC analysis.

b 3 equiv. NaOAc.

c 1.3 equiv. HP­(O)­Ph_2_.

d 1,4-dioxane.

e THF.

f **1a** (0.1 mmol), **2a** (0.13 mmol), 10 mol % Pd­(OAc)_2_, 10 mol % **L7** in 2 mL toluene.

g **1a** (0.1 mmol), **2a** (0.13 mmol), 5 mol % Pd­(OAc)_2_, 6 mol % **L7** in 2 mL toluene. Isolated yield.

After optimizing the reaction conditions, we expanded our study to include a wider range of substrates, investigating the effects of various substituents on the naphthalene and isoquinoline rings (Table 2). The presence of -Me, -OMe and -CO_2_Me groups in the naphthalene ring gave good yield and enantioselectivity (**3ba**–**3fa**, 58–93%, 92–94% ee). 6-MeO-substituted naphthalene decreased yield with good enantioselectivity (**3ea**, 58%, 94% ee). 6-*i*Pr-substituted isoquinoline-based *N*-heterobiaryl triflate was also compatible with the reaction, showing a slight decrease in yield and enantioselectivity (**3ga**, 60%, 85% ee). Next, incorporating a phenyl substituent into the naphthalene or isoquinoline ring also led to high yield and enantioselectivity (**3ha**–**3na**, 64–96% yield, 88–95% ee). To broaden the reaction scope, we examined the effect of various substituents on the naphthalene ring. Electron-donating groups (Me and OMe), electron-withdrawing groups (F), and 2-naphthalene and thiophene groups were well-tolerated, yielding satisfactory results (**3oa**–**3ta**, 74–96% yield, 90–93% ee). Moreover, *N*-heterobiaryl triflates bearing electron-donating groups (Me and OMe), electron-withdrawing groups (F), and thiophene on the isoquinoline ring also produced QUINAPOs efficiently (**3ua**–**3ya**, 67–97% yield, 86–94% ee). Furthermore, the -Ph group and -OMe positioned at various locations on *N*-heterobiaryl triflates also showed good practicality (**3za**–**3aga**, 65–88%, 90–95% ee). The quinazoline skeleton gave the desired product with moderate enantioselectivity (**3aha**, 81%, 73% ee) due to its quick racemization at high temperature.^38^

**Table 2:**
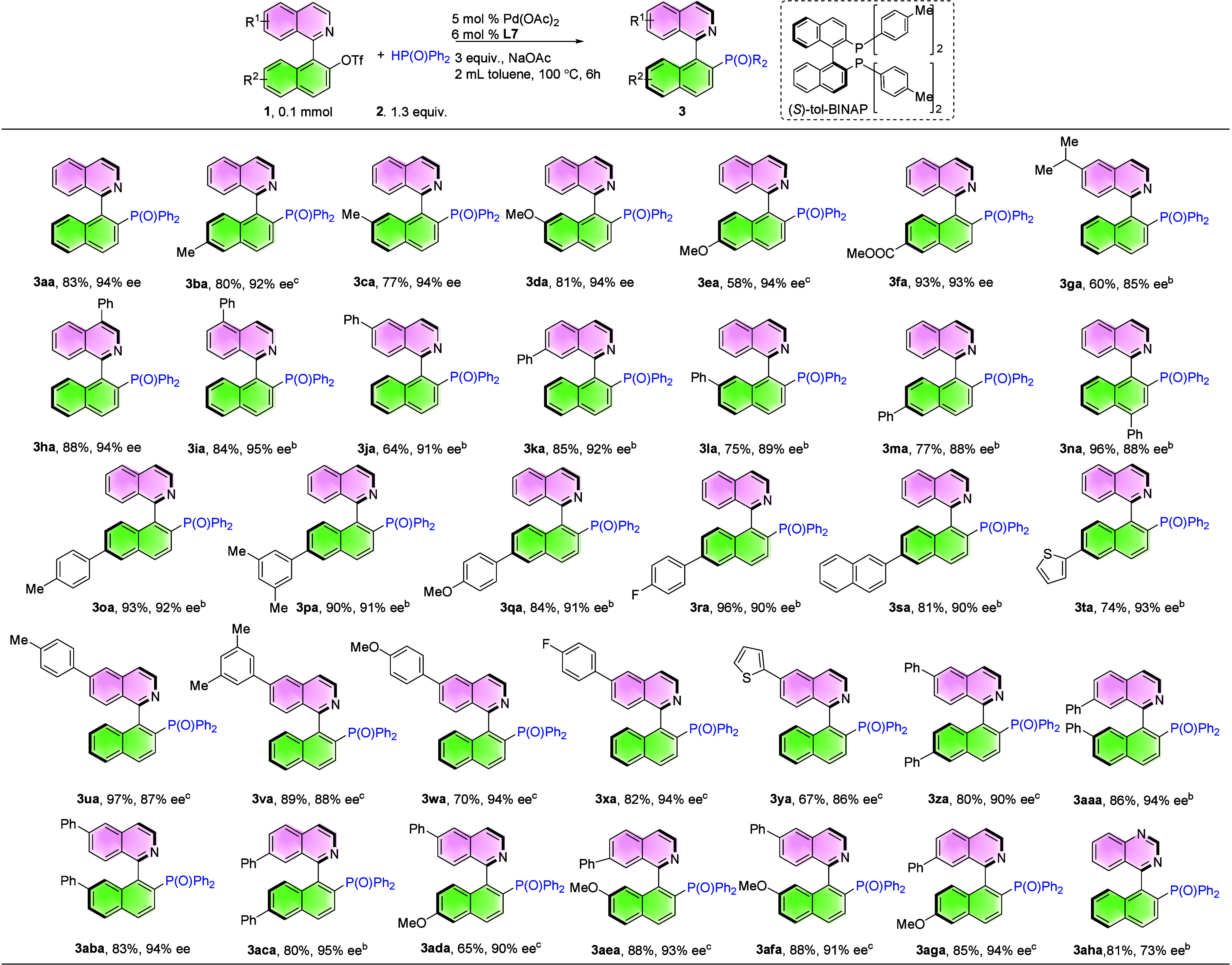
Evaluation of the Substrate Scope of *N*-Heterobiaryl Triflates with Different Substituents^a^

a Reaction conditions: **1** (0.1 mmol), **2a** (0.13 mmol), 5 mol % Pd­(OAc)_2_, 6 mol % **L7** and 3 equiv. NaOAc in 2 mL toluene, 100 °C, 6h. Ee was determined by HPLC analysis. Isolated yield.

b 12 h.

c 24 h.

Encouraged by the results obtained from *N*-heterobiaryl triflates, we further expanded this catalytic system with various HP­(O)­Ar_2_ to obtain chiral QUINAPOs (Table 3). Diphenylphosphine oxide with electron-donating groups (-Me, -*t*Bu, -OMe and -Ph) or with electron-withdrawing groups (F, Cl and CF_3_) was subjected to C­(*sp* ^2^)-P coupling, and the corresponding QUINAPOs were formed in good yields and enantioselectivities (**3ab**–**3aj**, 71–93%, 86–94% ee). *o*-Me-diphenylphosphine oxide did not give the product, mainly due to steric hindrance (**3ad**). Polysubstituted diphenylphosphine oxide and phosphine oxide with 2-naphthalene were tolerated well (**3ak**–**3am**, 70–98%, 91–94% ee). Dibenzylphosphine oxide was converted smoothly into the desired QUINAPO (**3an**, 90% yield, 20% ee). Ethyl­(phenyl)­phosphine oxide also gave the desired product in good yield and enantioselectivity with a dr of 1.6:1 (**3ao**, 87%, major 82% ee, minor 83% ee). As for ethyl phenylphosphinate, a moderate yield was obtained (**3ap**, 47%, major 2% ee, minor 20% ee). Phenyl­(*o*-tolyl)­phosphine oxide could detect only trace product. *tert*-Butyl-phenyl-phosphine oxide and dicyclohexylphosphine oxide did not produce the target product; instead, it yielded a detriflate and protonation products.^38^

**Table 3:**
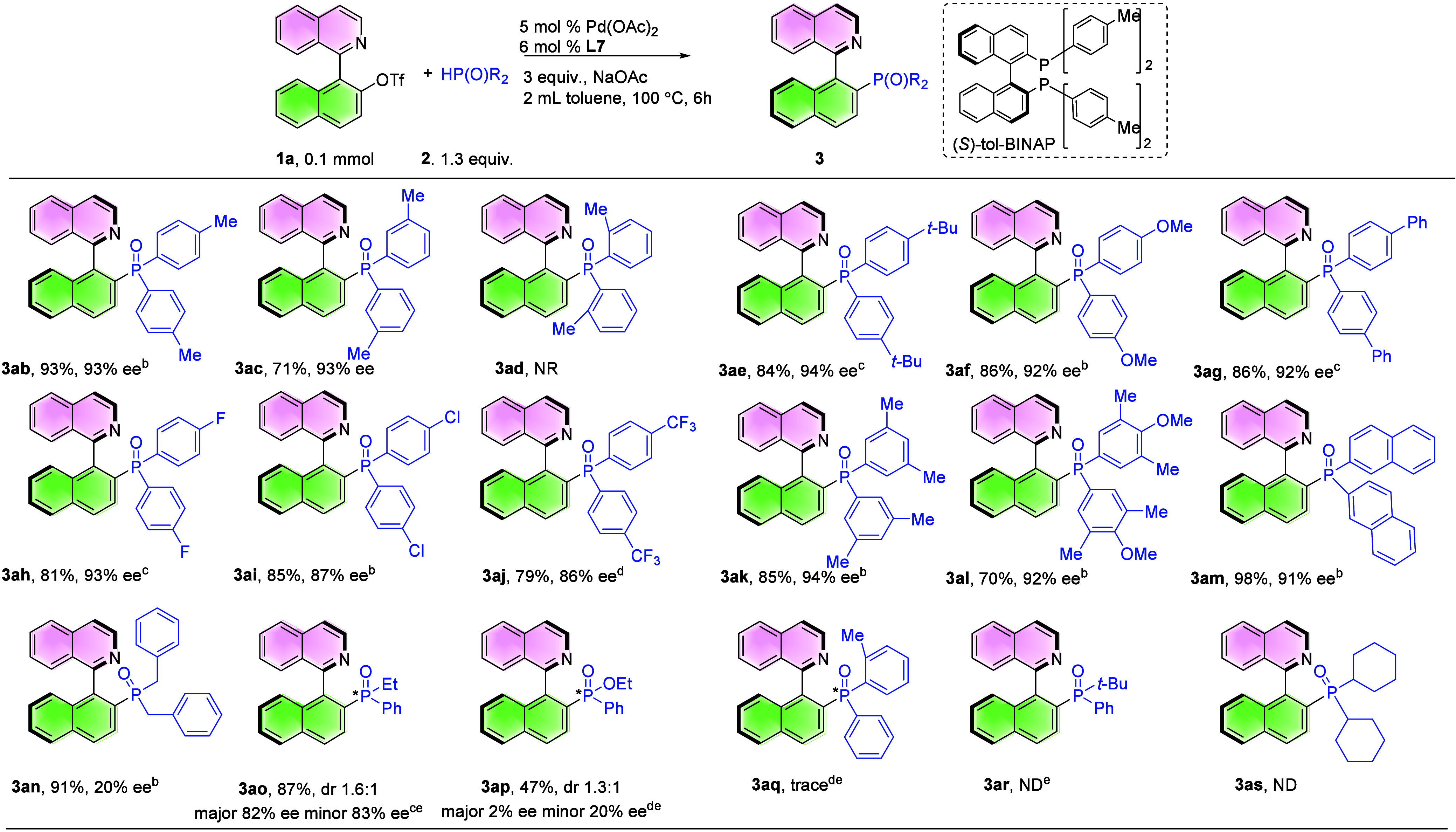
Evaluation of the Substrate Scope of HP­(O)­Ar_2_ ^a^

a Reaction conditions: 1a (0.1 mmol), 2 (0.13 mmol), 5 mol % Pd­(OAc)_2_, 6 mol % L7 and 3 equiv NaOAc in 2 mL toluene, 100 °C, 6h. Ee was determined by HPLC analysis. Isolated yield.

b DIPEA instead of NaOAc.

c 12 h.

d 24 h.

e 2 equiv **2**.

To evaluate the efficiency of the current method, a 1 mmol scale reaction was conducted, yielding **3aa** (82%, 93% ee). In addition, the 0.3 mmol scale reaction also gave satisfactory results (**3ha**, **3za** and **3aaa**). QUINAPOs were further converted to QUINAPs via reduction with HSiCl_3_, affording the product in good yield with a slight decrease in enantioselectivity (**3aa′**, **3ha′**, **3za′** and **3aaa′**). The configuration of the product (**3aa′**) was consistent with (*R*)-QUINAP (See SI) (Scheme 2a). These QUINAP ligands were subsequently employed in Pd-catalyzed allylic alkylation and Cu-catalyzed alkynylation of chromone, demonstrating the versatility of this methodology and the influence of various ligand substituents (Scheme 2b, 2c).

**Scheme 2:**
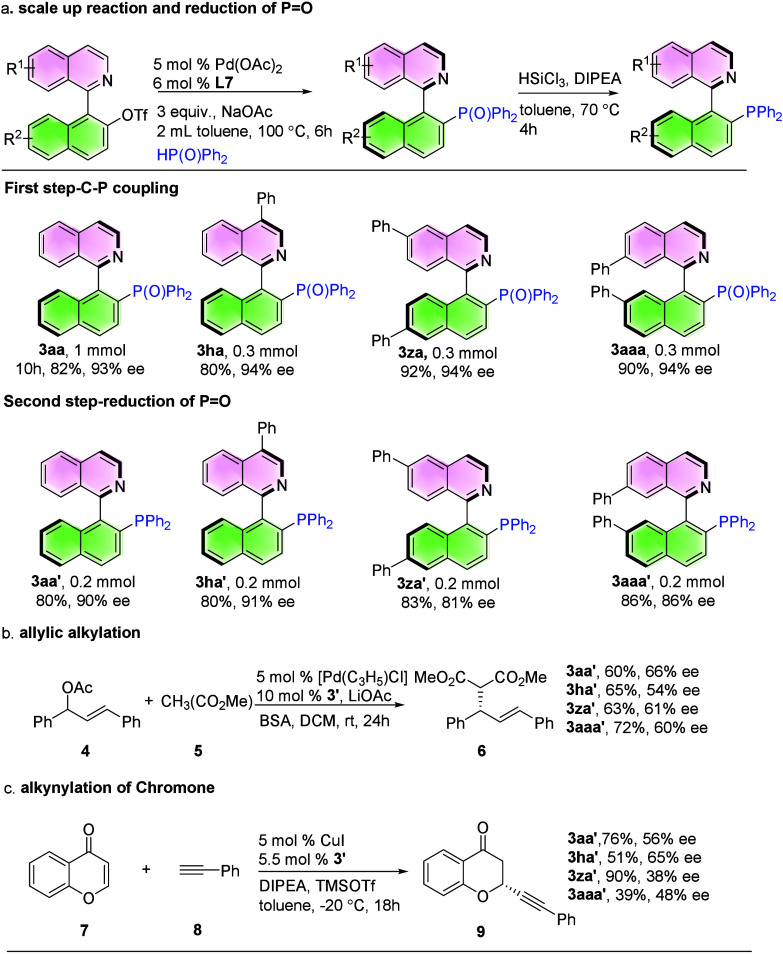
Access to Chiral QUINAPs and Applications in Enantioselective Catalysis

To gain insight into the Pd-catalyzed asymmetric C­(*sp* ^2^)-P coupling reaction, control experiments were conducted. The enantioselectivity decreased significantly in the absence of a nitrogen atom (**12**, 80%, 5% ee), suggesting that the formation of a five-membered palladacycle is critical for this asymmetric induction. The pyridine skeleton gave a racemic product (**13**, 90% yield, racemic), likely due to the small steric hindrance of the methyl group (Scheme 3a). Based on recent work about asymmetric synthesis of *N*-heterobiaryl derivatives via Pd-catalysis^11,18,19^ and the investigations of the control experiment, a possible mechanism is proposed as follows. First, isoquinoline’s nitrogen atom coordinates to the Pd^0^ **L7** complex, thereby facilitating the oxidative addition of the C–O bond in triflate **1a**, forming 5-membered cationic palladacycle (Ar–Pd^II^ **L7**) diastereoisomers **I** and **II**, which is proposed to be the enantio-determining step.^68^ Subsequent ligand exchange with SPO furnishes intermediate **III**, which can undergo a competitive protonolysis of the Pd–C bond in the biaryl moiety to give byproduct (**14**, yield <5% in most cases) that was isolated in the reaction. The main pathway nevertheless proceeds via reductive elimination to afford **3aa** and regenerate the Pd^0^ **L7** complex (Scheme 3b).

**Scheme 3:**
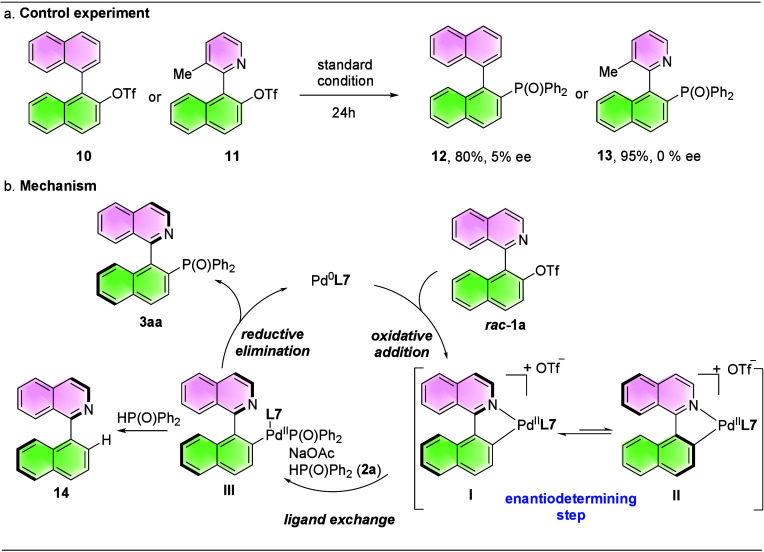
Mechanistic Investigations

In summary, we have established an atroposelective DYKAT strategy for the asymmetric synthesis of QUINAPOs derivatives via a Pd/(*S*)-tol-BINAP-catalyzed C­(*sp* ^2^)–P cross-coupling reaction. Various *N*-heterobiaryl triflates and HP­(O)­R_2_ were efficiently engaged in the reaction, producing chiral QUINAPOs with yields of up to 98% and enantioselectivities of up to 95% ee. This protocol facilitates the efficient synthesis of a diverse array of substituted QUINAP ligands, which have been successfully applied in the asymmetric allylic alkylation and alkynylation of chromones, thereby preliminarily demonstrating the influence of various ligand substituents. Upcoming studies will concentrate on developing a wider array of QUINAP ligands to solve difficult problems in asymmetric catalysis.

## Supplementary Material



## Notes

Experimental procedures and analysis data for all new compounds (PDF)
