# Supplementary material for: Atroposelective Pd-Catalyzed C(sp 2)–P Coupling Enabling Modular Assembly of Axially Chiral QUINAPO Ligands
Source: JACS Au. 2026 Jan 22;6(2):779–87. doi: 10.1021/jacsau.5c01633 (PMC12933337; doi:10.1021/jacsau.5c01633)
Supplement: Supplementary file 1 [file au5c01633_si_001.pdf]

Supporting Information

Atroposelective Pd-Catalyzed C(sp^2)–P Coupling Enabling Modular Assembly of Axially Chiral QUINAPO Ligands

Zhiping Yang,[a] Jiangtao Cheng,[a] and Jun (Joelle) Wang[a]*

[a] Department of Chemistry, Hong Kong Baptist University, Kowloon, Hong Kong, China.

CONTENT

1. General information 1
2. General procedure for Pd-catalyzed C(sp^2)-P coupling 2
3. Preparation of triflates 2
4. Large scale reaction and synthetic transformation of the products 4
5. Application of QUINAP derivatives in asymmetric reaction 6
6. Analytic data for the products 7
7. Refference 47
8. NMR Spectrum 48
9. HPLC spectrum 134

1. General information

NMR Spectra were recorded on a Bruker DPX-500 (400) spectrometer at 600 MHz or 400 MHz for ^{1}H NMR, 240MHz or 160MHz for ^{31}P NMR, 564 MHz or 376 MHz for ^{19}F NMR and 150 MHz or 100 MHz for ^{13}C NMR in $CDCl_3$ with tetramethylsilane (TMS) or the residual deuterated solvent peaks as internal standard. Chemical shifts (δ) are reported in ppm, and coupling constants (*J*) are in Hertz (Hz). Flash column chromatograph was carried out using 200-300 mesh silica gel at medium pressure. High resolution mass spectra (HRMS) were recorded on a LC-TOF spectrometer. ESI-HRMS data were acquired using a Thermo LTQ Orbitrap XL Instrument equipped with an ESI source. Unless otherwise noted, all reagents were purchased from commercial suppliers and used without purification. All air- and moisture-sensitive manipulations were carried out with standard Schlenk techniques under nitrogen or in a glove box under argon.

2. General procedure for Pd-catalyzed C(sp^2)-P coupling

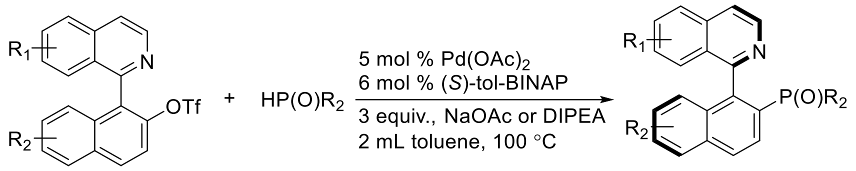

1, 0.1 mmol **2**, 1.3 equiv. **3**

In an argon-filled glovebox, an oven-dried vial fitted with a stirrer bar was charged with 5 mol % $Pd(OAc)_2$ (1.1 mg) and 6 mol % (*S*)-tol-BINAP (4.1 mg) in 2 mL toluene and the mixture was stirred at room temperature for 10 min. Then, 0.3 mmol NaOAc (24.6 mg) or DIPEA (38.7 mg) was added. After 0.1 mmol triflates (**1**) and 0.13 mmol $HP(O)R_2$ (**2**) were added, the mixture was allowed to stir for the indicated time at 100 °C. The residue was chromatographed on silica gel (EA / PE = 1:1 to 3:1) to get the corresponding QUINAPO derivatives (**3**). The ee value of **3** was determined by HPLC.

The racemic QUINAPO derivatives were directly prepared by heating a mixture of the corresponding 0.05 mmol starting triflates (**1**) and 0.065 mmol $HP(O)R_2$ (**2**), 10 mol % *rac*-BINAP (3.1 mg), 10 mol% $Pd(OAc)_2$ (1.1 mg), 0.15 mmol NaOAc (24.6 mg) in 1 mL toluene at 100 °C for 12 h.

3. Preparation of triflates

Procedure for the synthesis of QUINOL derivatives according to Tan's method (*Nat. Commun.* 2021, *12*, 2384-2392)

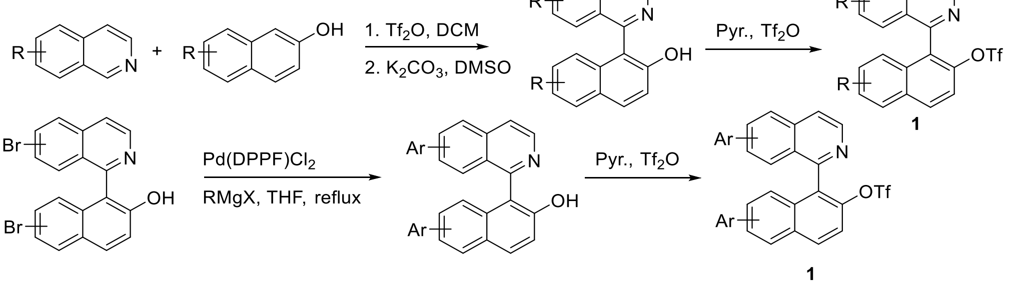

To a dried Schlenk tube was added 5Å molecular sieve (2.5 g), isoquinoline (12.5 mmol) and CH_2Cl_2 (40 mL) under argon atmosphere. After the solution was cooled to -50°C, Tf_2O (5 mmol) was added through a microsyringe and stirred for half an hour until white solid salts appeared. After adding the solution of 2-naphthol (5 mmol) in CH_2Cl_2 (40 mL), the reaction was slowly raised to room temperature, and then continued to stir for 2-48 hours monitored by TLC. The molecular sieve was removed by filtration, and the resulting filtrate was washed successively with 3M HCl, saturated sodium bicarbonate and brine, and then dried over anhydrous Na_2SO_4. After evaporating the solvent under reduced pressure, the resulting intermediate was dissolved in 40 mL of DMSO, and K_2CO_3 (1.38 g, 10 mmol) was then added. The mixture was stirred at 60 °C for about 3~4 hours until the intermediate was consumed completely. The reaction system was adjusted to weakly acidic with 3M HCl, and then to weakly basic with saturated sodium bicarbonate. The mixture was diluted with 20 mL of water, and extracted with EA (20 mL × 3). The combined organic phase was washed with brine (20 mL × 2), dried over anhydrous Na_2SO_4 and concentrated to afford the crude product, which was purified by column chromatography eluted with (EA / PE = 1:10 to 1:1) to afford the pure product.

Modified procedure for the synthesis of QUINOL derivatives with Ar-substituent

A solution of Br-QUINOL (2 mmol) and Pd(DPPF)Cl_2 (29.2, 0.04 mmol, 2 mol %) in 8 mL THF under N_2 atmosphere. Then, RMgX (X = Cl or Br) (6 mmol or 8 mmol) was added to the mixture at 0 ºC. The reaction mixture was stirred at 80 °C for 6-12 h monitored by TLC. After cooled to room temperature, the reaction was quenched with 1 M HCl aqueous solution. Then, $NaHCO_3$ (aq.) was added until pH > 7. The water phase was extracted with EA three times. The combined organic layer was dried over Na_2SO_4, filtered, and concentrated under reduced pressure. The crude material was purified by a SiO_2 flash column chromatography (EA / PE = 1:10 to 1:2) to afford QUINOL derivatives with Ar-substituent.

Procedure for the synthesis of triflates

Tf_2O (1.05 eq.) was added dropwise under argon to a solution of 2 mmol QUINOL derivatives and pyridine (2.0 eq.) in dry CH_2Cl_2. After stirring at room temperature for 1h (monitored by TLC), the reaction was quenched with saturated $NaHCO_3$, then extracted with CH_2Cl_2. The combined organic layer was dried over anhydrous Na_2SO_4, filtered and concentrated. The residue was purified by flash chromatography eluted with (EA / PE = 1:20 to 1:3) to afford triflates.

4. Large scale reaction and synthetic transformation of the products

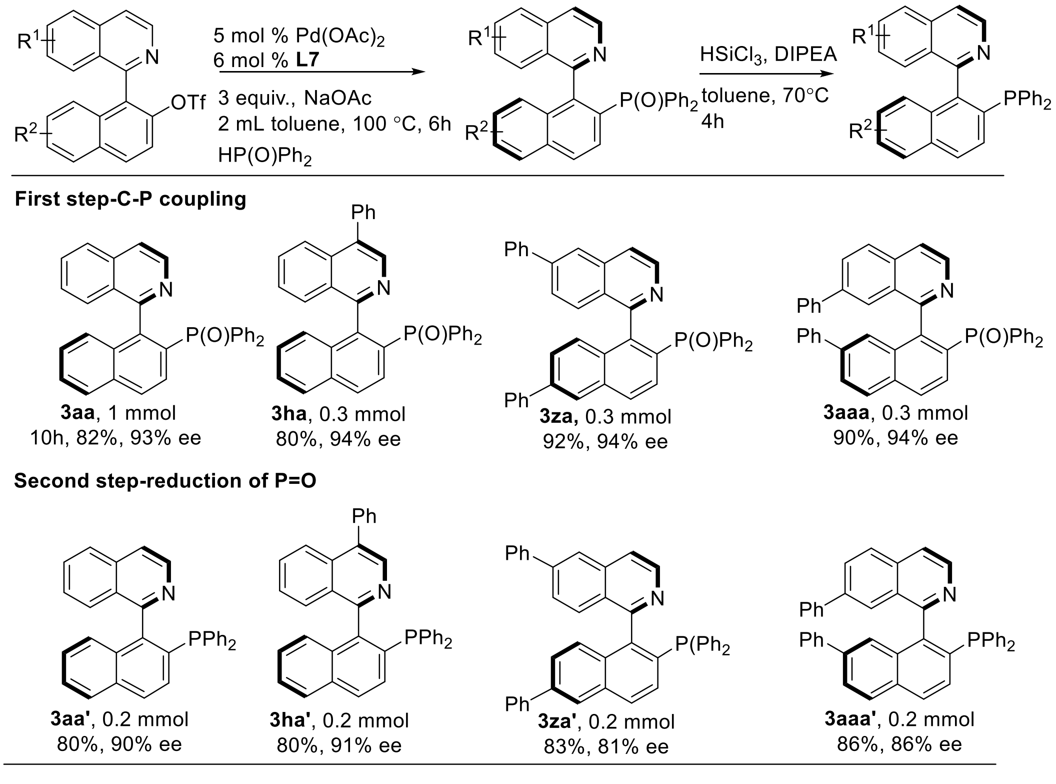

1 mmol scale reaction. In an argon-filled glovebox, an oven-dried vial fitted with a stirrer bar was charged with 5 mol % $Pd(OAc)_2$ (11.2 mg) and 6 mol % (*S*)-tol-BINAP (40.6 mg) in 20 mL toluene and the mixture was stirred at room temperature for 10 min. Then, 3.0 mmol NaOAc (246.0 mg) was added. After 1.0 mmol triflates (**1a**) and 1.3

mmol $HP(O)Ph_2$ (**2a**) were added, the mixture was allowed to stir for 10 h at 100 °C. The residue was chromatographed on silica gel (EA / PE = 3 : 1) to get the corresponding QUINAPO derivatives (**3aa,** 82%, 93% ee).

0.3 mmol scale reaction. In an argon-filled glovebox, an oven-dried vial fitted with a stirrer bar was charged with 5 mol % $Pd(OAc)_2$ (3.3 mg) and 6 mol % (*S*)-tol-BINAP (12.2 mg) in 6 mL toluene and the mixture was stirred at room temperature for 10 min. Then, 0.9 mmol NaOAc (73.8 mg) was added. After 1.0 mmol triflates (**1**) and 1.3 mmol $HP(O)Ph_2$ (**2a**) were added, the mixture was allowed to stir for 6 h at 100 °C. The residue was chromatographed on silica gel (EA / PE = 3 : 1) to get the corresponding QUINAPO derivatives (**3ha** (80%, 94% ee), **3za** (92%, 94% ee), **3aaa** (90%, 94% ee)).

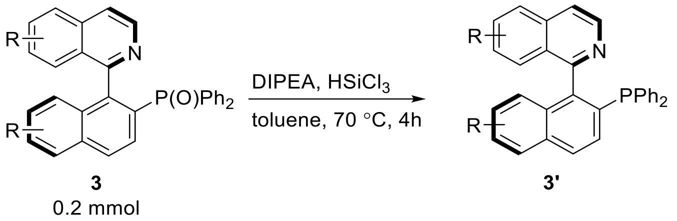

Reduction of P=O. An argon filled Schlenk tube was charged with **3** (0.2 mmol) and 4 mL toluene at Ar atmosphere. $HSiCl_3$ (7.5 equiv.) and DIPEA (9.5 equiv.) were added and the mixture was heated to 70 °C for 4 h, then cooled with ice, and diluted with CH_2Cl_2. A 2M NaOH solution was added carefully and the aqueous layer was extracted with CH_2Cl_2 twice. The combined organic layer was dried ($MgSO_4$), and concentrated to afford a white solid. The residue was purified by flash chromatography eluted with (EA / PE = 1:20 to 1:3) to afford **3’** (**3aa’** (80%, 90% ee), **3ha’** (80%, 91% ee), **3za’** (83%, 81% ee), **3aaa’** (86%, 86% ee)). During the purification of **3ha’**, **3za’** and **3aaa’**, we found that they were easily oxidized by air, resulting in partial phosphine oxide impurities appearing in the spectrum.

The absolute configurational determination of 3aa’

Compared with HPLC spectrum of commercial (*R*)-QUINAP, the absolute configurational determination of **3aa’** is ***R***.

The enantiomeric excess was determined by Daicel Chiralcel ID (0.46 cm x 25 cm), Hexanes / IPA = 70 / 30, 1.0 mL/min, λ = 254 nm, t (minor) = 7.97 min, t (major) = 9.93 min.

(*R*)-1-(2-(Diphenylphosphaneyl)naphthalen-1-yl)isoquinoline (3aa')

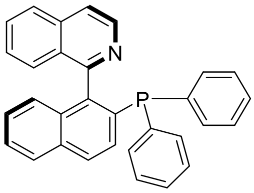

3aa'

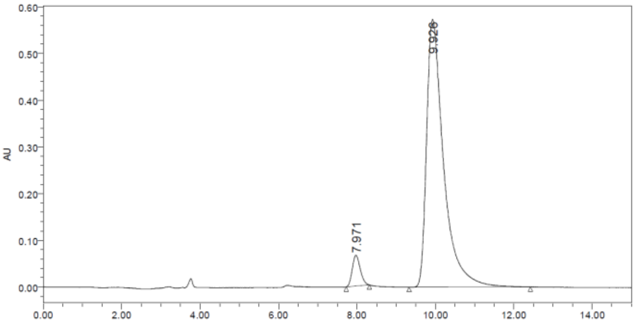

	RT	Area	% Area	Height
1	7.971	936333	5.15	66005
2	9.926	17262260	94.85	572982

R-QUINAP

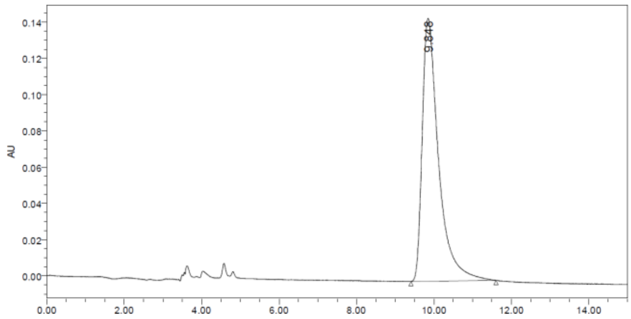

	RT	Area	% Area	Height
1	9.848	4246827	100.00	144962

5. Application of QUINAP derivatives in asymmetric reaction

allylic alkylation

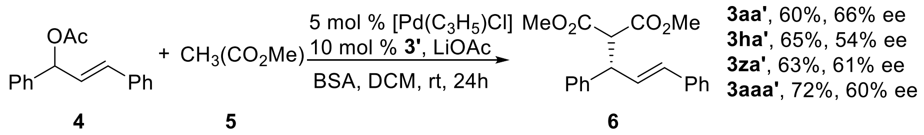

Allylic alkylation. In an argon-filled glovebox, an oven-dried vial fitted with a stirrer bar was charged with 5 mol % $[Pd(C_3H_5)Cl]_2$, 10 mol % ligand **3**' and 10 mol % LiOAc in 1 mL DCM and the mixture was stirred at room temperature for 10 min. Then, 0.1

mmol racemic 1,3-diphenyl-2-propenyl acetate (**4**), 0.3 mmol dimethyl malonate (**5**) and 0.3 mmol BSA were added and the resulting mixture was stirred at rt for 24 hours before diluted with Et_2O (5.0 mL) and quenched by saturated NH_4Cl aqueous solution (5.0 mL). The aqueous layer was extracted with Et_2O (3 x 5 mL) and the combined organics were washed with brine (10 mL), dried over Na_2SO_4, filtered, and concentrated. The obtained residue was purified by flash chromatography on silica gel (EA / PE = 1 / 20) to afford the product (**6**) as a colorless oil.

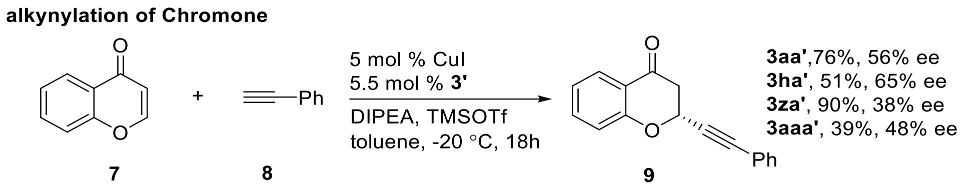

Alkynylation of Chromone. In an argon-filled glovebox, an oven-dried vial fitted with a stirrer bar was charged with 5 mol % CuI and 5.5 mol% ligand **3'** in 1 mL toluene and the mixture was stirred at room temperature for 10 min. Then, 0.16 mmol DIPEA, 0.1 mmol chromone (**7**) and 0.13 mmol phenylacetylene (**8**) were added and the resulting mixture was stirred at -78 °C and 0.13 mmol TMSOTf was added. The tube was then transferred to a -20 °C bath and allowed to stir for 18 hours. The reaction was then quenched with 3N HCl and allowed to stir for 2 hours until the silyl enol ether completely hydrolyzed as monitored by TLC. Saturate aqueous $NaHCO_3$ was added to neutralize the solution and the reaction mixture was extracted with EtOAc (3 x 5 mL). The organic layer was dried over anhydrous magnesium sulfate, filtered and the solvent removed in vacuo. The crude residue was purified by flash column chromatography on silica gel (EA / PE = 1 / 10) to afford the product (**9**) as a white solid.

6. Analytic data for the products

Due to C−P coupling and the complexity of the spectrum of Quinap derivatives, doublets in the aromatic region cannot be assigned completely and they are listed as singlets partially.

(*R*)-(1-(Isoquinolin-1-yl)naphthalen-2-yl)diphenylphosphine oxide (3aa)

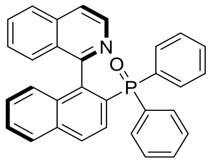

General procedure was used with triflates **1a** (40.3 mg, 0.1 mmol, 1 equiv.) and HP(O)Ph_2 **2a** (26.0 mg, 0.13 mmol, 1.3 equiv.) at 100 °C for 6 h to afford **3aa** as white wax (38.0 mg, 83% yield, 94% ee). $[\alpha]^{20}_{D}$ = 224.0 (c 1.0, $CHCl_3$). ^{1}H NMR (600 MHz, $CDCl_3$) δ 8.39 (s, 1H), 8.17-8.03 (m, 2H), 7.94 (d, *J* = 8.0 Hz, 1H), 7.89-7.79 (m, 2H), 7.65-7.59 (m, 1H), 7.55-7.49 (m, 2H), 7.49-7.42 (m, 2H), 7.42-7.34 (m, 2H), 7.28-7.21 (m, 2H), 7.20-7.17 (m, 1H), 7.14-7.04 (m, 2H), 7.01-6.92 (m, 1H), 6.91-6.76 (m, 3H). ^{13}C NMR (150 MHz, $CDCl_3$) δ 158.2 (d, *J* = 5.1 Hz), 141.4, 135.4, 134.7, 132.8, 132.5 (d, *J* = 11.5 Hz), 132.4 (d, *J* = 9.8 Hz), 132.1, 131.5 (d, *J* = 2.0 Hz), 131.0 (d, *J* = 10.1 Hz), 130.4, 130.0, 129.9, 129.3, 129.0, 128.7 (d, *J* = 11.6 Hz), 128.4 (d, *J* = 9.8 Hz), 128.1, 128.0, 127.9, 127.5, 127.2 (d, *J* = 13.0 Hz), 127.1 (d, *J* = 11.3 Hz), 126.9, 126.4, 121.1. ^{31}P NMR (240 MHz, $CDCl_3$) δ 30.7. The enantiomeric excess was determined by Daicel Chiralcel AD (0.46 cm x 25 cm), Hexanes / IPA = 70 / 30, 1.0 mL/min, λ = 230 nm, t (minor) = 9.4 min, t (major) = 11.6 min. HRMS (ESI-ion trap) *m/z*: $[M+H]^+$ calcd for $C_{31}H_{23}NOP$, 456.1512; found 456.1504.

(*R*)-(1-(Isoquinolin-1-yl)-6-methylnaphthalen-2-yl)diphenylphosphine oxide (3ba)

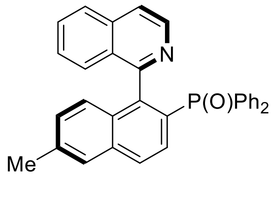

General procedure was used with triflates **1b** (41.7 mg, 0.1 mmol, 1 equiv.) and HP(O)Ph_2 **2a** (26.0 mg, 0.13 mmol, 1.3 equiv.) at 100 °C for 24 h to afford **3ba** as white wax (37.5 mg, 80% yield, 92% ee). $[\alpha]^{20}_{D}$ = 170.8 (c 1.0, $CHCl_3$). NMR (600 MHz, $CDCl_3$) δ 8.36 (d, *J* = 5.7 Hz, 1H), 8.12-8.06 (m, 1H), 8.00-7.97 (m, 1H), 7.88-7.81 (m, 2H), 7.70 (s, 1H), 7.63-7.59 (m, 1H), 7.52-7.48 (m, 1H), 7.47-7.43 (m, 1H), 7.43-7.40 (m, 1H), 7.40-7.36 (m, 2H), 7.24-7.20 (m, 1H), 7.19-7.16 (m, 1H), 7.10-7.04 (m, 3H), 6.97-6.92 (m, 1H), 6.83-6.79 (m, 2H), 6.77-6.73 (m, 1H), 2.46 (s, 3H). ^{13}C NMR (150

MHz, $CDCl_3$) δ 158.4 (d, *J* = 5.2 Hz), 141.4, 141.2 (d, *J* = 9.5 Hz), 138.0, 135.3, 135.0 (d, *J* = 2.0 Hz), 133.0, 132.4 (d, *J* = 9.9 Hz), 132.3, 131.6, 131.4 (d, *J* = 2.2 Hz), 131.0 (d, *J* = 10.3 Hz), 130.8 (d, *J* = 12.5 Hz), 130.2 (d, *J* = 2.2 Hz), 129.8, 129.3, 129.0, 129.0, 128.5 (d, *J* = 9.8 Hz), 128.3, 128.0 (d, *J* = 12.0 Hz), 127.5, 127.1, 127.0, 127.0, 126.9, 126.7, 126.4, 121.0, 21.6. ^{31}P NMR (240 MHz, $CDCl_3$) δ 30.2. The enantiomeric excess was determined by Daicel Chiralcel AD (0.46 cm x 25 cm), Hexanes / IPA = 70 / 30, 1.0 mL/min, λ = 254 nm, t (minor) = 8.6 min, t (major) = 13.5 min. HRMS (ESI-ion trap) *m/z*: $[M+H]^+$ calcd for $C_{32}H_{25}NOP$, 470.1668; found 470.1672.

(*R*)- (1-(Isoquinolin-1-yl)-7-methylnaphthalen-2-yl)diphenylphosphine oxide (3ca)

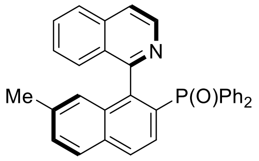

General procedure was used with triflates **1c** (41.7 mg, 0.1 mmol, 1 equiv.) and $HP(O)Ph_2$ **2a** (26.0 mg, 0.13 mmol, 1.3 equiv.) at 100 °C for 6 h to afford **3ca** as white wax (36.0 mg, 77% yield, 94% ee). $[\alpha]^{20}_D$ = 187.4 (c 1.0, $CHCl_3$). NMR (600 MHz, $CDCl_3$) δ 8.38 (s, 1H), 8.08-7.97 (m, 2H), 7.87-7.76 (m, 3H), 7.66-7.59 (m, 1H), 7.54-7.50 (m, 1H), 7.48-7.42 (m, 2H), 7.40-7.33 (m, 3H), 7.27-7.16 (m, 2H), 7.11-7.02 (m, 2H), 7.00-6.93 (m, 1H), 6.87-6.76 (m, 2H), 6.61 (s, 1H), 2.17 (s, 3H). ^{13}C NMR (150 MHz, $CDCl_3$) δ 158.4 (d, *J* = 5.2 Hz), 141.3, 140.6, 137.1, 135.4, 133.1, 133.0, 132.8 (d, *J* = 11.5 Hz), 132.4 (d, *J* = 9.5 Hz), 132.2, 131.5 (d, *J* = 2.1 Hz), 131.0 (d, *J* = 10.3 Hz), 130.3, 129.9, 129.8, 129.2, 129.1, 128.5 (d, *J* = 11.6 Hz), 128.1 (d, *J* = 12.4 Hz), 127.9, 127.5, 127.5, 127.2 (d, *J* = 12.8 Hz), 127.0, 126.4, 125.6, 121.2, 21.8. ^{31}P NMR (240 MHz, $CDCl_3$) δ 30.7. The enantiomeric excess was determined by Daicel Chiralcel AD (0.46 cm x 25 cm), Hexanes / IPA = 70 / 30, 1.0 mL/min, λ = 254 nm, t (minor) = 6.4 min, t (major) = 7.3 min. HRMS (ESI-ion trap) *m/z*: $[M+H]^+$ calcd for $C_{32}H_{25}NOP$, 470.1668; found 470.1672.

(*R*)-(1-(Isoquinolin-1-yl)-7-methoxynaphthalen-2-yl)diphenylphosphine oxide (3da)

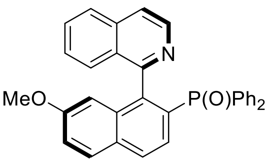

General procedure was used with triflates **1d** (43.3 mg, 0.1 mmol, 1 equiv.) and $HP(O)Ph_2$ **2a** (26.0 mg, 0.13 mmol, 1.3 equiv.) at 100 °C for 6 h to afford **3da** as white wax (39.4 mg, 81% yield, 94% ee). $[\alpha]^{20}_{D}$ = 153.4 (c 1.0, $CHCl_3$). ^1H NMR (600 MHz, $CDCl_3$) δ 8.38 (s, 1H), 8.02-7.91 (m, 2H), 7.83-7.36 (m, 3H), 7.65-7.59 (m, 1H), 7.54-7.49 (m, 1H), 7.47-7.40 (m, 2H), 7.40-7.32 (m, 2H), 7.29-7.16 (m, 3H), 7.15-7.04 (m, 2H), 7.02-6.93 (m, 1H), 6.91-6.77 (m, 2H), 6.10 (s, 1H), 3.33 (s, 3H). ^{13}C NMR (150 MHz, $CDCl_3$) δ 158.4 (d, *J* = 4.7 Hz), 158.2, 141.4, 139.8, 135.4, 133.8 (d, *J* = 12.3 Hz), 132.8, 132.3 (d, *J* = 10.6 Hz), 131.5 (d, *J* = 2.3 Hz), 131.0 (d, *J* = 10.1 Hz), 130.4, 130.3, 129.9, 129.6, 129.5, 128.8, 128.4 (d, *J* = 11.8 Hz), 128.1 (d, *J* = 13.0 Hz), 127.5, 127.2 (d, *J* = 12.4 Hz), 127.0, 126.4, 126.3 (d, *J* = 10.1 Hz), 121.1, 120.5, 105.3, 54.8. ^{31}P NMR (240 MHz, $CDCl_3$) δ 30.8. The enantiomeric excess was determined by Daicel Chiralcel AD (0.46 cm x 25 cm), Hexanes / IPA = 70 / 30, 1.0 mL/min, λ = 254 nm, t (minor) = 6.8 min, t (major) = 8.7 min. HRMS (ESI-ion trap) *m/z*: $[M+H]^+$ calcd for $C_{32}H_{25}NO_2P$, 486.1617; found 486.1615.

(*R*)-(1-(Isoquinolin-1-yl)-6-methoxynaphthalen-2-yl)diphenylphosphine oxide (3ea)

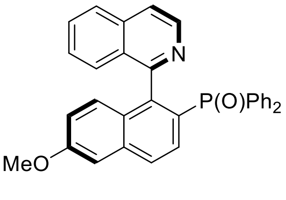

General procedure was used with triflates **1e** (43.3 mg, 0.1 mmol, 1 equiv.) and $HP(O)Ph_2$ **2a** (26.0 mg, 0.13 mmol, 1.3 equiv.) at 100 °C for 24 h to afford **3ea** as white wax (28.0 mg, 58% yield, 94% ee). $[\alpha]^{20}_{D}$ = 190.6 (c 1.0, $CHCl_3$). ^1H NMR (600 MHz, $CDCl_3$) δ 8.36 (s, 1H), 8.12-8.00 (m, 1H), 8.00-7.92 (m, 1H), 7.89-7.75 (m, 2H), 7.63-7.58 (m, 1H), 7.54-7.33 (m, 5H), 7.28-7.15 (m, 3H), 7.13-7.02 (m, 2H), 7.00-6.92 (m,

1H), 6.92-6.86 (m, 1H), 6.89-6.81 (m 2H), 6.78-6.72 (m, 1H), 3.91 (s, 3H). ^{13}C NMR (150 MHz, $CDCl_3$) δ 159.1, 158.3 (d, *J* = 4.7 Hz), 141.3, 141.2, 136.5, 135.4, 132.4 (d, *J* = 8.2 Hz), 131.5 (d, *J* = 2.2 Hz), 131.0 (d, *J* = 9.8 Hz), 130.3, 129.9, 129.8, 129.2 (d, *J* = 9.8 Hz), 129.0, 128.5, 128.1 (d, *J* = 12.4 Hz), 128.0, 127.6, 127.5, 127.3, 127.2, 127.1, 127.0, 126.6, 126.4, 121.1, 119.9, 105.8, 55.4. ^{31}P NMR (240 MHz, $CDCl_3$) δ 30.5. The enantiomeric excess was determined by Daicel Chiralcel AD (0.46 cm x 25 cm), Hexanes / IPA = 60 / 40, 1.0 mL/min, λ = 254 nm, t (minor) = 8.0 min, t (major) = 12.7 min. HRMS (ESI-ion trap) *m/z*: $[M+H]^+$ calcd for $C_{32}H_{25}NO_2P$, 486.1617; found 486.1615.

(*R*)-Methyl 6-(diphenylphosphoryl)-5-(isoquinolin-1-yl)-2-naphthoate (3fa)

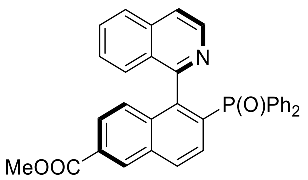

General procedure was used with triflates **1f** (46.1 mg, 0.1 mmol, 1 equiv.) and $HP(O)Ph_2$ **2a** (26.0mg, 0.13 mmol, 1.3 equiv.) at 100 °C for 6 h to afford **3fa** as white wax (48.0 mg, 93% yield, 93% ee). $[\alpha]^{20}_D$ = 148.4 (c 1.0, $CHCl_3$). ^{1}H NMR (600 MHz, $CDCl_3$) δ 8.68 (s, 1H), 8.38 (d, *J* = 5.3 Hz, 1H), 8.21-8.14 (m, 2H), 7.89-7.76 (m, 3H), 7.66-7.60 (m, 1H), 7.51 (t, *J* = 7.2 Hz, 1H), 7.49-7.43 (m, 2H), 7.42-7.35 (m, 2H), 7.23 (t, *J* = 7.5 Hz, 1H), 7.15-7.04 (m, 3H), 7.01-6.95 (m, 1H), 6.92 (d, *J* = 8.1 Hz, 1H), 6.87-6.79 (m, 2H), 3.92 (s, 3H). ^{13}C NMR (150 MHz, $CDCl_3$) δ 166.5, 157.5 (d, *J* = 4.7 Hz), 141.4, 141.3, 135.4, 134.5 (d, *J* = 11.0 Hz), 133.8, 132.4 (d, *J* = 98.7 Hz), 132.3 (d, *J* = 10.0 Hz), 131.7, 131.6, 131.0 (d, *J* = 10.6 Hz), 130.8, 130.5, 130.0, 129.9 (d, *J* = 11.1 Hz), 129.3 (d, *J* = 9.7 Hz), 129.0 (d, *J* = 39.0 Hz), 128.2 (d, *J* = 12.8 Hz), 127.3, 127.2, 127.2, 127.2, 126.5, 126.3, 121.3, 52.3. ^{31}P NMR (240 MHz, $CDCl_3$) δ 30.2. The enantiomeric excess was determined by Daicel Chiralcel AD (0.46 cm x 25 cm), Hexanes / IPA = 80 / 20, 1.0 mL/min, λ = 254 nm, t (minor) = 24.4 min, t (major) = 26.8 min. HRMS (ESI-ion trap) *m/z*: $[M+H]^+$ calcd for $C_{33}H_{25}NO_3P$, 514.1567; found 514.1566.

(*R*)-(1-(6-Isopropylisoquinolin-1-yl)naphthalen-2-yl)diphenylphosphine oxide (3ga)

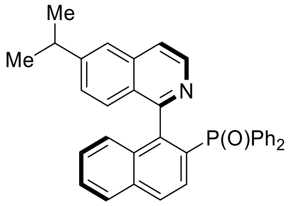

General procedure was used with triflates **1g** (44.5 mg, 0.1 mmol, 1 equiv.) and HP(O)Ph$_2$ **2a** (26.0 mg, 0.13 mmol, 1.3 equiv.) at 100 °C for 12 h to afford **3ga** as white wax (30.0 mg, 60% yield, 85% ee). $[\alpha]^{20}_{D}$ = 203.8 (c 0.5, CHCl$_3$). ^{1}H NMR (600 MHz, CDCl$_3$) δ 8.37-8.30 (m, 1H), 8.23-8.15 (m, 1H), 8.11-8.04 (m, 1H), 7.97-7.91 (m, 1H), 7.92-7.82 (m, 2H), 7.55-7.50 (m, 1H), 7.49-7.43 (m, 1H), 7.43-7.35 (m, 4H), 7.27-7.21 (m, 1H), 7.13-7.00 (m, 4H), 6.95-6.85 (m, 2H), 6.80-6.71 (m, 2H), 3.06-2.92 (m, 1H), 1.30 (t, *J* = 6.8 Hz, 6H).^{13}C NMR (150 MHz, CDCl$_3$) δ 157.8 (d, *J* = 4.7 Hz), 150.8, 141.5, 141.4, 135.7, 134.7, 132.9, 132.6, 132.5, 132.4, 132.2, 132.1, 131.5, 130.9 (d, *J* = 10.3 Hz), 130.1, 130.1, 129.4, 128.6 (d, *J* = 11.5 Hz), 128.4 (d, *J* = 9.6 Hz), 128.1, 128.0, 127.9, 127.8, 127.4, 127.1, 127.0, 127.0, 122.6, 121.0, 34.3, 23.7, 23.6. ^{31}P NMR (240 MHz, CDCl$_3$) δ 30.4. The enantiomeric excess was determined by Daicel Chiralcel AD (0.46 cm x 25 cm), Hexanes / IPA = 70 / 30, 1.0 mL/min, λ = 254 nm, t (minor) = 6.5 min, t (major) = 10.9 min. HRMS (ESI-ion trap) *m/z*: [M+H]$^+$ calcd for $C_{34}H_{29}NOP$, 498.1981; found 498.1983.

(*R*)-Diphenyl(1-(4-phenylisoquinolin-1-yl)naphthalen-2-yl)phosphine oxide (3ha)

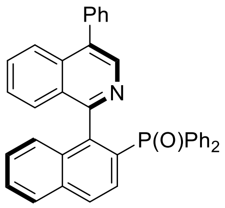

General procedure was used with triflates **1h** (47.9 mg, 0.1 mmol, 1 equiv.) and HP(O)Ph$_2$ **2a** (26.0 mg, 0.13 mmol, 1.3 equiv.) at 100 °C for 6 h to afford **3ha** as white wax (46.7 mg, 88% yield, 94% ee). $[\alpha]^{20}_{D}$ = 164.6 (c 1.0, CHCl$_3$). ^{1}H NMR (600 MHz, CDCl$_3$) δ 8.33 (s, 1H), 8.27-8.21 (m, 1H), 8.11 (d, *J* = 8.7 Hz, 1H), 7.98-7.92 (m, 3H),

7.76 (d, J = 8.4 Hz, 1H), 7.58-7.51 (m, 5H), 7.51-7.45 (m, 3H), 7.42-7.38 (m, 2H), 7.33-7.26 (m, 3H), 7.12-7.07 (m, 2H), 7.00-6.92 (m, 2H), 6.82-6.77 (m, 2H). ^{13}C NMR (150 MHz, $CDCl_3$) δ 157.6 (d, J = 5.2 Hz), 141.2, 141.2 (d, J = 9.6 Hz), 136.9, 134.8 (d, J = 1.8 Hz), 133.4, 133.1, 132.9, 132.7, 132.6, 132.5 (d, J = 10.1 Hz), 132.3 (d, J = 6.1 Hz), 131.6, 131.5 (d, J = 2.0 Hz), 131.0 (d, J = 10.6 Hz), 130.6, 130.2, 130.0, 129.9, 128.9, 128.8, 128.6, 128.5, 128.1, 128.1, 128.0, 127.9, 127.9, 127.8, 127.1, 127.0, 126.9, 126.8, 124.5. ^{31}P NMR (240 MHz, $CDCl_3$) δ 30.1. The enantiomeric excess was determined by Daicel Chiralcel AD (0.46 cm x 25 cm), Hexanes / IPA = 70 / 30, 1.0 mL/min, λ = 230 nm, t (minor) = 6.9 min, t (major) = 8.5 min. HRMS (ESI-ion trap) *m/z*: $[M+H]^+$ calcd for $C_{37}H_{27}NOP$, 532.1825; found 532.1821.

(*R*)-Diphenyl(1-(5-phenylisoquinolin-1-yl)naphthalen-2-yl)phosphine oxide (3ia)

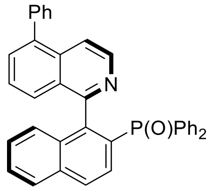

General procedure was used with triflates **1i** (47.9 mg, 0.1 mmol, 1 equiv.) and $HP(O)Ph_2$ **2a** (26.0 mg, 0.13 mmol, 1.3 equiv.) at 100 °C for 12 h to afford **3ia** as white wax (37.4 mg, 84% yield, 95% ee). $[\alpha]^{20}_D$ = 244.3 (c 1.0, $CHCl_3$). ^{1}H NMR (600 MHz, $CDCl_3$) δ 8.39-8.34 (m, 1H), 8.27-8.20 (m, 1H), 8.16-8.11 (m, 1H), 7.98 (d, J = 8.8 Hz, 1H), 7.96-7.89 (m, 2H), 7.60-7.54 (m, 4H), 7.53-7.47 (m, 5H), 7.45-7.41 (m, 2H), 7.33-7.27 (m, 2H), 7.26-7.22 (m, 1H), 7.16-7.09 (m, 2H), 7.05-7.99 (m, 1H), 6.94 (d, J = 9.3 Hz, 1H), 6.90-6.79 (m, 2H).^{13}C NMR (150 MHz, $CDCl_3$) δ 158.5 (d, J = 4.8 Hz), 141.7, 141.5 (d, J = 9.1 Hz), 139.2, 138.9, 134.8, 133.5, 132.9, 132.6 (d, J = 10.7 Hz), 132.5 (d, J = 10.0 Hz), 132.2, 131.5, 131.1 (d, J = 10.1 Hz), 130.5, 130.2, 130.1, 129.8, 129.5, 129.3, 128.8 (d, J = 11.9 Hz), 128.5, 128.4, 128.1, 128.1, 127.9, 127.7, 127.1, 127.1, 127.0, 127.0, 126.4, 119.0. ^{31}P NMR (240 MHz, $CDCl_3$) δ 30.4. The enantiomeric excess was determined by Daicel Chiralcel AD (0.46 cm x 25 cm), Hexanes / IPA = 70 / 30, 1.0 mL/min, λ = 230 nm, t (minor) = 8.5 min, t (major) = 17.1 min. HRMS (ESI-ion trap) *m/z*: $[M+H]^+$ calcd for $C_{37}H_{27}NOP$, 532.1825; found 532.1821.

(*R*)-Diphenyl(1-(6-phenylisoquinolin-1-yl)naphthalen-2-yl)phosphine oxide (3ja)

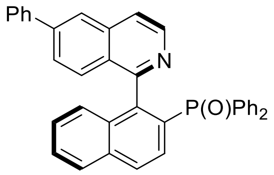

General procedure was used with triflates **1j** (47.9 mg, 0.1 mmol, 1 equiv.) and HP(O)Ph_2 **2a** (26.0 mg, 0.13 mmol, 1.3 equiv.) at 100 °C for 12 h to afford **3ja** as white wax (34.2 mg, 64% yield, 91% ee). $[\alpha]^{20}_D$ = 186.8 (c 1.0, $CHCl_3$). ^1H NMR (600 MHz, $CDCl_3$) δ 8.41 (d, *J* = 5.6 Hz, 1H), 8.19-8.12 (m, 1H), 8.12-8.08 (m, 1H), 7.96 (d, *J* = 8.0 Hz, 1H), 7.89-7.83 (m, 2H), 7.80 (s, 1H), 7.64 (d, *J* = 7.7 Hz, 2H), 7.54 (t, *J* = 7.2 Hz, 1H), 7.52-7.45 (m, 5H), 7.44-7.38 (m, 3H), 7.30-7.22 (m, 2H), 7.15-7.09 (m, 2H), 7.01-6.95 (m, 2H), 6.88-6.80 (m, 2H). ^{13}C NMR (150 MHz, $CDCl_3$) δ 158.1 (d, *J* = 4.8 Hz), 142.6, 141.9, 141.4 (d, *J* = 9.3 Hz), 140.1, 135.7, 134.7 (d, *J* = 1.8 Hz), 132.9, 132.6 (d, *J* = 10.6 Hz), 132.4 (d, *J* = 10.2 Hz), 132.3, 132.2, 131.6, 131.5 (d, *J* = 2.8 Hz), 131.0 (d, *J* = 10.2 Hz), 130.3, 130.1, 129.4, 129.0, 128.7 (d, *J* = 11.5 Hz), 128.4 (d, *J* = 9.8 Hz), 128.2-127.9 (m), 127.4, 127.2, 127.1, 126.9, 126.8, 124.1, 121.3. ^{31}P NMR (240 MHz, $CDCl_3$) δ 30.1. The enantiomeric excess was determined by Daicel Chiralcel AD (0.46 cm x 25 cm), Hexanes / IPA = 70 / 30, 1.0 mL/min, λ = 254 nm, t (minor) = 14.8 min, t (major) = 20.7 min. HRMS (ESI-ion trap) *m/z*: $[M+H]^+$ calcd for $C_{37}H_{27}NOP$, 532.1825; found 532.1821.

(*R*)-Diphenyl(1-(7-phenylisoquinolin-1-yl)naphthalen-2-yl)phosphine oxide (3ka)

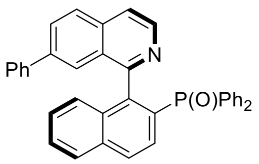

General procedure was used with triflates **1k** (47.9 mg, 0.1 mmol, 1 equiv.) and HP(O)Ph_2 **2a** (26.0 mg, 0.13 mmol, 1.3 equiv.) at 100 °C for 12 h to afford **3ka** as white wax (45.4 mg, 85% yield, 92% ee). $[\alpha]^{20}_D$ = 272.6 (c 1.0, $CHCl_3$). ^1H NMR (600 MHz, $CDCl_3$) δ 8.44 (d, *J* = 5.6 Hz, 1H), 8.27-8.20 (m, 1H), 8.13-8.07 (m, 1H), 7.97-7.86 (m, 3H), 7.79-7.74 (m, 1H), 7.71-7.67 (m, 1H), 7.53-7.48 (m, 2H), 7.43-7.43 (m, 1H), 7.41-7.34 (m, 6H), 7.33-7.28 (m, 2H), 7.27-7.20 (m, 1H), 7.14-7.06 (m, 2H), 6.95-6.86 (m,

2H), 6.82-6.73 (m, 2H).^{13}C NMR (150 MHz, $CDCl_3$) δ 158.6 (d, J = 5.0 Hz), 141.4, 114.2 (d, J = 9.0 Hz), 140.1, 139.8, 134.7 (d, J = 1.8 Hz), 134.5, 132.8, 132.5 (d, J = 11.0 Hz), 132.4 (d, J = 10.2 Hz), 132.2 (d, J = 21.9 Hz), 131.5 (d, J = 2.0 Hz), 130.9 (d, J = 10.4 Hz), 130.3 (d, J = 2.0 Hz), 130.0, 129.7, 129.3, 129.2, 128.9, 128.8, 128.4 (d, J = 9.7 Hz), 128.1 (d, J = 7.3 Hz), 128.0, 127.9, 127.6, 127.3, 127.1, 127.0, 127.0, 126.8, 125.1, 120.9. ^{31}P NMR (240 MHz, $CDCl_3$) δ 30.4. The enantiomeric excess was determined by Daicel Chiralcel AD (0.46 cm x 25 cm), Hexanes / IPA = 70 / 30, 1.0 mL/min, λ = 254 nm, t (minor) = 9.6 min, t (major) = 11.8 min. HRMS (ESI-ion trap) *m/z*: $[M+H]^+$ calcd for $C_{37}H_{27}NOP$, 532.1825; found 532.1821.

(*R*)-(1-(Isoquinolin-1-yl)-7-phenylnaphthalen-2-yl)diphenylphosphine oxide (3la)

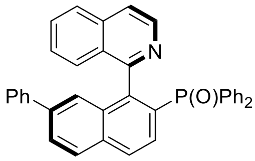

General procedure was used with triflates **1l** (47.9 mg, 0.1 mmol, 1 equiv.) and $HP(O)Ph_2$ **2a** (26.0 mg, 0.13 mmol, 1.3 equiv.) at 100 °C for 12 h to afford **3la** as white wax (40.0 mg, 75% yield, 89% ee). $[\alpha]^{20}_{D}$ = 101.7 (c 1.0, $CHCl_3$). ^{1}H NMR (600 MHz, $CDCl_3$) δ 8.42 (d, J = 5.6 Hz, 1H), 8.19-8.11 (m, 2H), 8.04 (d, J = 8.4 Hz, 1H), 7.93-7.86 (m, 2H), 7.81 (d, J = 8.5 Hz, 1H), 7.67-7.63 (m, 1H), 7.56-7.41 (m, 5H), 7.30-7.22 (m, 7H), 7.15-7.9 (m, 2H), 7.06 (s, 1H), 7.03-6.97 (m, 1H), 6.88-6.82 (m, 2H).^{13}C NMR (150 MHz, $CDCl_3$) δ 158.1 (d, J = 5.0 Hz), 141.6 (d, J = 7.8 Hz), 141.4, 140.5, 139.8, 135.3, 133.9, 133.0, 132.8 (d, J = 11.3 Hz), 132.4 (d, J = 10.2 Hz), 132.2 (d, J = 13.4 Hz), 131.5 (d, J = 2.0 Hz), 131.0 (d, J = 10.4 Hz), 130.6, 130.3, 130.0, 129.9, 129.1, 128.6-128.3 (m), 128.1 (d, J = 11.8 Hz), 127.8, 127.4, 127.3, 127.3, 127.2,127.1, 127.0, 126.5, 124.7, 121.2. ^{31}P NMR (240 MHz, $CDCl_3$) δ 30.1. The enantiomeric excess was determined by Daicel Chiralcel AD (0.46 cm x 25 cm), Hexanes / IPA = 70 / 30, 1.0 mL/min, λ = 254 nm, t (minor) = 7.1 min, t (major) = 10.8 min. HRMS (ESI-ion trap) *m/z*: $[M+H]^+$ calcd for $C_{37}H_{27}NOP$, 532.1825; found 532.1821.

(*R*)-(1-(Isoquinolin-1-yl)-6-phenylnaphthalen-2-yl)diphenylphosphine oxide (3ma)

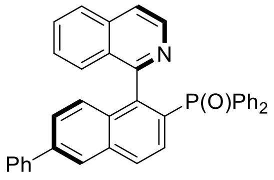

General procedure was used with triflates **1m** (47.9 mg, 0.1 mmol, 1 equiv.) and $HP(O)Ph_2$ **2a** (26.0 mg, 0.13 mmol, 1.3 equiv.) at 100 °C for 12 h to afford **3ma** as white wax (41 mg, 77% yield, 88% ee). $[\alpha]^{20}_{D}$ = 132.6 (c 1.0, $CHCl_3$). ^1H NMR (600 MHz, $CDCl_3$) δ 8.42 (d, *J* = 5.6 Hz, 1H), 8.22-8.13 (m, 3H), 7.94-7.85 (m, 2H), 7.71-7.64 (m, 3H), 7.58-7.45 (m, 7H), 7.45-7.39 (m, 3H), 7.30-7.23 (m, 2H), 7.16-7.08 (m, 2H), 7.02-6.94 (m, 2H), 6.88-6.80 (m, 2H). ^{13}C NMR (150 MHz, $CDCl_3$) δ 158.2 (d, *J* = 4.6 Hz), 141.5, 141.3 (d, *J* = 8.7 Hz), 140.5, 140.2, 135.4, 135.1 (d, *J* = 2.3 Hz), 132.9, 132.5 (d, *J* = 10.2 Hz), 132.2, 131.8, 131.7, 131.5 (d, *J* = 2.0 Hz), 131.0 (d, *J* = 10.5 Hz), 130.5 (d, *J* = 9.9 Hz), 130.3 (d, *J* = 2.6 Hz), 130.0, 129.9, 129.4, 129.0, 128.9, 128.9, 128.6 (d, *J* = 11.7 Hz), 128.1 (d, *J* = 12.6 Hz), 127.8, 127.5, 127.4, 127.2, 127.1, 127.0, 126.8, 126.5, 125.7, 121.1. ^{31}P NMR (240 MHz, $CDCl_3$) δ 30.2. The enantiomeric excess was determined by Daicel Chiralcel AD (0.46 cm x 25 cm), Hexanes / IPA = 70 / 30, 1.0 mL/min, λ = 270 nm, t (major) = 10.7 min, t (minor) = 11.9 min. HRMS (ESI-ion trap) *m/z*: $[M+H]^+$ calcd for $C_{37}H_{27}NOP$, 532.1825; found 532.1821.

(*R*)-(1-(Isoquinolin-1-yl)-4-phenylnaphthalen-2-yl)diphenylphosphine oxide (3na)

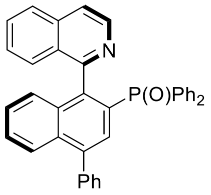

General procedure was used with triflates **1n** (47.9 mg, 0.1 mmol, 1 equiv.) and $HP(O)Ph_2$ **2a** (26.0 mg, 0.13 mmol, 1.3 equiv.) at 100 °C for 12 h to afford **3na** as white wax (51.5 mg, 96% yield, 88% ee). $[\alpha]^{20}_{D}$ = 154.5 (c 1.0, $CHCl_3$). ^1H NMR (600 MHz, $CDCl_3$) δ 8.41 (d, *J* = 5.1 Hz, 1H), 8.14 (d, *J* = 11.4 Hz, 1H), 8.02 (d, *J* = 8.3 Hz, 1H), 7.95-7.88 (m, 2H), 7.64-7.60 (m, 1H), 7.56-7.38 (m, 11H), 7.32-7.20 (m, 2H), 7.10-

7.03 (m, 2H), 6.95-6.89 (m, 2H), 6.81-6.73 (m, 2H). ^{13}C NMR (150 MHz, $CDCl_3$) δ 158.5 (d, *J* = 4.7 Hz), 141.5, 141.0 (d, *J* = 11.6 Hz), 140.6 (d, *J* = 8.7 Hz), 139.6, 135.3, 133.1 (d, *J* = 2.1 Hz), 133.0, 132.9, 132.5 (d, *J* = 10.6 Hz), 132.2 (d, *J* = 4.9 Hz), 131.5, 131.5, 131.4, 130.9 (d, *J* = 10.8 Hz), 130.2 (d, *J* = 2.0 Hz), 130.2, 129.9, 129.8, 129.2, 129.1, 128.3, 128.1, 128.0, 127.9, 127.6 (d, *J* = 6.2 Hz), 127.3, 127.1, 127.0, 126.8, 126.4, 126.3, 121.1. ^{31}P NMR (240 MHz, $CDCl_3$) δ 30.2. The enantiomeric excess was determined by Daicel Chiralcel IC (0.46 cm x 25 cm), Hexanes / IPA = 70 / 30, 1.0 mL/min, λ = 254 nm, t (major) = 16.1 min, t (minor) = 43.0 min. HRMS (ESI-ion trap) *m/z*: $[M+H]^+$ calcd for $C_{37}H_{27}NOP$, 532.1825; found 532.1821.

(*R*)-(1-(Isoquinolin-1-yl)-6-(p-tolyl)naphthalen-2-yl)diphenylphosphine oxide (3oa)

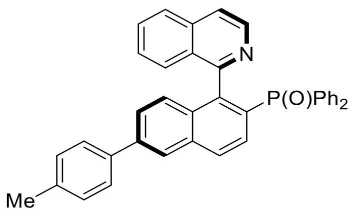

General procedure was used with triflates **1o** (49.3 mg, 0.1 mmol, 1 equiv.) and $HP(O)Ph_2$ **2a** (26.0 mg, 0.13 mmol, 1.3 equiv.) at 100 °C for 12 h to afford **3oa** as white wax (50.7 mg, 93% yield, 92% ee). $[\alpha]^{20}_D$ = 92.9 (c 1.0, $CHCl_3$). ^{1}H NMR (600 MHz, $CDCl_3$) δ 8.40 (d, *J* = 5.8 Hz, 1H), 8.17-8.08 (m, 3H), 7.90-7.82 (m, 2H), 7.67-7.62 (m, 1H), 7.58-7.45 (m, 6H), 7.43-7.38 (m, 2H), 7.29-7.22 (m, 4H), 7.13-7.08 (m, 2H), 7.00-6.95 (m, 1H), 6.93 (d, *J* = 9.0, Hz, 1H), 6.87-6.81 (m, 2H), 2.39 (s, 3H). ^{13}C NMR (150 MHz, $CDCl_3$) δ 158.2 (d, *J* = 4.6 Hz), 141.3, 141.2 (d, *J* = 9.2 Hz), 140.5, 137.7, 137.2, 135.4, 135.1 (d, *J* = 1.8 Hz), 132.8, 132.5, 132.4, 132.1, 131.6, 131.5, 131.5, 131.4, 131.0 (d, *J* = 10.5 Hz), 130.4, 130.0, 129.7, 129.6, 129.0, 129.0, 128.9, 128.9, 128.8, 128.8, 128.8, 128.1 (d, *J* = 12.6 Hz), 127.4 (d, *J* = 19.8 Hz), 127.1 (d, *J* = 20.6 Hz), 127.1, 126.7, 126.4, 125.3, 121.2, 21.1. ^{31}P NMR (240 MHz, $CDCl_3$) δ 30.5. The enantiomeric excess was determined by Daicel Chiralcel IA (0.46 cm x 25 cm), Hexanes / IPA = 70 / 30, 1.0 mL/min, λ = 254 nm, t (minor) = 9.8 min, t (major) = 12.1 min. HRMS (ESI-ion trap) *m/z*: $[M+H]^+$ calcd for $C_{38}H_{29}NOP$, 546.1981; found 546.1978.

(*R*)-(6-(3,5-Dimethylphenyl)-1-(isoquinolin-1-yl)naphthalen-2-yl)diphenylphosphine oxide (3pa)

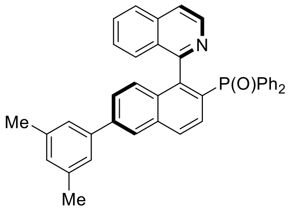

General procedure was used with triflates **1p** (50.7 mg, 0.1 mmol, 1 equiv.) and HP(O)Ph2 **2a** (26.0 mg, 0.13 mmol, 1.3 equiv.) at 100 °C for 12 h to afford **3pa** as white wax (50.2 mg, 90% yield, 91% ee). $[\alpha]^{20}_{D}$ = 104.5 (c 1.0, $CHCl_3$). ^1H NMR (600 MHz, $CDCl_3$) δ 8.40 (d, *J* = 5.6 Hz, 1H), 8.18-8.10 (m, 3H), 7.91-7.82 (m, 2H), 7.67-7.61 (m, 1H), 7.55-7.51 (m, 1H), 7.51-7.44 (m, 3H), 7.44-7.37 (m, 2H), 7.30-7.22 (m, 4H), 7.15-7.08 (m, 2H), 7.02 (s, 1H), 7.01-6.96 (m, 1H), 6.93 (d, *J* = 9.4, Hz, 1H), 6.87-6.81 (m, 2H), 2.38 (s, 6H). ^{13}C NMR (150 MHz, $CDCl_3$) δ 158.2 (d, *J* = 5.6 Hz), 141.3, 141.2 (d, *J* = 7.6 Hz), 140.8, 140.1, 138.4, 135.4, 135.0 (d, *J* = 2.3 Hz), 132.8, 132.5, 132.4, 132.1, 131.6 (d, *J* = 12.7 Hz), 131.5 (d, *J* = 2.0 Hz), 131.4, 131.0 (d, *J* = 10.7 Hz), 130.4 (d, *J* = 2.3 Hz), 129.9, 129.6, 129.4, 129.0, 129.0, 128.9 (d, *J* = 11.3 Hz), 128.7 (d, *J* = 9.8 Hz), 128.1 (d, *J* = 12.6 Hz), 127.5, 127.3, 127.2, 127.1, 127.0, 126.9, 126.4, 125.6, 125.2, 121.2, 21.3. ^{31}P NMR (240 MHz, $CDCl_3$) δ 30.5. The enantiomeric excess was determined by Daicel Chiralcel IA (0.46 cm x 25 cm), Hexanes / IPA = 60 / 40, 1.0 mL/min, λ = 254 nm, t (minor) = 6.0 min, t (major) = 7.5 min. HRMS (ESI-ion trap) *m/z*: $[M+H]^+$ calcd for $C_{39}H_{31}NOP$, 560.2138; found 560.2139.

(*R*)-(1-(Isoquinolin-1-yl)-6-(4-methoxyphenyl)naphthalen-2-yl)diphenylphosphine oxide (3qa)

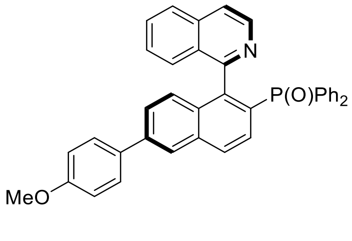

General procedure was used with triflates **1q** (50.9 mg, 0.1 mmol, 1 equiv.) and HP(O)Ph2 **2a** (26.0 mg, 0.13 mmol, 1.3 equiv.) at 100 °C for 12 h to afford **3qa** as white

wax (47.0 mg, 84% yield, 91% ee). $[\alpha]^{20}_{D}$ = 93.9 (c 1.0, $CHCl_3$). ^{1}H NMR (600 MHz, $CDCl_3$) δ 8.40 (d, *J* = 5.5, Hz, 1H), 8.15-8.05 (m, 3H), 7.88-7.82 (m, 2H), 7.66-7.62 (m, 1H), 7.59 (d, *J* = 8.4, Hz, 2H), 7.54-7.50 (m, 1H), 7.50-7.43 (m, 3H), 7.41-7.35 (s, 2H), 7.27-7.21 (m, 2H), 7.15-7.08 (m, 2H), 7.01-6.96 (m, 3H), 6.91 (d, *J* = 9.0, Hz, 1H), 6.87-6.81 (m, 2H), 3.83 (s, 3H). ^{13}C NMR (150 MHz, $CDCl_3$) δ 159.5, 158.1 (d, *J* = 4.9 Hz), 141.3, 141.2 (d, *J* = 9.0 Hz), 140.1, 135.4, 135.1 (d, *J* = 2.0 Hz), 132.7, 132.5, 132.4 (d, *J* = 9.7 Hz), 132.0 (d, *J* = 8.1 Hz), 131.5, 131.4, 131.3, 131.0 (d, *J* = 10.1 Hz), 130.4 (d, *J* = 2.0 Hz), 130.0, 129.5, 129.0, 128.8, 128.7, 128.3, 128.1 (d, *J* = 12.3 Hz), 127.4 (d, *J* = 17.0 Hz), 127.2 (d, *J* = 12.0 Hz), 127.0, 126.5 (d, *J* = 18.9 Hz), 124.8, 121.1, 114.3, 55.3. ^{31}P NMR (240 MHz, $CDCl_3$) δ 30.5. The enantiomeric excess was determined by Daicel Chiralcel AD (0.46 cm x 25 cm), Hexanes / IPA = 70 / 30, 1.0 mL/min, λ = 230 nm, t (major) = 14.9 min, t (minor) = 19.6 min. HRMS (ESI-ion trap) *m/z*: $[M+H]^+$ calcd for $C_{38}H_{29}NO_2P$, 562.1930; found 562.1932.

(*R*)-(6-(4-Fluorophenyl)-1-(isoquinolin-1-yl)naphthalen-2-yl)diphenylphosphine oxide (3ra)

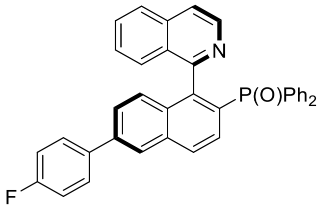

General procedure was used with triflates **1r** (49.7 mg, 0.1 mmol, 1 equiv.) and $HP(O)Ph_2$ **2a** (26.0 mg, 0.13 mmol, 1.3 equiv.) at 100 ºC for 12 h to afford **3ra** as white wax (52.8 mg, 96% yield, 90% ee). $[\alpha]^{20}_{D}$ = 104.9 (c 1.0, $CHCl_3$). ^{1}H NMR (600 MHz, $CDCl_3$) δ 8.40 (d, *J* = 5.6 Hz, 1H), 8.16-8.05 (m, 3H), 7.88-7.80 (m, 2H), 7.67-7.63 (m, 1H), 7.63-7.56 (m, 2H), 7.55-7.51 (m, 1H), 7.49-7.42 (m, 3H), 7.41-7.35 (m, 2H), 7.28-7.19 (m, 2H), 7.17-7.08 (m, 4H), 7.01-6.96 (m, 1H), 6.93 (d, *J* = 9.0 Hz, 1H), 6.88-6.81 (m, 2H). ^{13}C NMR (150 MHz, $CDCl_3$) δ 163.5, 161.9, 158.0 (d, *J* = 4.6 Hz), 141.2, 141.1, 139.5, 136.2 (d, *J* = 3.1 Hz), 135.4, 135.0 (d, *J* = 2.0 Hz), 132.6, 132.3 (d, *J* = 10.0 Hz), 132.0 (d, *J* = 9.6 Hz), 131.6, 131.6, 131.3, 131.0 (d, *J* = 10.7 Hz), 130.5 (d, *J* = 1.9 Hz), 130.1, 130.0, 129.3, 129.0, 128.9, 128.8, 128.1 (d, *J* = 13.3 Hz), 127.5 (d, *J*

= 16.6 Hz), 127.2 (d, J = 12.3 Hz), 127.1, 126.6, 126.5, 125.5, 121.3. ^{31}P NMR (240 MHz, $CDCl_3$) δ 30.4. ^{19}F NMR (564 MHz, $CDCl_3$) δ -114.5. The enantiomeric excess was determined by Daicel Chiralcel IA (0.46 cm x 25 cm), Hexanes / IPA = 60 / 40, 1.0 mL/min, λ = 254 nm, t (minor) = 9.2 min, t (major) = 11.8 min. HRMS (ESI-ion trap) *m/z*: $[M+H]^+$ calcd for $C_{37}H_{26}FNOP$, 550.1731; found 550.1732.

(*R*)-(5-(Isoquinolin-1-yl)-[2,2'-binaphthalen]-6-yl)diphenylphosphine oxide (3sa)

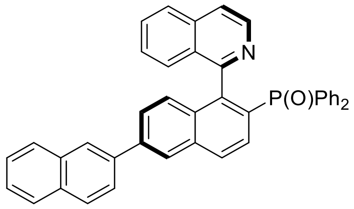

General procedure was used with triflates **1s** (52.9 mg, 0.1 mmol, 1 equiv.) and $HP(O)Ph_2$ **2a** (26.0 mg, 0.13 mmol, 1.3 equiv.) at 100 °C for 12 h to afford **3sa** as white wax (47.0 mg, 81% yield, 90% ee). $[\alpha]^{20}_D$ = 80.9 (c 1.0, $CHCl_3$). ^{1}H NMR (600 MHz, $CDCl_3$) δ 8.43 (d, J = 5.8, Hz, 1H), 8.26 (s, 1H), 8.18-8.13 (m, 2H), 8.10 (s, 1H), 7.94-7.90 (m, 1H), 7.90-7.83 (m, 4H), 7.81-7.77 (m, 1H), 7.68-7.61 (m, 2H), 7.56-7.45 (m, 5H), 7.43-7.37 (m, 2H), 7.29-7.23 (m, 2H), 7.17-7.10 (m, 2H), 7.03-6.97 (m, 2H), 6.90-6.83 (m, 2H). ^{13}C NMR (150 MHz, $CDCl_3$) δ 158.1 (d, J = 4.4 Hz), 141.2, 141.1, 140.4, 137.3, 135.4, 135.1, 133.5, 132.8, 132.7, 132.4 (d, J = 10.0 Hz), 132.0 (d, J = 11.4 Hz), 131.7 (d, J = 11.6 Hz), 131.6 (d, J = 1.9 Hz), 131.4, 131.0 (d, J = 10.0 Hz), 130.5 (d, J = 2.0 Hz), 130.1, 130.0, 129.3, 129.0, 129.0, 128.9, 128.9, 128.6, 128.2, 128.1 (d, J = 12.7 Hz), 127.6, 127.5 (d, J = 5.2Hz), 127.2 (d, J = 12.4 Hz), 127.0 (d, J = 31.0 Hz), 126.5, 126.4, 126.3, 126.2, 126.0, 125.3, 121.3. ^{31}P NMR (240 MHz, $CDCl_3$) δ 30.4. The enantiomeric excess was determined by Daicel Chiralcel AD (0.46 cm x 25 cm), Hexanes / IPA = 60 / 40, 1.0 mL/min, λ = 254 nm, t (major) = 10.5 min, t (minor) = 15.5 min. HRMS (ESI-ion trap) *m/z*: $[M+H]^+$ calcd for $C_{41}H_{29}NO_2P$, 582.1981; found 582.1982.

(*R*)-(1-(Isoquinolin-1-yl)-6-(thiophen-2-yl)naphthalen-2-yl)diphenylphosphine oxide (3ta)

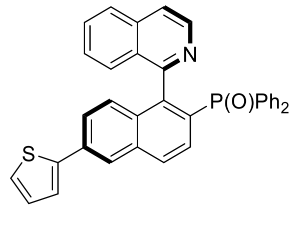

General procedure was used with triflates **1t** (48.5 mg, 0.1 mmol, 1 equiv.) and $HP(O)Ph_2$ **2a** (26.0 mg, 0.13 mmol, 1.3 equiv.) at 100 °C for 12 h to afford **3ta** as white wax (40.0 mg, 74% yield, 93% ee). $[\alpha]^{20}_D$ = 92.6 (c 1.0, $CHCl_3$). ^1H NMR (600 MHz, $CDCl_3$) δ 8.39 (d, *J* = 5.9, Hz, 1H), 8.15-8.10 (m, 2H), 8.10-8.06 (m, 1H), 7.89-7.81 (m, 2H), 7.66-7.61 (m, 1H), 7.54-7.4 (m, 4H), 7.43-7.37 (m, 3H), 7.33-7.29 (s, 1H), 7.27-7.23 (m, 1H), 7.23-7.19 (m, 1H), 7.13-7.06 (m, 3H), 7.00-6.95 (m, 1H), 6.87 (d, *J* = 8.9, Hz, 1H), 6.95-6.80 (m, 2H). ^{13}C NMR (150 MHz, $CDCl_3$) δ 158.0 (d, *J* = 4.8 Hz), 143.3, 141.3, 141.3 (d, *J* = 9.1 Hz), 135.4, 135.0 (d, *J* = 2.0 Hz), 133.7, 132.7, 132.4, 132.4, 132.0, 131.7 (d, *J* = 11.7 Hz), 131.6 (d, *J* = 2.1 Hz), 131.3, 131.0 (d, *J* = 10.2 Hz), 130.4 (d, *J* = 2.6 Hz), 130.0, 129.9, 129.2, 129.0 (d, *J* = 24.8 Hz), 128.7 (d, *J* = 11.9 Hz), 128.2, 128.1 (d, *J* = 11.8 Hz), 127.6, 127.4, 127.2, 127.1 (d, *J* = 8.0 Hz), 126.4, 125.8, 125.5, 124.2, 123.9, 121.2. ^{31}P NMR (240 MHz, $CDCl_3$) δ 30.4. The enantiomeric excess was determined by Daicel Chiralcel IA (0.46 cm x 25 cm), Hexanes / IPA = 70 / 30, 1.0 mL/min, λ = 254 nm, t (minor) = 12.0 min, t (major) = 15.3 min. HRMS (ESI-ion trap) *m/z*: $[M+H]^+$ calcd for $C_{35}H_{25}NOPS$, 538.1389; found 538.1393.

(*R*)-Diphenyl(1-(6-(p-tolyl)isoquinolin-1-yl)naphthalen-2-yl)phosphine oxide (3ua)

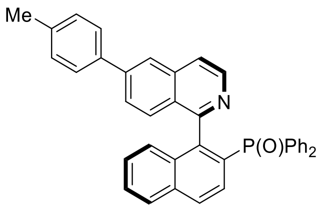

General procedure was used with triflates **1u** (49.3 mg, 0.1 mmol, 1 equiv.) and $HP(O)Ph_2$ **2a** (26.0 mg, 0.13 mmol, 1.3 equiv.) at 100 °C for 24 h to afford **3ua** as white wax (52.9 mg, 97% yield, 87% ee). $[\alpha]^{20}_D$ = 148.5 (c 1.0, $CHCl_3$). ^1H NMR (600 MHz,

$CDCl_3$) δ 8.40 (d, *J* = 5.6 Hz, 1H), 8.18-8.13 (m, 1H), 8.11-8.08 (m, 1H), 7.96 (d, *J* = 8.1 Hz, 1H), 7.90-7.83 (m, 2H), 7.78 (s, 1H), 7.57-7.52 (m, 3H), 7.50-7.45 (m, 3H), 7.42-7.38 (m, 2H), 7.31 (d, *J* = 8.0 Hz, 2H), 7.29-7.25 (m, 1H), 7.24-7.21 (m, 1H), 7.15-7.09 (m, 2H), 7.00-6.95 (m, 2H), 6.86-6.82 (m, 2H), 2.42 (s, 3H). ^{13}C NMR (150 MHz, $CDCl_3$) δ 158.0 (d, *J* = 5.0 Hz), 142.5, 141.7, 141.4 (d, *J* = 9.3 Hz), 138.1, 137.1, 135.8, 134.7, 132.8, 132.5 (d, *J* = 11.7 Hz), 132.5, 132.4, 132.2 (d, *J* = 10.0 Hz), 131.5, 131.0 (d, *J* = 10.2 Hz), 130.3 (d, *J* = 2.7 Hz), 130.0, 129.7, 129.3, 128.7 (d, *J* = 11.2 Hz), 128.4 (d, *J* = 9.8 Hz), 128.1, 128.1, 128.0, 128.0, 127.9, 127.2, 127.1, 126.8 (d, *J* = 25.3 Hz), 123.7, 121.3, 21.1. ^{31}P NMR (240 MHz, $CDCl_3$) δ 30.4. The enantiomeric excess was determined by Daicel Chiralcel AD (0.46 cm x 25 cm), Hexanes / IPA = 60 / 40, 1.0 mL/min, λ = 254 nm, t (minor) = 10.8 min, t (major) = 12.4 min. HRMS (ESI-ion trap) *m/z*: $[M+H]^+$ calcd for $C_{38}H_{29}NOP$, 546.1981; found 546.1978.

(*R*)-(1-(6-(3,5-Dimethylphenyl)isoquinolin-1-yl)naphthalen-2-yl)diphenylphosphine oxide (3va)

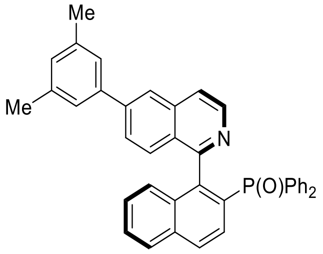

General procedure was used with triflates **1v** (50.7 mg, 0.1 mmol, 1 equiv.) and $HP(O)Ph_2$ **2a** (26.0 mg, 0.13 mmol, 1.3 equiv.) at 100 °C for 24 h to afford **3va** as white wax (49.7 mg, 89% yield, 88% ee). $[\alpha]^{20}_D$ = 169.9 (c 1.0, $CHCl_3$). ^1H NMR (600 MHz, $CDCl_3$) δ 8.41 (d, *J* = 5.4 Hz, 1H), 8.19-8.13 (m, 1H), 8.12-8.08 (m, 1H), 7.96 (d, *J* = 8.6 Hz, 1H), 7.90-7.84 (m, 2H), 7.79 (s, 1H), 7.54 (t, *J* = 6.9 Hz, 1H), 7.51-7.45 (m, 3H), 7.44-7.38 (s, 2H), 7.30-7.24 (m, 1H), 7.22 (d, *J* = 8.0 Hz, 1H), 7.16-7.10 (m, 2H), 7.07 (s, 1H), 7.02-6.97 (m, 1H), 6.96 (d, *J* = 8.7 Hz, 1H), 6.88-6.81 (m, 2H), 2.42 (s, 6H). ^{13}C NMR (150 MHz, $CDCl_3$) δ 158.0 (d, *J* = 5.1 Hz), 143.0, 141.6, 141.3 (d, *J* = 9.2 Hz), 140.0, 138.5, 135.8, 134.7, 132.7, 132.6, 132.5, 132.4 (d, *J* = 10.0 Hz), 132.1 (d, *J* = 6.9 Hz), 131.5 (d, *J* = 2.0 Hz), 131.4, 131.1 (d, *J* = 10.1 Hz), 130.4, 130.0, 129.8,

129.3, 128.8 (d, J = 11.4 Hz), 128.4 (d, J = 10.4 Hz), 128.1 (d, J = 15.7 Hz), 128.1, 127.9 (d, J = 16.1 Hz), 127.2, 127.1, 127.0 (d, J = 11.2 Hz), 125.3, 124.0, 121.4, 21.4. ^{31}P NMR (240 MHz, $CDCl_3$) δ 30.6. The enantiomeric excess was determined by Daicel Chiralcel AD (0.46 cm x 25 cm), Hexanes / IPA = 60 / 40, 1.0 mL/min, λ = 254 nm, t (minor) = 5.5 min, t (major) = 11.2 min. HRMS (ESI-ion trap) *m/z*: $[M+H]^+$ calcd for $C_{39}H_{31}NOP$, 560.2138; found 560.2139.

(*R*)-(1-(6-(4-Methoxyphenyl)isoquinolin-1-yl)naphthalen-2-yl)diphenylphosphine oxide (3wa)

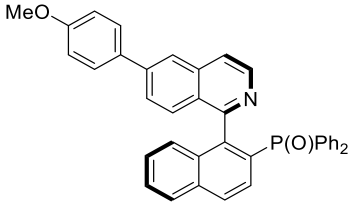

General procedure was used with triflates **1w** (50.9 mg, 0.1 mmol, 1 equiv.) and $HP(O)Ph_2$ **2a** (26.0 mg, 0.13 mmol, 1.3 equiv.) at 100 °C for 24 h to afford **3wa** as white wax (39.0 mg, 70% yield, 94% ee). $[\alpha]^{20}_D$ = 186.3 (c 1.0, $CHCl_3$). ^{1}H NMR (600 MHz, $CDCl_3$) δ 8.39 (d, J = 5.4 Hz, 1H), 8.15-8.07 (m, 2H), 7.95 (d, J = 8.1 Hz, 1H), 7.87-7.81 (m, 2H), 7.76 (s, 1H), 7.59 (d, J = 7.6 Hz, 2H), 7.54 (t, J = 7.3 Hz, 1H), 7.50-7.43 (m, 3H), 7.42-7.37 (m, 2H), 7.30-7.25 (m, 1H), 7.23-7.19 (m, 1H), 7.17-7.10 (m, 2H), 7.06-6.98 (m, 3H), 6.98-6.94 (m, 1H), 6.90-6.82 (m, 2H), 3.87 (s, 3H). ^{13}C NMR (150 MHz, $CDCl_3$) δ 159.9, 157.9 (d, J = 4.5 Hz), 142.3, 141.6, 141.3 (d, J = 8.8 Hz), 135.9, 134.7, 132.7, 132.6 (d, J = 12.0 Hz), 132.4 (d, J = 10.1 Hz), 132.1 (d, J = 17.5 Hz), 131.6, 131.5, 131.1 (d, J = 10.5 Hz), 130.4, 130.0, 129.3, 128.8 (d, J = 10.8 Hz), 128.5, 128.4 (d, J = 10.0 Hz), 128.1 (d, J = 15.0 Hz), 128.1, 128.0 (d, J = 4.2 Hz), 127.9, 127.3 (d, J = 12.8 Hz), 127.2, 126.8 (d, J = 35.8 Hz), 123.3, 121.3, 114.5, 55.3. ^{31}P NMR (240 MHz, $CDCl_3$) δ 30.4. The enantiomeric excess was determined by Daicel Chiralcel AD (0.46 cm x 25 cm), Hexanes / IPA = 60 / 40, 1.0 mL/min, λ = 254 nm, t (minor) = 12.3 min, t (major) = 17.2 min. HRMS (ESI-ion trap) *m/z*: $[M+H]^+$ calcd for $C_{38}H_{29}NO_2P$, 562.1930; found 562.1924.

(*R*)-(1-(6-(4-Fluorophenyl)isoquinolin-1-yl)naphthalen-2-yl)diphenylphosphine oxide (3xa)

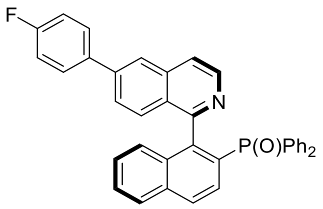

General procedure was used with triflates **1x** (49.7 mg, 0.1 mmol, 1 equiv.) and HP(O)Ph2 **2a** (26.0 mg, 0.13 mmol, 1.3 equiv.) at 100 °C for 24 h to afford **3xa** as white wax (45.0 mg, 82% yield, 94% ee). $[\alpha]^{20}_{D}$ = 186.4 (c 1.0, $CHCl_3$). ^1H NMR (600 MHz, $CDCl_3$) δ 8.40 (d, *J* = 5.7 Hz, 1H), 8.11-8.05 (m, 2H), 7.96 (d, *J* = 7.9 Hz, 1H), 7.86-7.80 (m, 2H), 7.77 (s, 1H), 7.63-7.58 (m, 2H), 7.54 (t, *J* = 7.4 Hz, 1H), 7.51 (d, *J* = 5.7 Hz, 1H), 7.49-7.45 (m, 1H), 7.44-7.37 (m, 3H), 7.30-7.23 (m, 1H), 7.21-7.12 (m, 4H), 7.03 (t, *J* = 7.0 Hz, 1H), 6.96 (d, *J* = 9.1 Hz, 1H), 6.92-6.86 (m, 2H). ^{13}C NMR (150 MHz, $CDCl_3$) δ 162.9 (d, *J* = 248.6 Hz), 158.1 (d, *J* = 5.2 Hz), 141.9, 141.6, 141.4 (d, *J* = 8.4 Hz), 136.2 (d, *J* = 3.2 Hz), 135.7, 134.7 (d, *J* = 2.1 Hz), 132.7 (d, *J* = 24.8 Hz), 132.5, 132.4, 132.3, 132.2 (d, *J* = 29.4 Hz), 131.6, 131.2, 131.1, 130.5 (d, *J* = 3.0 Hz), 129.9, 129.2, 129.1, 129.0, 128.7 (d, *J* = 11.8 Hz), 128.4 (d, *J* = 10.0 Hz), 128.2 (d, *J* = 4.1 Hz), 128.1 (d, *J* = 3.2 Hz), 128.0, 127.3 (d, *J* = 12.4 Hz), 127.2, 126.7 (d, *J* = 35.0 Hz), 124.0, 121.2, 115.9 (d, *J* = 20.8 Hz). ^{31}P NMR (240 MHz, $CDCl_3$) δ 30.3. ^{19}F NMR (564 MHz, $CDCl_3$) δ -114.0. The enantiomeric excess was determined by Daicel Chiralcel IA (0.46 cm x 25 cm), Hexanes / IPA = 60 / 40, 1.0 mL/min, λ = 270 nm, t (minor) = 10.6 min, t (major) = 12.3 min. HRMS (ESI-ion trap) *m/z*: $[M+H]^+$ calcd for $C_{37}H_{26}FNOP$, 550.1731; found 550.1732.

(*R*)-Diphenyl(1-(6-(thiophen-2-yl)isoquinolin-1-yl)naphthalen-2-yl)phosphine oxide (3ya)

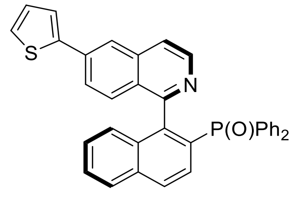

General procedure was used with triflates **1y** (48.5 mg, 0.1 mmol, 1 equiv.) and HP(O)Ph2 **2a** (26.0 mg, 0.13 mmol, 1.3 equiv.) at 100 °C for 24 h to afford **3ya** as white wax (36.0 mg, 67% yield, 86% ee). $[\alpha]^{20}_{D}$ = 181.6 (c 1.0, $CHCl_3$). ^{1}H NMR (600 MHz, $CDCl_3$) δ 8.37 (d, *J* = 5.6 Hz, 1H), 8.13-8.06 (m, 2H), 7.95 (d, *J* = 8.0 Hz, 1H), 7.87-7.79 (m, 3H), 7.54 (t, *J* = 7.1 Hz, 1H), 7.50-7.44 (m, 3H), 7.44-7.36 (m, 4H), 7.29-7.24 (m, 1H), 7.20-7.10 (m, 4H), 7.05-7.00 (m, 1H), 6.97-6.93 (m, 1H), 6.91-6.85 (m, 2H). ^{13}C NMR (150 MHz, $CDCl_3$) δ 158.0 (d, *J* = 4.5 Hz), 143.1, 142.0, 141.2 (d, *J* = 9.0 Hz), 135.9, 135.8, 134.7 (d, *J* = 2.2 Hz), 132.8, 132.5 (d, *J* = 11.4 Hz), 132.4 (d, *J* = 10.4 Hz), 132.2 (d, *J* = 28.7 Hz), 131.6, 131.1 (d, *J* = 11.0 Hz), 130.5 (d, *J* = 2.9 Hz), 130.0, 129.4, 128.8 (d, *J* = 11.9 Hz), 128.4, 128.4, 128.2 (d, *J* = 4.6 Hz), 128.1, 128.0 (d, *J* = 9.5 Hz), 127.3 (d, *J* = 12.0 Hz), 127.2, 126.8, 126.4, 125.5, 124.6, 122.3, 121.1. ^{31}P NMR (240 MHz, $CDCl_3$) δ 30.2. The enantiomeric excess was determined by Daicel Chiralcel AD (0.46 cm x 25 cm), Hexanes / IPA = 60 / 40, 1.0 mL/min, λ = 230 nm, t (minor) = 11.2 min, t (major) = 15.0 min. HRMS (ESI-ion trap) *m/z*: $[M+H]^+$ calcd for $C_{35}H_{25}NOPS$, 538.1389; found 538.1393.

(*R*)-Diphenyl(6-phenyl-1-(6-phenylisoquinolin-1-yl)naphthalen-2-yl)phosphine oxide (3za)

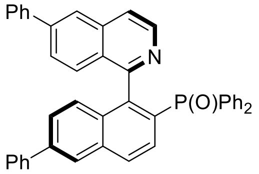

General procedure was used with triflates **1z** (55.5 mg, 0.1 mmol, 1 equiv.) and HP(O)Ph2 **2a** (26.0 mg, 0.13 mmol, 1.3 equiv.) at 100 °C for 24 h to afford **3za** as white wax (48.5 mg, 80% yield, 90% ee). $[\alpha]^{20}_{D}$ = 59.8 (c 1.0, $CHCl_3$). ^{1}H NMR (600 MHz, $CDCl_3$) δ 8.44 (d, *J* = 5.5 Hz, 1H), 8.22-8.14 (m, 3H), 7.93-7.86 (m, 2H), 7.82 (s, 1H), 7.69-7.64 (m, 4H), 7.57-7.53 (m, 1H), 7.53-7.40 (m, 10H), 7.39-7.35 (m, 1H), 7.31 (d, *J* = 8.9 Hz, 1H), 7.18-7.11 (m, 2H), 7.05 (d, *J* = 9.5 Hz, 1H), 7.02-6.98 (m, 1H), 6.89-6.83 (m, 2H). ^{13}C NMR (150 MHz, $CDCl_3$) δ 158.1 (d, *J* = 4.8 Hz), 142.6, 141.9, 141.3 (d, *J* = 9.6 Hz), 140.5, 140.1 (d, *J* = 7.8 Hz), 135.7, 135.1 (d, *J* = 2.0 Hz), 132.9, 132.4

(d, J = 9.6 Hz), 132.2, 131.7 (d, J = 11.3 Hz), 131.5 (d, J = 1.7 Hz), 131.0 (d, J = 10.0 Hz), 130.3, 129.7 (d, J = 100.7 Hz), 129.0, 128.9, 128.1, 128.1, 128.1, 128.0, 127.8, 127.5, 127.4 (d, J = 10.9 Hz), 127.2 (d, J = 11.2 Hz), 126.8 (d, J = 1.8 Hz), 125.7, 124.2, 121.3. ^{31}P NMR (240 MHz, $CDCl_3$) δ 30.1. The enantiomeric excess was determined by Daicel Chiralcel IA (0.46 cm x 25 cm), Hexanes / IPA = 60 / 40, 1.0 mL/min, λ = 254 nm, t (minor) = 9.3 min, t (major) = 10.4 min. HRMS (ESI-ion trap) *m/z*: $[M+H]^+$ calcd for $C_{43}H_{31}NOP$, 608.2138; found 608.2137.

(*R*)-Diphenyl(7-phenyl-1-(7-phenylisoquinolin-1-yl)naphthalen-2-yl)phosphine oxide (3aaa)

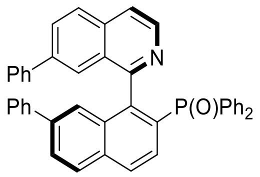

General procedure was used with triflates **1aa** (55.5 mg, 0.1 mmol, 1 equiv.) and $HP(O)Ph_2$ **2a** (26.0 mg, 0.13 mmol, 1.3 equiv.) at 100 °C for 12 h to afford **3aaa** as white wax (52.5 mg, 86% yield, 94% ee). $[\alpha]^{20}_D$ = -13.1 (c 1.0, $CHCl_3$). ^{1}H NMR (600 MHz, $CDCl_3$) δ 8.48 (d, J = 5.5 Hz, 1H), 8.26-8.20 (m, 1H), 8.17-8.12 (m, 1H), 8.05-8.00 (m, 1H), 7.95-7.89 (m, 2H), 7.81-7.76 (m, 2H), 7.73-7.69 (m, 1H), 7.52 (d, J = 5.8 Hz, 1H), 7.50-7.46 (m, 1H), 7.44-7.35 (m, 7H), 7.33-7.30 (m, 1H), 7.29-7.19 (m, 5H), 7.17-7.11 (m, 2H), 7.07 (s, 1H), 6.99-6.94 (m, 1H), 6.86-6.80 (m, 2H). ^{13}C NMR (150 MHz, $CDCl_3$) δ 158.4 (d, J = 5.0 Hz), 141.3, 140.5, 140.1, 140.0, 139.9, 134.5, 133.9 (d, J = 2.5Hz), 132.9 (d, J = 11.7 Hz), 132.7, 132.4 (d, J = 10.7 Hz), 132.0 (d, J = 8.5 Hz), 131.6, 131.4, 131.0 (d, J = 10.7 Hz), 130.4 (d, J = 2.1 Hz), 130.3, 129.8, 129.6, 129.3, 128.8, 128.6, 128.6, 128.4 (d, J = 9.9 Hz), 128.1 (d, J = 12.7 Hz), 128.0, 127.6, 127.3, 127.3, 127.2, 127.1, 125.0, 124.6, 121.0. ^{31}P NMR (240 MHz, $CDCl_3$) δ 30.7. The enantiomeric excess was determined by Daicel Chiralcel IA (0.46 cm x 25 cm), Hexanes / IPA = 60 / 40, 1.0 mL/min, λ = 254 nm, t (minor) = 6.8 min, t (major) = 11.3 min. HRMS (ESI-ion trap) *m/z*: $[M+H]^+$ calcd for $C_{43}H_{31}NOP$, 608.2138; found 608.2137.

(*R*)-Diphenyl(7-phenyl-1-(6-phenylisoquinolin-1-yl)naphthalen-2-yl)phosphine oxide (3aba)

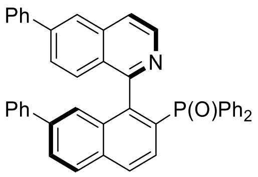

General procedure was used with triflates **1ab** (55.5 mg, 0.1 mmol, 1 equiv.) and $HP(O)Ph_2$ **2a** (26.0 mg, 0.13 mmol, 1.3 equiv.) at 100 °C for 6 h to afford **3aba** as white wax (50.6 mg, 83% yield, 94% ee). $[\alpha]^{20}_{D}$ = -16.9 (c 1.0, $CHCl_3$). ^1H NMR (600 MHz, $CDCl_3$) δ 8.45 (d, *J* = 6.0 Hz, 1H), 8.18-8.11 (m, 2H), 8.05 (d, *J* =8.3 Hz, 1H), 7.92-7.85 (m, 2H), 7.83-7.79 (m, 2H), 7.68-7.64 (m, 2H), 7.53-7.48 (m, 5H), 7.45-7.40 (m, 3H), 7.34-7.31 (m, 1H), 7.31-7.25 (m, 3H), 7.24-7.20 (m, 1H), 7.18-7.11 (m, 3H), 7.05-6.99 (m, 1H), 6.91-6.84 (m, 2H). ^{13}C NMR (150 MHz, $CDCl_3$) δ 157.9 (d, *J* = 5.1 Hz), 142.6, 141.8, 141.5 (d, *J* = 9.3 Hz), 140.5, 140.0, 139.9, 135.7, 133.9 (d, *J* = 2.0 Hz), 132.9 (d, *J* = 11.7 Hz), 132.4 (d, *J* = 10.0 Hz), 132.1, 131.6, 131.4, 131.0 (d, *J* = 10.7 Hz), 130.5, 130.4, 129.8, 128.9, 128.6, 128.5, 128.4, 128.2, 128.1 (d, *J* = 6.0 Hz), 127.9, 127.4, 127.3, 127.3, 127.2 (d, *J* = 12.5 Hz), 126.9, 124.7, 124.2, 121.4. ^{31}P NMR (240 MHz, $CDCl_3$) δ 30.4. The enantiomeric excess was determined by Daicel Chiralcel IC (0.46 cm x 25 cm), Hexanes / IPA = 60 / 40, 1.0 mL/min, λ = 254 nm, t (major) = 21.9 min, t (minor) = 33.8 min. HRMS (ESI-ion trap) *m/z*: $[M+H]^+$ calcd for $C_{43}H_{31}NOP$, 608.2138; found 608.2137.

(*R*)-Diphenyl(6-phenyl-1-(7-phenyl-8,8a-dihydroisoquinolin-1-yl)naphthalen-2-yl)phosphine oxide (3aca)

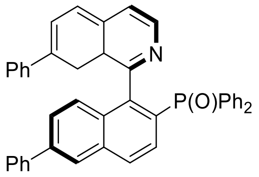

General procedure was used with triflates **1ac** (55.5 mg, 0.1 mmol, 1 equiv.) and $HP(O)Ph_2$ **2a** (26.0 mg, 0.13 mmol, 1.3 equiv.) at 100 °C for 12 h to afford **3aca** as white wax (48.3 mg, 80% yield, 95% ee). $[\alpha]^{20}_{D}$ = 49.7 (c 1.0, $CHCl_3$). ^1H NMR (600

MHz, $CDCl_3$) δ 8.47 (d, J = 5.4 Hz, 1H), 8.30-8.23 (m, 1H), 8.20-8.12 (m, 2H), 7.96-7.89 (m, 2H), 7.81-7.77 (m, 1H), 7.74-7.70 (m, 1H), 7.68-7.62 (m, 2H), 7.53-7.49 (m, 2H), 7.49-7.35 (m, 11H), 7.34-7.30 (m, 1H), 7.16-7.09 (m, 2H), 6.99-6.91 (m, 2H), 6.84-6.77 (m, 2H). ^{13}C NMR (150 MHz, $CDCl_3$) δ 158.5 (d, J = 5.0 Hz), 141.4, 141.0 (d, J = 10.2 Hz), 140.5, 140.1 (d, J = 6.7 Hz), 140.0, 135.1, 134.5, 132.7, 132.5 (d, J = 10.6 Hz), 132.0 (d, J = 24.2 Hz), 131.7 (d, J = 11.1 Hz), 131.6, 131.4, 131.0 (d, J = 10.0 Hz), 130.4 (d, J = 2.7 Hz), 129.8, 129.2, 129.1, 129.0, 128.9, 128.8 (d, J = 2.2 Hz), 128.1 (d, J = 12.3 Hz), 127.8 (d, J = 12.0 Hz), 127.3, 127.3 (d, J = 34.3 Hz), 127.1 (d, J = 2.8 Hz), 126.9, 125.8, 125.1, 120.9. ^{31}P NMR (240 MHz, $CDCl_3$) δ 30.7. The enantiomeric excess was determined by Daicel Chiralcel IC (0.46 cm x 25 cm), Hexanes / IPA = 60 / 40, 1.0 mL/min, λ = 254 nm, t (major) = 21.4 min, t (minor) = 40.9 min. HRMS (ESI-ion trap) *m/z*: $[M+H]^+$ calcd for $C_{43}H_{31}NOP$, 608.2138; found 608.2137.

(*R*)-(6-Methoxy-1-(6-phenylisoquinolin-1-yl)naphthalen-2-yl)diphenylphosphine oxide (3ada)

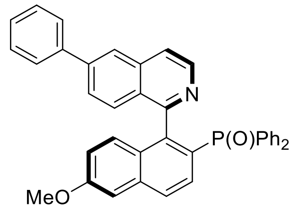

General procedure was used with triflates **1ad** (50.9 mg, 0.1 mmol, 1 equiv.) and $HP(O)Ph_2$ **2a** (26.0 mg, 0.13 mmol, 1.3 equiv.) at 100 °C for 24 h to afford **3ada** as white wax (36.4 mg, 64% yield, 90% ee). $[\alpha]^{20}_D$ = 150.6 (c 1.0, $CHCl_3$). ^{1}H NMR (600 MHz, $CDCl_3$) δ 8.39 (d, J = 5.5 Hz, 1H), 8.13-8.07 (m, 1H), 8.00-7.96 (m, 1H), 7.88-7.82 (m, 2H), 7.78 (s, 1H), 7.66-7.62 (m, 2H), 7.52-7.36 (m, 9H), 7.27-7.21 (m, 2H), 7.14-7.08 (m, 2H), 7.00-6.95 (m, 1H), 6.94-6.90 (m, 1H), 6.88-6.81 (m, 3H), 3.91 (s, 3H). ^{13}C NMR (150 MHz, $CDCl_3$) δ 159.1, 158.2 (d, J = 4.7 Hz), 142.5, 141.8, 141.3 (d, J = 9.1 Hz), 140.1, 136.5 (d, J = 2.1 Hz), 135.7, 133.1, 132.5, 132.4, 132.4, 131.8, 131.4, 131.0 (d, J = 10.3 Hz), 130.2, 129.2 (d, J = 10.1 Hz), 128.9, 128.5, 128.1, 128.0, 128.0, 127.9, 127.5 (d, J = 11.9 Hz), 127.4, 127.1 (d, J = 12.2 Hz), 126.8, 126.7, 124.1,

121.2, 119.9, 105.7, 55.3. ^{31}P NMR (240 MHz, $CDCl_3$) δ 30.2. The enantiomeric excess was determined by Daicel Chiralcel AD (0.46 cm x 25 cm), Hexanes / IPA = 60 / 40, 1.0 mL/min, λ = 254 nm, t (minor) = 11.1 min, t (major) = 25.0 min. HRMS (ESI-ion trap) *m/z*: $[M+H]^+$ calcd for $C_{38}H_{29}NO_2P$, 562.1930; found 562.1924.

(*R*)-(7-Methoxy-1-(7-phenylisoquinolin-1-yl)naphthalen-2-yl)diphenylphosphine oxide (3aea)

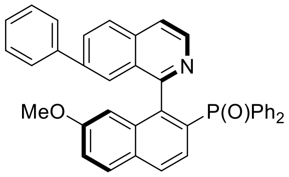

General procedure was used with triflates **1ae** (50.9 mg, 0.1 mmol, 1 equiv.) and $HP(O)Ph_2$ **2a** (26.0 mg, 0.13 mmol, 1.3 equiv.) at 100 ºC for 24 h to afford **3aea** as white wax (49.6 mg, 88% yield, 93% ee). $[\alpha]^{20}_D$ = 141.8 (c 1.0, $CHCl_3$). ^{1}H NMR (600 MHz, $CDCl_3$) δ 8.44 (d, *J* = 5.0 Hz, 1H), 8.11-8.05 (m, 1H), 8.05-8.00 (m, 1H), 7.91-7.82 (m, 3H), 7.80-7.75 (m, 1H), 7.72-7.67 (m, 1H), 7.48 (d, *J* = 5.7 Hz, 1H), 7.46-7.35 (m, 8H), 7.33-7.29 (m, 1H), 7.21-7.17 (m, 1H), 7.14-7.07 (m, 2H), 6.96-7.90 (m, 1H), 6.84-6.76 (m, 2H), 6.13 (s, 1H), 3.35 (s, 3H). ^{13}C NMR (150 MHz, $CDCl_3$) δ 158.8 (d, *J* = 4.8 Hz), 158.2, 141.5, 140.1, 139.8, 139.7 (d, *J* = 9.6 Hz), 134.5, 133.8 (d, *J* = 11.9 Hz), 133.0, 132.4 (d, *J* = 10.8 Hz), 132.3, 131.7, 131.5, 131.0 (d, *J* = 9.9 Hz), 130.4, 130.3, 130.3, 129.7, 129.6, 129.6, 129.0, 128.8, 128.5 (d, *J* = 12.2 Hz), 128.0 (d, *J* = 12.2 Hz), 127.6, 127.3, 127.1, 127.0, 127.0, 126.3 (d, *J* = 8.2 Hz), 125.0, 120.8, 120.4, 105.4, 54.8. ^{31}P NMR (240 MHz, $CDCl_3$) δ 30.4. The enantiomeric excess was determined by Daicel Chiralcel IA (0.46 cm x 25 cm), Hexanes / IPA = 60 / 40, 1.0 mL/min, λ = 254 nm, t (minor) = 6.6 min, t (major) = 7.5 min. HRMS (ESI-ion trap) *m/z*: $[M+H]^+$ calcd for $C_{38}H_{29}NO_2P$, 562.1930; found 562.1924.

(*R*)-(7-Methoxy-1-(6-phenylisoquinolin-1-yl)naphthalen-2-yl)diphenylphosphine oxide (3afa)

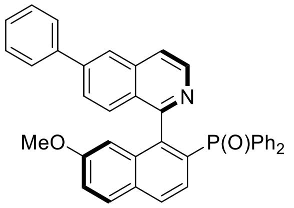

General procedure was used with triflates **1af** (50.9 mg, 0.1 mmol, 1 equiv.) and $HP(O)Ph_2$ **2a** (26.0 mg, 0.13 mmol, 1.3 equiv.) at 100 °C for 24 h to afford **3afa** as white wax (49.3 mg, 88% yield, 91% ee). $[\alpha]^{20}_{D}$ = 83.0 (c 1.0, $CHCl_3$). ^1H NMR (600 MHz, $CDCl_3$) δ 8.41 (d, *J* = 5.5 Hz, 1H), 8.04-7.94 (m, 2H), 7.88-7.79 (m, 4H), 7.68-7.63 (m, 2H), 7.52-7.45 (m, 5H), 7.44-7.37 (m, 3H), 7.31-7.27 (m, 1H), 7.24-7.19 (m, 1H), 7.17-7.09 (m, 2H), 7.04-6.97 (m, 1H), 6.90-6.81 (m, 2H), 6.20 (s, 1H), 3.39 (s, 3H). ^{13}C NMR (150 MHz, $CDCl_3$) δ 158.3 (d, *J* = 5.1 Hz), 158.3, 142.5, 142.0, 140.0, 139.9 (d, *J* = 10.0 Hz), 135.7, 133.8 (d, *J* = 11.9 Hz), 133.1, 132.4 (d, *J* = 9.8 Hz), 131.8, 131.4 (d, *J* = 2.0 Hz), 131.1 (d, *J* = 10.3 Hz), 130.5, 130.4, 130.3, 129.8, 129.6, 129.0, 128.3 (d, *J* = 11.6 Hz), 128.1 (d, *J* = 4.8 Hz), 128.0, 128.0, 128.0, 127.4, 127.2 (d, *J* = 12.5 Hz), 126.8, 126.4 (d, *J* = 9.9 Hz), 124.1, 121.2, 120.5, 105.3, 54.9. ^{31}P NMR (240 MHz, $CDCl_3$) δ 30.2. The enantiomeric excess was determined by Daicel Chiralcel AD (0.46 cm x 25 cm), Hexanes / IPA = 60 / 40, 1.0 mL/min, λ = 254 nm, t (minor) = 6.1 min, t (major) = 9.9 min. HRMS (ESI-ion trap) *m/z*: $[M+H]^+$ calcd for $C_{38}H_{29}NO_2P$, 562.1930; found 562.1924.

(*R*)-(6-Methoxy-1-(7-phenylisoquinolin-1-yl)naphthalen-2-yl)diphenylphosphine oxide (3aga)

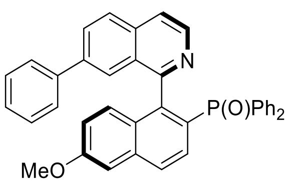

General procedure was used with triflates **1ag** (50.9 mg, 0.1 mmol, 1 equiv.) and $HP(O)Ph_2$ **2a** (26.0 mg, 0.13 mmol, 1.3 equiv.) at 100 °C for 24 h to afford **3aga** as white wax (47.6 mg, 85% yield, 94% ee). $[\alpha]^{20}_{D}$ = 170.4 (c 1.0, $CHCl_3$). ^1H NMR (600 MHz, $CDCl_3$) δ 8.42 (d, *J* = 5.6 Hz, 1H), 8.19 (t, *J* = 9.1 Hz, 1H), 8.02-7.97 (m, 1H),

7.93-7.86 (m, 2H), 7.77-7.73 (m, 1H), 7.69-7.65 (m, 1H), 7.48-7.42 (m, 2H), 7.41-7.33 (m, 6H), 7.33-7.28 (m, 2H), 7.21 (d, J = 1.9 Hz, 1H), 7.12-7.05 (m, 2H), 6.93-6.86 (m, 2H), 6.82-6.73 (m, 3H), 3.88 (s, 3H). ^{13}C NMR (150 MHz, $CDCl_3$) δ 159.0, 158.7 (d, J = 4.9 Hz), 141.4, 141.0 (d, J = 9.4 Hz), 140.1, 139.8, 136.5, 134.4, 133.1, 132.4 (d, J = 10.4 Hz), 131.8, 131.4, 130.9 (d, J = 10.2 Hz), 130.2, 129.6, 129.2, 129.1, 128.8, 128.4, 128.0, 128.0, 128.0, 127.9, 127.7, 127.6, 127.3, 127.1, 127.0, 126.6, 125.1, 120.8, 120.0, 105.8, 55.3. ^{31}P NMR (240 MHz, $CDCl_3$) δ 30.4. The enantiomeric excess was determined by Daicel Chiralcel IC (0.46 cm x 25 cm), Hexanes / IPA = 50 / 50, 1.0 mL/min, λ = 254 nm, t (major) = 20.6 min, t (minor) = 29.8 min. HRMS (ESI-ion trap) *m/z*: $[M+H]^+$ calcd for $C_{38}H_{29}NO_2P$, 562.1930; found 562.1924.

(*R*)-Diphenyl(1-(quinazolin-4-yl)naphthalen-2-yl)phosphine oxide (3aha)

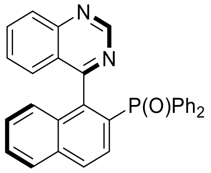

General procedure was used with triflates **1ah** (40.2 mg, 0.1 mmol, 1 equiv.) and $HP(O)Ph_2$ **2a** (26.0 mg, 0.13 mmol, 1.3 equiv.) at 100 °C for 12 h to afford **1aha** as white wax (37.0 mg, 81% yield, 73% ee). $[\alpha]^{20}_D$ = 141.0 (c 1.0, $CHCl_3$). ^{1}H NMR (600 MHz, $CDCl_3$) δ 9.17 (s, 1H), 8.11-8.06 (m, 1H), 8.03-7.98 (m, 1H), 7.96-7.90 (m, 2H), 7.79-7.74 (m, 3H), 7.55 (t, J = 7.4 Hz, 1H), 7.51-7.46 (m, 1H), 7.42-7.38 (m, 2H), 7.32-7.27 (m, 2H), 7.23-7.18 (m, 3H), 7.14-7.10 (m, 1H), 6.99-6.94 (m, 2H), 6.89 (d, J = 8.4 Hz, 1H). ^{13}C NMR (150 MHz, $CDCl_3$) δ 167.5 (d, J = 4.6 Hz), 154.0, 149.5, 139.0 (d, J = 8.3 Hz), 134.5 (d, J = 2.5 Hz), 133.7, 132.5, 132.3 (d, J = 10.0 Hz), 132.2, 131.8, 131.8 (d, J = 2.3 Hz), 131.7 (d, J = 10.8 Hz), 131.5, 131.3, 131.3, 131.1 (d, J = 2.1 Hz), 130.5, 130.4, 129.9, 129.3, 129.2, 128.6, 128.5, 128.4, 128.3, 128.2, 128.2, 128.2, 127.6, 127.5, 127.1, 126.3, 125.6. ^{31}P NMR (240 MHz, $CDCl_3$): 29.4. The enantiomeric excess was determined by Daicel Chiralcel AD (0.46 cm x 25 cm), Hexanes / IPA = 60 / 40, 1.0 mL/min, λ = 254 nm, t (minor) = 8.2 min, t (major) = 10.9 min. HRMS (ESI-ion trap) *m/z*: $[M+H]^+$ calcd for $C_{30}H_{22}N_2OP$, 457.1464; found 457.1458.

(*R*)- (1-(Isoquinolin-1-yl)naphthalen-2-yl)di-p-tolylphosphine oxide (3ab)

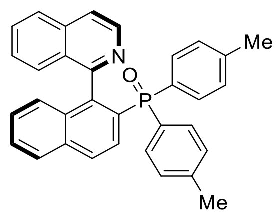

General procedure was used with triflates **1a** (40.3 mg, 0.1 mmol, 1 equiv.) and di-p-tolylphosphine oxide **2b** (29.9 mg, 0.13 mmol, 1.3 equiv.) at 100 ºC for 6 h to afford **3ab** as white wax (45.0 mg, 93% yield, 93% ee). $[\alpha]^{20}_{D}$ = 224.3 (c 1.0, $CHCl_3$). ^{1}H NMR (600 MHz, $CDCl_3$) δ 8.39 (d, *J* = 5.4 Hz, 1H), 8.18-8.13 (m, 1H), 8.09-8.05 (m, 1H), 7.93 (d, *J* = 8.1 Hz, 1H), 7.74-7.68 (m, 2H), 7.63-7.60 (m, 1H), 7.54-7.49 (m, 2H), 7.45 (d, *J* = 5.8 Hz, 1H), 7.25-7.14 (m, 5H), 6.96-6.90 (m, 2H), 6.82 (d, *J* = 8.4 Hz, 1H), 6.59-6.55 (m, 2H), 2.36 (s, 3H), 2.09 (s, 3H). ^{13}C NMR (150 MHz, $CDCl_3$) δ 158.4 (d, *J* = 5.1 Hz), 141.9 (d, *J* = 3.1 Hz), 141.4, 141.1 (d, *J* = 8.8 Hz), 140.6 (d, *J* = 2.4 Hz), 135.3, 134.7, 132.6 (d, *J* = 11.1 Hz), 132.4 (d, *J* = 10.3 Hz), 131.0 (d, *J* = 10.0 Hz), 130.5, 130.5, 129.8, 129.6, 129.3 (d, *J* = 12.8 Hz), 129.1, 128.9 (d, *J* = 13.0 Hz), 128.7 (d, *J* = 11.8 Hz), 128.5 (d, *J* = 9.3 Hz), 128.1, 128.0, 127.9, 127.8, 127.6, 127.0, 127.0, 126.9, 126.3, 120.8, 21.5, 21.3.^{31}P NMR (240 MHz, $CDCl_3$) δ 30.8. The enantiomeric excess was determined by Daicel Chiralcel AD (0.46 cm x 25 cm), Hexanes / IPA = 70 / 30, 1.0 mL/min, λ = 230 nm, t (minor) = 12.8 min, t (major) = 14.3 min. HRMS (ESI-ion trap) *m/z*: $[M+H]^+$ calcd for $C_{33}H_{27}NOP$, 484.1825; found 484.1821.

(*R*)-(1-(Isoquinolin-1-yl)naphthalen-2-yl)di-m-tolylphosphine oxide (3ac)

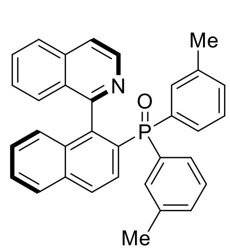

General procedure was used with triflates **1a** (40.3 mg, 0.1 mmol, 1 equiv.) and di-*m*-tolylphosphine oxide **2c** (29.9 mg, 0.13 mmol, 1.3 equiv.) at 100 ºC for 6 h to afford **3ac** as white wax (34.5 mg, 71% yield, 93% ee). $[\alpha]^{20}_{D}$ = 212.2 (c 1.0, $CHCl_3$). ^{1}H NMR (600 MHz, $CDCl_3$) δ 8.41 (d, *J* = 5.3 Hz, 1H), 8.16-8.11 (m, 1H), 8.10-8.06 (m, 1H),

7.94 (d, J = 8.3 Hz, 1H), 7.68-7.61 (m, 3H), 7.54-7.49 (m, 2H), 7.46 (d, J = 5.7 Hz, 1H), 7.29-7.21 (m, 4H), 7.19-7.15 (m, 1H), 7.00 (d, J = 13.7 Hz, 1H), 6.84 (d, J = 8.7 Hz, 1H), 6.78-6.68 (m, 3H), 2.30 (s, 3H), 1.98 (s, 3H). ^{13}C NMR (150 MHz, $CDCl_3$) δ 158.2 (d, J = 5.0 Hz), 141.0, 140.9 (d, J = 9.3 Hz), 137.9 (d, J = 11.9 Hz), 136.9 (d, J = 11.9 Hz), 135.4, 134.6 (d, J = 1.8 Hz), 132.6, 132.5, 132.5, 132.4 (d, J = 2.6 Hz), 131.8, 131.5, 131.4, 131.1, 130.4, 130.0, 129.7, 129.7, 128.9, 128.8, 128.7, 128.7, 128.6, 128.4 (d, J = 9.6 Hz), 128.0, 127.9, 127.9, 127.5, 127.1, 127.0, 126.9 (d, J = 11.6 Hz), 126.3, 121.2, 21.3, 21.1.^{31}P NMR (240 MHz, $CDCl_3$) δ 30.7. The enantiomeric excess was determined by Daicel Chiralcel AD (0.46 cm x 25 cm), Hexanes / IPA = 70 / 30, 1.0 mL/min, λ = 254 nm, t (minor) = 5.8 min, t (major) = 8.3 min. HRMS (ESI-ion trap) *m/z*: $[M+H]^+$ calcd for $C_{33}H_{27}NOP$, 484.1825; found 484.1821.

(*R*)-bis(4-(*tert*-Butyl)phenyl)(1-(isoquinolin-1-yl)naphthalen-2-yl)phosphine oxide (3ae)

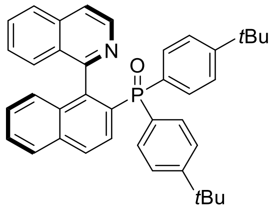

General procedure was used with triflates **1a** (40.3 mg, 0.1 mmol, 1 equiv.) and bis(4-(tert-butyl)phenyl)phosphine oxide **2e** (40.8 mg, 0.13 mmol, 1.3 equiv.) at 100 °C for 12 h to afford **3ae** as white wax (47.9 mg, 84% yield, 94% ee). $[\alpha]^{20}_D$ = 216.7 (c 1.0, $CHCl_3$). ^{1}H NMR (600 MHz, $CDCl_3$) δ 8.39 (d, J = 5.7 Hz, 1H), 8.16-8.09 (m, 1H), 8.09-8.04 (m, 1H), 7.93 (d, J = 8.4 Hz, 1H), 7.77-7.71 (m, 2H), 7.64-7.61 (m, 1H), 7.54-7.48 (m, 2H), 7.43-7.36 (m, 3H), 7.27-7.19 (m, 3H), 7.07-7.00 (m, 2H), 6.88-6.84 (m, 2H), 6.82 (d, J = 8.6 Hz, 1H), 1.29 (s, 9H), 1.15 (s, 9H). ^{13}C NMR (150 MHz, $CDCl_3$) δ 158.2 (d, J = 5.0 Hz), 154.7 (d, J = 3.0 Hz), 153.4 (d, J = 2.4 Hz), 141.3, 140.8 (d, J = 8.7 Hz), 135.2, 134.6 (d, J = 2.0 Hz), 132.5 (d, J = 11.2 Hz), 132.1 (d, J = 11.2 Hz), 131.1 (d, J = 10.8 Hz), 130.5, 130.3, 130.0, 129.8, 129.7, 129.1, 129.0, 128.7 (d, J = 11.5 Hz), 128.5 (d, J = 10.0 Hz), 128.3, 128.0, 127.8, 127.6, 127.0 (d, J = 6.6 Hz), 126.8, 126.5, 125.1 (d, J = 12.8 Hz), 124.2 (d, J = 12.3 Hz), 120.9, 34.8, 34.5, 31.0, 30.9.^{31}P

NMR (240 MHz, $CDCl_3$) δ 30.1. The enantiomeric excess was determined by Daicel Chiralcel AD (0.46 cm x 25 cm), Hexanes / IPA = 70 / 30, 1.0 mL/min, λ = 214 nm, t (minor) = 6.9 min, t (major) = 9.3 min. HRMS (ESI-ion trap) *m/z*: $[M+H]^+$ calcd for $C_{39}H_{39}NOP$, 568.2764; found 568.2747.

(*R*)-(1-(Isoquinolin-1-yl)naphthalen-2-yl)bis(4-methoxyphenyl)phosphine oxide (3af)

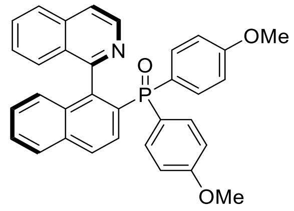

General procedure was used with triflates **1a** (40.3 mg, 0.1 mmol, 1 equiv.) and bis(4-methoxyphenyl)phosphine oxide **2f** (34.0 mg, 0.13 mmol, 1.3 equiv.) at 100 °C for 6 h to afford **3af** as white wax (44.3 mg, 86% yield, 92% ee). $[\alpha]^{20}_{D}$ = 166.2 (c 1.0, $CHCl_3$). ^1H NMR (600 MHz, $CDCl_3$) δ 8.41 (d, *J* = 5.5 Hz, 1H), 8.21-8.12 (m, 1H), 8.10-8.02 (m, 1H), 7.92 (d, *J* = 8.4 Hz, 1H), 7.78-7.68 (m, 2H), 7.68-7.62 (m, 1H), 7.55-7.46 (m, 3H), 7.27-7.19 (m, 2H), 7.18-7.11 (m, 1H), 6.98-6.91 (m, 2H), 6.91-6.83 (m, 2H), 6.79 (d, *J* = 8.6 Hz, 1H), 6.24 (d, *J* = 5.7 Hz, 2H), 3.79 (s, 3H), 3.61 (s, 3H). ^{13}C NMR (150 MHz, $CDCl_3$) δ 162.2, 161.0, 158.4 (d, *J* = 3.6 Hz), 141.1, 140.5 (d, *J* = 9.0 Hz), 135.6, 134.7 (d, *J* = 1.7 Hz), 134.3 (d, *J* = 11.6 Hz), 132.9 (d, *J* = 11.9 Hz), 132.6 (d, *J* = 11.1 Hz), 130.2, 129.1, 128.9 (d, *J* = 11.2 Hz), 128.5 (d, *J* = 9.9 Hz), 128.1, 127.9, 127.7, 127.2 (d, *J* = 6.9 Hz), 126.8, 126.4, 121.3, 113.7 (d, *J* = 14.1 Hz), 112.9 (d, *J* = 13.1 Hz), 55.3, 55.0. ^{31}P NMR (240 MHz, $CDCl_3$) δ 30.3. The enantiomeric excess was determined by Daicel Chiralcel OD (0.46 cm x 25 cm), Hexanes / IPA = 70 / 30, 1.0 mL/min, λ = 230 nm, t (minor) = 8.0 min, t (major) = 10.6 min. HRMS (ESI-ion trap) *m/z*: $[M+H]^+$ calcd for $C_{33}H_{27}NO_3P$, 516.1723; found 516.1714.

(*R*)-di([1,1'-Biphenyl]-4-yl)(1-(isoquinolin-1-yl)naphthalen-2-yl)phosphine oxide (3ag)

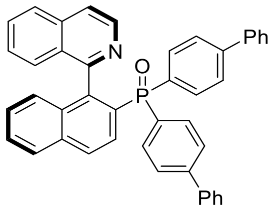

General procedure was used with triflates **1a** (40.3 mg, 0.1 mmol, 1 equiv.) and di([1,1'-biphenyl]-4-yl)phosphine oxide **2g** (46.0 mg, 0.13 mmol, 1.3 equiv.) at 100 °C for 12 h to afford **3ag** as white wax (52.3 mg, 86% yield, 92% ee). $[\alpha]^{20}_{D}$ = 288.1 (c 1.0, $CHCl_3$). ^{1}H NMR (600 MHz, $CDCl_3$) δ 8.44 (d, *J* = 5.5 Hz, 1H), 8.32-8.26 (m, 1H), 8.17-8.12 (m, 1H), 8.02-7.95 (m, 3H), 7.67-7.63 (m, 2H), 7.62-7.58 (m, 2H), 7.54 (t, *J* = 6.2 Hz, 1H), 7.51-7.41(m, 7H), 7.41-7.33 (m, 4H), 7.29-7.23 (m, 3H), 7.19-7.11 (m, 2H), 7.04-6.97 (m, 2H), 6.88 (d, *J* = 8.4 Hz, 1H). ^{13}C NMR (150 MHz, $CDCl_3$) δ 158.2 (d, *J* = 5.1 Hz), 144.3 (d, *J* = 2.5 Hz), 142.8 (d, *J* = 1.8 Hz), 141.3, 140.9 (d, *J* = 9.9 Hz), 139.9 (d, *J* = 19.8 Hz), 135.2, 134.7 (d, *J* = 2.1 Hz), 132.9 (d, *J* = 10.6 Hz), 132.5 (d, *J* = 12.0 Hz), 131.4 (d, *J* = 10.9 Hz), 131.3, 130.5 (d, *J* = 14.5 Hz), 130.1, 130.0, 129.8, 129.5, 129.1, 128.9 (d, *J* = 11.5 Hz), 128.8, 128.7, 128.4 (d, *J* = 9.8 Hz), 128.1, 128.0, 127.8, 127.4, 127.2, 127.0, 126.8, 126.8, 126.5, 125.8 (d, *J* = 12.4 Hz), 121.0. ^{31}P NMR (240 MHz, $CDCl_3$) δ 30.5. The enantiomeric excess was determined by Daicel Chiralcel AD (0.46 cm x 25 cm), Hexanes / IPA = 70 / 30, 1.0 mL/min, λ = 214 nm, t (minor) = 18.1 min, t (major) = 28.3 min. HRMS (ESI-ion trap) *m/z*: $[M+H]^+$ calcd for $C_{43}H_{31}NOP$, 608.2138; found 608.2137.

(*R*)-bis(4-Fluorophenyl)(1-(isoquinolin-1-yl)naphthalen-2-yl)phosphine oxide (3ah)

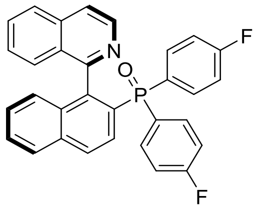

General procedure was used with triflates **1a** (40.3 mg, 0.1 mmol, 1 equiv.) and bis(4-

fluorophenyl)phosphane **3ah** (30.9 mg, 0.13 mmol, 1.3 equiv.) at 100 °C for 12 h to afford **3ah** as white wax (40.0 mg, 81% yield, 93% ee). $[\alpha]^{20}_{D}$ = 205.6 (c 1.0, $CHCl_3$). ^1H NMR (600 MHz, $CDCl_3$) δ 8.39 (d, *J* = 5.9 Hz, 1H), 8.23-8.18 (m, 1H), 8.14-8.10 (m, 1H), 7.98-7.94 (m, 1H), 7.94-7.88 (m, 2H), 7.69-7.65 (m, 1H), 7.55 (q, *J* = 7.0 Hz, 2H), 7.49 (d, *J* = 5.5 Hz, 1H), 7.28-7.22 (m, 2H), 7.16-7.08 (m, 3H), 7.00-6.92 (m, 2H), 6.83 (d, *J* = 8.0 Hz, 1H), 6.40 (t, *J* = 8.2 Hz, 2H). ^{13}C NMR (150 MHz, $CDCl_3$) δ 165.2 (dd, *J* = 190.4, 3.1 Hz), 163.5 (dd, *J* = 189.2, 2.4 Hz), 158.2 (d, *J* = 5.5 Hz), 141.4, 141.0 (d, *J* = 9.7 Hz), 135.3, 135.1 (dd, *J* = 11.5, 8.4 Hz), 134.8 (d, *J* = 2.2 Hz), 133.2 (dd, *J* = 12.3, 9.1 Hz), 132.4 (d, *J* = 11.5 Hz), 130.2, 129.6, 129.1, 129.0, 128.9, 128.9, 128.4, 128.2, 128.1, 128.1, 127.7 (dd, *J* = 21.4, 2.9 Hz), 127.3, 127.3, 127.1, 126.8, 126.5, 121.3, 115.6 (dd, *J* = 20.8, 13.4 Hz), 114.5 (dd, *J* = 21.9, 13.6 Hz). ^{31}P NMR (240 MHz, $CDCl_3$) δ 29.4. ^{19}F NMR (564 MHz, $CDCl_3$) δ -106.8, -108.0. The enantiomeric excess was determined by Daicel Chiralcel AD (0.46 cm x 25 cm), Hexanes / IPA = 70 / 30, 1.0 mL/min, λ = 254 nm, t (minor) = 7.7 min, t (major) = 12.3 min. HRMS (ESI-ion trap) *m/z*: $[M+H]^+$ calcd for $C_{31}H_{21}F_2NOP$, 492.1323; found 492.1321.

(*R*)-bis(4-Chlorophenyl)(1-(isoquinolin-1-yl)naphthalen-2-yl)phosphine oxide (3ai)

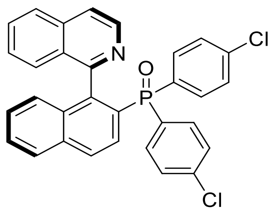

General procedure was used with triflates **1a** (40.3 mg, 0.1 mmol, 1 equiv.) and bis(4-fluorophenyl)phosphane **2i** (35.1 mg, 0.13 mmol, 1.3 equiv.) at 100 °C for 6 h to afford **3ai** as white wax (44.8 mg, 85% yield, 87% ee). $[\alpha]^{20}_{D}$ = 250.8 (c 1.0, $CHCl_3$). ^1H NMR (600 MHz, $CDCl_3$) δ 8.39 (d, *J* = 5.9 Hz, 1H), 8.23-8.17 (m, 1H), 8.15-8.10 (m, 1H), 7.98-7.94 (m, 1H), 7.88-7.81 (m, 2H), 7.70-7.67 (m, 1H), 7.60-7.53 (m, 2H), 7.51 (d, *J* = 6.0 Hz, 1H), 7.41-7.37 (m, 2H), 7.30-7.23 (m, 2H), 7.15-7.10 (m, 1H), 6.89-6.82 (m, 3H), 6.69-6.64 (m, 2H). ^{13}C NMR (150 MHz, $CDCl_3$) δ 158.1 (d, *J* = 5.5 Hz), 141.3, 141.0 (d, *J* = 9.8 Hz), 138.6 (d, *J* = 3.7 Hz), 137.1 (d, *J* = 3.2 Hz), 135.3, 134.9, 134.0 (d, *J* = 10.7 Hz), 132.4 (d, *J* = 12.0 Hz), 131.9 (d, *J* = 11.2 Hz), 130.7, 130.3, 130.0 (d,

J = 6.6 Hz), 129.4 (d, J = 4.0 Hz), 129.2 (d, J = 11.7 Hz), 129.0, 128.6 (d, J = 13.1 Hz), 128.2 (d, J = 13.4 Hz), 128.0 (d, J = 9.6 Hz), 127.5, 127.4, 127.4, 127.4, 127.2, 126.8 (d, J = 11.5 Hz), 121.3. ^{31}P NMR (240 MHz, $CDCl_3$) δ 29.6. The enantiomeric excess was determined by Daicel Chiralcel AD (0.46 cm x 25 cm), Hexanes / IPA = 70 / 30, 1.0 mL/min, λ = 230 nm, t (minor) = 10.4 min, t (major) = 15.2 min. HRMS (ESI-ion trap) *m/z*: $[M+H]^+$ calcd for $C_{31}H_{21}Cl_2NOP$, 524.0732; found 524.0729.

(*R*)-(1-(Isoquinolin-1-yl)naphthalen-2-yl)bis(4-(trifluoromethyl)phenyl) phosphine oxide (3aj)

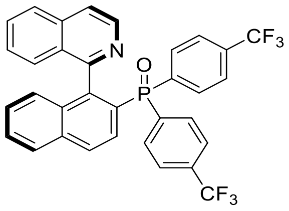

General procedure was used with triflates **1a** (40.3 mg, 0.1 mmol, 1 equiv.) and bis(4-(trifluoromethyl)phenyl)phosphine oxide **2j** (43.9 mg, 0.13 mmol, 1.3 equiv.) at 100 °C for 24 h to afford **3aj** as white wax (46.7 mg, 79% yield, 86% ee). $[\alpha]^{20}_D$ = 188.4 (c 1.0, $CHCl_3$). ^{1}H NMR (600 MHz, $CDCl_3$) δ 8.41-8.34 (m, 1H), 8.29-8.19 (m, 1H), 8.19-8.05 (m, 3H), 8.01-7.93 (m, 1H), 7.73-7.64 (m, 2H), 7.62-7.50 (m, 3H), 7.46-7.40 (m, 1H), 7.32-7.23 (m, 2H), 7.17-7.10 (m, 1H), 7.10-7.03 (m, 2H), 7.00-6.92 (m, 2H), 6.90-6.81 (m, 1H). ^{13}C NMR (150 MHz, $CDCl_3$) δ 157.9 (d, J = 5.3 Hz), 141.3, 141.1 (d, J = 10.9 Hz), 135.6 (dd, J = 140.7, 102.6 Hz) , 135.2, 135.0, 133.7 (q, J = 31.1 Hz), 133.1 (d, J = 10.5 Hz), 132.4 (d, J = 12.3 Hz), 132.0 (q, J = 32.9 Hz), 131.0 (d, J = 10.8 Hz), 130.3, 129.4 (d, J = 11.1 Hz), 129.0, 128.7, 128.9, 128.7, 128.4, 128.2, 128.0, 127.9 (d, J = 9.4 Hz), 127.5 (d, J = 8.7 Hz), 127.0, 126.9, 126.8, 125.1 (dd, J = 12.4, 3.2 Hz), 124.3 (d, J = 53.0 Hz), 123.7 (dd, J = 11.8, 2.7 Hz), 122.4 (d, J = 55.0 Hz), 121.6. ^{31}P NMR (240 MHz, $CDCl_3$) δ 28.7. ^{19}F NMR (564 MHz, $CDCl_3$) δ -63.2, -63.4. The enantiomeric excess was determined by Daicel Chiralcel AD (0.46 cm x 25 cm), Hexanes / IPA = 70 / 30, 1.0 mL/min, λ = 230 nm, t (minor) = 6.3 min, t (major) = 7.8 min. HRMS (ESI-ion trap) *m/z*: $[M+H]^+$ calcd for $C_{33}H_{21}F_6NOP$, 592.1259; found 592.1246.

(*R*)-Bis(3,5-Dimethylphenyl)(1-(isoquinolin-1-yl)naphthalen-2-yl)phosphine oxide (3ak)

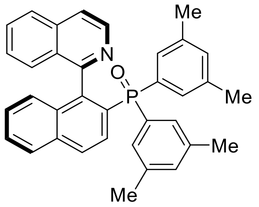

General procedure was used with triflates **1a** (40.3 mg, 0.1 mmol, 1 equiv.) and bis(3,5-dimethylphenyl)phosphine oxide **2k** (33.5 mg, 0.13 mmol, 1.3 equiv.) at 100 ºC for 6 h to afford **3ak** as white wax (43.7 mg, 85% yield, 94% ee). $[\alpha]^{20}_{D}$ = 256.6 (c 1.0, $CHCl_3$). ^{1}H NMR (600 MHz, $CDCl_3$) 8.43 (d, *J* = 5.8 Hz, 1H), 8.21-8.16 (m, 1H), 8.11-8.05 (m, 1H), 7.94 (d, *J* = 8.6 Hz, 1H), 7.66-7.61 (m, 1H), 7.54-7.43 (m, 5H), 7.27-7.20 (m, 2H), 7.19-7.14 (m, 1H), 7.06 (s, 1H), 6.82 (d, *J* = 8.7 Hz, 1H), 6.65 (d, *J* = 12.7 Hz, 2H), 6.50 (s, 1H), 2.27 (s, 6H), 1.94 (s, 6H). ^{13}C NMR (150 MHz, $CDCl_3$) δ 158.4 (d, *J* = 5.0 Hz), 141.1, 141.0 (d, *J* = 8.1 Hz), 138.3 (d, *J* = 12.4 Hz), 137.6 (d, *J* = 13.4 Hz), 136.5 (d, *J* = 12.8 Hz), 135.2, 134.6 (d, *J* = 2.0 Hz), 133.4, 133.3 (d, *J* = 2.2 Hz), 132.6 (d, *J* = 3.0 Hz), 132.5, 132.5, 132.5, 131.8 (d, *J* = 5.6 Hz), 131.1, 131.0, 130.3, 130.0, 130.0, 129.7, 129.1 (d, *J* = 10.4 Hz), 128.8-128.6 (m), 128.0, 127.9, 127.7, 127.5, 126.9 (d, *J* = 2.1 Hz), 126.7, 126.1, 121.0, 21.3, 21.0.^{31}P NMR (240 MHz, $CDCl_3$) δ 30.8. The enantiomeric excess was determined by Daicel Chiralcel AD (0.46 cm x 25 cm), Hexanes / IPA = 70 / 30, 1.0 mL/min, λ = 230 nm, t (minor) = 4.3 min, t (major) = 6.4 min. HRMS (ESI-ion trap) *m/z*: $[M+H]^+$ calcd for $C_{35}H_{31}NOP$, 512.2138; found 512.2135.

(*R*)-(1-(Isoquinolin-1-yl)naphthalen-2-yl)bis(4-methoxy-3,5-dimethylphenyl) phosphine oxide (3al)

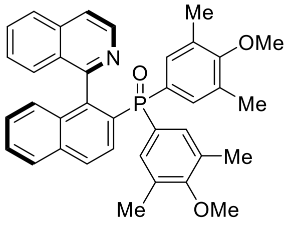

General procedure was used with triflates **1a** (40.3 mg, 0.1 mmol, 1 equiv.) and bis(4-

methoxy-3,5-dimethylphenyl)phosphine oxide **2l** (41.3 mg, 0.13 mmol, 1.3 equiv.) at 100 °C for 6 h to afford **3al** as white wax (39.2 mg, 70% yield, 92% ee). $[\alpha]^{20}_{D}$ = 240.0 (c 1.0, $CHCl_3$). ^1H NMR (600 MHz, $CDCl_3$) 8.44 (d, *J* = 5.7 Hz, 1H), 8.22-8.16 (m, 1H), 8.11-8.07 (m, 1H), 7.94 (d, *J* = 8.5 Hz, 1H), 7.67-7.63 (m, 1H), 7.54-7.45 (m, 5H), 7.28-7.21 (m, 2H), 7.20-7.16 (m, 1H), 6.79 (d, *J* = 8.8 Hz, 1H), 6.66 (d, *J* = 12.2 Hz, 2H), 3.71 (s, 3H), 3.52 (s, 3H), 2.23 (s, 6H), 1.90 (s, 6H). ^{13}C NMR (150 MHz, $CDCl_3$) δ 159.8 (d, *J* = 3.4 Hz), 158.9 (d, *J* = 3.4 Hz), 158.4 (d, *J* = 5.0 Hz), 141.1, 141.0 (d, *J* = 8.1 Hz), 135.2, 134.6 (d, *J* = 2.0 Hz), 133.1 (d, *J* = 11.6 Hz), 132.5 (d, *J* = 11.8 Hz), 132.3 (d, *J* = 11.2 Hz), 131.0, 130.9, 130.3, 130.0, 129.8, 129.8, 128.9, 128.9, 128.8, 128.6 (d, *J* = 9.4 Hz), 128.1, 127.8, 127.5, 127.4, 127.0, 126.9, 126.9, 126.2, 126.1, 120.9, 59.5, 59.2, 16.1, 15.9. ^{31}P NMR (240 MHz, $CDCl_3$) δ 30.2. The enantiomeric excess was determined by Daicel Chiralcel OD (0.46 cm x 25 cm), Hexanes / IPA = 70 / 30, 1.0 mL/min, λ = 230 nm, t (minor) = 4.5 min, t (major) = 5.7 min. HRMS (ESI-ion trap) *m/z*: $[M+H]^+$ calcd for $C_{37}H_{35}NO_3P$, 572.2349; found 572.2340.

(*R*)-(1-(Isoquinolin-1-yl)naphthalen-2-yl)di(naphthalen-2-yl)phosphine oxide (3am)

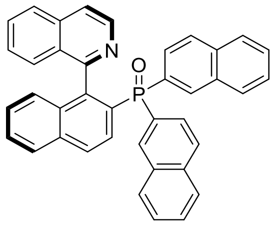

General procedure was used with triflates **1a** (40.3 mg, 0.1 mmol, 1 equiv.) and di(naphthalen-2-yl)phosphine oxide **2m** (39.3 mg, 0.13 mmol, 1.3 equiv.) at 100 °C for 6 h to afford **3am** as white wax (54.3 mg, 98% yield, 91% ee). $[\alpha]^{20}_{D}$ = 319.0 (c 1.0, $CHCl_3$). ^1H NMR (600 MHz, $CDCl_3$) 8.47 (d, *J* = 14.2 Hz, 1H), 8.28-8.19 (m, 2H), 8.13-8.07 (m, 1H), 7.98-7.91 (m, 2H), 7.89-7.83 (m, 3H), 7.66 (d, *J* = 14.8 Hz, 1H), 7.60-7.49 (m, 4H), 7.48-7.41 (m, 2H), 7.38-7.33 (m, 1H), 7.28-7.17 (m, 5H), 7.15-7.07 (m, 3H), 6.88 (d, *J* = 8.2 Hz, 1H). ^{13}C NMR (150 MHz, $CDCl_3$) δ 157.8 (d, *J* = 5.5 Hz), 141.5 (d, *J* = 9.2 Hz), 141.3, 135.0, 134.7 (dd, *J* = 28.0, 1.9 Hz), 133.4 (d, *J* = 9.7 Hz), 133.8 (d, *J* = 2.0 Hz), 133.0 (d, *J* = 9.6 Hz), 132.6 (d, *J* = 11.2 Hz), 132.3 (d, *J* = 13.5

Hz), 131.6 (d, J = 13.6 Hz), 130.0 (d, J = 11.5 Hz), 129.7, 129.4 (d, J = 5.1 Hz), 129.3, 129.2, 128.9-128.7 (m), 128.5 (d, J = 9.9 Hz), 128.1, 128.0, 128.0, 127.9 (d, J = 11.8 Hz), 127.7, 127.5, 127.4, 127.3, 127.1, 127.1, 127.0, 126.9, 126.9, 126.6, 126.4 (d, J = 11.4 Hz), 126.0, 126.0, 121.2. ^{31}P NMR (240 MHz, $CDCl_3$) δ 30.5. The enantiomeric excess was determined by Daicel Chiralcel AD (0.46 cm x 25 cm), Hexanes / IPA = 60 / 40, 1.0 mL/min, λ = 254 nm, t (minor) = 13.5 min, t (major) = 27.8 min. HRMS (ESI-ion trap) *m/z*: $[M+H]^+$ calcd for $C_{39}H_{27}NOP$, 556.1825; found 556.1810.

(*R*)-Dibenzyl(1-(isoquinolin-1-yl)naphthalen-2-yl)phosphine oxide (3an)

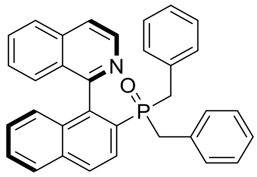

General procedure was used with triflates **1a** (40.3 mg, 0.1 mmol, 1 equiv.) and dibenzylphosphine oxide **2n** (29.9 mg, 0.13 mmol, 1.3 equiv.) at 100 °C for 6 h to afford **3an** as white wax (44.0 mg, 91% yield, 20% ee). $[\alpha]^{20}_{D}$ = -24.6 (c 1.0, $CHCl_3$). ^{1}H NMR (600 MHz, $CDCl_3$) 8.84 (d, J = 5.4 Hz, 1H), 8.00-7.88 (m, 4H), 7.87-7.79 (m, 1H), 7.70-7.62 (m, 1H), 7.57-7.50 (m, 1H), 7.36-7.20 (m, 4H), 7.16-6.96 (m, 10H), 6.88 (s, 2H), 3.60-3.50 (m, 1H), 3.20-3.11 (m, 1H), 3.10-2.97 (m, 1H), 2.71-2.56 (m, 1H). ^{13}C NMR (150 MHz, $CDCl_3$) δ 159.3 (d, J = 4.1 Hz), 140.0 (d, J = 7.2 Hz), 135.7, 134.5, 132.3 (d, J = 11.5 Hz), 131.6, 131.5, 131.4, 130.6, 130.0 (dd, J = 28.6, 5.3 Hz), 129.8 (d, J = 4.1 Hz), 129.6, 129.2, 128.7 (d, J = 20.0 Hz), 128.4, 128.3, 128.1, 128.0, 127.8 (d, J = 32.9 Hz), 127.1 (d, J = 35.5 Hz), 126.9, 126.9, 126.6, 126.3 (d, J = 3.1 Hz), 121.5, 37.4, 37.0, 37.0, 36.6.^{31}P NMR (240 MHz, $CDCl_3$) δ 36.8. The enantiomeric excess was determined by Daicel Chiralcel AD (0.46 cm x 25 cm), Hexanes / IPA = 70 / 30, 1.0 mL/min, λ = 230 nm, t (major) = 12.7 min, t (minor) = 25.4 min. HRMS (ESI-ion trap) *m/z*: $[M+H]^+$ calcd for $C_{33}H_{27}NOP$, 484.1825; found 484.1821.

(*R*)-Ethyl(1-(isoquinolin-1-yl)naphthalen-2-yl)(phenyl)phosphine oxide (3ao)

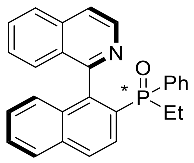

General procedure was used with triflates **1a** (40.3 mg, 0.1 mmol, 1 equiv.) and dibenzylphosphine oxide **2n** (30.8 mg, 0.2 mmol, 2 equiv.) at 100 °C for 12 h to afford **3ao** as white wax (35.6 mg, 87% yield, dr 1.6:1, minor 83% ee, major 82% ee). (EA to CH_3OH/EA = 1 : 20). ^{1}H NMR (600 MHz, $CDCl_3$) 8.82-8.85 (m, 1H), 8.20-8.16 (m, 0.27H), 8.17-8.11 (m, 0.93H), 7.97-7.94 (m, 0.3H), 7.93-7.89 (m, 0.92H), 7.77 (d, J = 5.8 Hz, 0.48H), 7.73-7.68 (m, 0.28H), 7.67-7.60 (m, 1H), 7.59-7.53 (m, 1H), 7.53-7.46 (m, 1.17H), 7.46-7.42 (m, 0.32H), 7.42-7.36 (m, 0.52H), 7.36-7.22 (m, 2.50H), 7.20-7.15 (m, 0.30H), 7.06-7.02 (m, 0.3H), 7.02-6.96 (m, 1H), 6.93-6.75 (m, 0.83H), 6.74-6.68 (m, 0.54H), 2.56-2.50 (m, 0.34H), 2.41-2.31 (m, 0.34H), 2.05-1.97 (m, 0.30H), 1.94-1.83 (m, 0.30H), 1.58-1.51 (m, 0.43H), 1.50-1.44 (m, 0.43H), 1.25-1.19 (m, 0.62H), 1.14-1.08 (m, 0.62H), 0.91-0.80 (m, 1.76H). ^{13}C NMR (150 MHz, $CDCl_3$) δ ^{13}C NMR (150 MHz, $CDCl_3$) δ 158.7, 158.6, 158.3, 158.3, 141.9, 141.3, 140.8, 140.8, 140.0, 139.9, 135.6, 135.3, 134.6, 134.6, 134.4, 134.4, 133.5, 132.8, 132.5, 132.5, 132.5, 132.4, 132.0, 131.4, 131.0, 130.9, 130.8, 130.7, 130.5, 130.4, 130.4, 130.3, 130.1, 129.9, 129.8, 129.8, 129.3, 129.2, 129.1, 128.6, 128.6, 128.5, 128.5, 128.4, 128.4, 128.3, 128.2, 128.2, 128.1, 128.1, 128.0, 127.9, 127.7, 127.5, 127.5, 127.5, 127.4, 127.3, 127.3, 127.2, 127.2, 126.9, 126.9, 126.8, 126.8, 126.7, 126.5, 126.2, 121.4, 120.8, 114.0, 22.6, 22.5, 22.3, 22.3, 22.2, 21.9, 21.8, 5.5, 5.4, 5.4, 5.4, 5.3.^{31}P NMR (240 MHz, $CDCl_3$) δ 35.9, 34.7. The enantiomeric excess was determined by Daicel Chiralcel AD (0.46 cm x 25 cm), Hexanes / IPA = 70 / 30, 1.0 mL/min, λ = 230 nm, t1 (manor) = 6.1 min, t1 (major) = 6.9 min, t2 (major) = 9.9 min, t2 (minor) = 11.1 min. HRMS (ESI-ion trap) *m/z*: $[M+H]^+$ calcd for $C_{27}H_{23}NOP$, 408.1512; found 408.1510.

(*R*)-Ethyl (1-(isoquinolin-1-yl)naphthalen-2-yl)(phenyl)phosphinate (3ap)

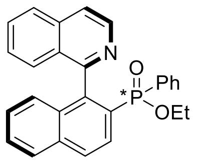

General procedure was used with triflates **1a** (40.3 mg, 0.1 mmol, 1 equiv.) and dibenzylphosphine oxide **2n** (34.0 mg, 0.2 mmol, 2 equiv.) at 100 °C for 12 h to afford **3ap** as white wax (20.0 mg, 47% yield, dr 1.3:1, minor 20% ee, major 2% ee). (EA to CH_3OH/EA = 1 : 20). ^{1}H NMR (600 MHz, $CDCl_3$) 8.61 (d, *J* = 5.7 Hz, 0.59H), 8.52 (d, *J* = 5.7 Hz, 0.41H), 8.42-8.37 (m, 0.43H), 8.20-8.15 (m, 0.58H), 8.14-8.10 (m, 0.44), 8.09-8.05 (m, 0.57H), 7.78-7.88 (m, 1.64H), 7.84-7.78 (m, 0.87H), 7.76 (d, *J* = 5.8 Hz, 0.58H), 7.72 (d, *J* = 5.8 Hz, 0.46H), 7.66-7.62 (m, 0.58H), 7.62-7.56 (m, 1.36H), 7.56-7.48 (m, 2.00H), 7.40-7.35 (m, 0.70H), 7.31-7.18 (m, 5.22H), 7.15-7.11 (m, 0.56H), 7.09-7.00 (m, 2.00H), 6.97-6.93 (m, 0.46H), 4.41-3.97 (m, 1.12H), 3.66-3.57 (m, 0.89H), 1.30-1.26 (m, 1.51H), 0.67 (t, *J* = 7.3 Hz, 1.70H). ^{13}C NMR (150 MHz, $CDCl_3$) δ 159.3, 159.2, 158.2, 158.2, 142.1, 142.0, 141.8, 141.8, 141.3, 141.2, 135.6, 135.5, 134.9, 134.9, 134.8, 134.7, 132.6, 132.6, 132.5, 132.5, 132.3, 132.2, 132.2, 132.1, 131.6, 131.6, 131.5, 131.5, 131.4, 131.3, 131.2, 131.2, 131.1, 131.0, 131.0, 130.0, 129.9, 129.2, 129.2, 128.9, 128.8, 128.7, 128.6, 128.6, 128.5, 128.4, 128.4, 128.3, 128.2, 128.1, 128.0, 127.9, 127.9, 127.8, 127.7, 127.5, 127.3, 127.1, 127.0, 126.9, 126.9, 126.9, 126.9, 126.5, 126.4, 120.7, 120.5, 120.4, 114.0, 60.7, 60.7, 60.5, 60.5, 60.4, 60.4, 16.4, 16.3, 16.2, 16.2, 16.1, 15.5, 15.4, 15.4, 14.1. ^{31}P NMR (240 MHz, $CDCl_3$) δ 31.3, 28.8. The enantiomeric excess was determined by Daicel Chiralcel AD (0.46 cm x 25 cm), Hexanes / IPA = 70 / 30, 1.0 mL/min, λ = 230 nm, t1 (minor) = 5.6 min, t1 (major) = 7.6 min, t2 (major) = 6.7 min, t2 (minor) = 9.9 min. HRMS (ESI-ion trap) *m/z*: $[M+H]^+$ calcd for $C_{24}H_{22}NO_2P$, 424.1461; found 424.1460.

(*R*)-1-(2-(Diphenylphosphaneyl)naphthalen-1-yl)isoquinoline (3aa')

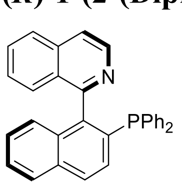

white wax (70.0 mg, 80% yield, 90% ee). $[\alpha]^{20}_{D}$ = 169.8 (c 0.33, $CHCl_3$).^{1}H NMR (600 MHz, $CDCl_3$) δ 8.66 (d, J = 5.2 Hz, 1H), 7.96-7.89 (m, 3H), 7.78 (d, J = 5.2 Hz, 1H), 7.67-7.62 (m, 1H), 7.51 (t, J = 7.4 Hz, 1H), 7.48-7.44 (m, 1H), 7.36-7.17 (m, 13H), 7.13 (d, J = 8.2 Hz, 1H). ^{13}C NMR (150 MHz, $CDCl_3$) δ 160.4 (d, J = 6.7 Hz), 144.3 (d, J = 34.2 Hz), 142.2, 137.4 (d, J = 12.3 Hz), 137.3 (d, J = 11.2 Hz), 135.9, 134.8 (d, J = 13.9 Hz), 133.7, 133.6, 133.5, 133.2, 133.1, 132.6 (d, J = 7.9 Hz), 130.0 (d, J = 6.1 Hz), 128.9 (d, J = 2.7 Hz), 128.6, 128.4, 128.2, 128.2, 128.0, 127.9, 127.4, 127.0, 126.8, 126.6, 120.3. ^{31}P NMR (240 MHz, $CDCl_3$) δ -14.1. The enantiomeric excess was determined by Daicel Chiralcel ID (0.46 cm x 25 cm), Hexanes / IPA = 70 / 30, 1.0 mL/min, λ = 254 nm, t (minor) = 8.0 min, t (major) = 9.9 min. HRMS (ESI-ion trap) *m/z*: $[M+H]^+$ calcd for $C_{31}H_{24}NP$, 440.1563; found 440.1566.

(*R*)-1-(2-(Diphenylphosphaneyl)naphthalen-1-yl)-4-phenylisoquinoline (3ha')

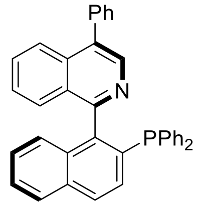

white wax (80.7 mg, 80% yield, 91% ee). $[\alpha]^{20}_{D}$ = 131.4 (c 0.33, $CHCl_3$).^{1}H NMR (600 MHz, $CDCl_3$) δ 8.61 (s, 1H), 8.03 (d, J = 8.6 Hz, 1H), 7.97-7.91 (m, 2H), 7.71-7.67 (m, 2H), 7.62-7.58 (m, 3H), 7.55-7.50 (m, 2H), 7.48 (dd, J = 8.4, 3.1 Hz, 1H), 7.42-7.40 (m, 1H), 7.32-7.29 (m, 6H), 7.28-7.20 (m, 7H). ^{13}C NMR (150 MHz, $CDCl_3$) δ 159.7 (d, J = 6.7 Hz), 144.1 (d, J = 32.0 Hz), 142.0, 137.4, 137.3, 137.3, 137.2, 135.0 (d, J = 13.6 Hz), 134.2, 133.8, 133.7, 133.6, 133.3, 133.2, 132.8, 132.7 (d, J = 7.4 Hz), 130.4, 130.3 (d, J = 18.4 Hz), 128.7, 128.6, 128.5, 128.4, 128.3, 128.2, 128.2, 128.1, 127.9 (d, J = 10.8 Hz), 127.7, 126.8, 126.7, 126.6, 125.0. ^{31}P NMR (240 MHz, $CDCl_3$) δ -13.6. The enantiomeric excess was determined by Daicel Chiralcel ID (0.46 cm x 25 cm), Hexanes / IPA = 70 / 30, 1.0 mL/min, λ = 254 nm, t (minor) = 5.9 min, t (major) = 8.1 min. HRMS (ESI-ion trap) *m/z*: $[M+H]^+$ calcd for $C_{37}H_{27}NP$, 518.1876; found 516.1871.

(*R*)-1-(2-(Diphenylphosphaneyl)-6-phenylnaphthalen-1-yl)-6-phenylisoquinoline (3za')

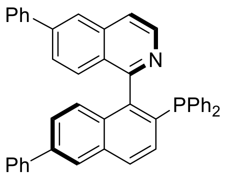

white wax (98.8 mg, 83% yield, 81% ee). $[\alpha]^{20}_{D}$ = -7.5 (c 0.33, $CHCl_3$).^{1}H NMR (600 MHz, $CDCl_3$) δ 8.72 (d, *J* = 5.5 Hz, 1H), 8.14 (d, *J* = 16.2 Hz, 2H), 8.02 (d, *J* = 8.5 Hz, 1H), 7.87-7.84 (m, 1H), 7.75-7.70 (m, 4H), 7.63-7.60 (m, 1H), 7.56-7.45 (m, 8H), 7.43-7.39 (m, 2H), 7.35-7.32 (m, 5H), 7.31-7.27 (m, 1H), 7.27-7.23 (m, 4H). ^{13}C NMR (150 MHz, $CDCl_3$) δ 160.2 (d, *J* = 6.6 Hz), 144.0 (d, *J* = 33.1 Hz), 142.7, 142.6, 140.6, 140.2, 139.4, 137.4-137.2 (m), 136.2, 134.9 (d, *J* = 12.6 Hz), 133.9, 133.7, 133.2, 133.1, 131.8 (d, *J* = 8.4 Hz), 130.4, 128.9, 128.8, 128.4, 128.3, 128.2, 128.1 (d, *J* =5.5 Hz), 127.9, 127.5, 127.3, 127.1, 126.8, 126.3, 125.7, 124.5, 120.5. ^{31}P NMR (240 MHz, $CDCl_3$) δ -14.0. The enantiomeric excess was determined by Daicel Chiralcel ID (0.46 cm x 25 cm), Hexanes / IPA = 70 / 30, 1.0 mL/min, λ = 254 nm, t (minor) = 11.2 min, t (major) = 15.8 min. HRMS (ESI-ion trap) *m/z*: $[M+H]^+$ calcd for $C_{43}H_{31}NP$, 592.2189; found 592.2190.

(*R*)-1-(2-(Diphenylphosphaneyl)-7-phenylnaphthalen-1-yl)-7-phenylisoquinoline (3aaa')

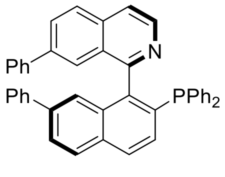

white wax (101.8 mg, 86% yield, 86% ee). $[\alpha]^{20}_{D}$ = -28.5 (c 0.33, $CHCl_3$).^{1}H NMR (600 MHz, $CDCl_3$) δ 8.72 (d, *J* = 5.7 Hz, 1H), 8.01-7.95 (m, 3H), 7.90-7.87 (m, 1H), 7.81 (d, *J* = 5.4 Hz, 1H), 7.77-7.73 (m, 1H), 7.54-7.50 (m, 2H), 7.40-7.37 (m, 2H), 7.34-7.12 (m, 20H). ^{13}C NMR (150 MHz, $CDCl_3$) δ 160.7 (d, *J* = 6.7 Hz), 144.5 (d, *J* = 34.8 Hz), 142.2, 141.0, 140.2, 140.1, 139.4, 137.8 (d, *J* = 11.7 Hz), 137.0 (d, *J* = 12.8 Hz), 135.2 (d, *J* = 12.0 Hz), 135.1, 133.8, 133.6, 133.0, 132.9, 131.8, 130.3, 130.0, 128.6, 128.5, 128.5, 128.3, 128.2, 128.2, 128.2, 128.0, 127.5, 127.4, 127.4 (d, *J* = 2.0 Hz), 127.1,

126.9, 125.2, 124.6, 120.2. ^{31}P NMR (240 MHz, $CDCl_3$) δ -14.6. The enantiomeric excess was determined by Daicel Chiralcel IC (0.46 cm x 25 cm), Hexanes / IPA = 70 / 30, 1.0 mL/min, λ = 254 nm, t (major) = 5.0 min, t (minor) = 11.1 min. HRMS (ESI-ion trap) *m/z*: $[M+H]^+$ calcd for $C_{43}H_{31}NP$, 592.2189; found 592.2190.

Dimethyl (*S,E*)-2-(1,3-diphenylallyl)malonate (6)

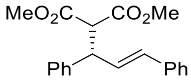

Colorless oil (60% yield, 66% ee). ^{1}H NMR (600 MHz, $CDCl_3$) δ 7.44-7.17 (m, 10H), 6.53 (d, *J* = 15.7 Hz, 1H), 6.38 (dd, *J* = 15.7, 8.6 Hz, 1H), 4.32 (dd, *J* = 10.9, 8.6 Hz, 1H), 4.01 (d, *J* = 10.9 Hz, 1H), 3.74 (s, 3H), 3.56 (s, 3H). The enantiomeric excess was determined by Daicel Chiralcel AD (0.46 cm x 25 cm), Hexanes / IPA = 85 / 15, 1.0 mL/min, λ = 254 nm, t (minor) = 9.5 min, t (major) = 12.8 min. NMR is consistent with the reported data[1].

(*R*)-2-(phenylethynyl)chroman-4-one (9)

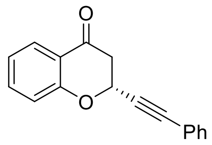

Colorless oil, (51% yield, 65% ee). ^{1}H NMR (500 MHz, Chloroform-*d*) δ 7.93 (dd, *J* = 8.2, 1.8 Hz, 1H), 7.51 (ddd, *J* = 8.3, 7.2, 1.8 Hz, 1H), 7.43–7.41 (m, 2H), 7.35-7.28 (m, 3H), 7.08–7.05 (m, 2H), 5.50 (dd, *J*= 7.7, 5.3 Hz, 1H), 3.09–3.07 (m, 2H). The enantiomeric excess was determined by Daicel Chiralcel AD (0.46 cm x 25 cm), Hexanes / IPA = 95 / 5, 1.0 mL/min, λ = 254 nm, t (minor) = 10.0 min, t (major) = 10.6 min. NMR is consistent with the reported data[2].

(*R*)-1,1'-Binaphthalen]-2-yldiphenylphosphine oxide (12)

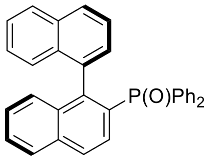

General procedure was used with triflates **10** (40.2 mg, 0.1 mmol, 1 equiv.) and $HP(O)Ph_2$ **2a** (26.0 mg, 0.13 mmol, 1.3 equiv.) at 100 ºC for 24 h to afford **12** as white wax (36.6 mg, 80% yield, 5% ee). ^1H NMR (600 MHz, $CDCl_3$) δ 8.03-7.98 (m, 1H), 7.97-7.92 (m, 1H), 7.90-7.82 (m, 1H), 7.69-7.59 (m, 2H), 7.58-7.51 (m, 3H), 7.51-7.46 (m, 1H), 7.41-7.17 (m, 8H), 7.17-7.12 (m, 1H), 7.12-7.03 (m, 2H), 7.01-6.88 (m, 3H); ^{13}C NMR (150 MHz, $CDCl_3$) δ 144.2 (d, J = 9.1 Hz), 134.6, 134.6, 134.5, 133.3 (d, J = 11.0 Hz), 132.7, 132.6 (d, J = 15.1 Hz), 132.1, 131.7 (d, J = 8.8 Hz), 131.4, 130.9, 130.7 (d, J = 9.6 Hz), 130.4, 130.2, 130.1, 129.5, 128.8, 128.7, 128.7, 127.9, 127.8 (d, J = 5.0 Hz), 127.8, 127.8, 127.7, 127.4 (d, J = 12.1 Hz), 126.7 (d, J = 16.0 Hz), 125.6, 125.3, 124.6; ^{31}P NMR (240 MHz, $CDCl_3$) δ 28.5 ppm; The enantiomeric excess was determined by Daicel Chiralcel OD (0.46 cm x 25 cm), Hexanes / IPA = 80 / 20, 1.0 mL/min, λ = 230 nm, t (major) = 6.0 min, t (minor) = 8.2 min. HRMS (ESI-ion trap) *m/z*: $[M+H]^+$ calcd for $C_{32}H_{24}OP$, 455.1559; found 455.1558.

(*R*)-(1-(3-Methylpyridin-2-yl)naphthalen-2-yl)diphenylphosphine oxide (13)

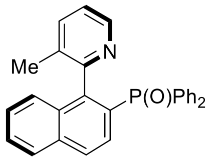

General procedure was used with triflates **11** (36.7 mg, 0.1 mmol, 1 equiv.) and $HP(O)Ph_2$ **2a** (26.0 mg, 0.13 mmol, 1.3 equiv.) at 100 ºC for 24 h to afford **13** as white wax (40.0 mg, 95% yield, 0% ee). ^1H NMR (600 MHz, $CDCl_3$) δ 8.19 (s, 1H), 7.98-7.89 (m, 2H), 7.88-7.81 (m, 2H), 7.81-7.74 (m, 1H), 7.60-7.50 (m, 2H), 7.50-7.31 (m, 7H), 7.30-7.22 (m, 2H), 7.13-7.02 (m, 2H), 1.91 (s, 3H). ^{13}C NMR (150 MHz, $CDCl_3$) δ 173.2, 155.6 (d, J = 4.2 Hz), 145.7, 143.3 (d, J = 8.0 Hz), 137.1, 134.8, 134.3, 133.2, 132.5, 132.3 (d, J = 9.5 Hz), 131.8 (d, J = 11.6 Hz), 131.6, 131.4 (d, J = 9.8 Hz), 131.0, 128.4-127.8 (m), 127.5, 127.2, 126.0, 123.0, 19.1. ^{31}P NMR (240 MHz, $CDCl_3$): 29.6. The enantiomeric excess was determined by Daicel Chiralcel AD (0.46 cm x 25 cm), Hexanes / IPA = 60 / 40, 1.0 mL/min, λ = 230 nm, t (minor) = 5.1 min, t (major) = 6.7 min. HRMS (ESI-ion trap) *m/z*: $[M+H]^+$ calcd for $C_{28}H_{23}NOP$, 420.1512; found 420.1503.

1-(Naphthalen-1-yl)isoquinoline (14)

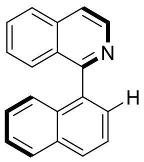

^{1}H NMR (600 MHz, $CDCl_3$) δ 8.73 (d, J = 5.9 Hz, 1H), 8.29 (d, J = 8.1 Hz, 1H), 8.00-7.94 (m, 2H), 7.79 (d, J = 5.5 Hz, 1H), 7.72 (t, J = 7.0 Hz, 1H), 7.67-7.63 (m, 3H), 7.63-7.60 (m, 1H), 7.51 (t, J = 7.7 Hz, 1H), 7.46-7.40 (m, 2H), 7.38-7.30 (m, 1H). ^{13}C NMR (150 MHz, $CDCl_3$) δ 160.4, 142.3, 136.9, 136.4, 133.6, 132.2, 130.2, 128.8, 128.3, 127.8, 127.6, 127.2, 126.8, 126.3, 125.9, 125.2, 120.3.

7. Refference

[1] J.-H. Wu, S. Fang, X. Zheng, J. He, Y. Ma, Z. Su, T. Wang, *Angew. Chem. Int. Ed.* **2023**, *62*, e202309515.

[2] Meng, L.; Ngai, K.-Y. Chang, X.; Lin, Z.; Wang, J. *Org. Lett.* **2020**, *22*, 1155-1159

8. NMR Spectrum

(*R*)-(1-(Isoquinolin-1-yl)naphthalen-2-yl)diphenylphosphine oxide (3aa)

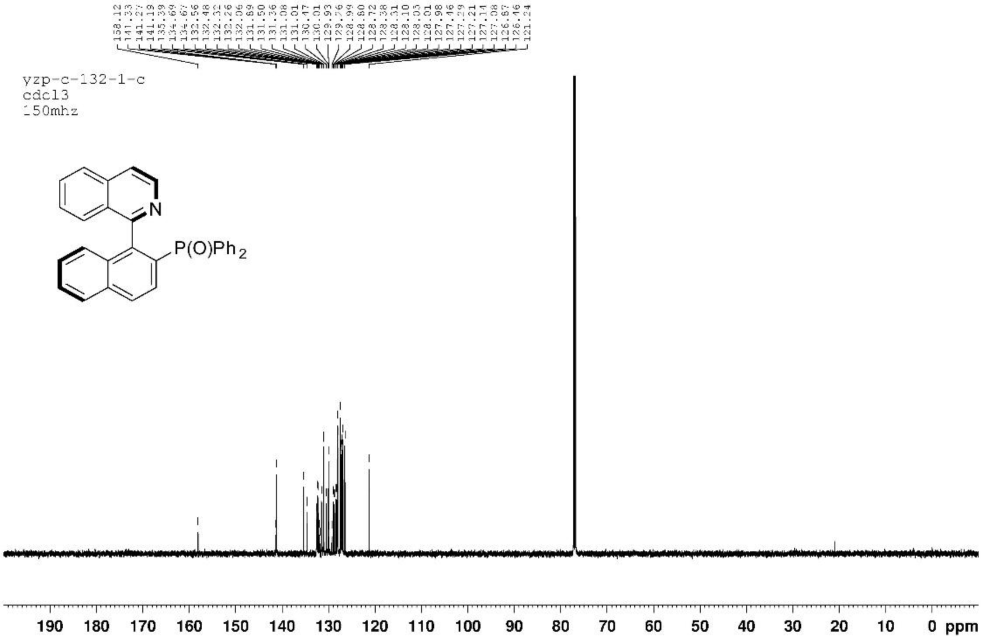

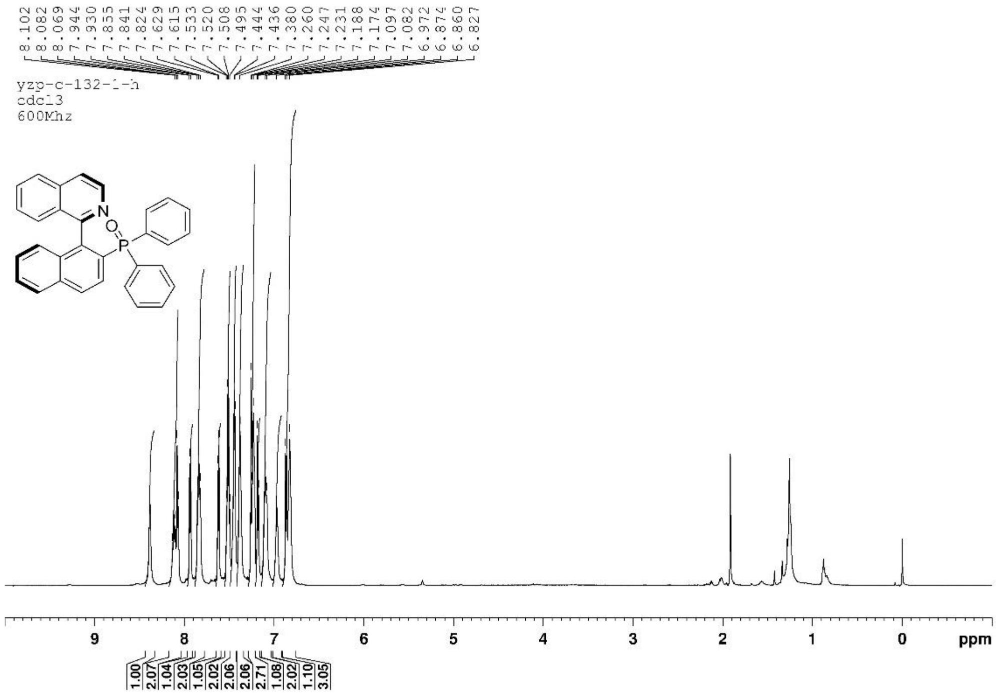

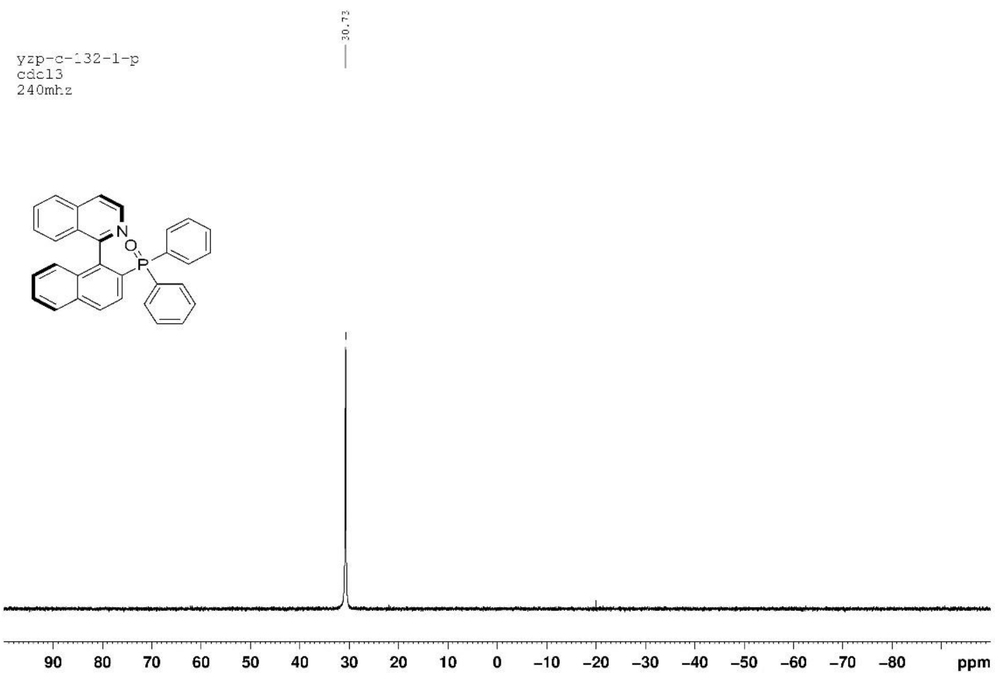

(*R*)-(1-(Isoquinolin-1-yl)-6-methylnaphthalen-2-yl)diphenylphosphine oxide (3ba)

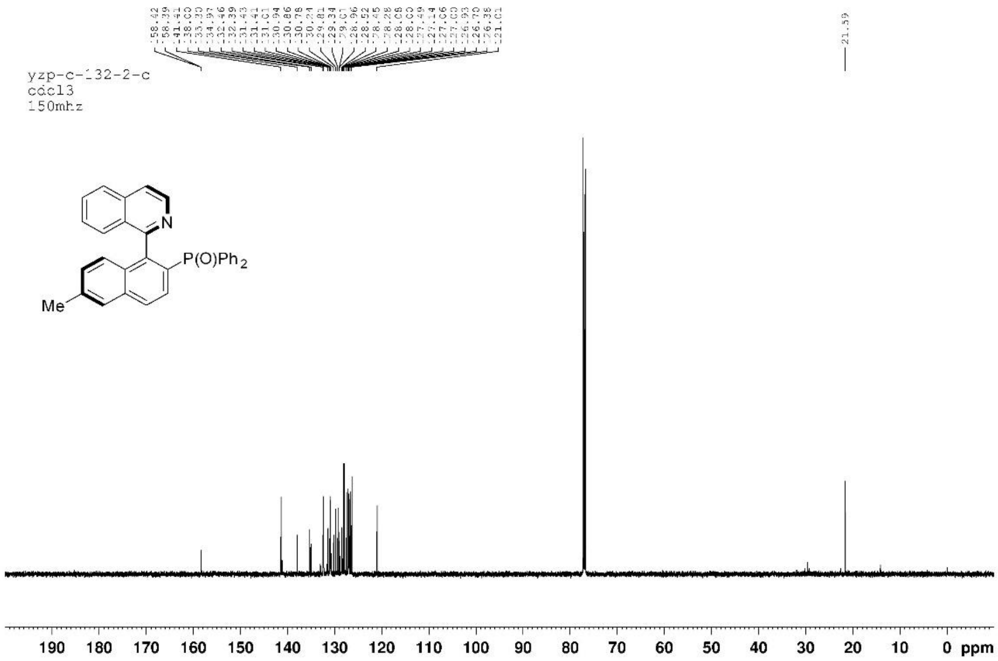

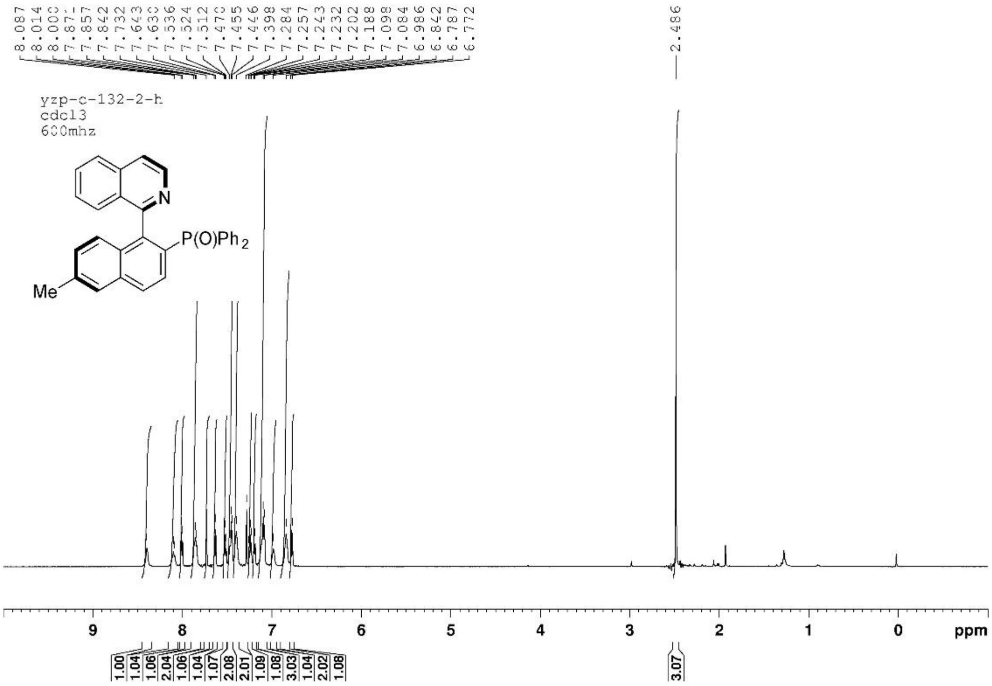

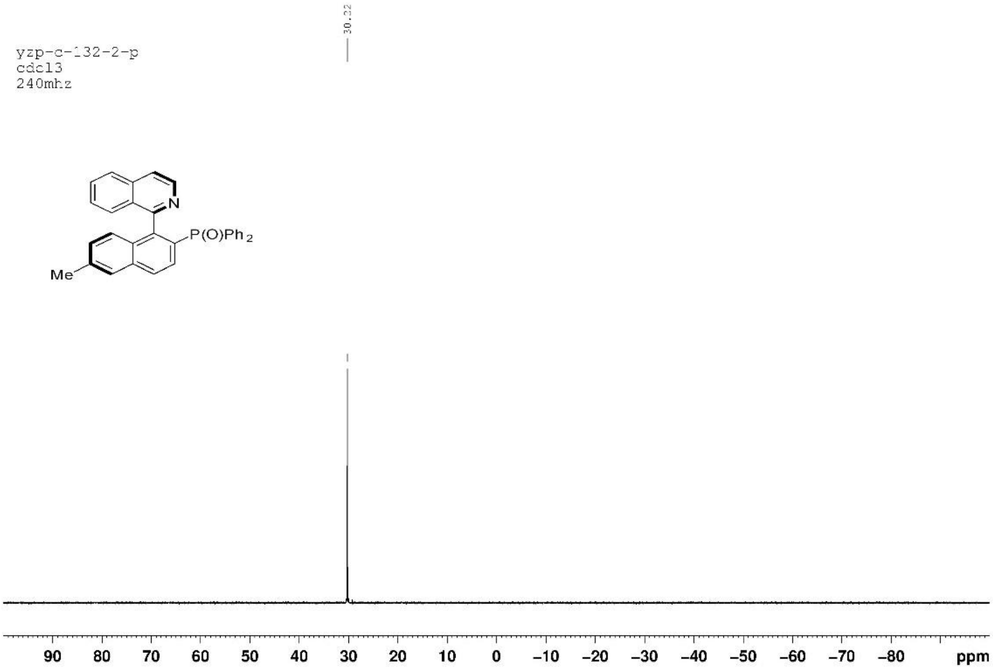

(*R*)- (1-(Isoquinolin-1-yl)-7-methylnaphthalen-2-yl)diphenylphosphine oxide (3ca)

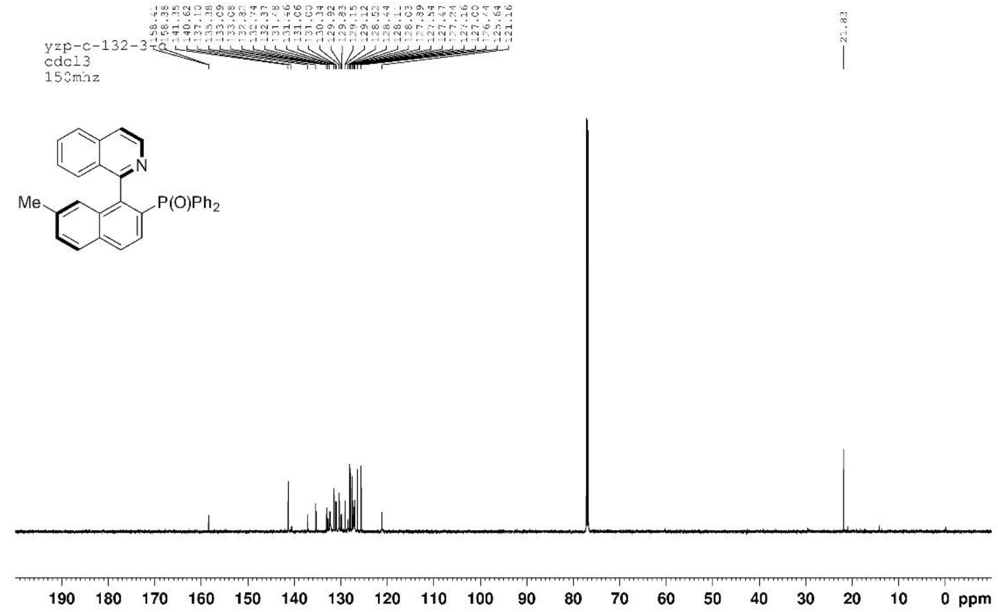

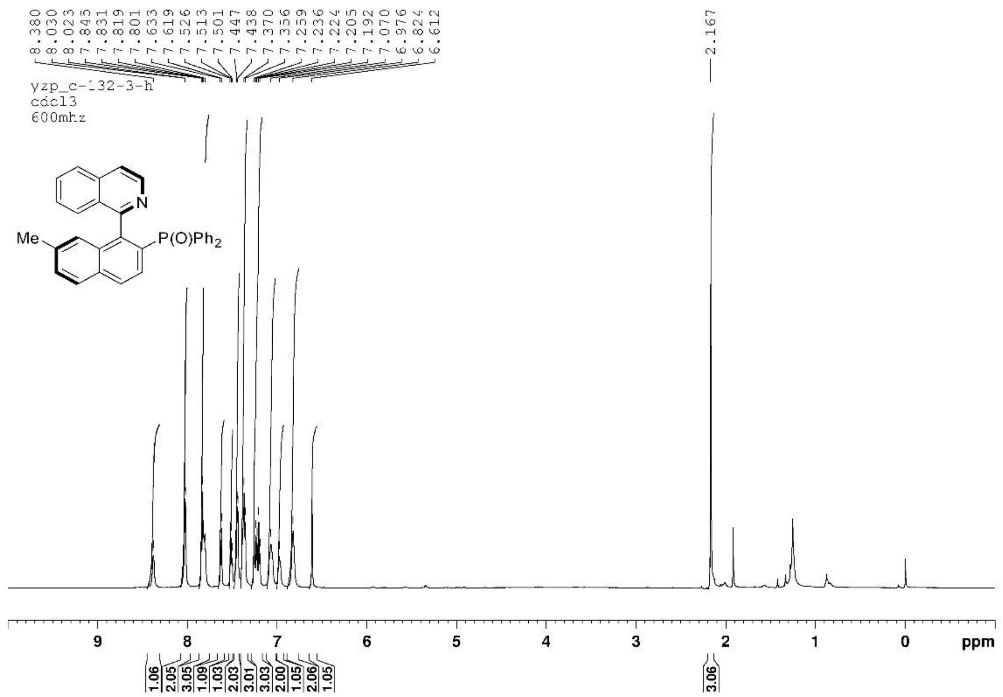

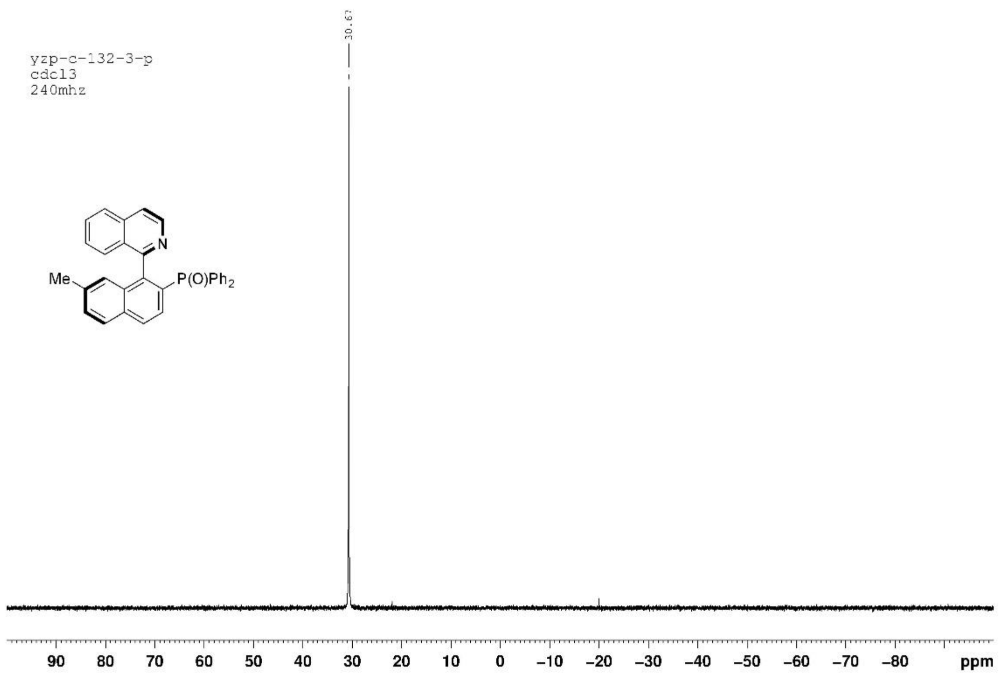

(*R*)-(1-(Isoquinolin-1-yl)-7-methoxynaphthalen-2-yl)diphenylphosphine oxide (3da)

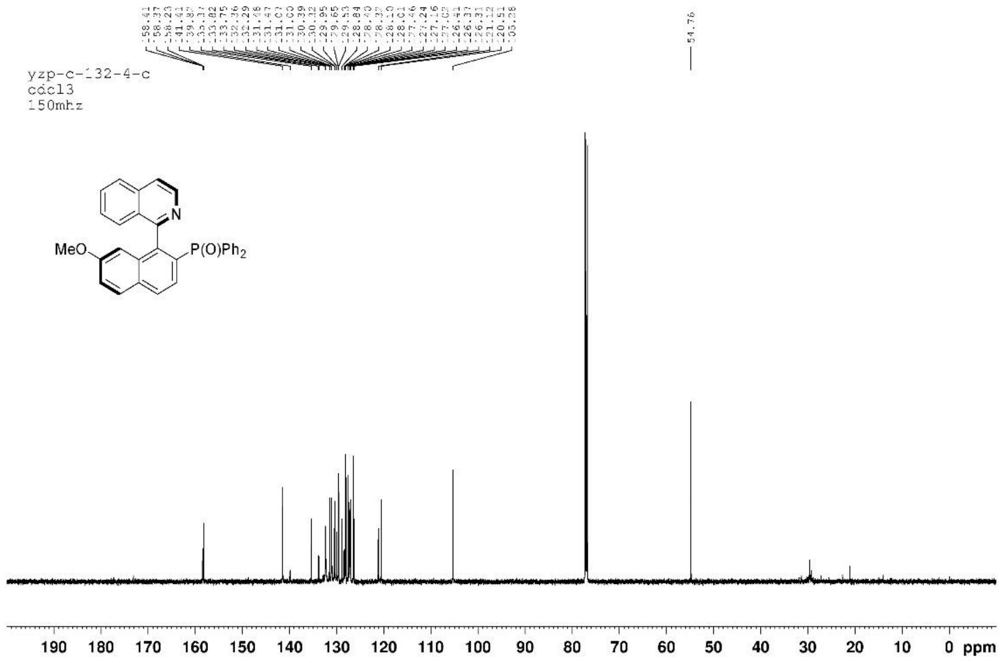

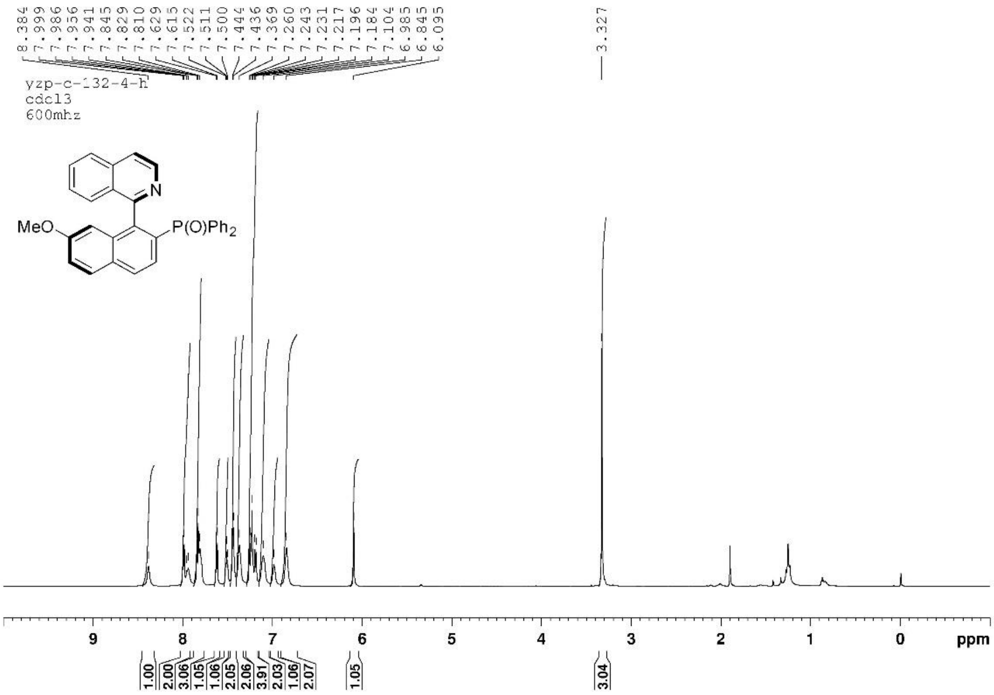
yzp-c-132-4-h
cdcl3
600mhz
MeO
P(O)Ph2
N
8.384
7.999
7.986
7.956
7.941
7.845
7.829
7.810
7.629
7.615
7.522
7.511
7.500
7.444
7.436
7.369
7.260
7.243
7.231
7.217
7.196
7.184
7.104
6.985
6.845
6.095
3.327
9
8
7
6
5
4
3
2
1
0
ppm
1.00
2.00
3.06
1.05
1.06
1.06
2.05
2.06
3.91
2.03
1.06
2.07
1.05
3.04

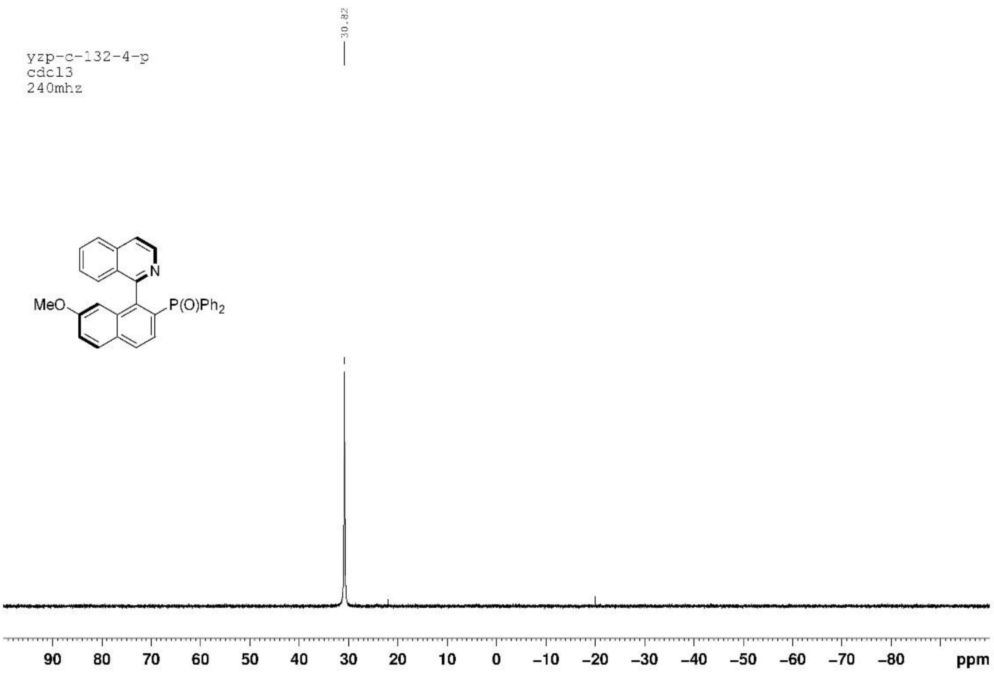
yzp-c-132-4-p
cdcl3
240mhz
30.82
MeO
P(O)Ph2
N
90
80
70
60
50
40
30
20
10
0
−10
−20
−30
−40
−50
−60
−70
−80
ppm

(*R*)-(1-(Isoquinolin-1-yl)-6-methoxynaphthalen-2-yl)diphenylphosphine oxide (3ea)

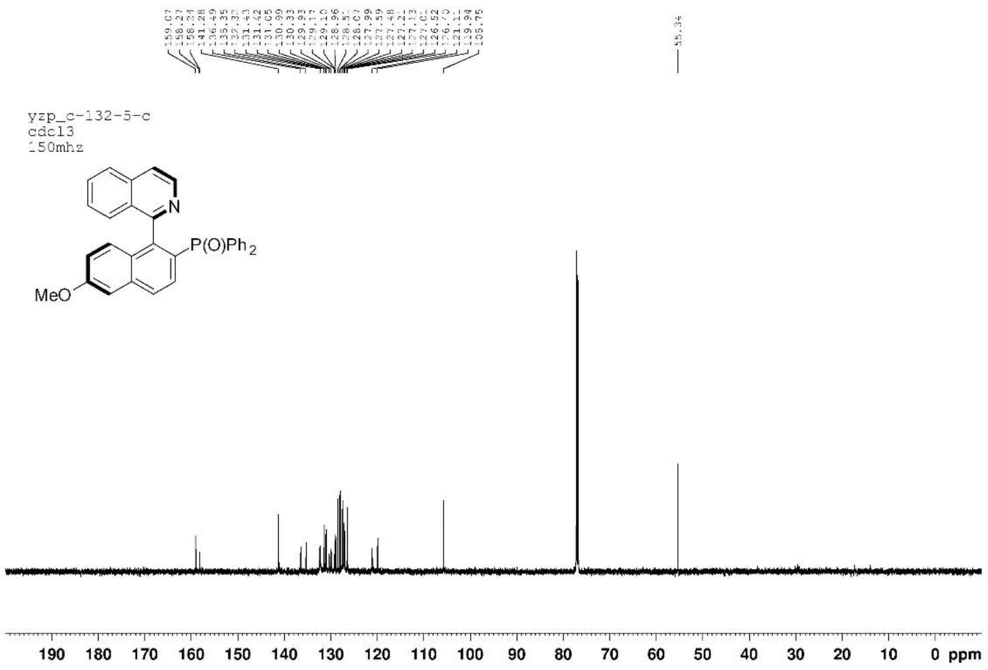

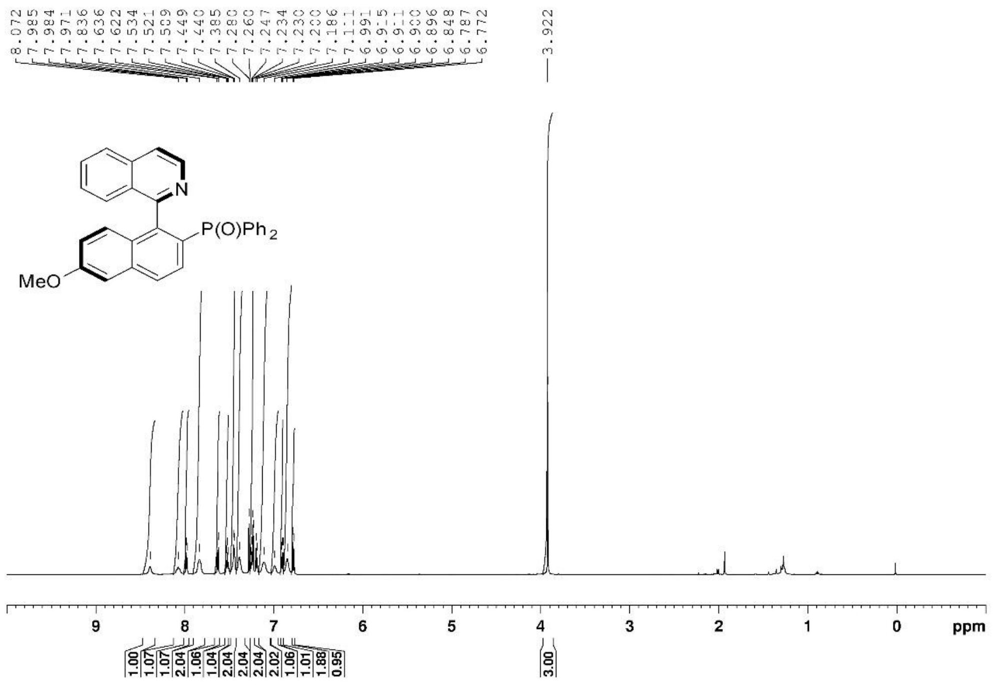

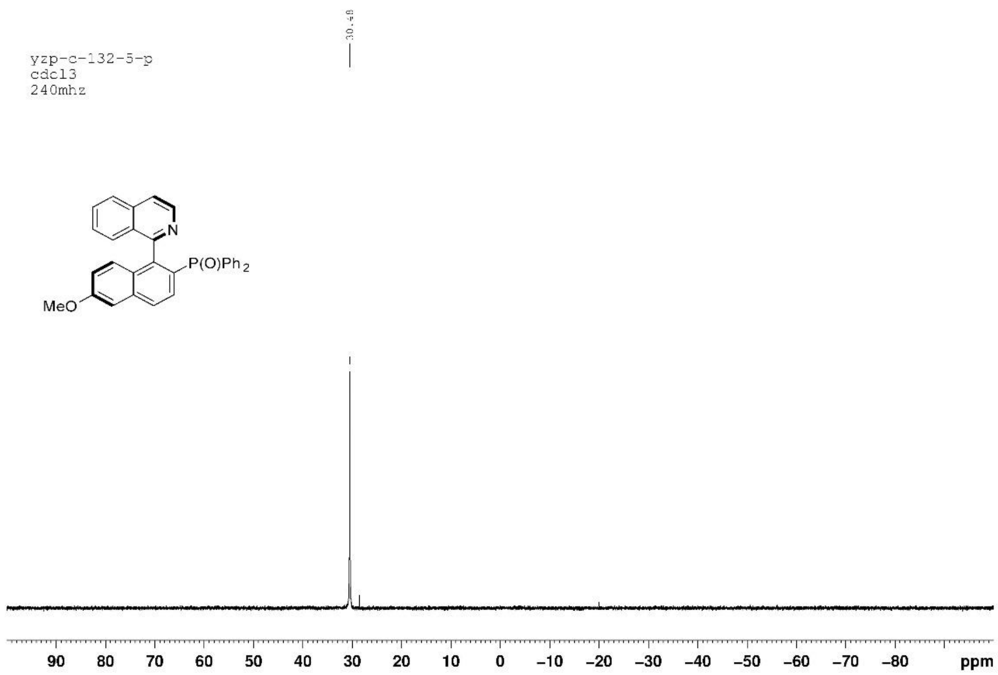

(*R*)-Methyl 6-(diphenylphosphoryl)-5-(isoquinolin-1-yl)-2-naphthoate (3fa)

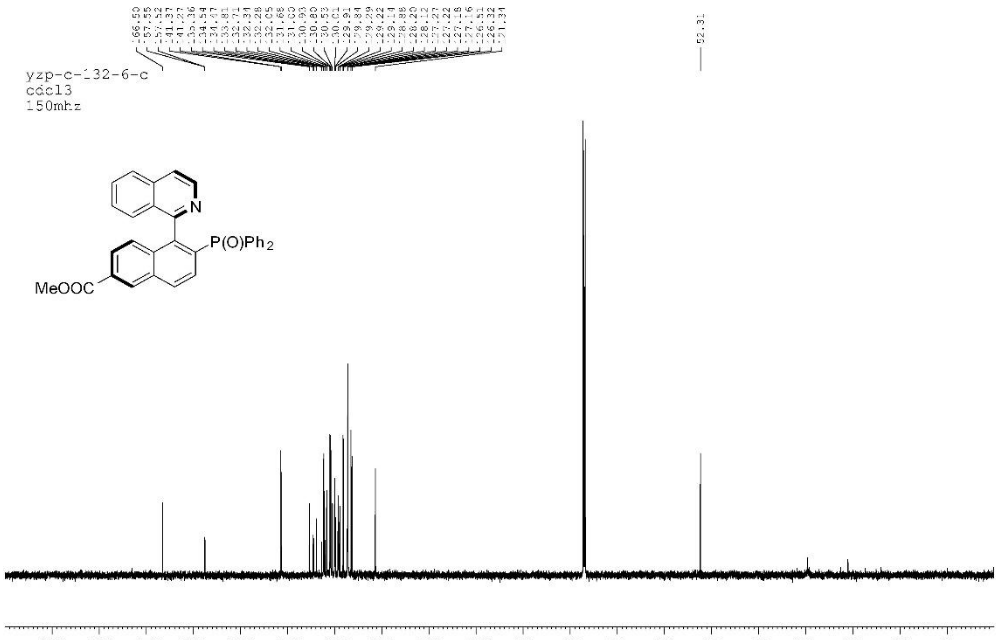

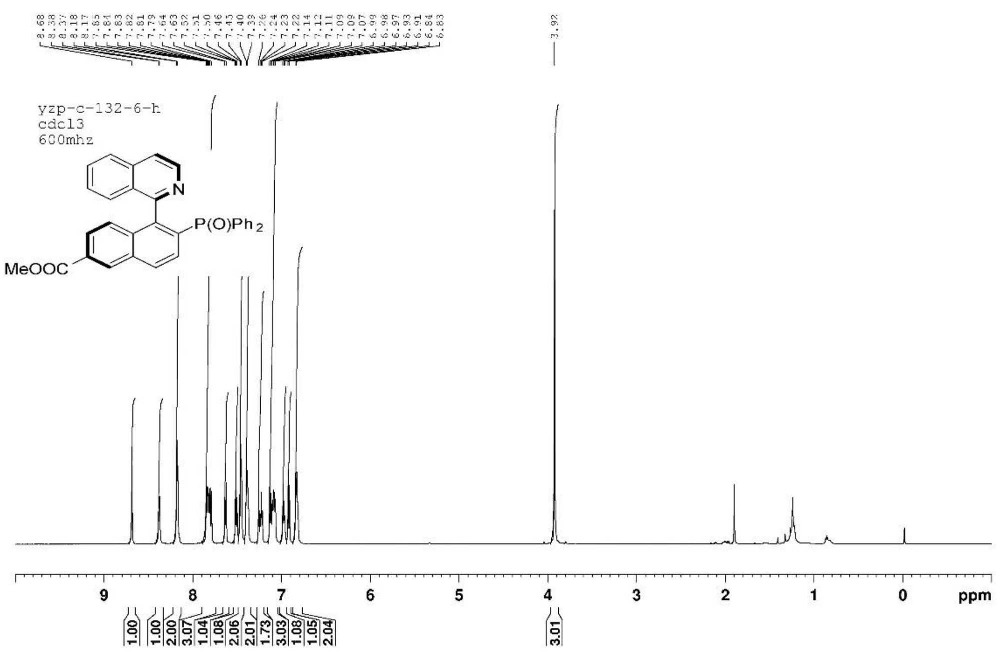
yzp-c-132-6-h
cdcl3
600mhz
P(O)Ph2
MeOOC
N
ppm

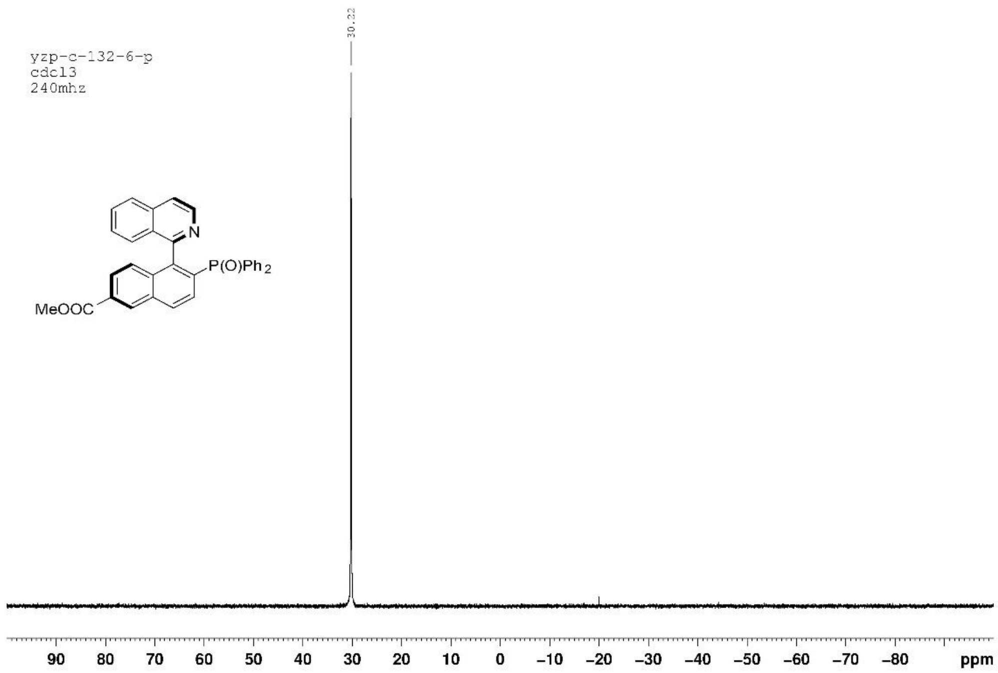
yzp-c-132-6-p
cdcl3
240mhz
P(O)Ph2
MeOOC
N
ppm

(*R*)-(1-(6-Isopropylisoquinolin-1-yl)naphthalen-2-yl)diphenylphosphine oxide (3ga)

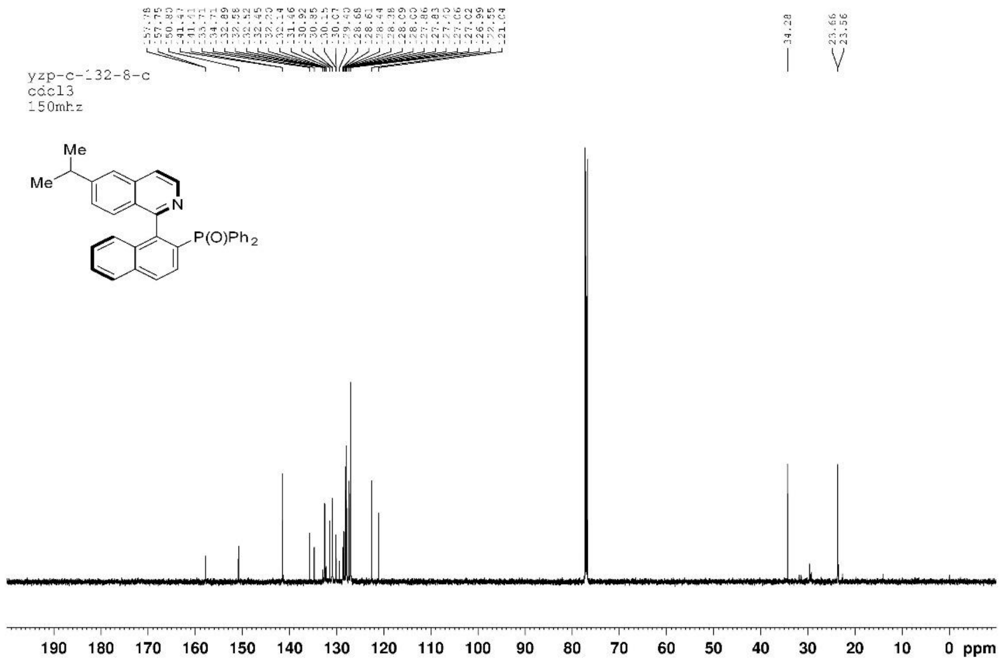

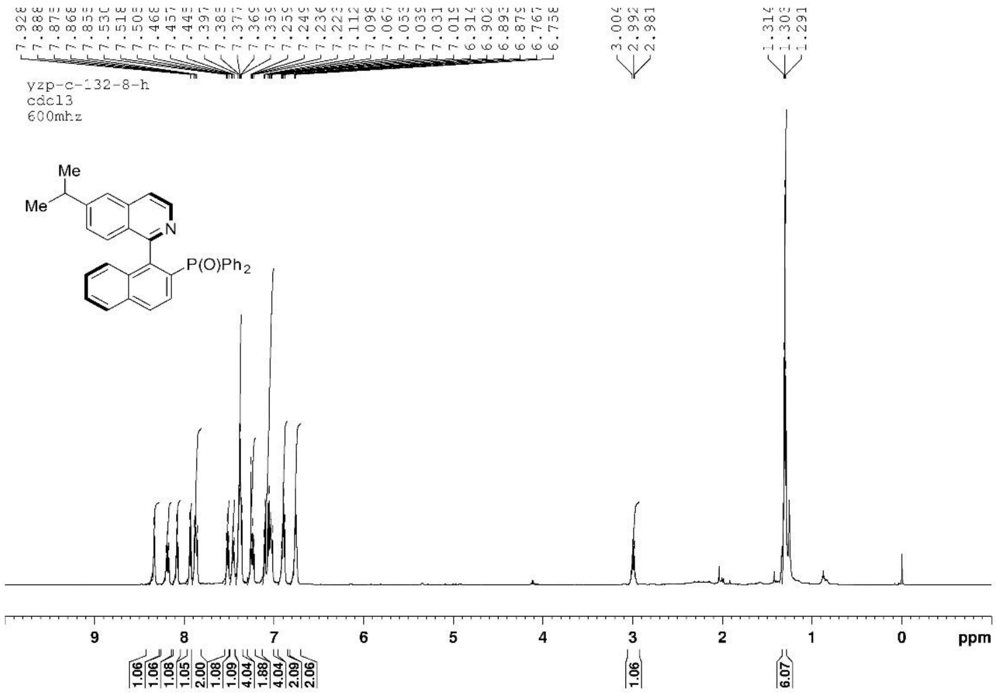

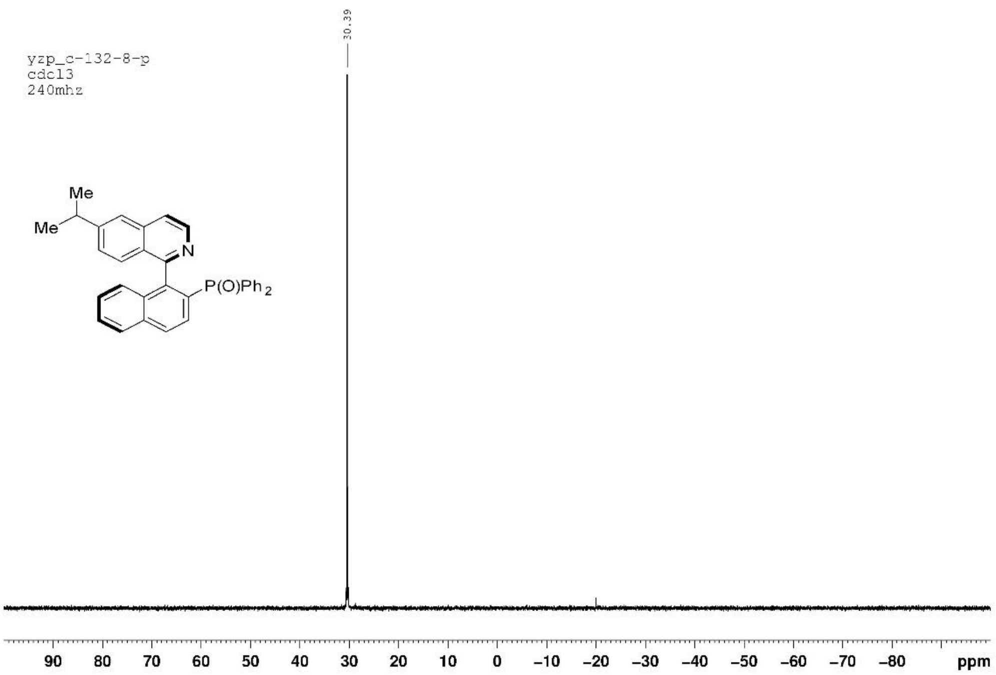

(*R*)-Diphenyl(1-(4-phenylisoquinolin-1-yl)naphthalen-2-yl)phosphine oxide (3ha)

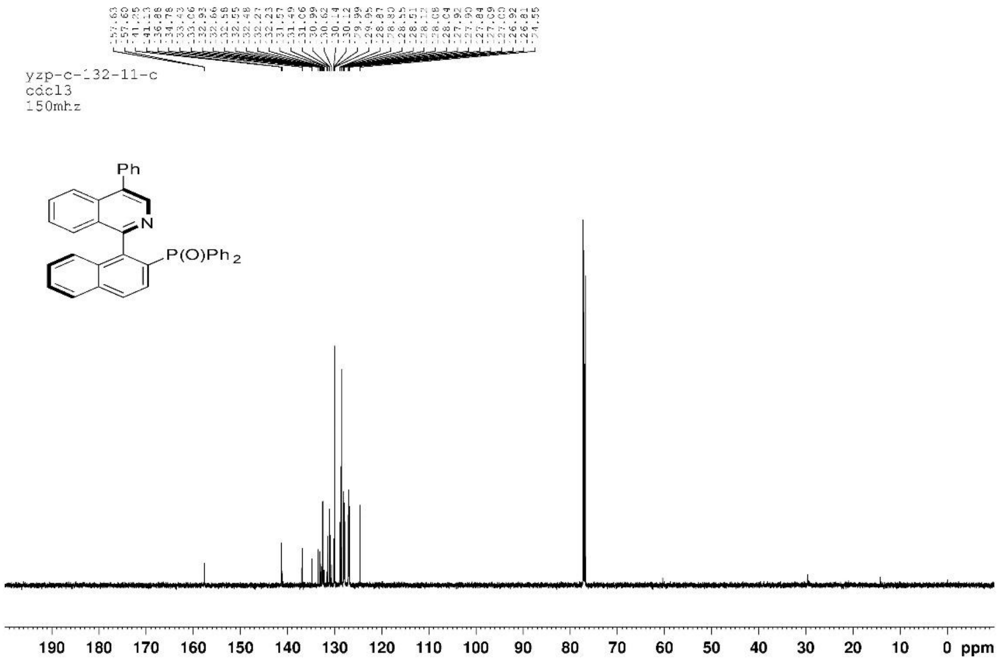

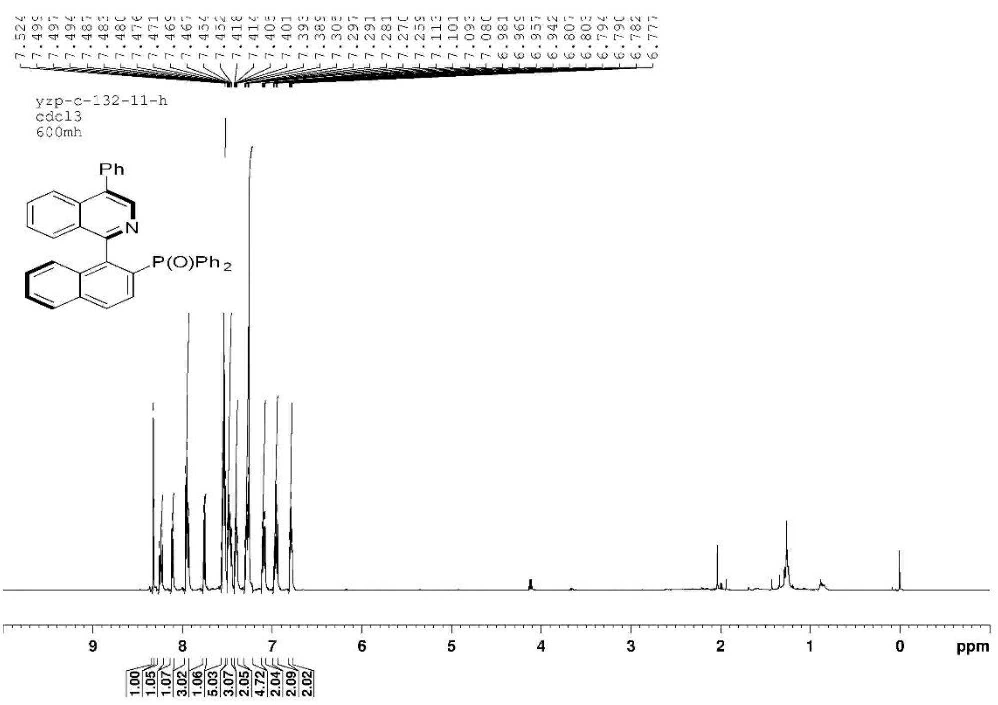
yzp-c-132-11-h
cdcl3
600mh
Ph
N
P(O)Ph 2
ppm

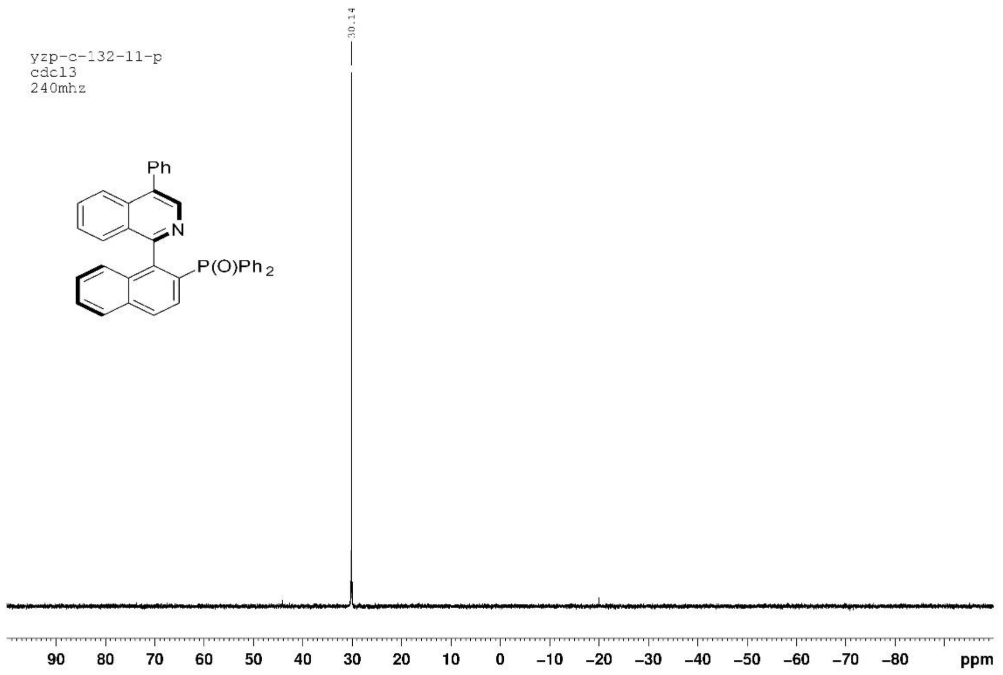
30.14
yzp-c-132-11-p
cdcl3
240mhz
Ph
N
P(O)Ph 2
ppm

(*R*)-Diphenyl(1-(5-phenylisoquinolin-1-yl)naphthalen-2-yl)phosphine oxide (3ia)

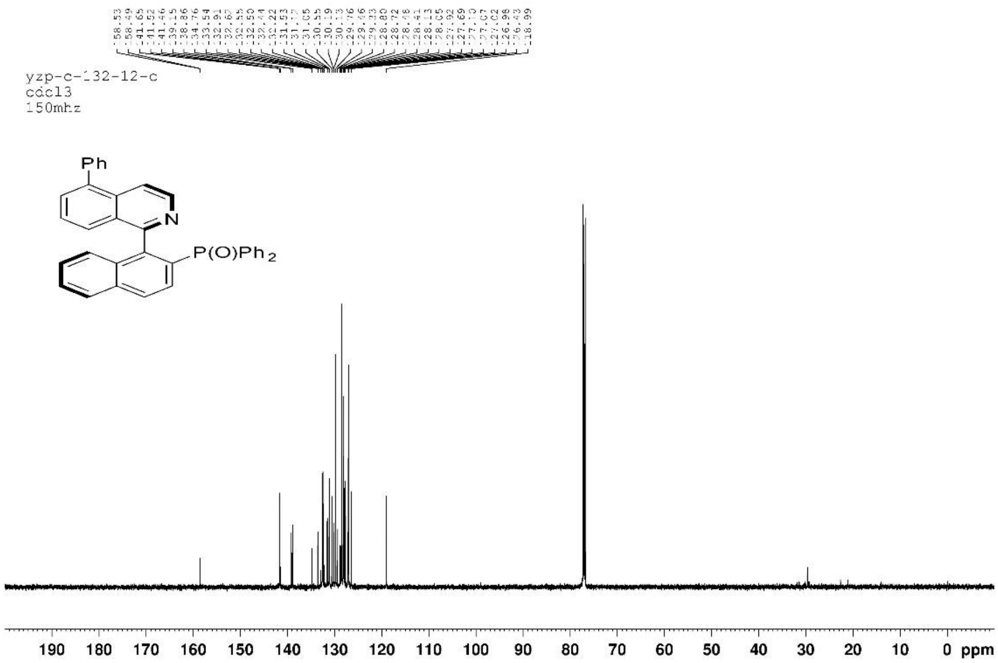

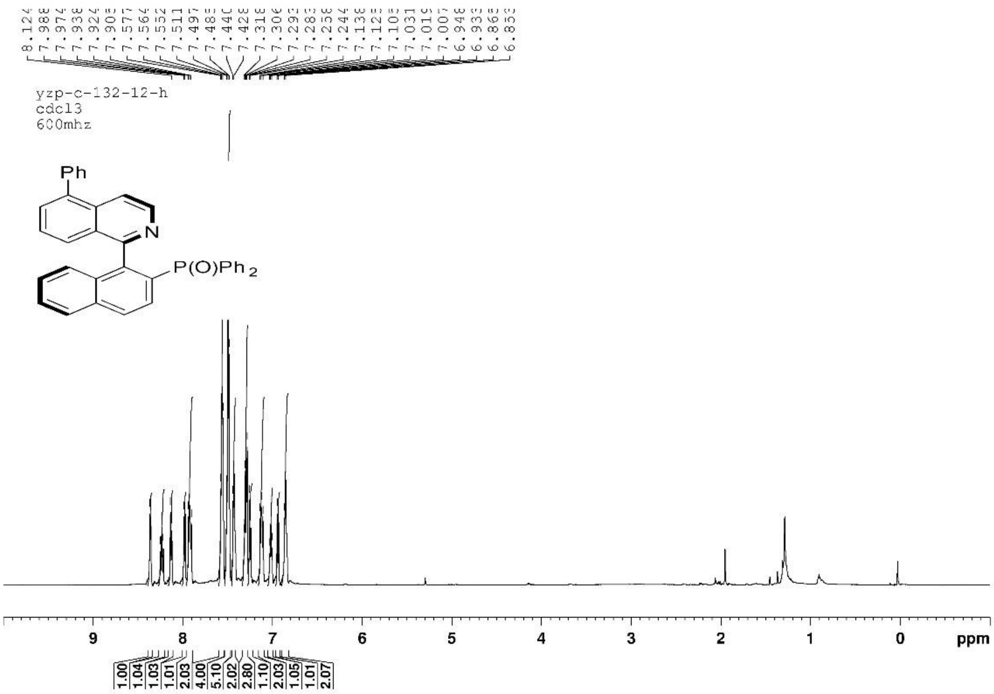

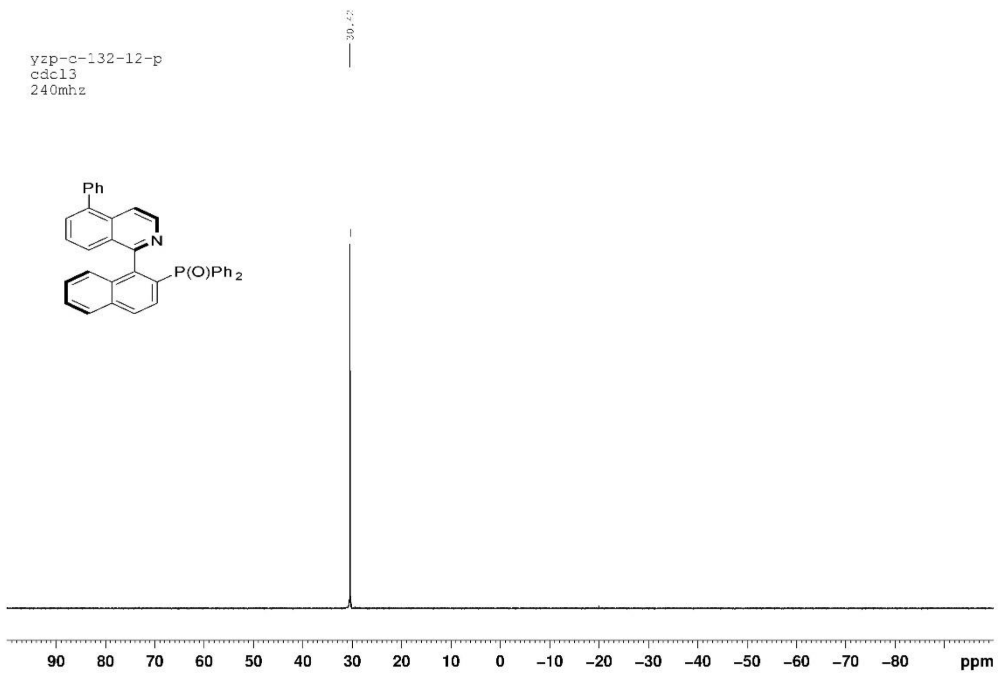

(*R*)-Diphenyl(1-(6-phenylisoquinolin-1-yl)naphthalen-2-yl)phosphine oxide (3ja)

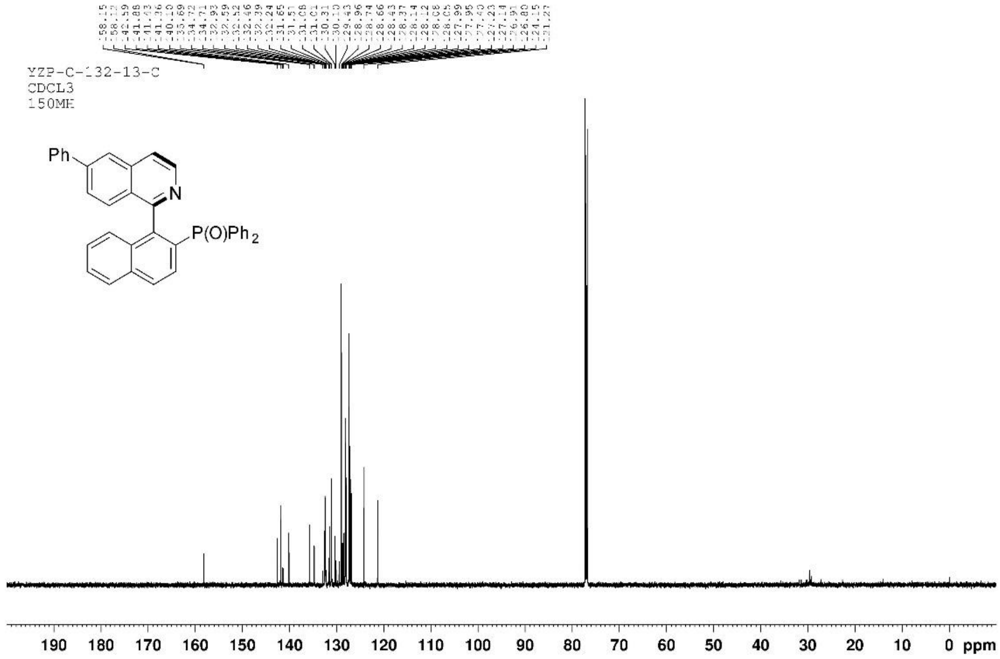

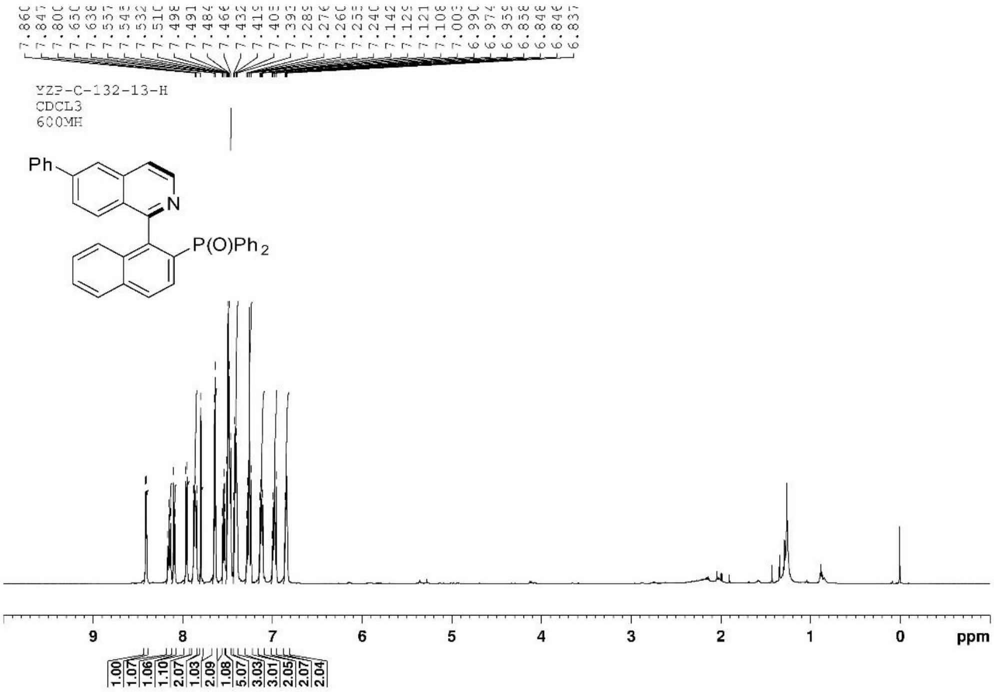
YZP-C-132-13-H
CDCL3
600MH
Ph
N
P(O)Ph2
ppm

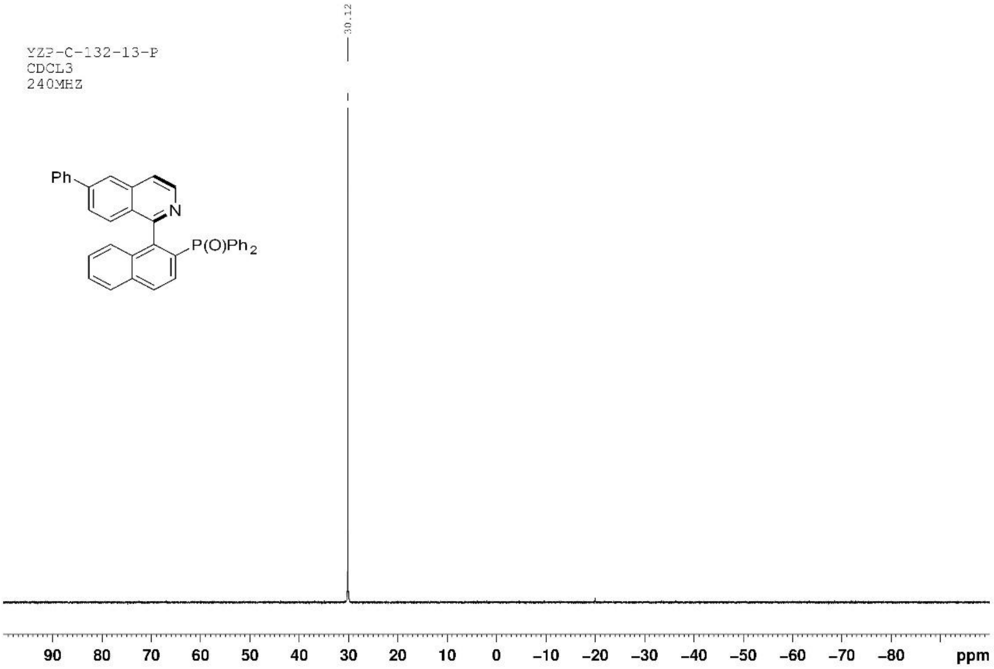
30.12
YZP-C-132-13-P
CDCL3
240MHZ
Ph
N
P(O)Ph2
ppm

(*R*)-Diphenyl(1-(7-phenylisoquinolin-1-yl)naphthalen-2-yl)phosphine oxide (3ka)

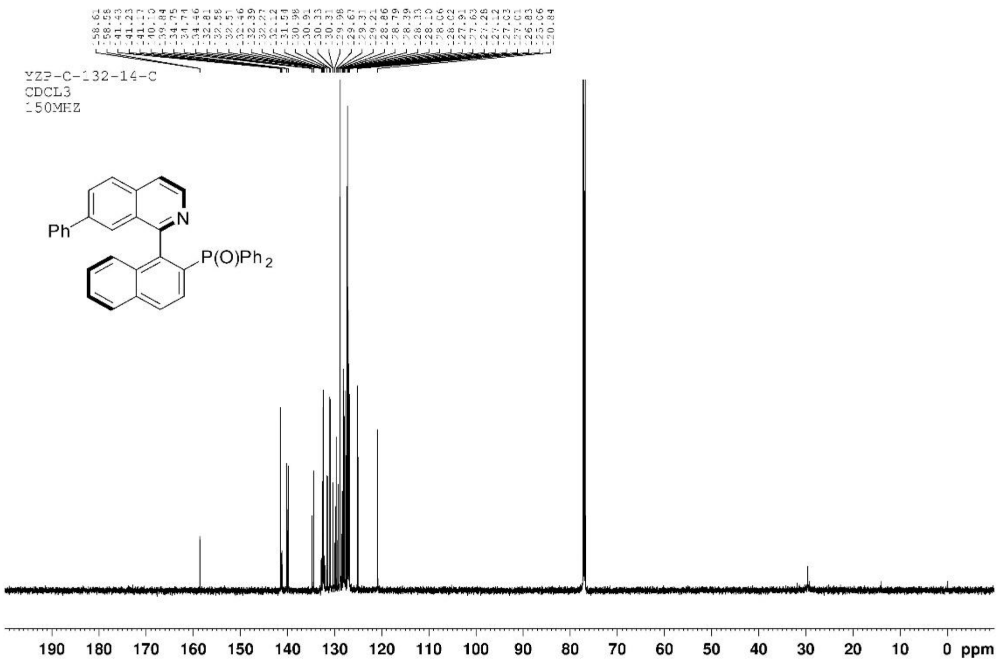

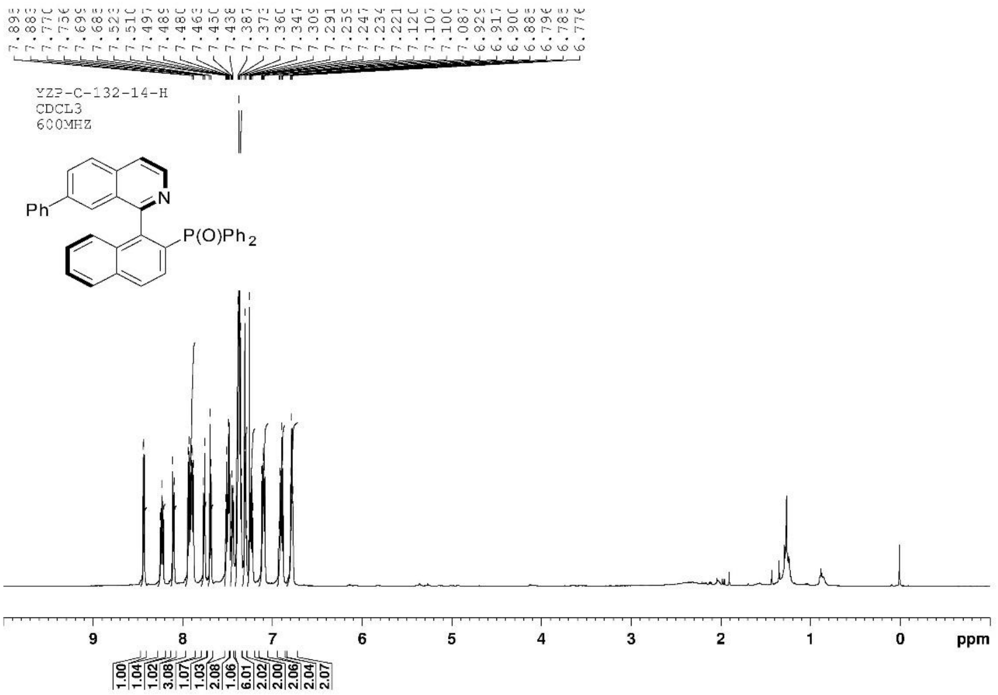

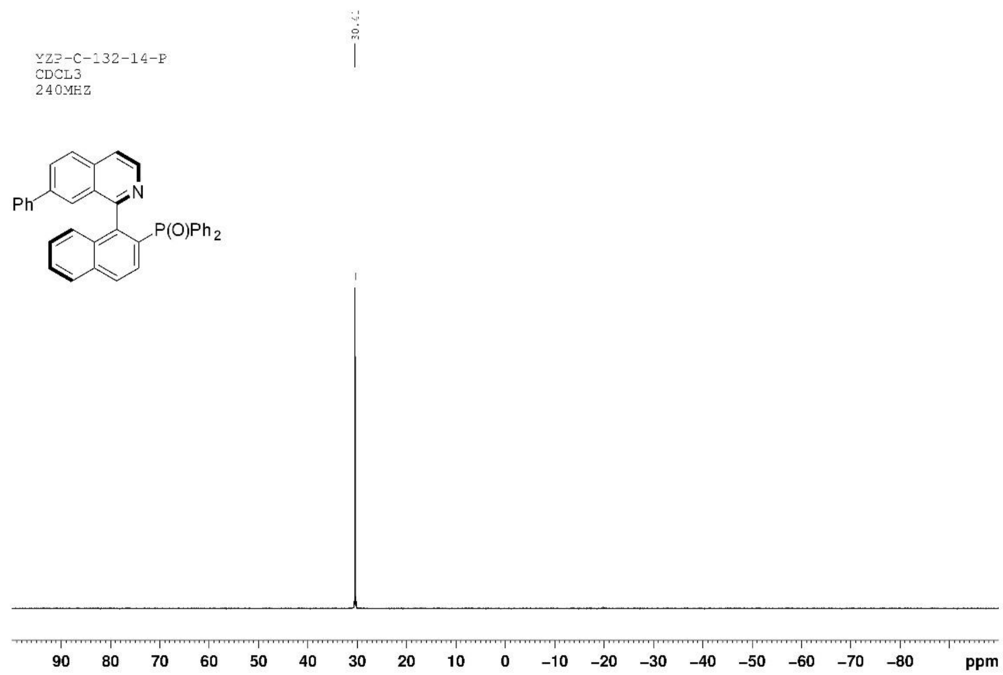

(*R*)-(1-(Isoquinolin-1-yl)-7-phenylnaphthalen-2-yl)diphenylphosphine oxide (3la)

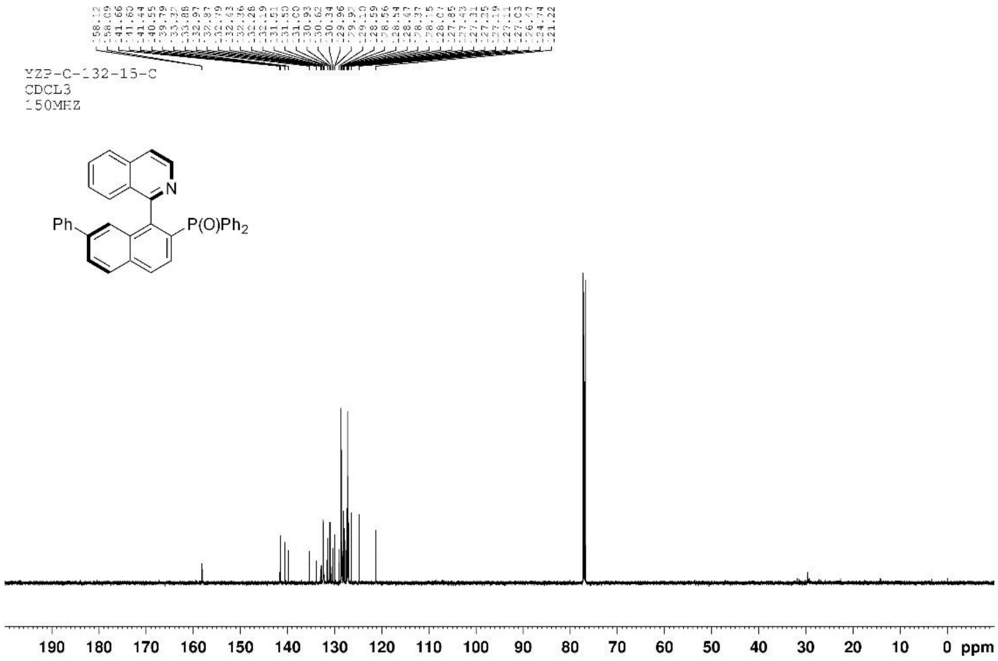

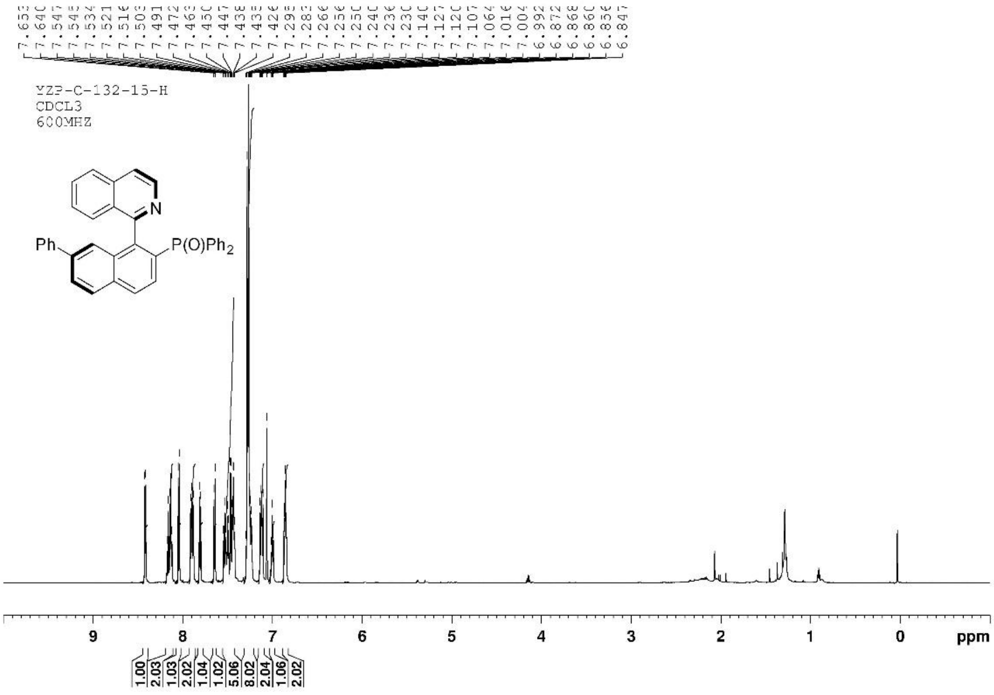
YZP-C-132-15-H
CDCL3
600MHZ
N
Ph
P(O)Ph2
9
8
7
6
5
4
3
2
1
0
ppm
1.00
2.03
1.03
2.02
1.04
1.02
5.06
8.02
2.04
1.06
2.02

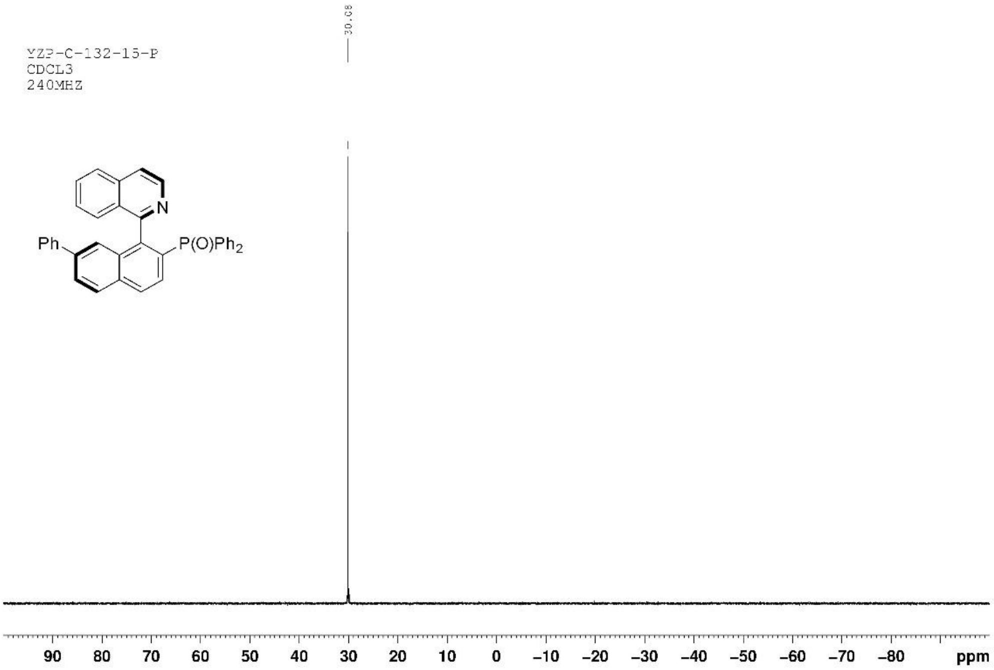
30.08
YZP-C-132-15-P
CDCL3
240MHZ
N
Ph
P(O)Ph2
90
80
70
60
50
40
30
20
10
0
−10
−20
−30
−40
−50
−60
−70
−80
ppm

(*R*)-(1-(Isoquinolin-1-yl)-6-phenylnaphthalen-2-yl)diphenylphosphine oxide (3ma)

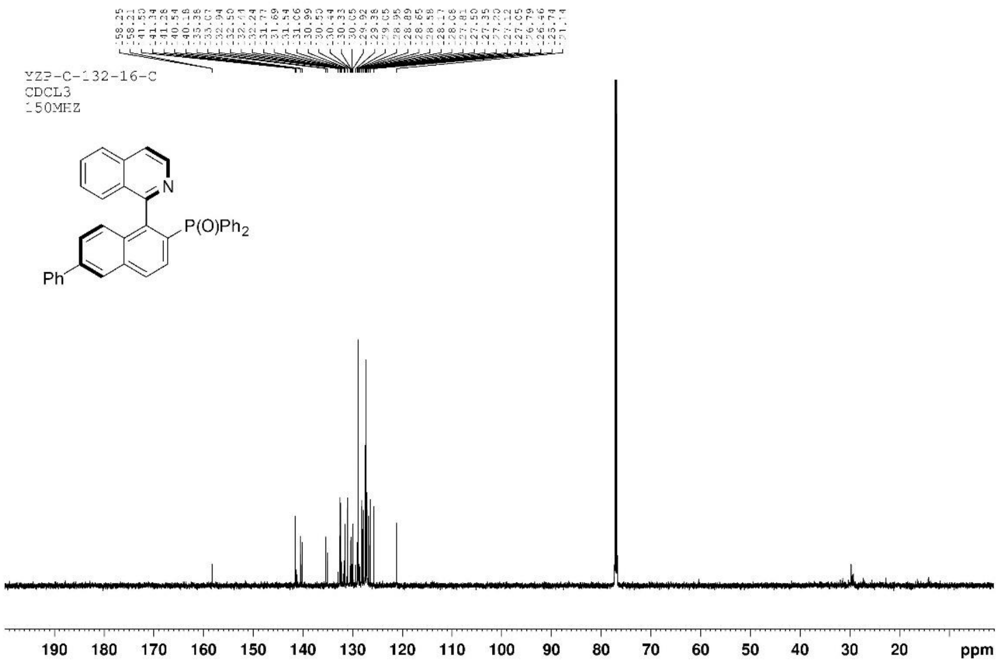

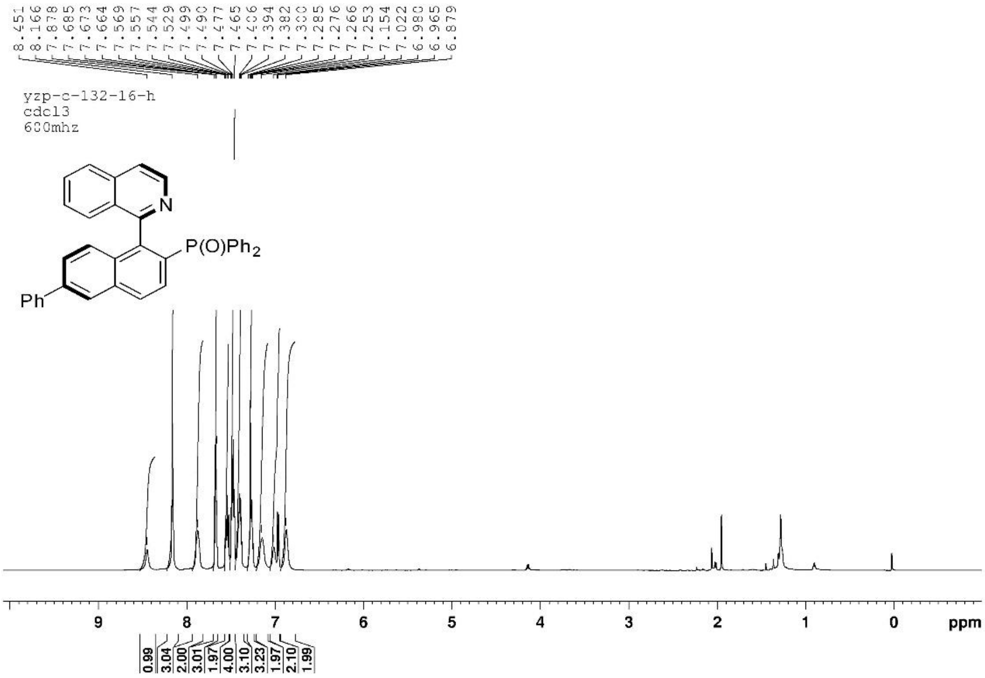

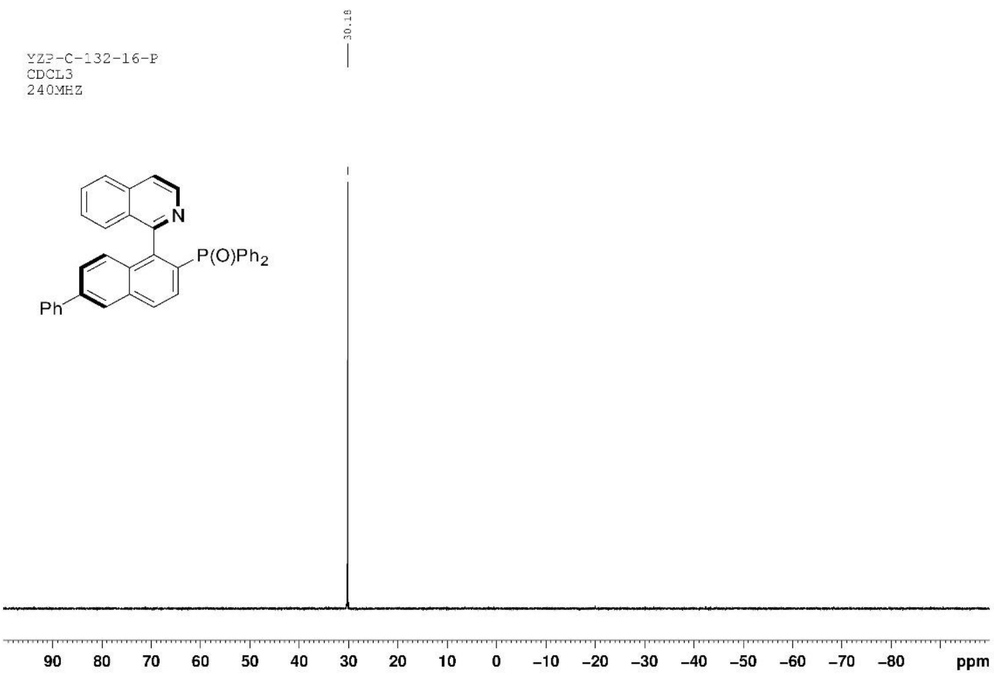

(*R*)-(1-(Isoquinolin-1-yl)-4-phenylnaphthalen-2-yl)diphenylphosphine oxide (3na)

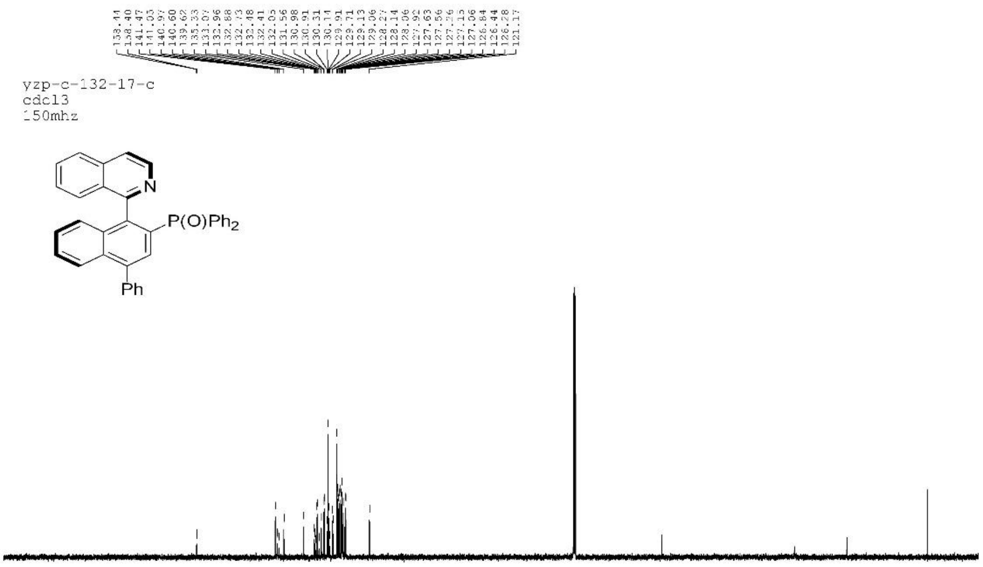

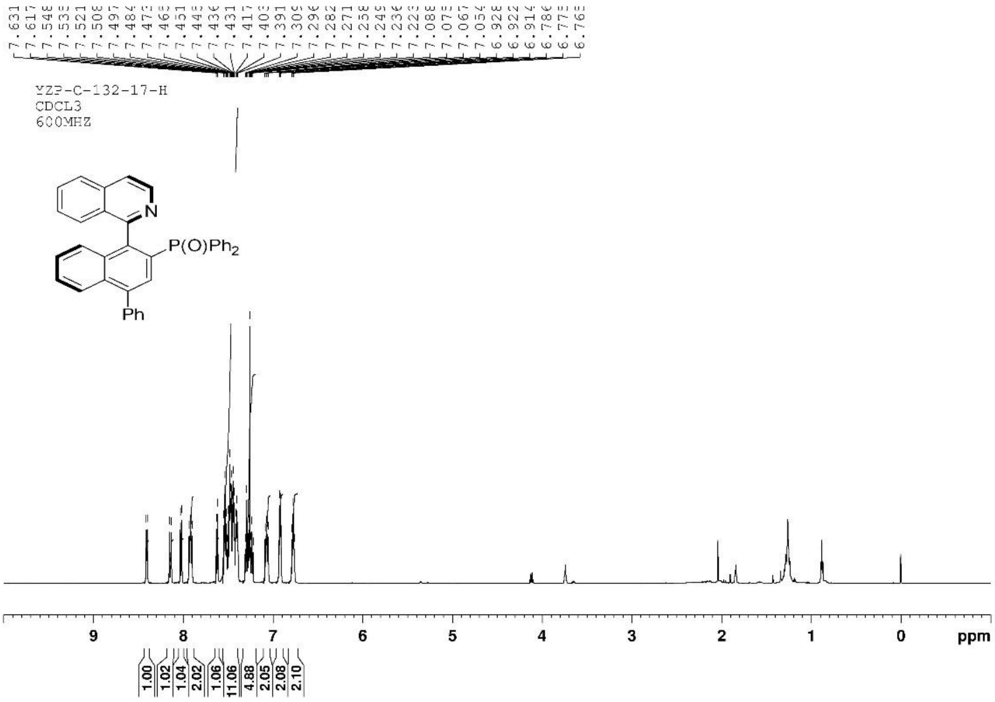
YZP-C-132-17-H
CDCL3
600MHZ
N
P(O)Ph2
Ph
ppm

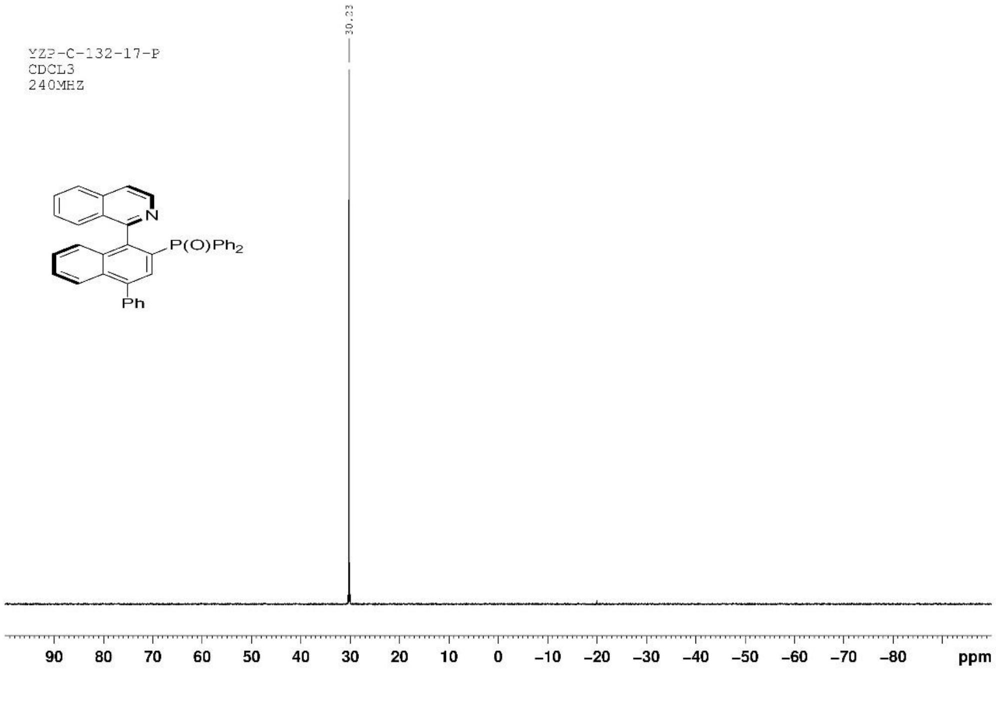
30.23
YZP-C-132-17-P
CDCL3
240MHZ
N
P(O)Ph2
Ph
ppm

(*R*)-(1-(Isoquinolin-1-yl)-6-(p-tolyl)naphthalen-2-yl)diphenylphosphine oxide (3oa)

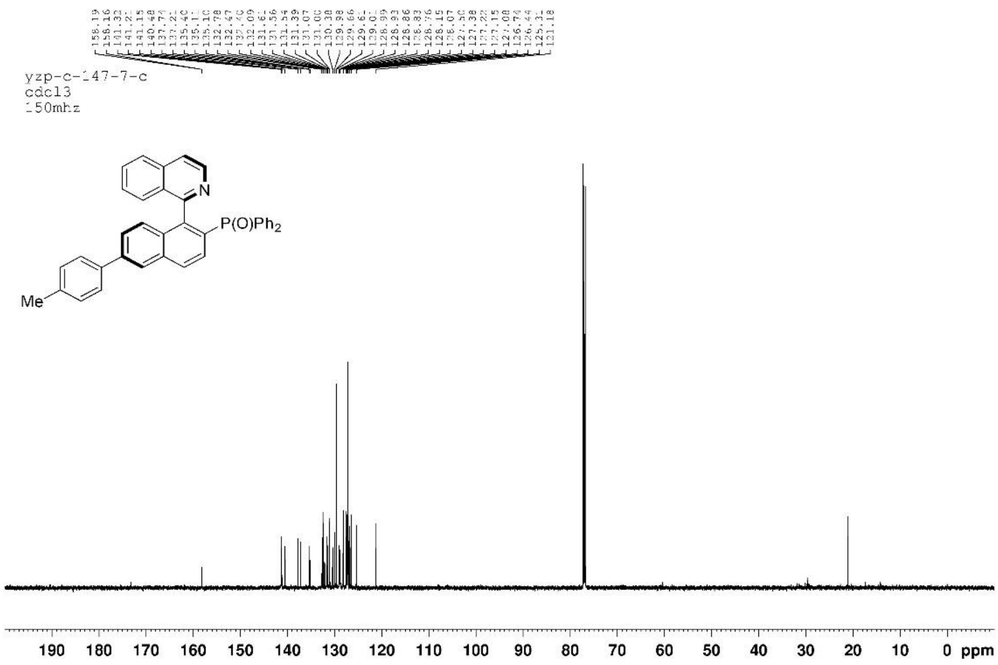

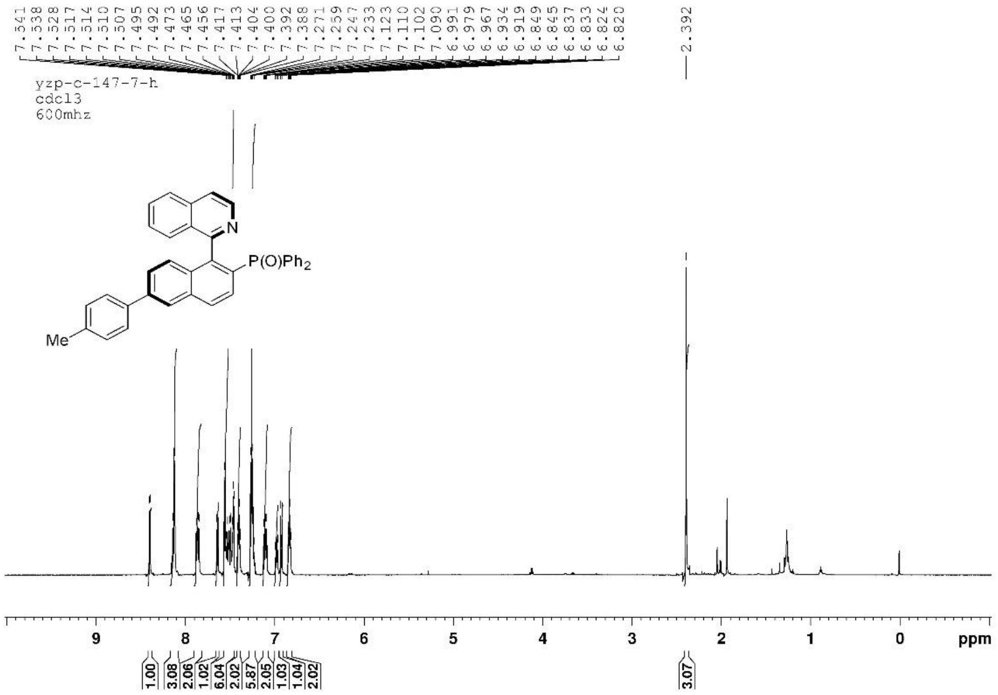

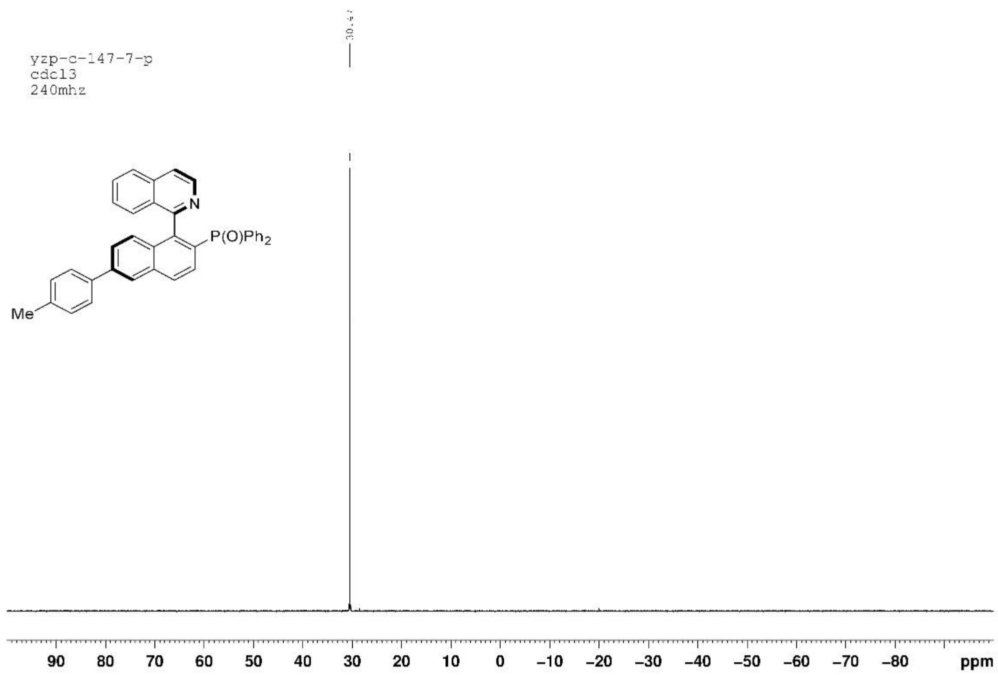

(*R*)-(6-(3,5-Dimethylphenyl)-1-(isoquinolin-1-yl)naphthalen-2-yl)diphenylphosphine oxide (3pa)

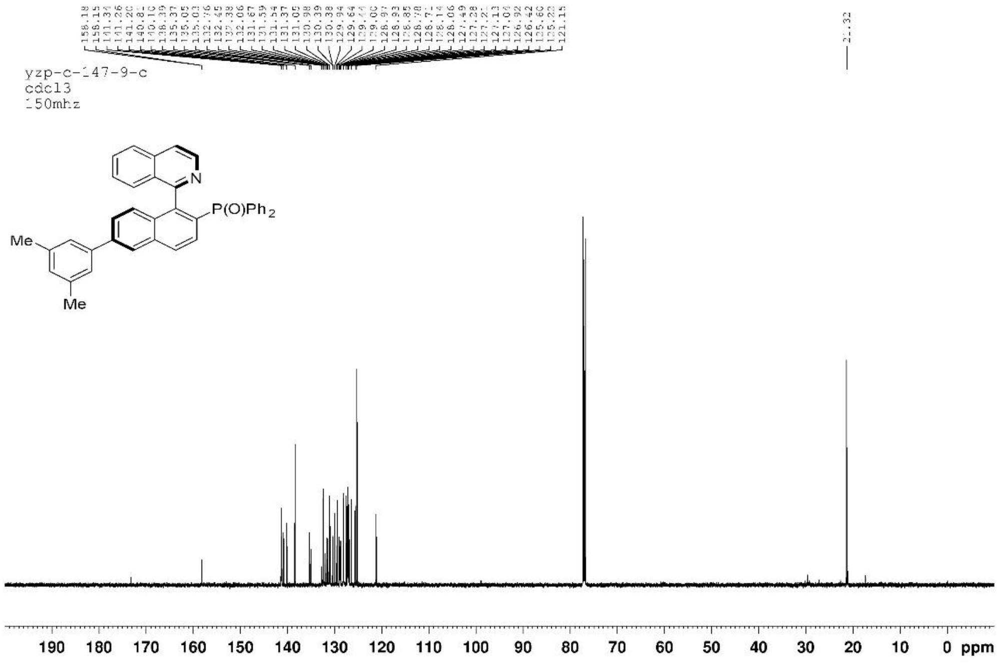

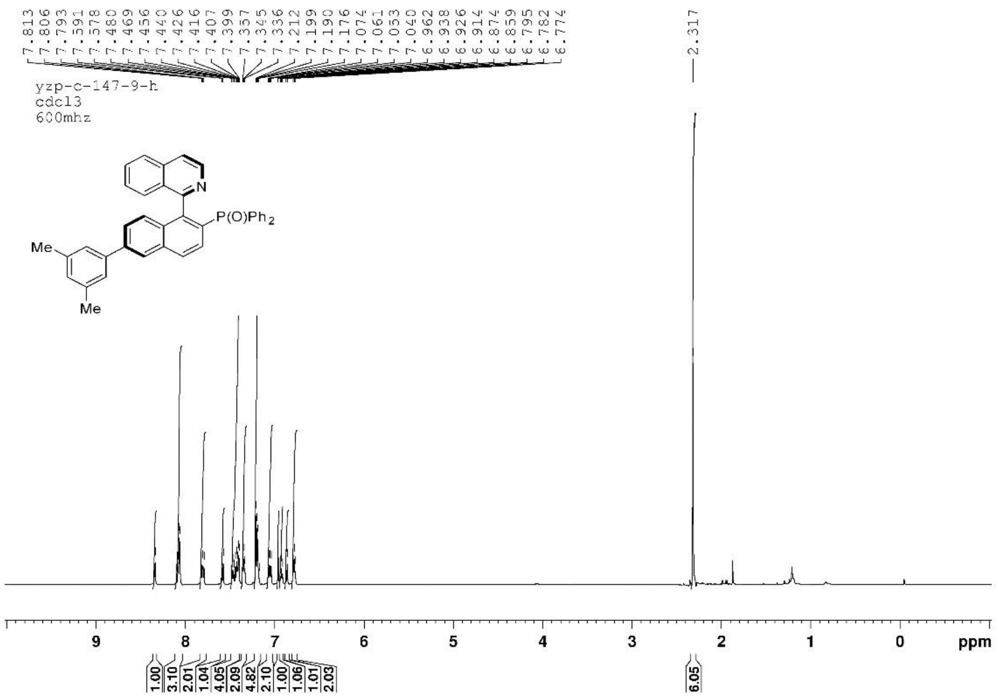
7.813
7.806
7.793
7.591
7.578
7.480
7.469
7.456
7.440
7.426
7.416
7.407
7.399
7.357
7.345
7.336
7.212
7.199
7.190
7.176
7.074
7.061
7.053
7.040
6.962
6.938
6.926
6.914
6.874
6.859
6.795
6.782
6.774
2.317
yzp-c-147-9-h
cdcl3
600mhz
N
P(O)Ph2
Me
Me
9
8
7
6
5
4
3
2
1
0
ppm
1.00
3.10
2.01
1.04
4.05
2.09
4.82
2.10
1.00
1.06
1.01
2.03
6.05

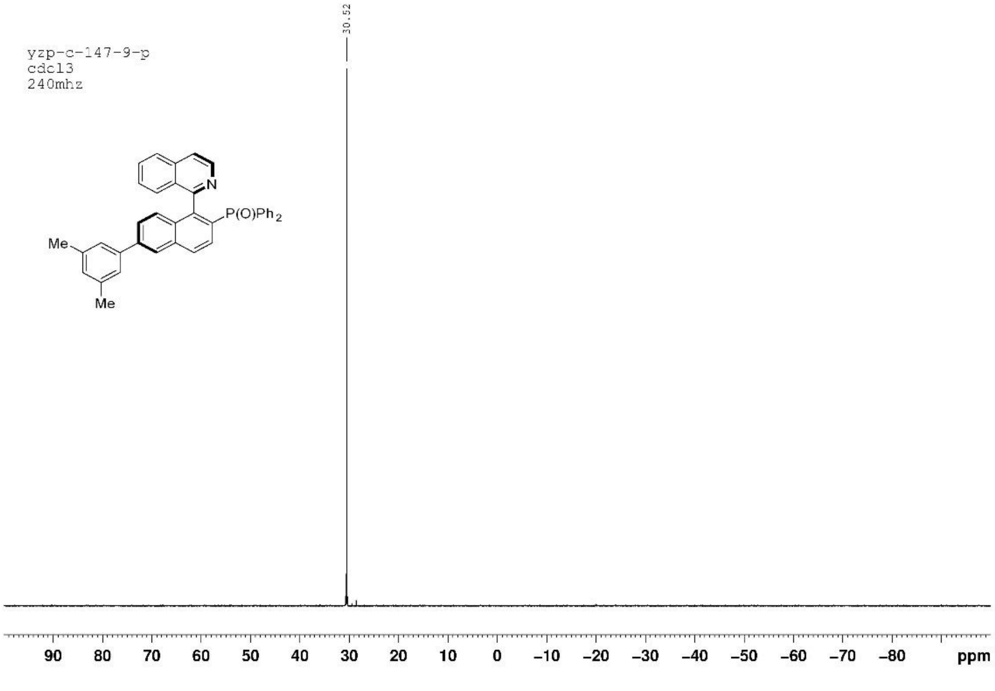
30.52
yzp-c-147-9-p
cdcl3
240mhz
N
P(O)Ph2
Me
Me
90
80
70
60
50
40
30
20
10
0
–10
–20
–30
–40
–50
–60
–70
–80
ppm

(*R*)-(1-(Isoquinolin-1-yl)-6-(4-methoxyphenyl)naphthalen-2-yl)diphenylphosphine oxide (3qa)

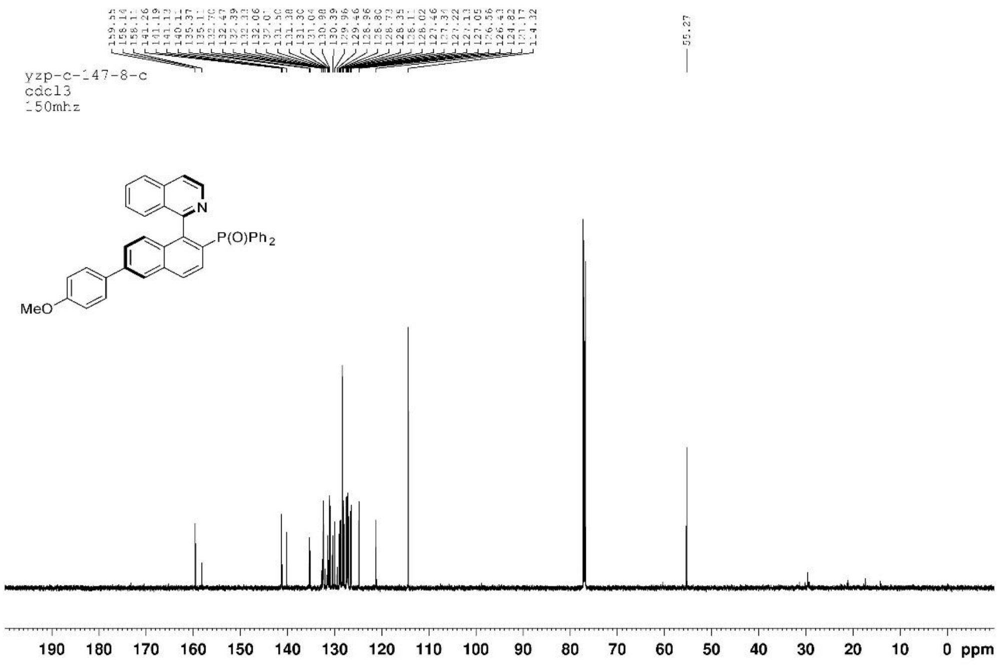

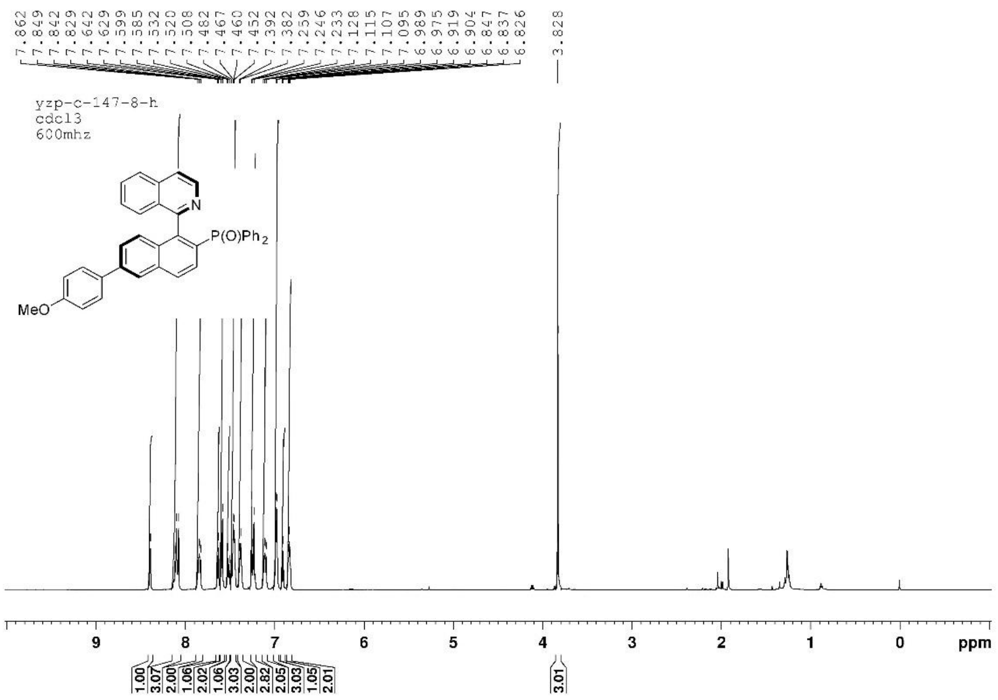

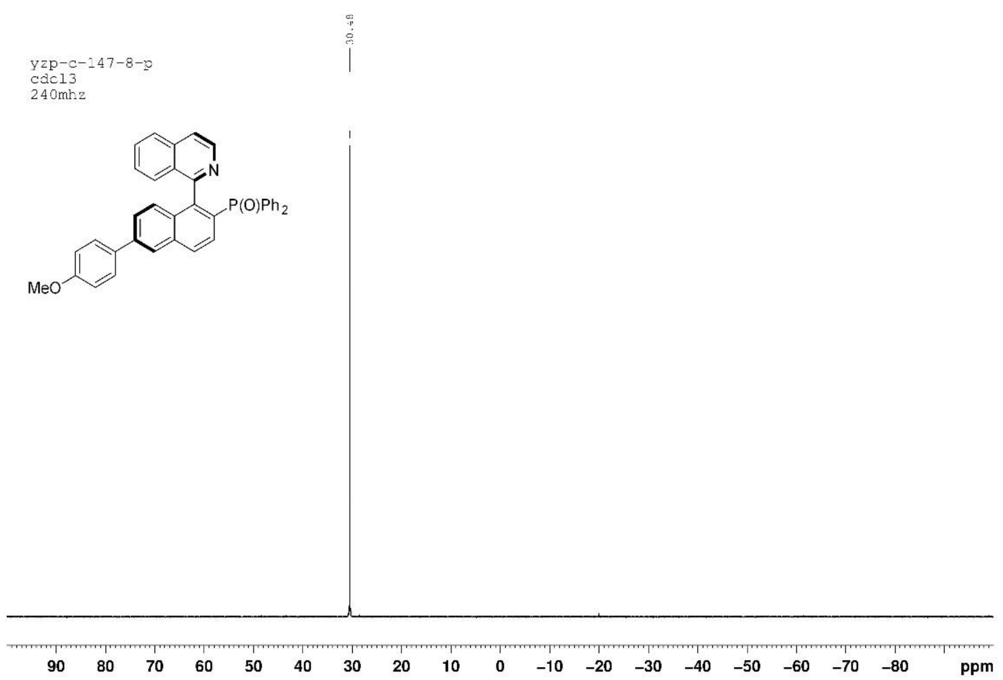

(*R*)-(6-(4-Fluorophenyl)-1-(isoquinolin-1-yl)naphthalen-2-yl)diphenylphosphine oxide (3ra)

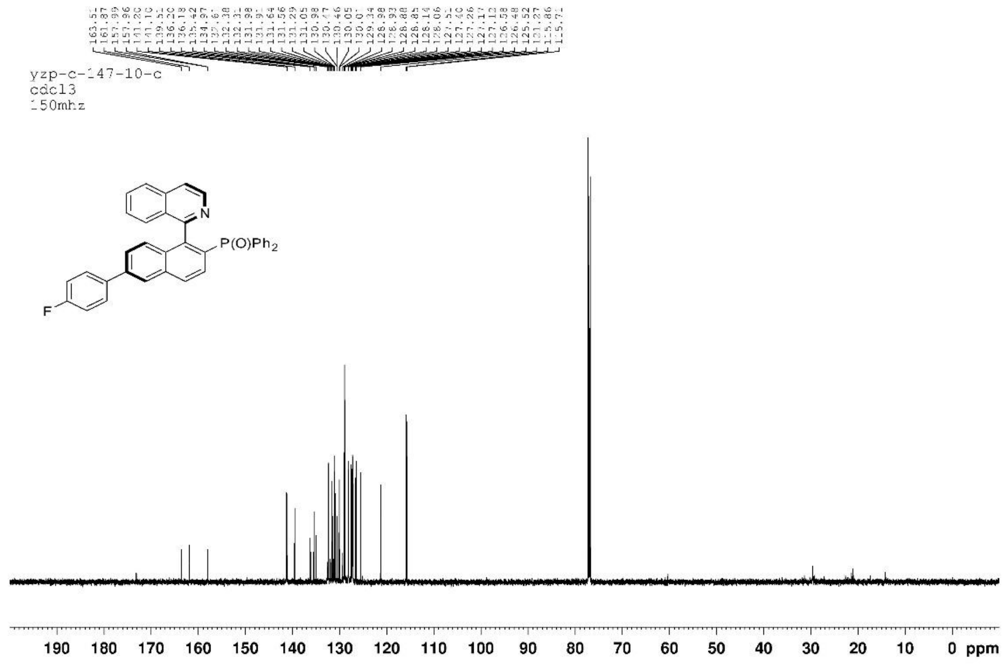

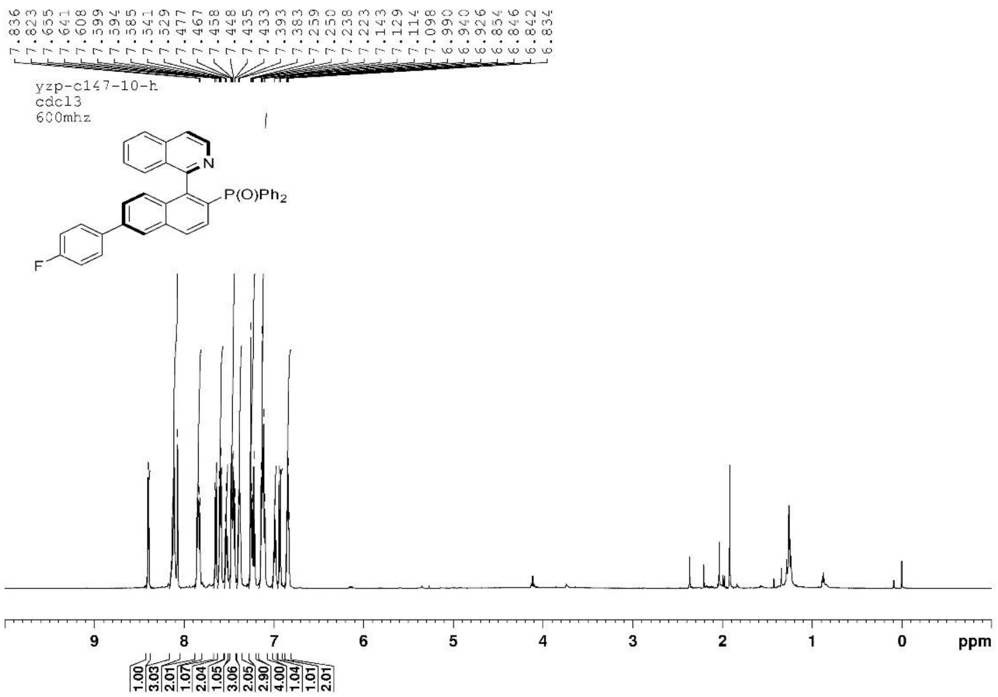
7.836
7.823
7.655
7.641
7.608
7.599
7.594
7.585
7.541
7.529
7.477
7.467
7.458
7.448
7.435
7.433
7.393
7.383
7.259
7.250
7.238
7.223
7.143
7.129
7.114
7.098
6.990
6.940
6.926
6.854
6.846
6.842
6.834
yzp-c147-10-h
cdcl3
600mhz
N
P(O)Ph2
F
9
8
7
6
5
4
3
2
1
0
ppm
1.00
3.03
2.01
1.07
2.04
1.05
3.06
2.05
2.90
4.00
1.04
1.01
2.01

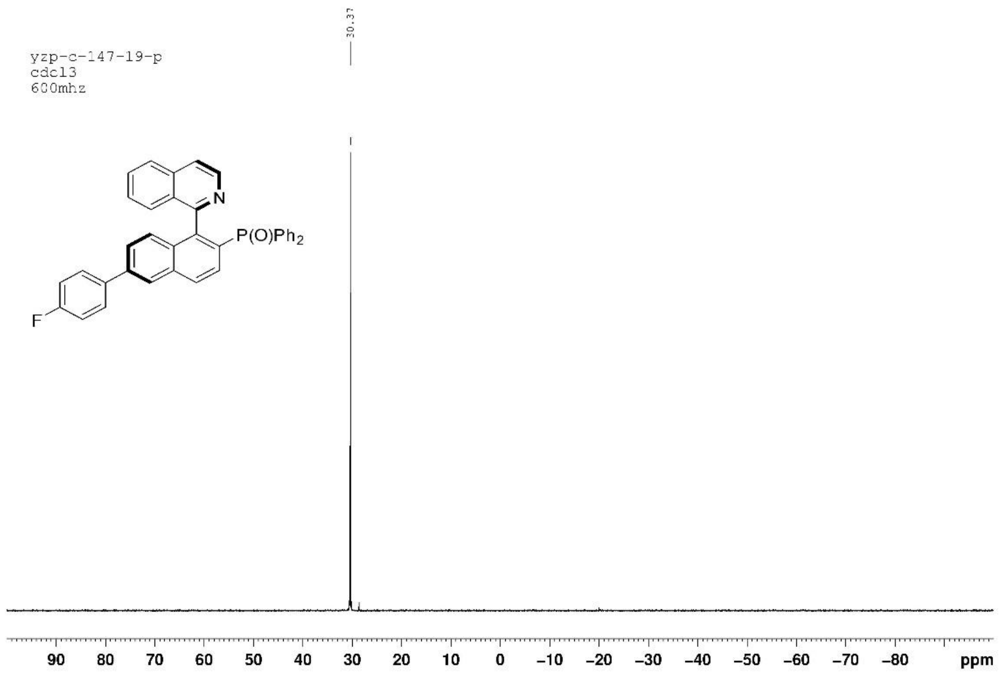
30.37
yzp-c-147-19-p
cdcl3
600mhz
N
P(O)Ph2
F
90
80
70
60
50
40
30
20
10
0
−10
−20
−30
−40
−50
−60
−70
−80
ppm

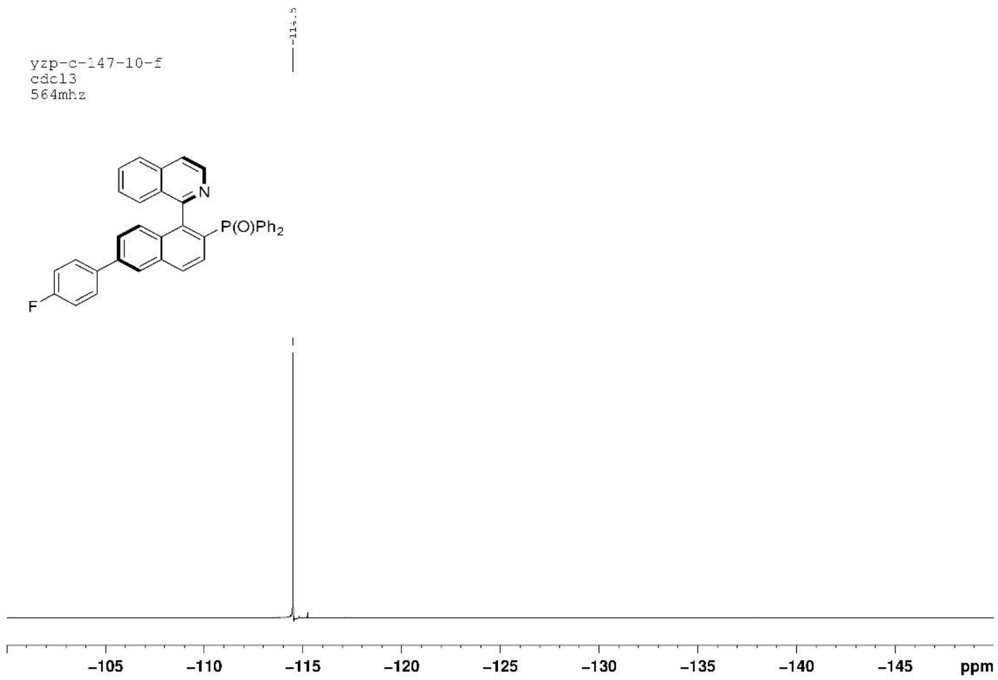

(*R*)-(5-(Isoquinolin-1-yl)-[2,2'-binaphthalen]-6-yl)diphenylphosphine oxide (3sa)

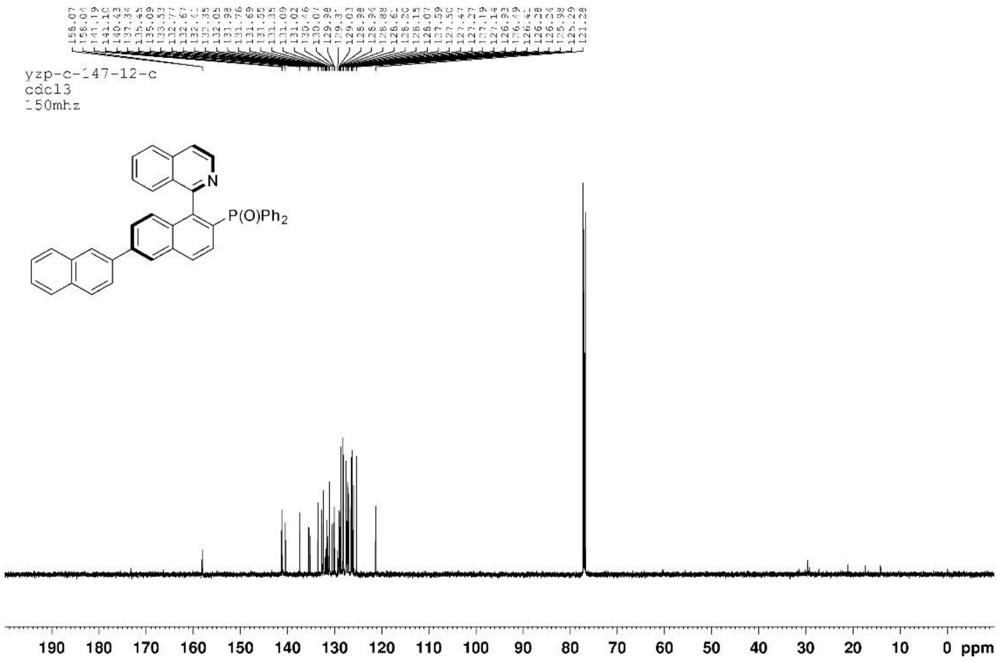

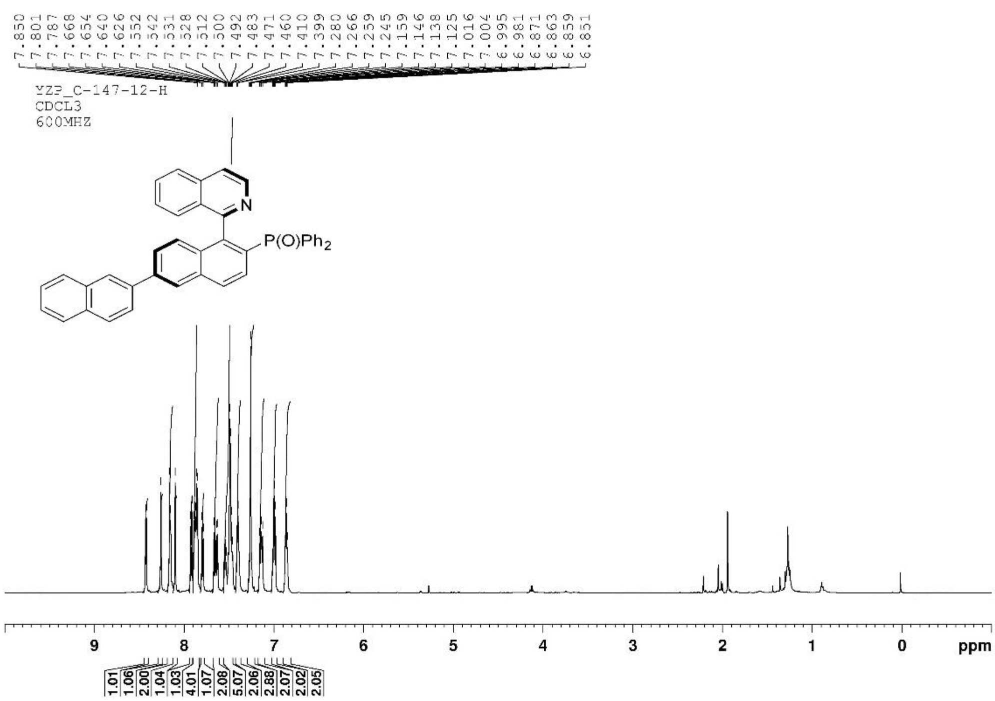

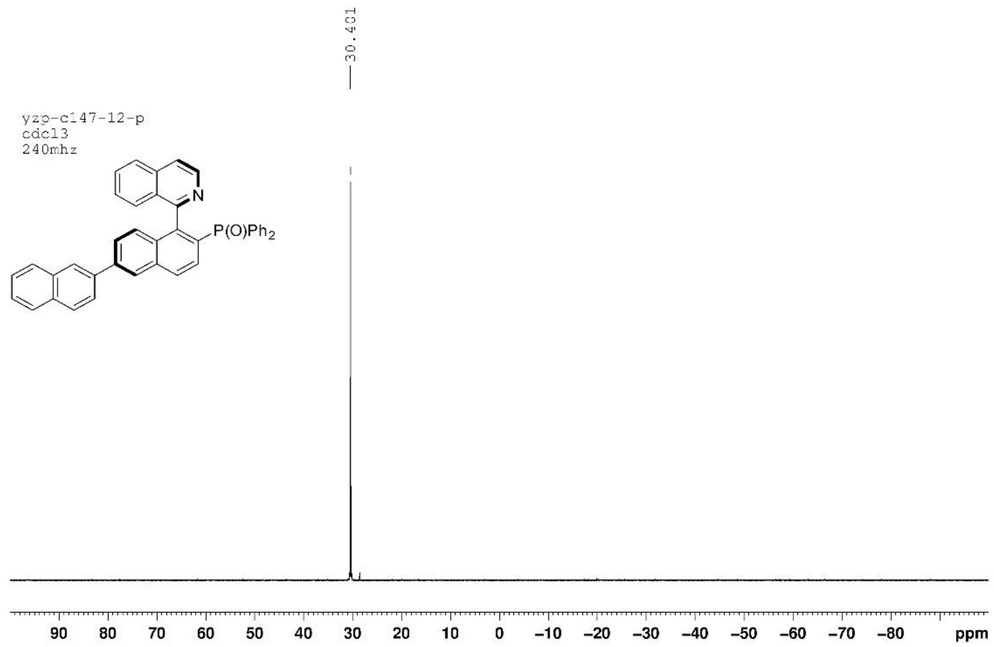

(*R*)-(1-(Isoquinolin-1-yl)-6-(thiophen-2-yl)naphthalen-2-yl)diphenylphosphine oxide (3ta)

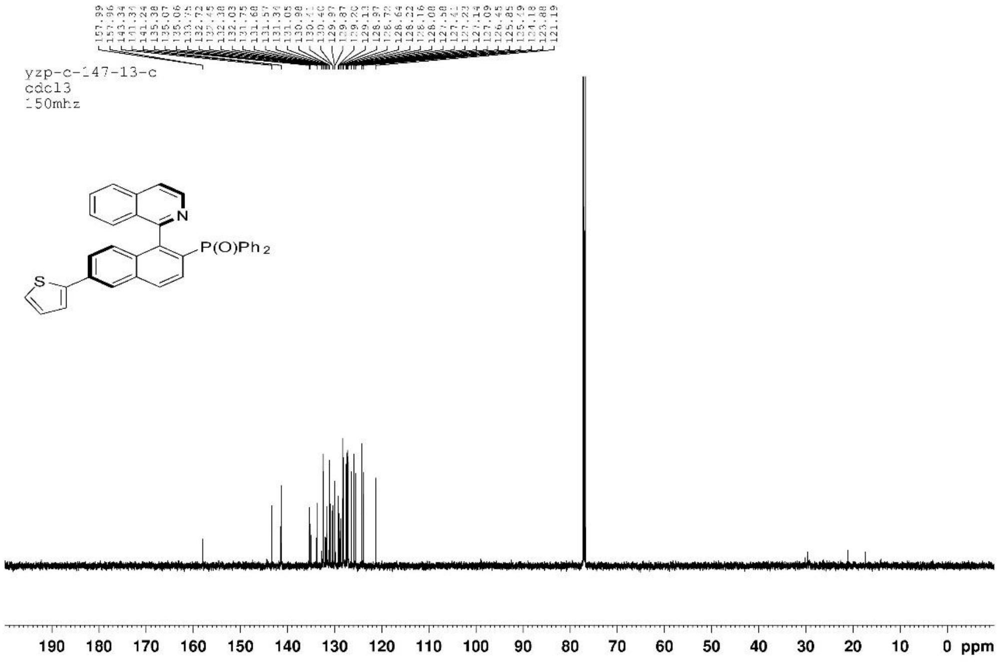

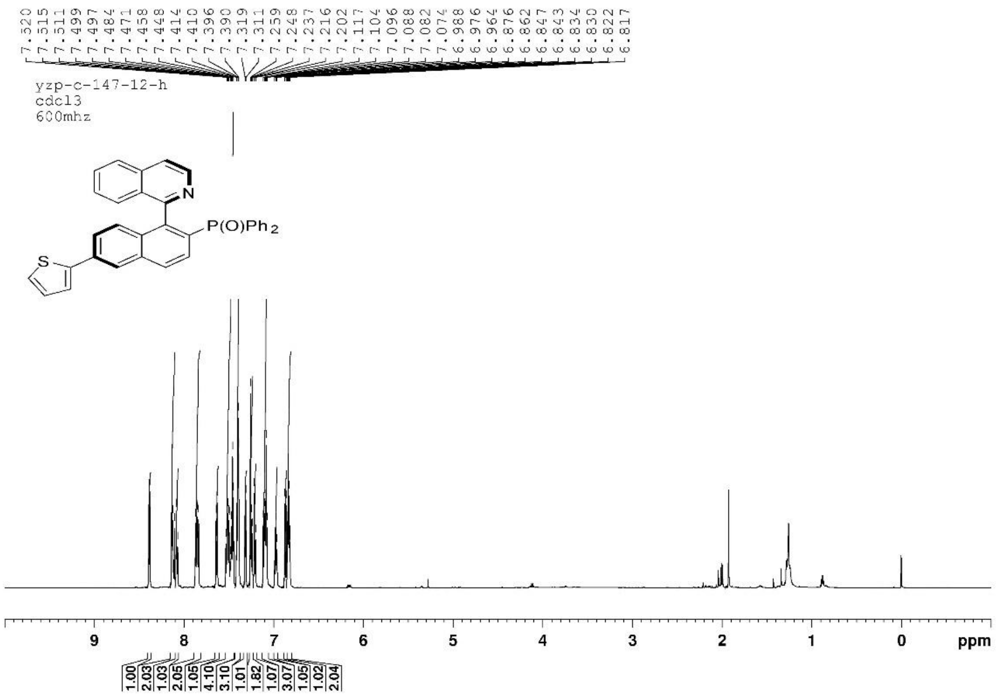

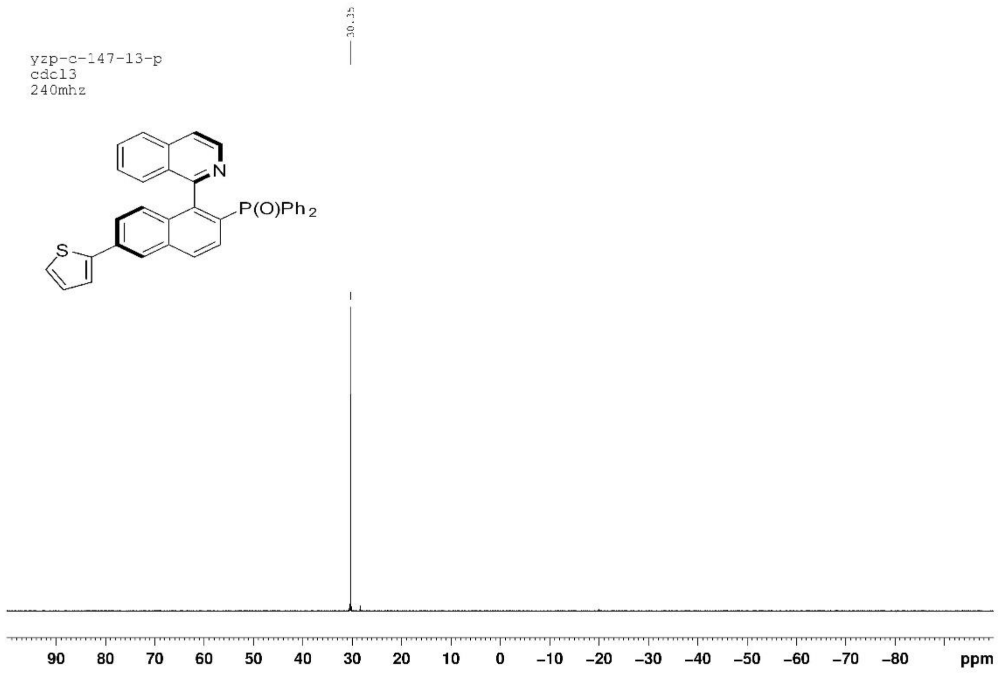

(*R*)-Diphenyl(1-(6-(p-tolyl)isoquinolin-1-yl)naphthalen-2-yl)phosphine oxide (3ua)

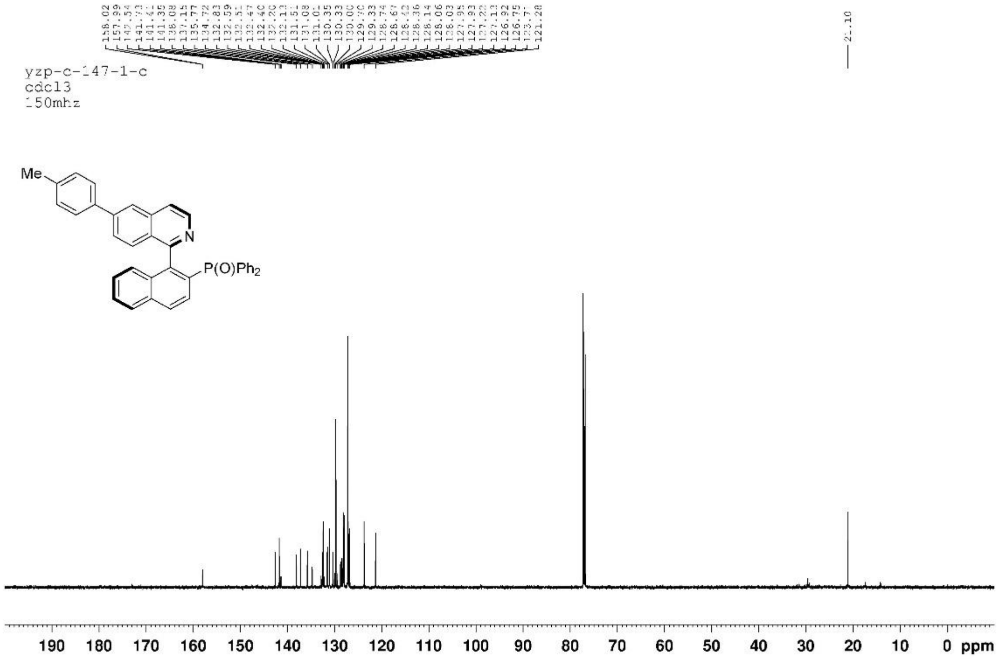

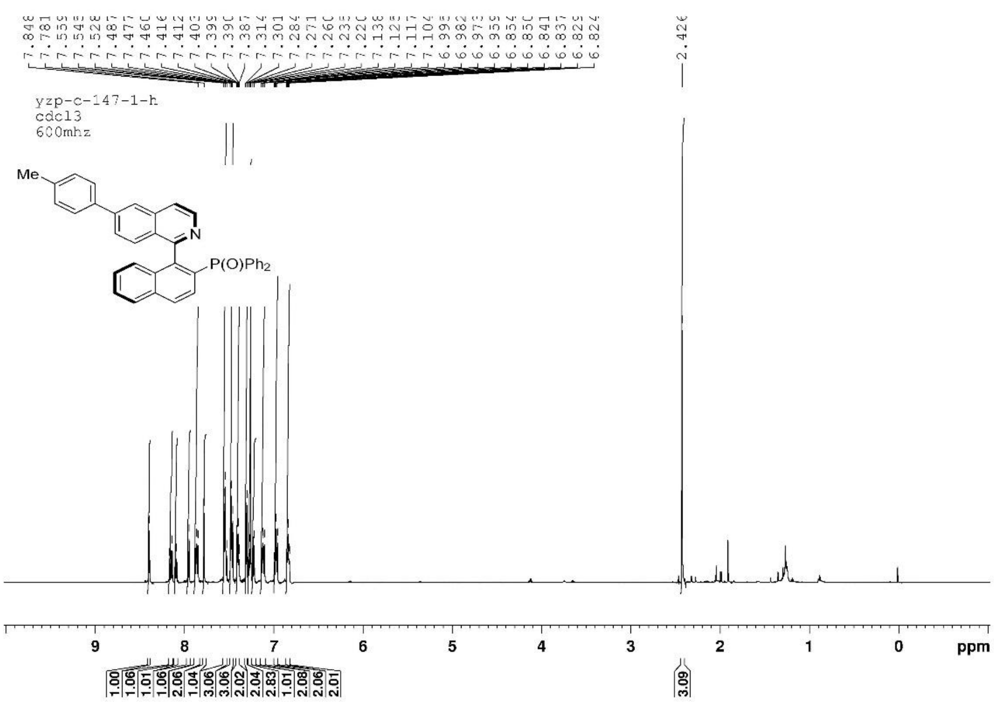

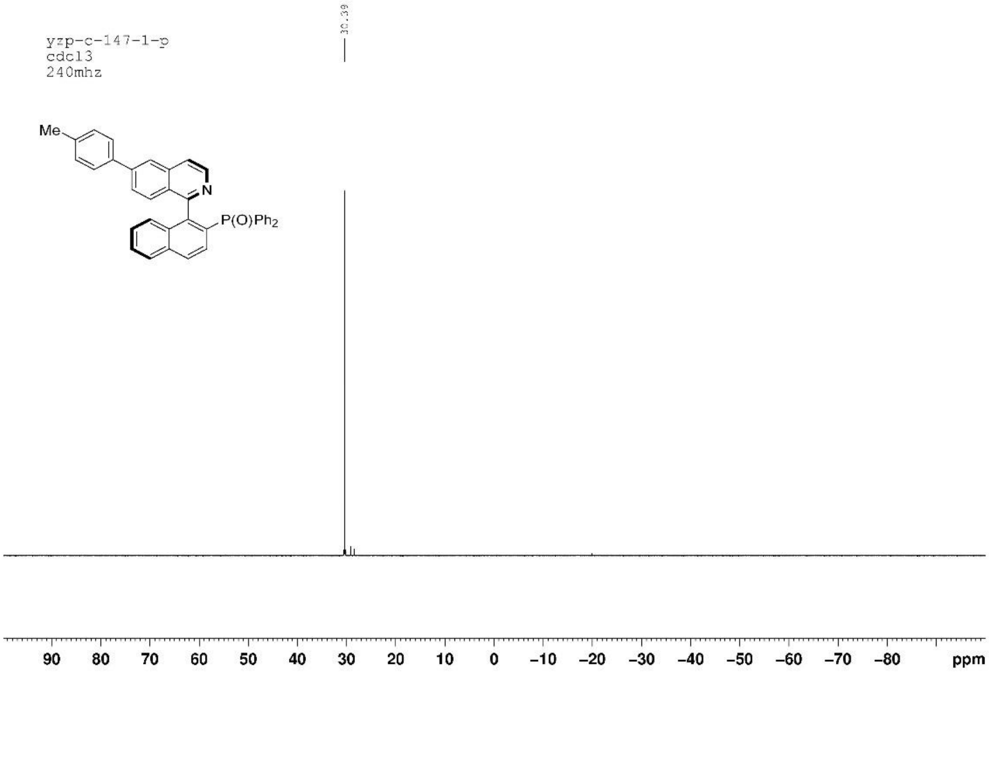

(*R*)-(1-(6-(3,5-Dimethylphenyl)isoquinolin-1-yl)naphthalen-2-yl)diphenylphosphine oxide (3va)

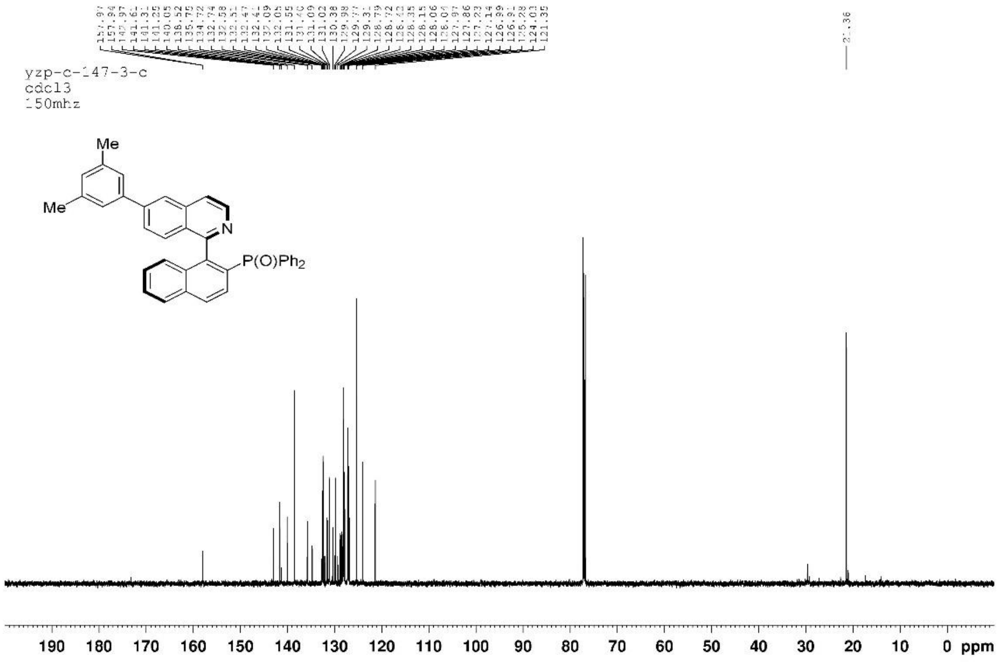

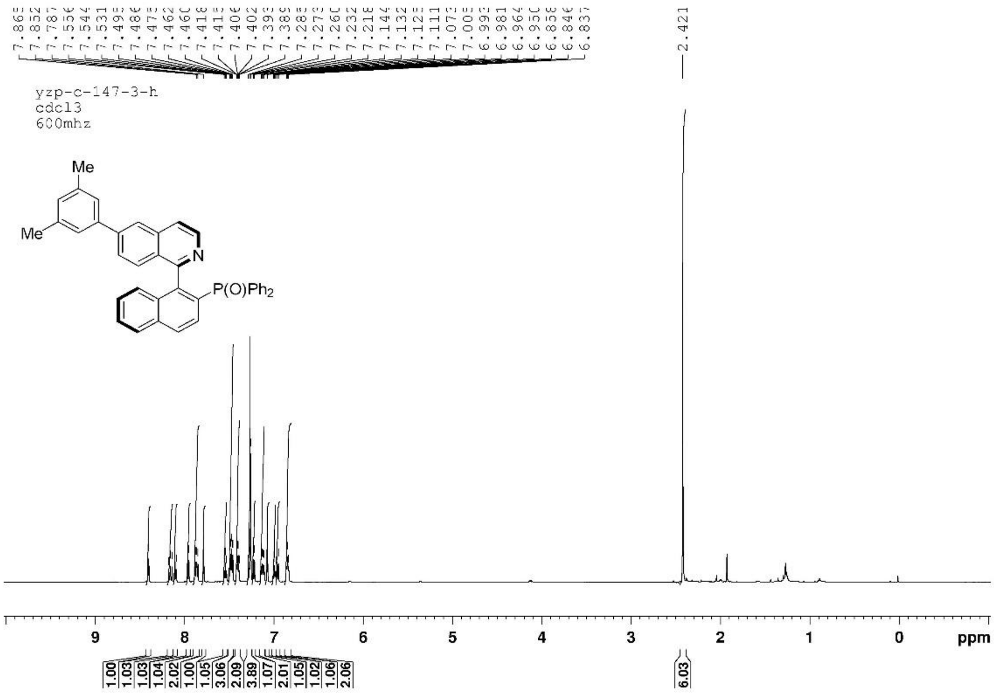

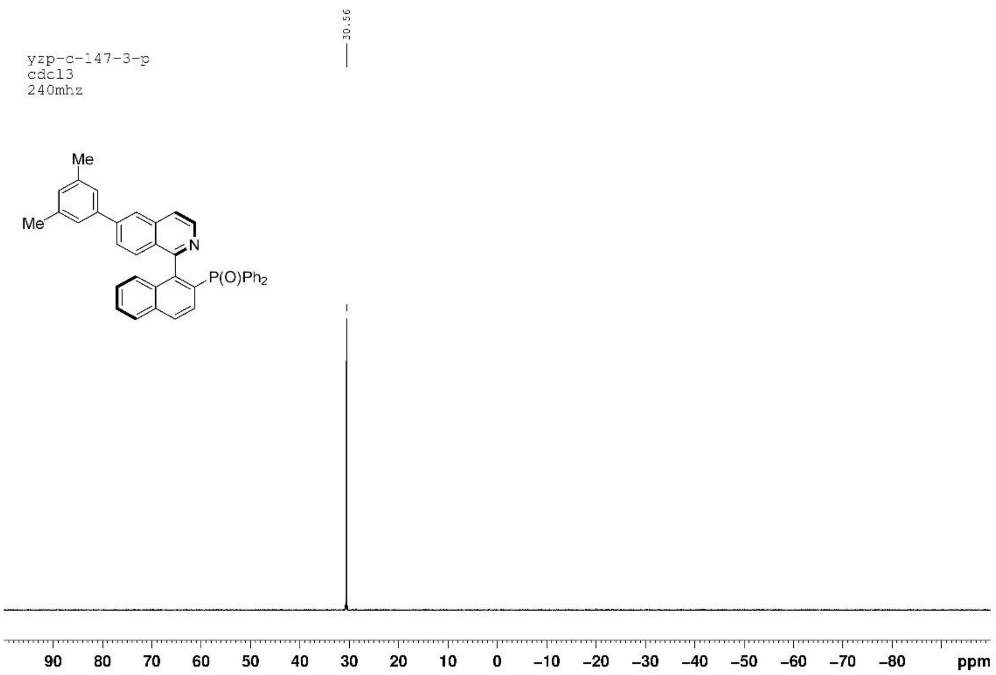

(*R*)-(1-(6-(4-Methoxyphenyl)isoquinolin-1-yl)naphthalen-2-yl)diphenylphosphine oxide (3wa)

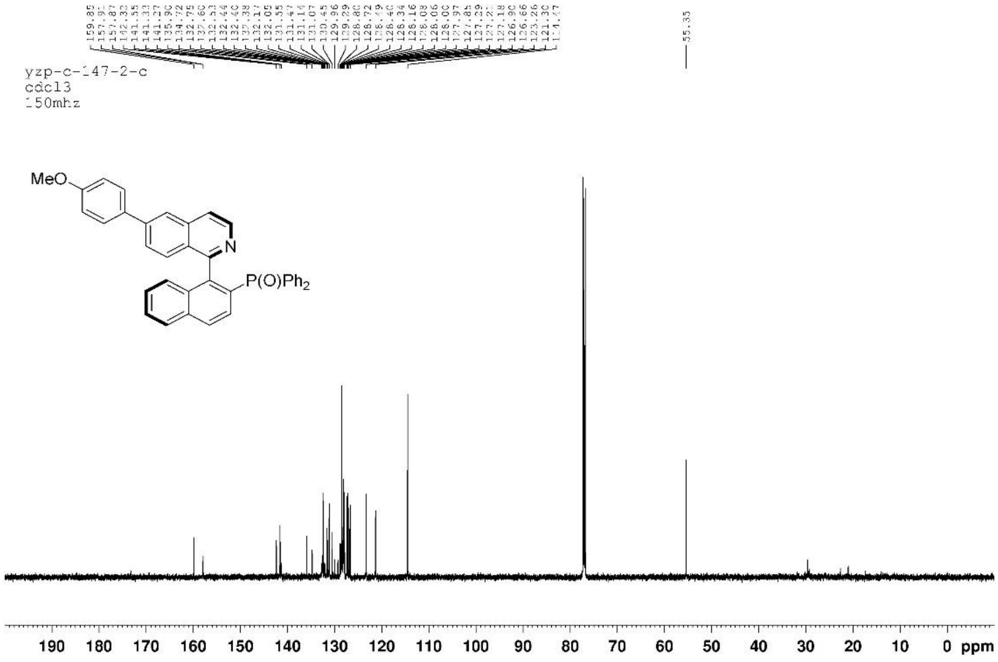

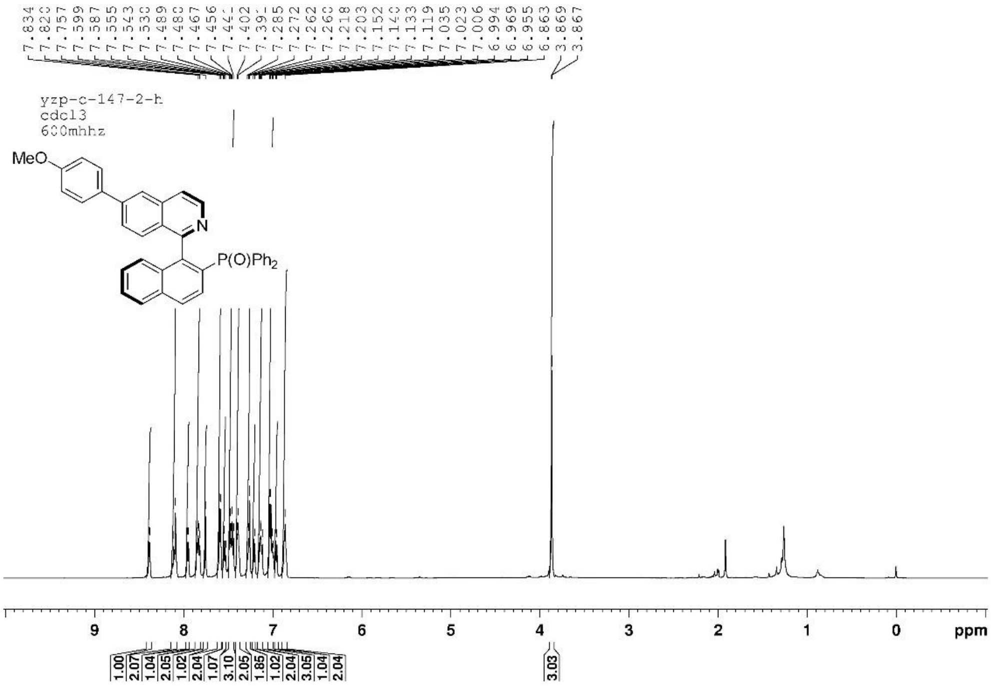

yzp-c-147-2-h
cdcl3
600mhhz
MeO
N
P(O)Ph2
ppm

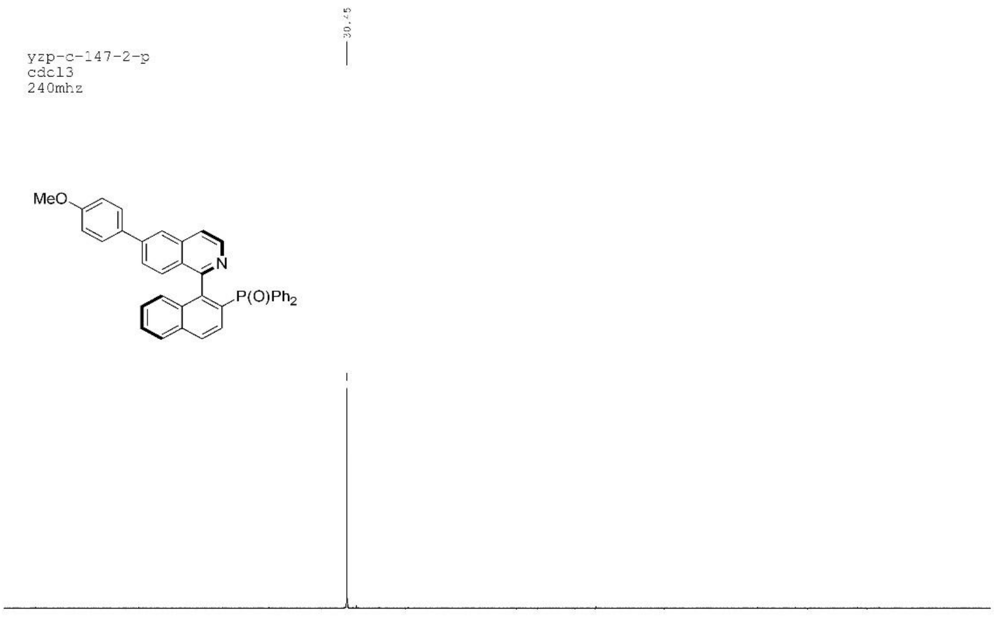

30.45
yzp-c-147-2-p
cdcl3
240mhz
MeO
N
P(O)Ph2
ppm

(*R*)-(1-(6-(4-Fluorophenyl)isoquinolin-1-yl)naphthalen-2-yl)diphenylphosphine oxide (3xa)

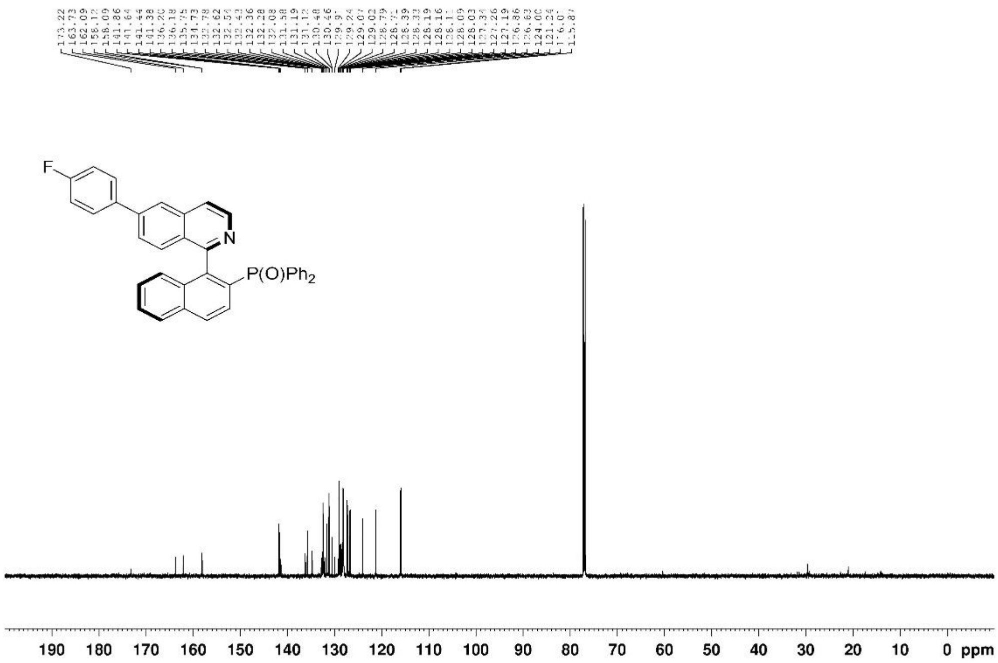

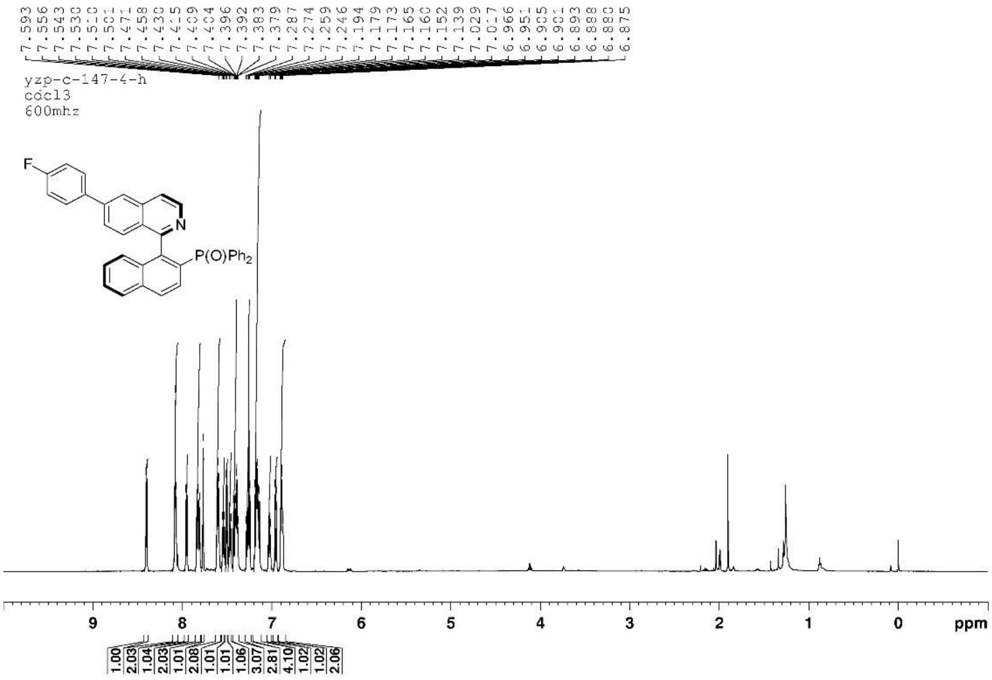

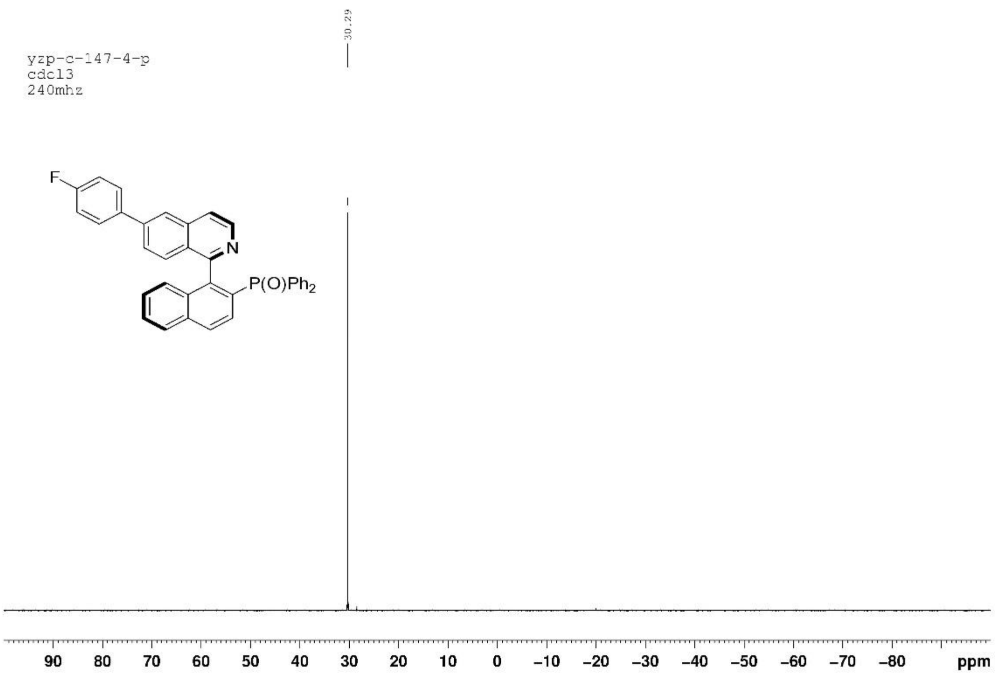

30.29
yzp-c-147-4-p
cdcl3
240mhz
F
N
P(O)Ph2
90 80 70 60 50 40 30 20 10 0 −10 −20 −30 −40 −50 −60 −70 −80 ppm

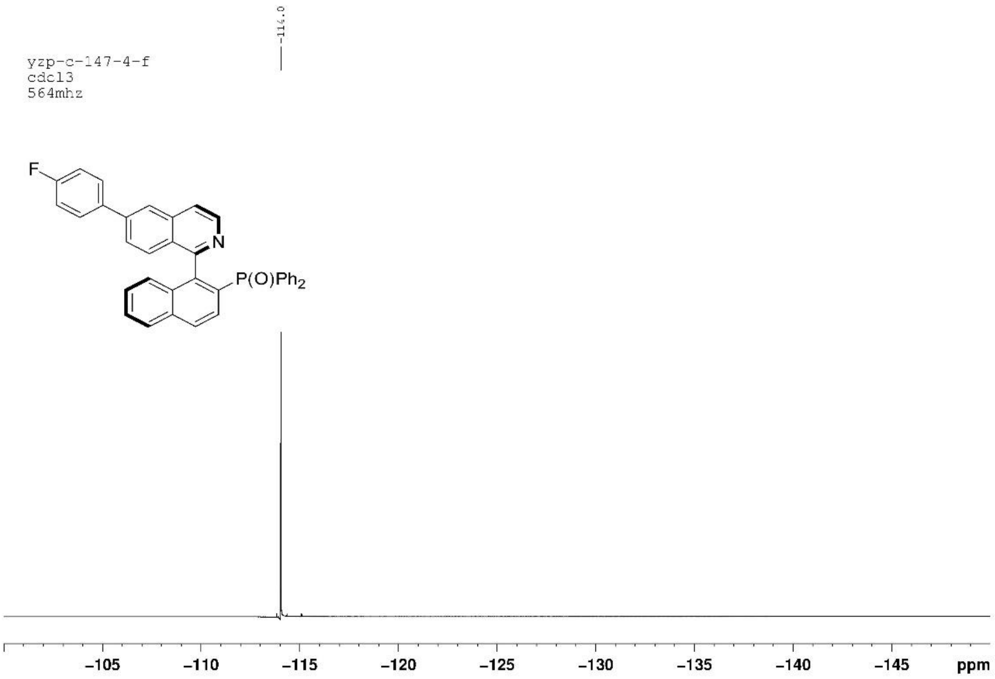

-114.0
yzp-c-147-4-f
cdcl3
564mhz
F
N
P(O)Ph2
−105 −110 −115 −120 −125 −130 −135 −140 −145 ppm

(*R*)-Diphenyl(1-(6-(thiophen-2-yl)isoquinolin-1-yl)naphthalen-2-yl)phosphine oxide (3ya)

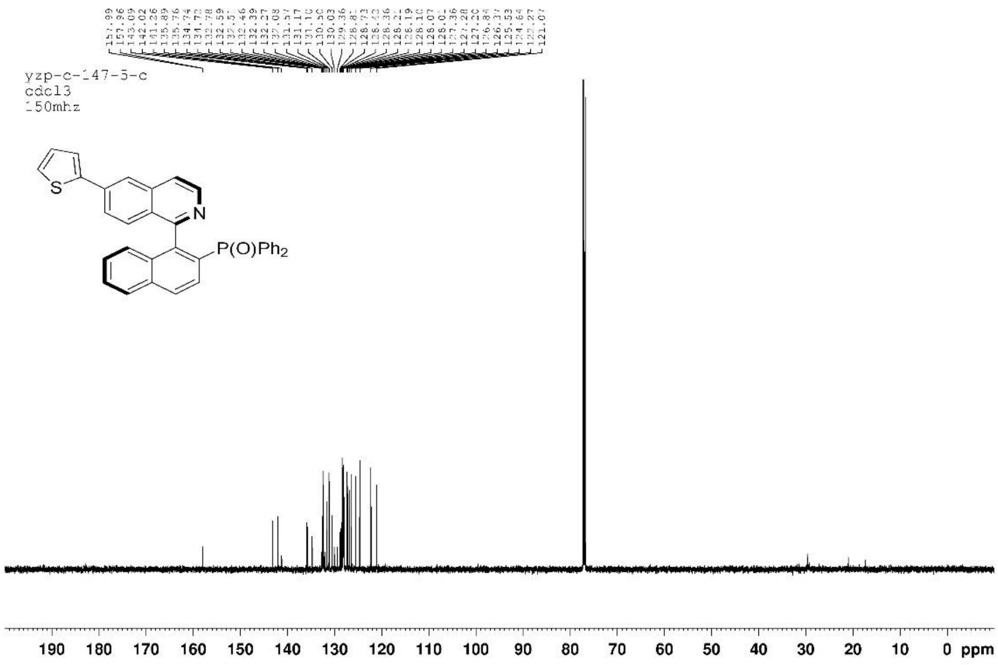

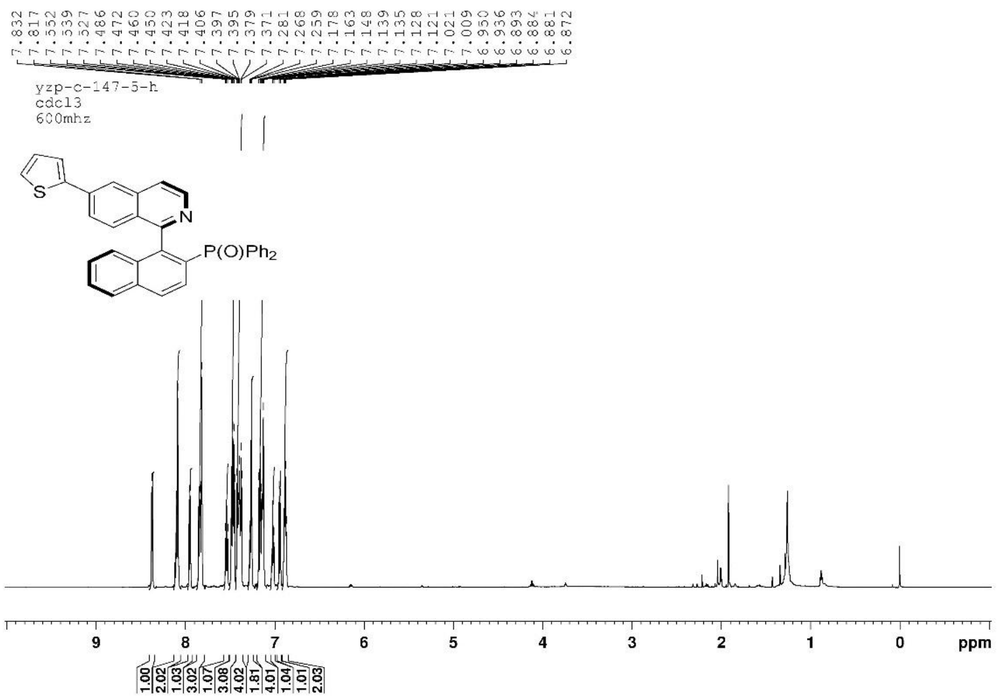

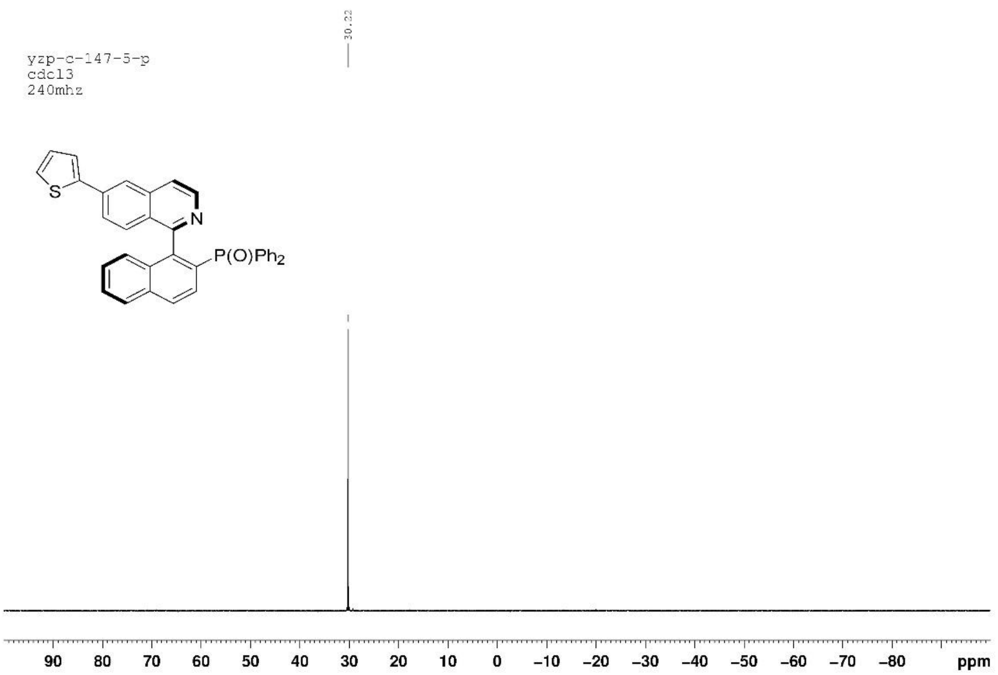

(*R*)-Diphenyl(6-phenyl-1-(6-phenylisoquinolin-1-yl)naphthalen-2-yl)phosphine oxide (3za)

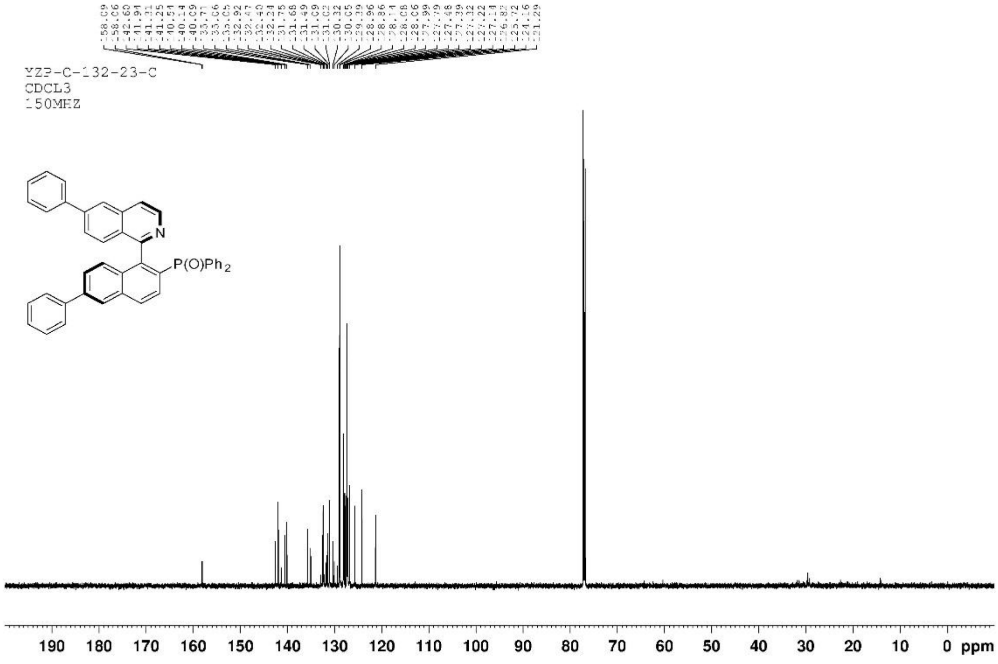

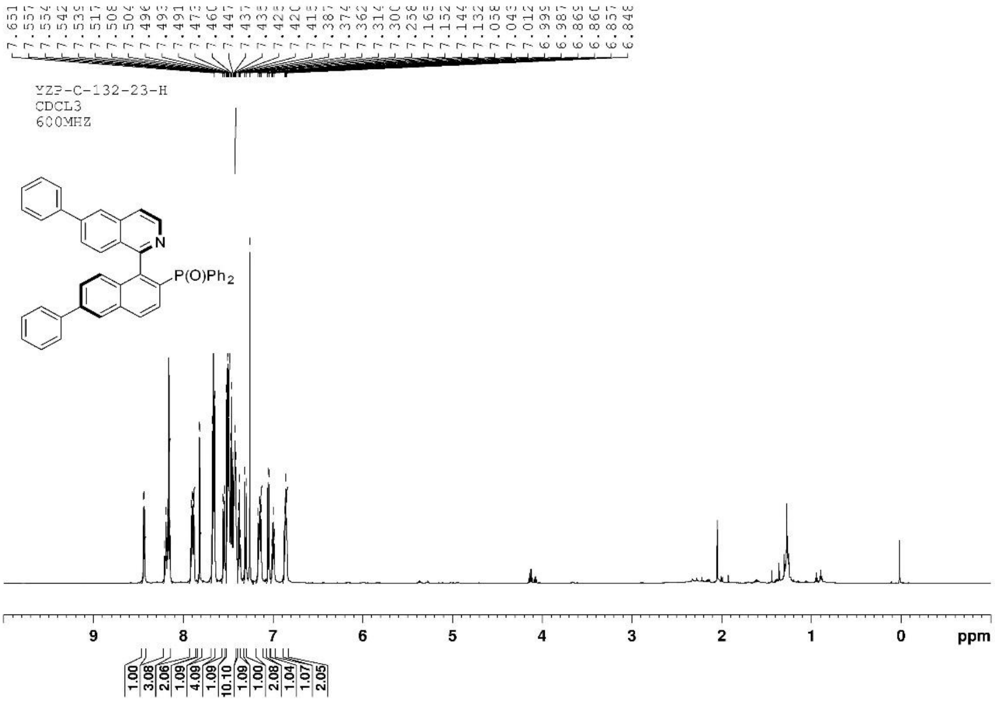
YZP-C-132-23-H
CDCL3
600MHZ
P(O)Ph2
N
9
8
7
6
5
4
3
2
1
0
ppm

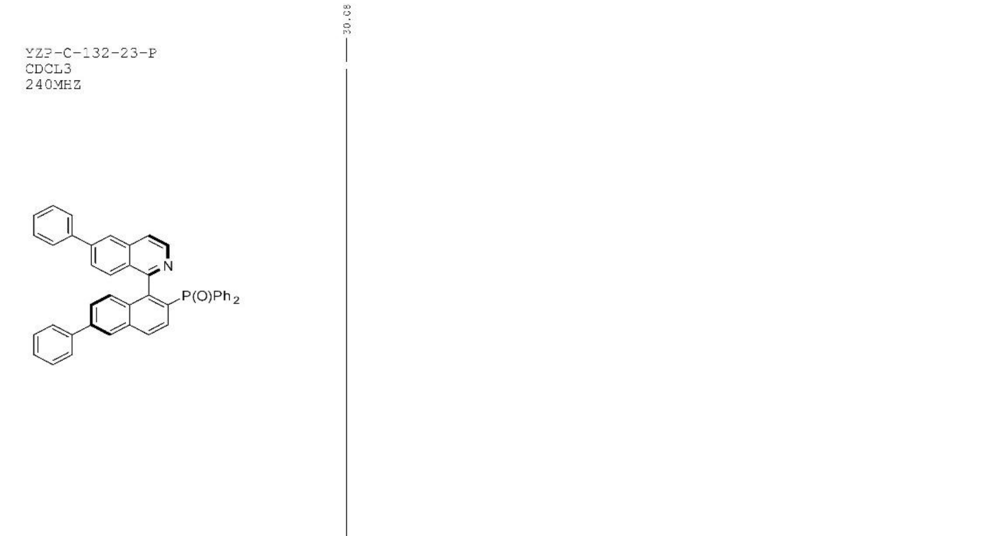
30.08
YZP-C-132-23-P
CDCL3
240MHZ
P(O)Ph2
N
90
80
70
60
50
40
30
20
10
0
−10
−20
−30
−40
−50
−60
−70
−80
ppm

(*R*)-Diphenyl(7-phenyl-1-(7-phenylisoquinolin-1-yl)naphthalen-2-yl)phosphine oxide (3aaa)

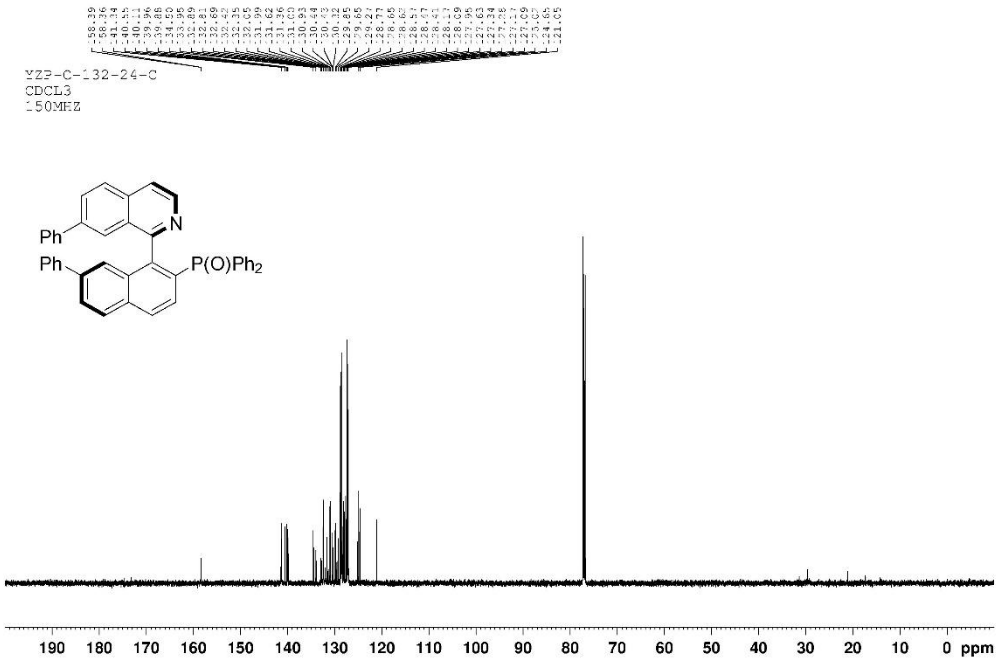

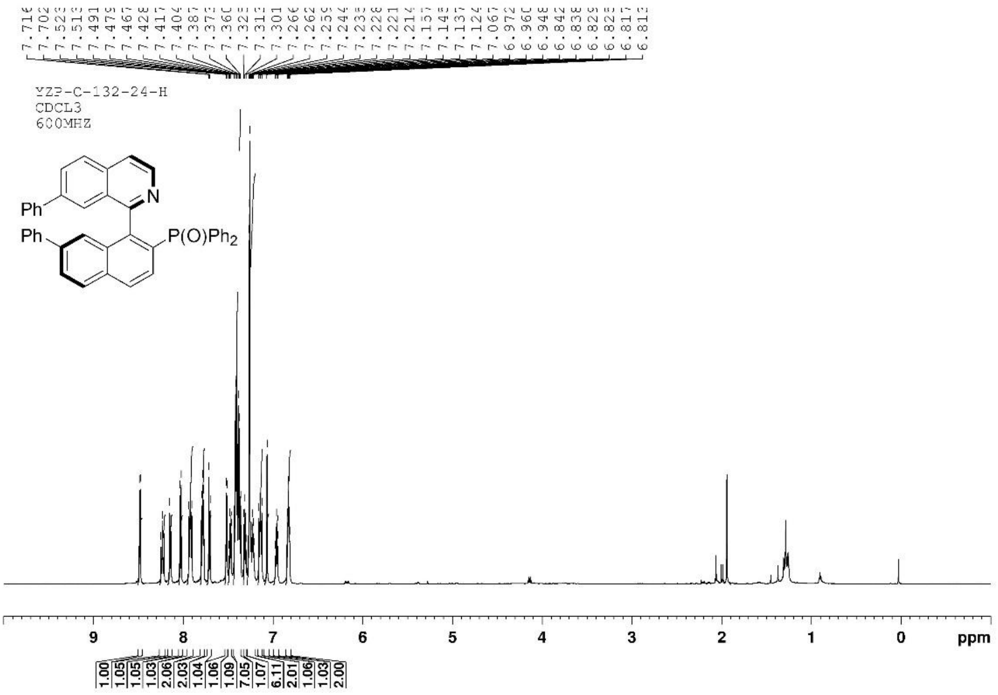

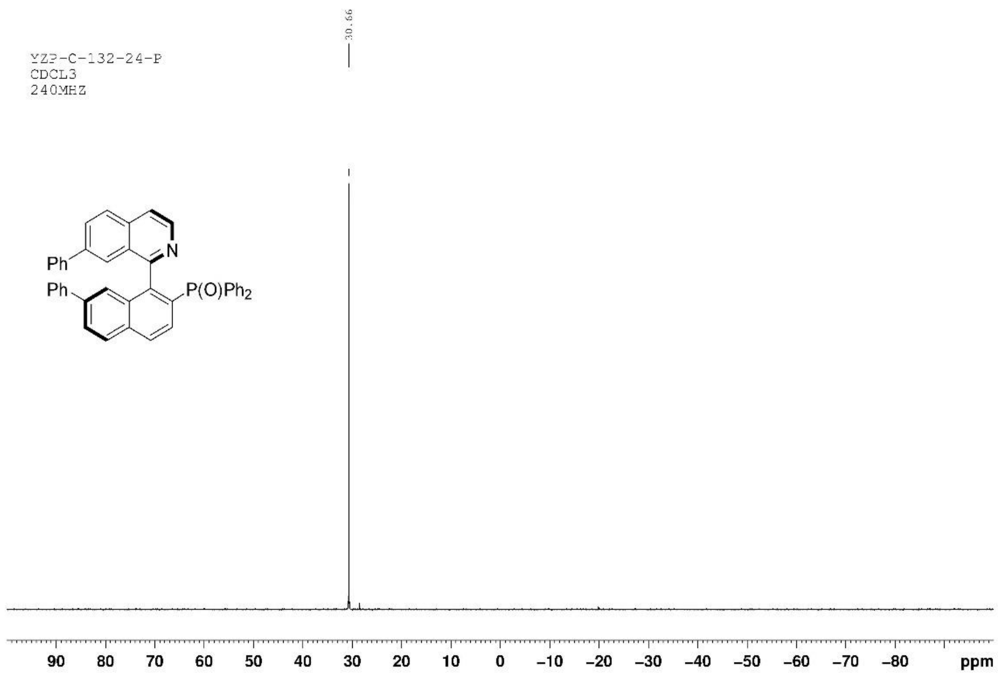

(*R*)-diphenyl(7-phenyl-1-(6-phenylisoquinolin-1-yl)naphthalen-2-yl)phosphine oxide (3aba)

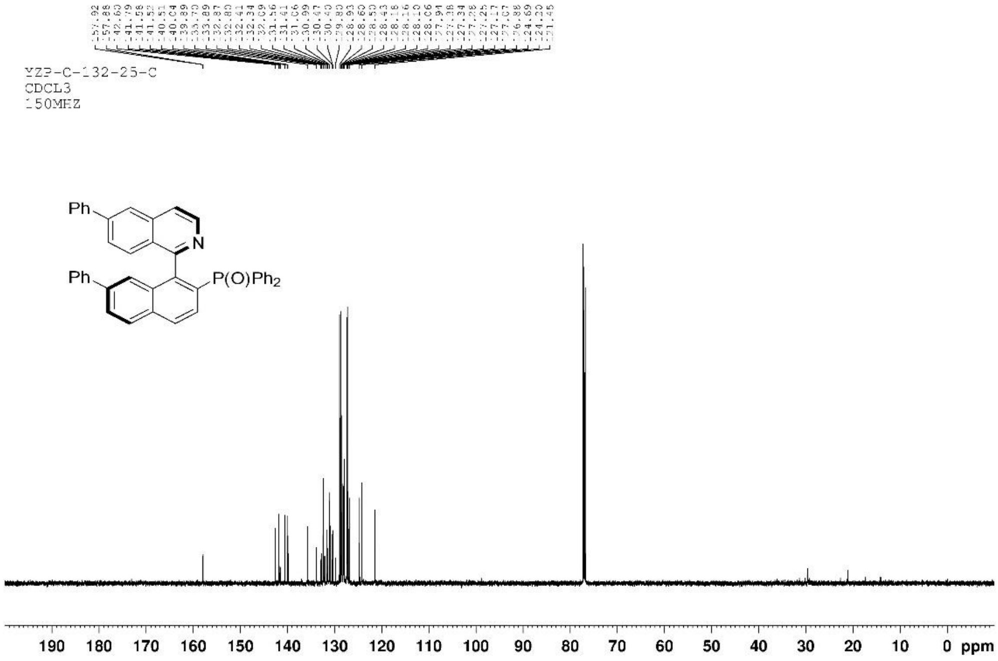

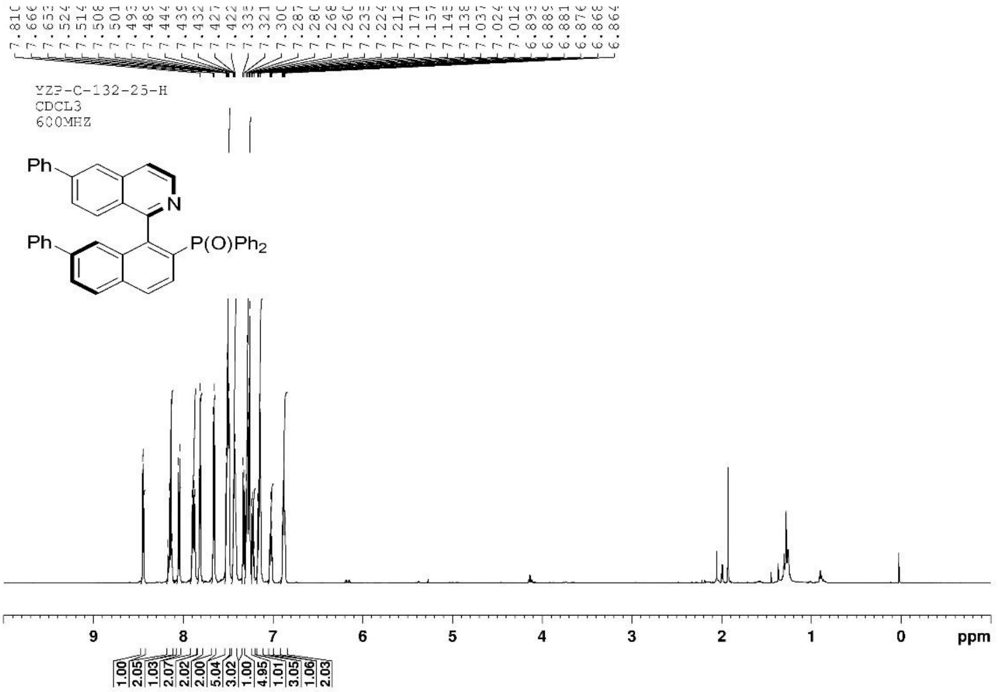
YZP-C-132-25-H
CDCL3
600MHZ
Ph
N
Ph
P(O)Ph2
9
8
7
6
5
4
3
2
1
0
ppm
1.00
2.05
1.03
2.07
2.02
2.00
5.04
3.02
1.00
4.95
1.01
3.05
1.06
2.03

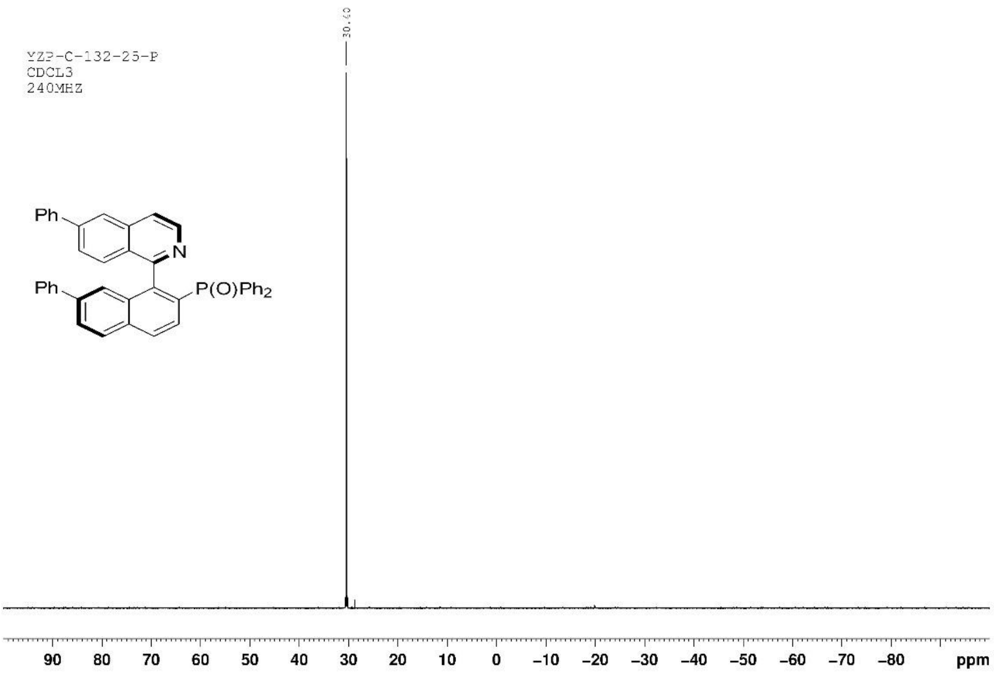
30.40
YZP-C-132-25-P
CDCL3
240MHZ
Ph
N
Ph
P(O)Ph2
90
80
70
60
50
40
30
20
10
0
−10
−20
−30
−40
−50
−60
−70
−80
ppm

(*R*)-Diphenyl(6-phenyl-1-(7-phenyl-8,8a-dihydroisoquinolin-1-yl)naphthalen-2-yl)phosphine oxide (3aca)

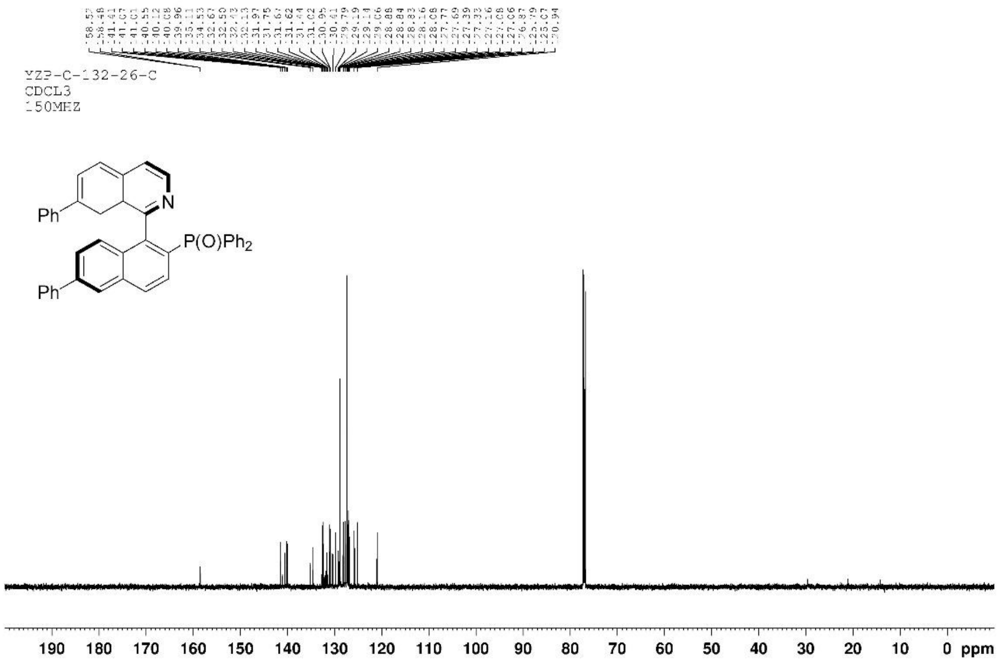

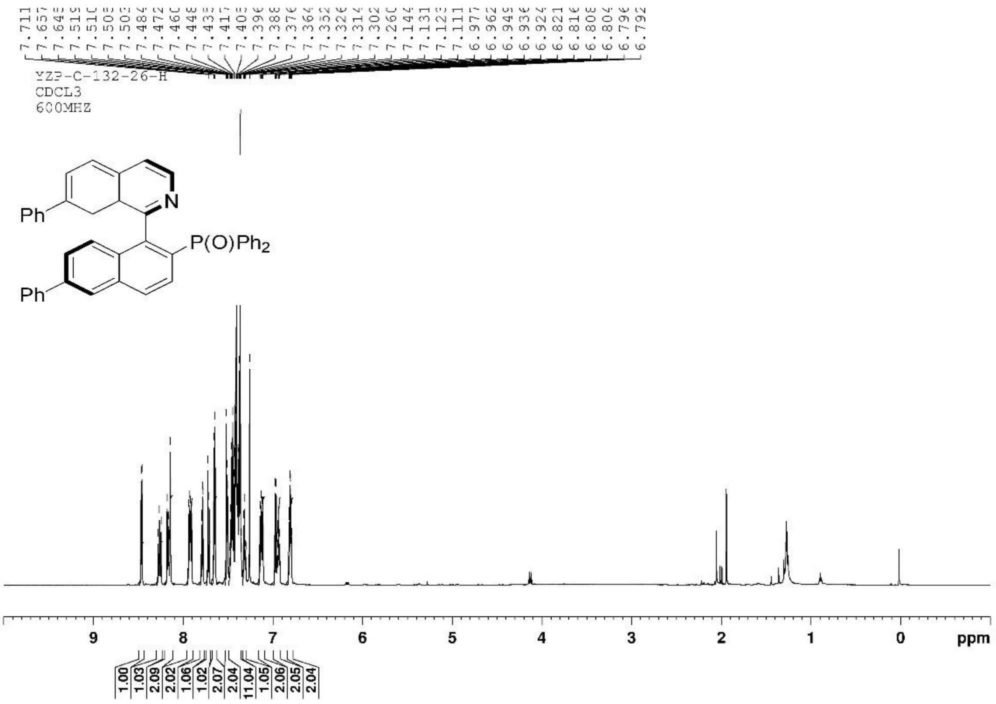

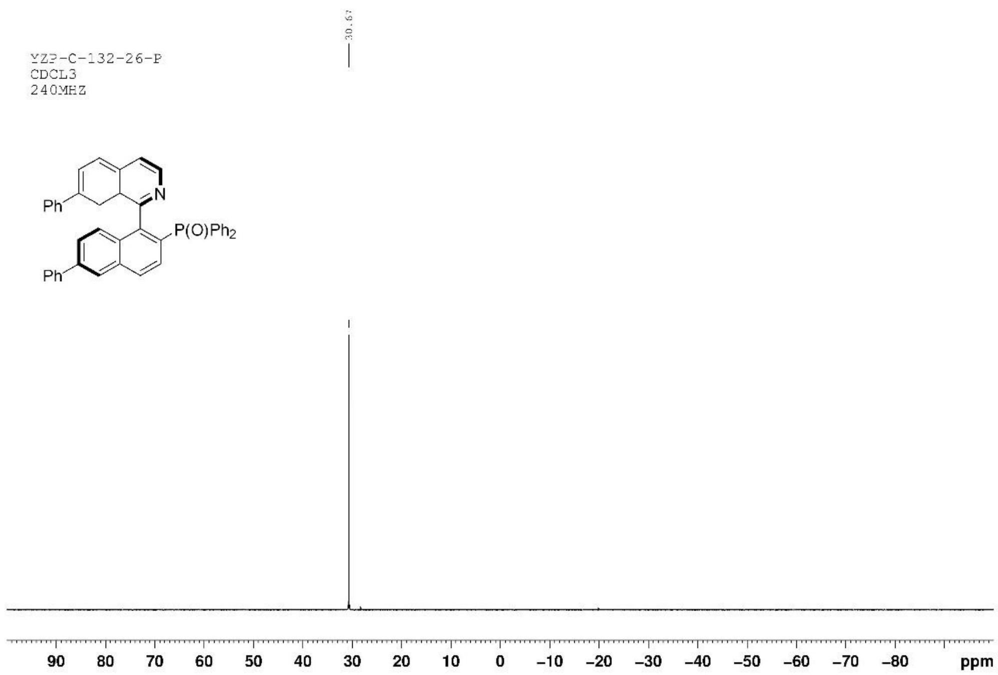

(*R*)-(6-Methoxy-1-(6-phenylisoquinolin-1-yl)naphthalen-2-yl)diphenylphosphine oxide (3ada)

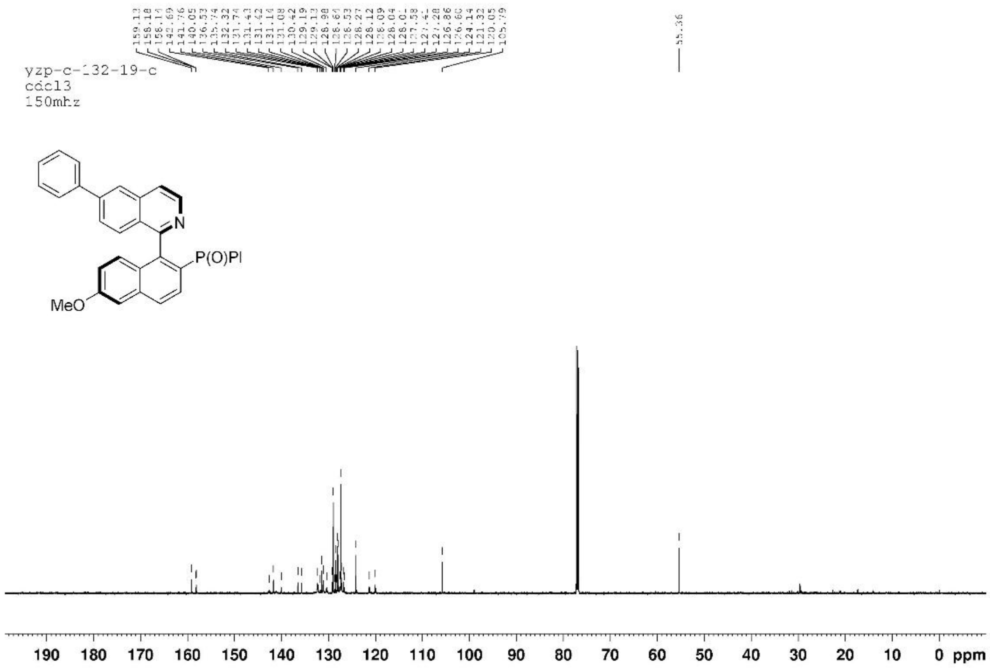

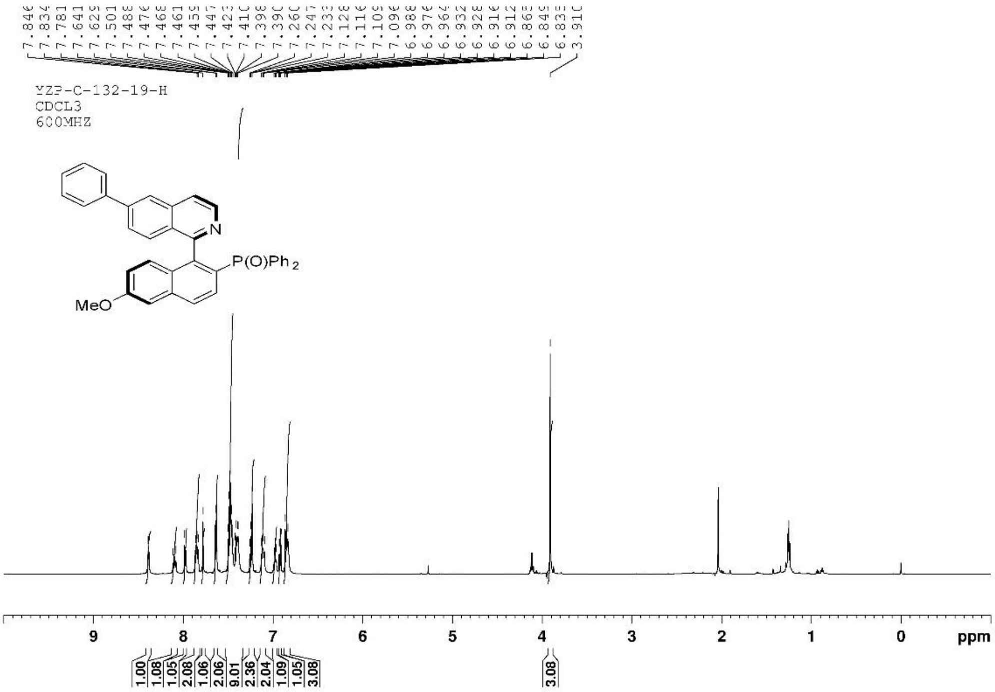
YZP-C-132-19-H
CDCL3
600MHZ
P(O)Ph2
N
MeO
9
8
7
6
5
4
3
2
1
0
ppm
1.00
1.08
1.05
2.08
1.06
2.06
9.01
2.36
2.04
1.09
1.05
3.08
3.08

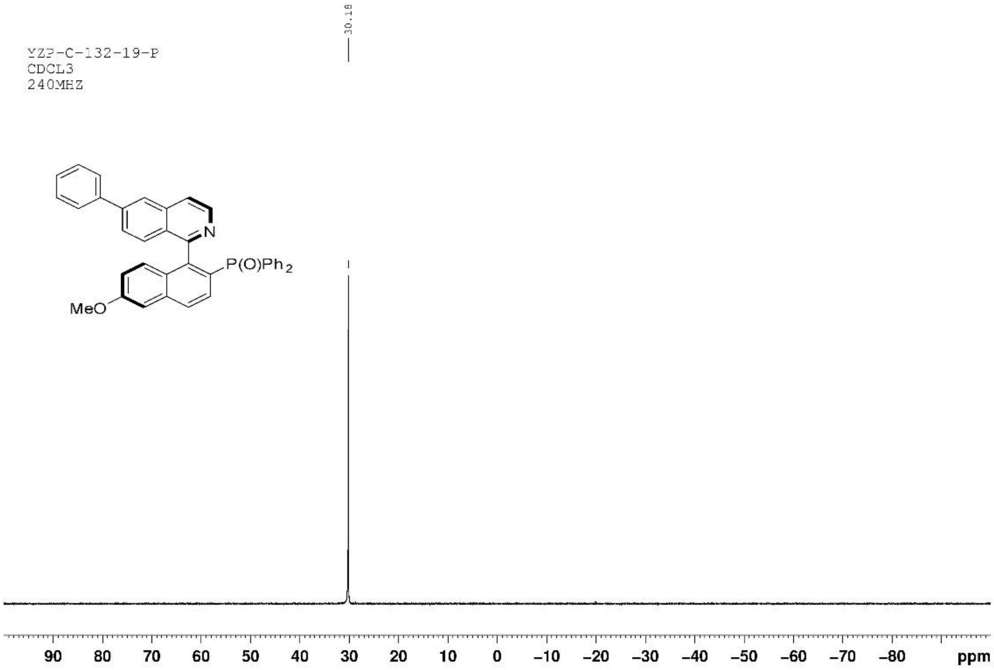
30.18
YZP-C-132-19-P
CDCL3
240MHZ
P(O)Ph2
N
MeO
90
80
70
60
50
40
30
20
10
0
–10
–20
–30
–40
–50
–60
–70
–80
ppm

(*R*)-(7-Methoxy-1-(7-phenylisoquinolin-1-yl)naphthalen-2-yl)diphenylphosphine oxide (3aea)

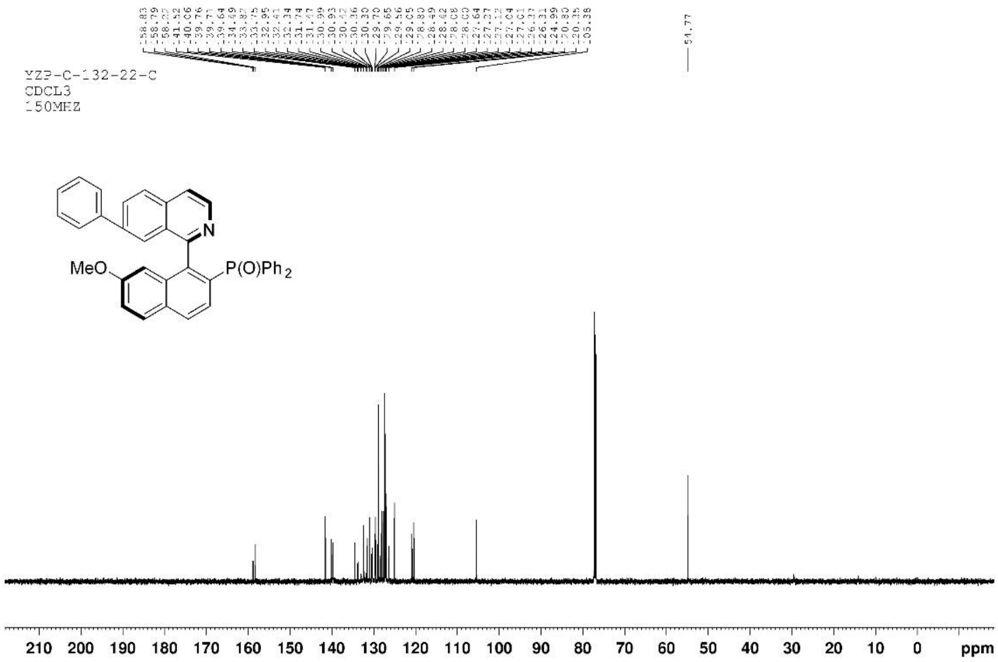

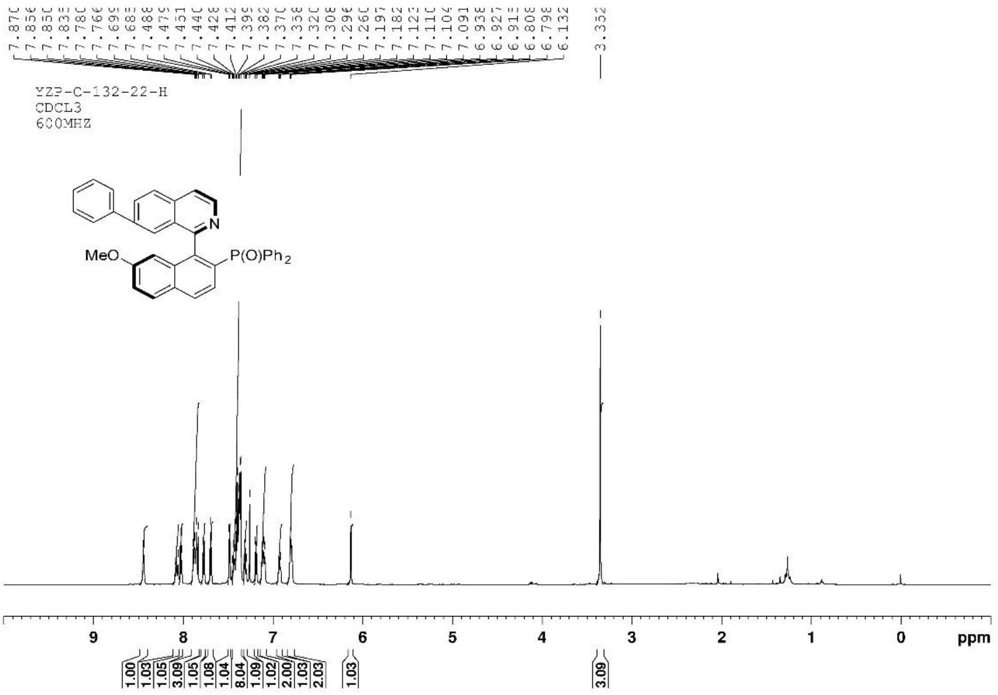

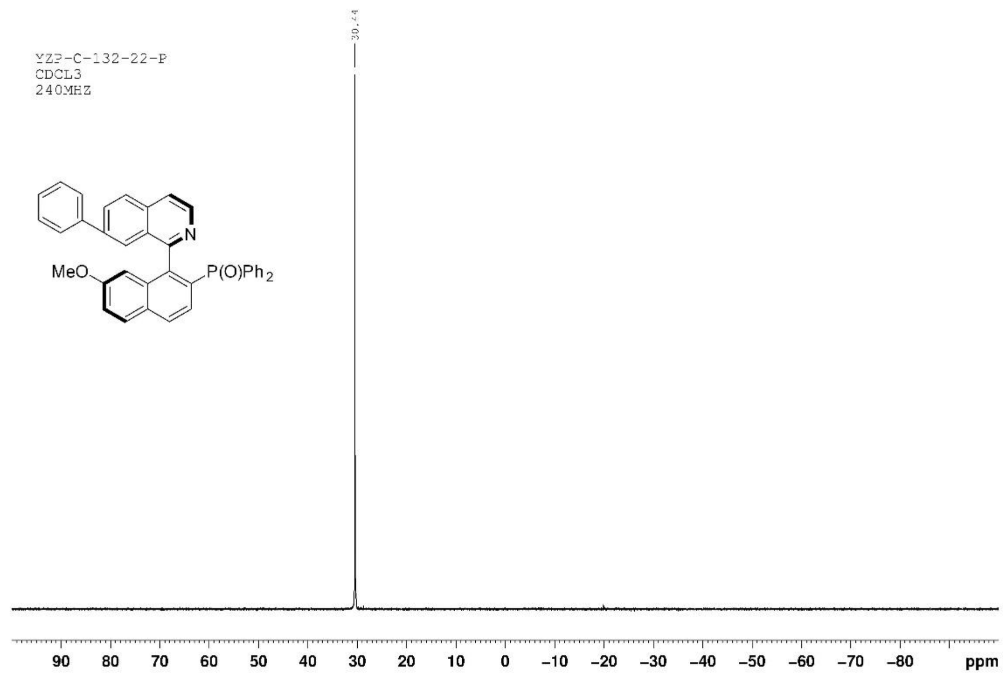

(*R*)-(7-Methoxy-1-(6-phenylisoquinolin-1-yl)naphthalen-2-yl)diphenylphosphine oxide (3afa)

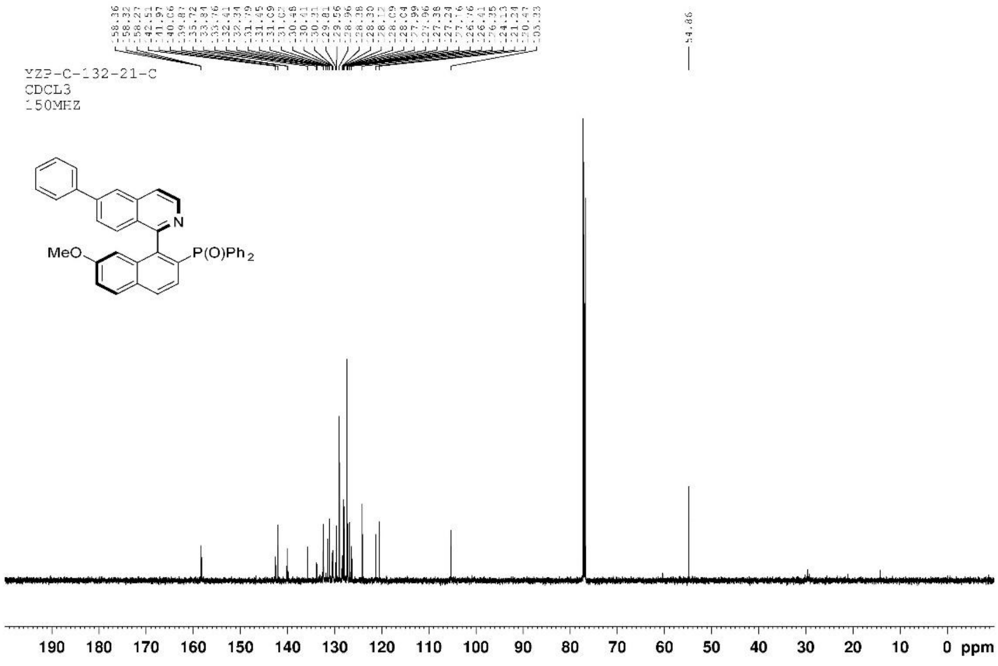

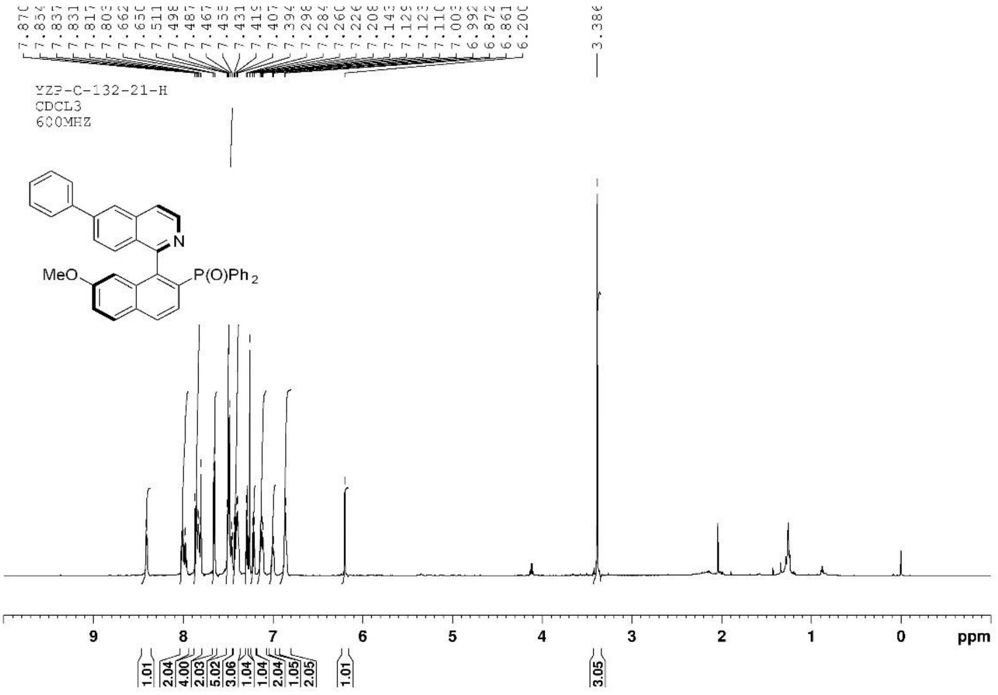
YZP-C-132-21-H
CDCL3
600MHZ
MeO
P(O)Ph2
N
3.386
ppm

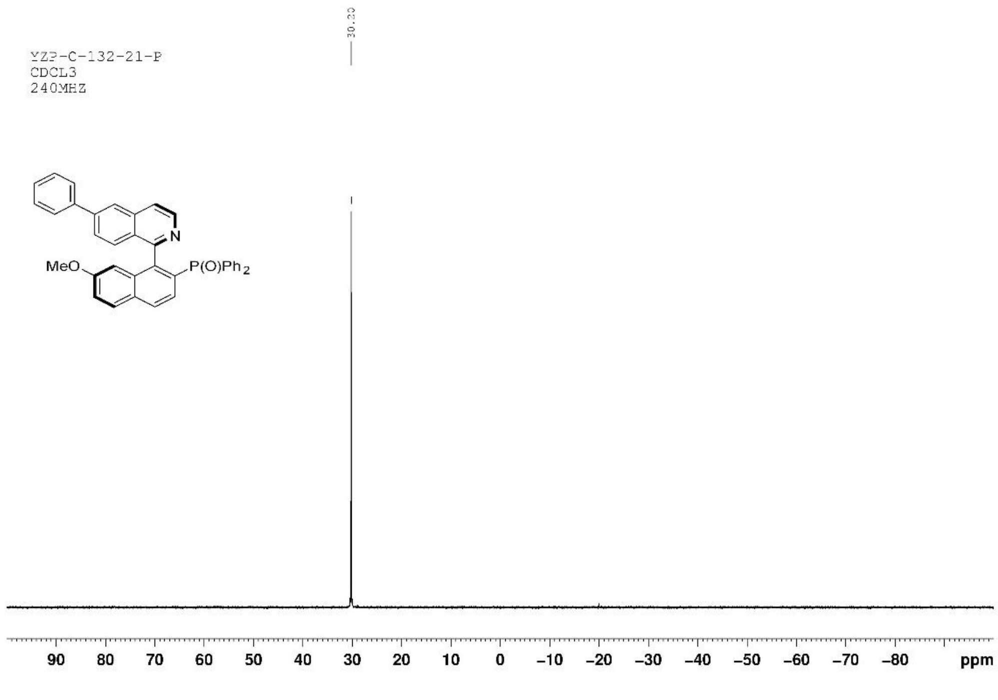
YZP-C-132-21-P
CDCL3
240MHZ
MeO
P(O)Ph2
N
ppm

(*R*)-(6-Methoxy-1-(7-phenylisoquinolin-1-yl)naphthalen-2-yl)diphenylphosphine oxide (3aga)

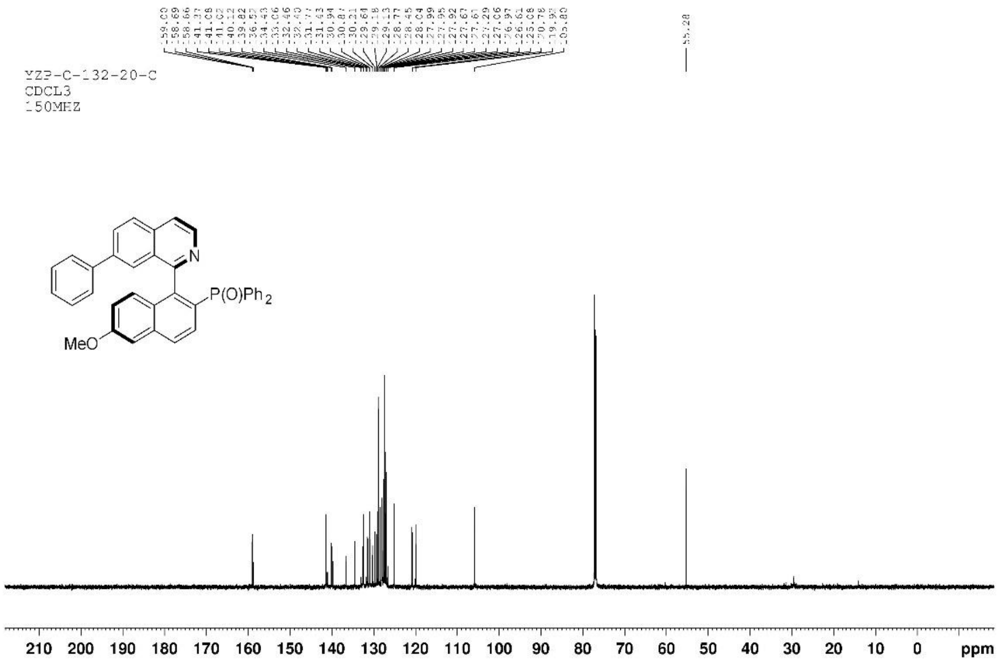

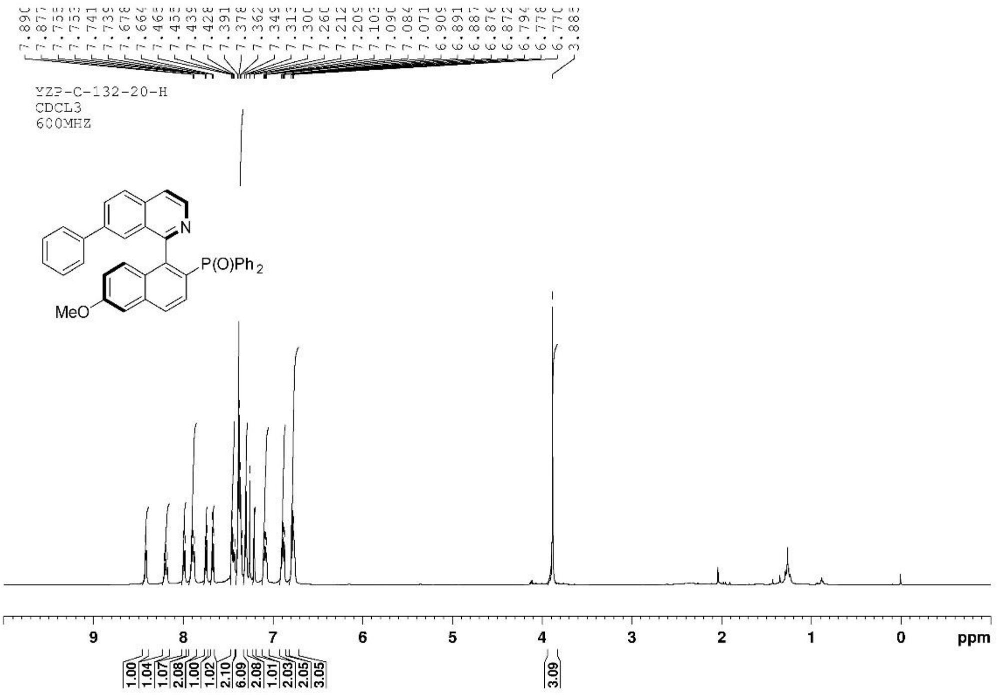

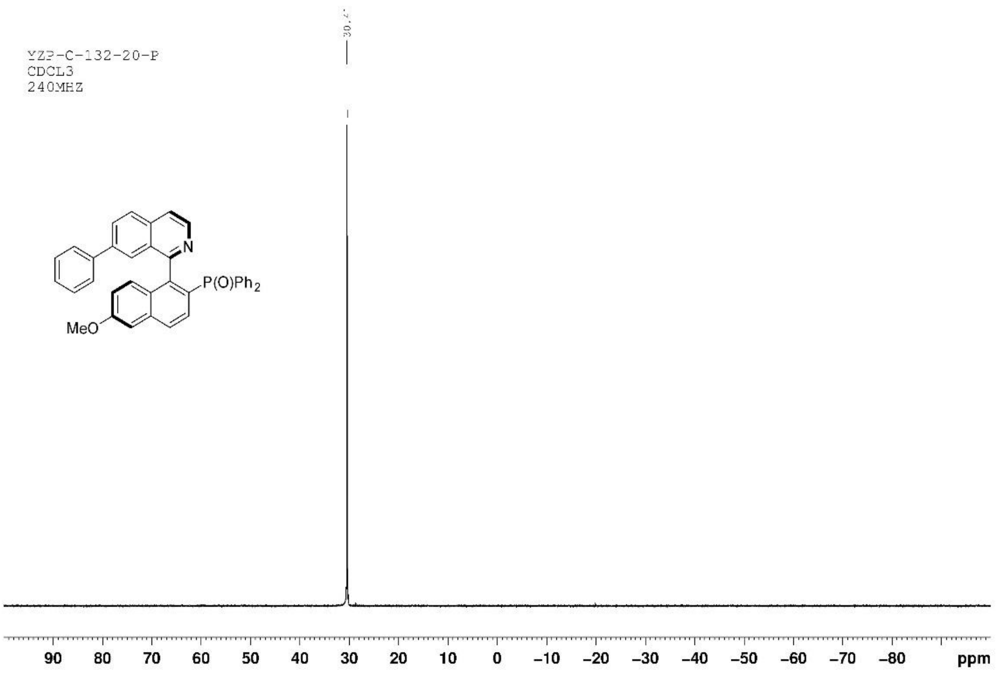

(*R*)-Diphenyl(1-(quinazolin-4-yl)naphthalen-2-yl)phosphine oxide (3aha)

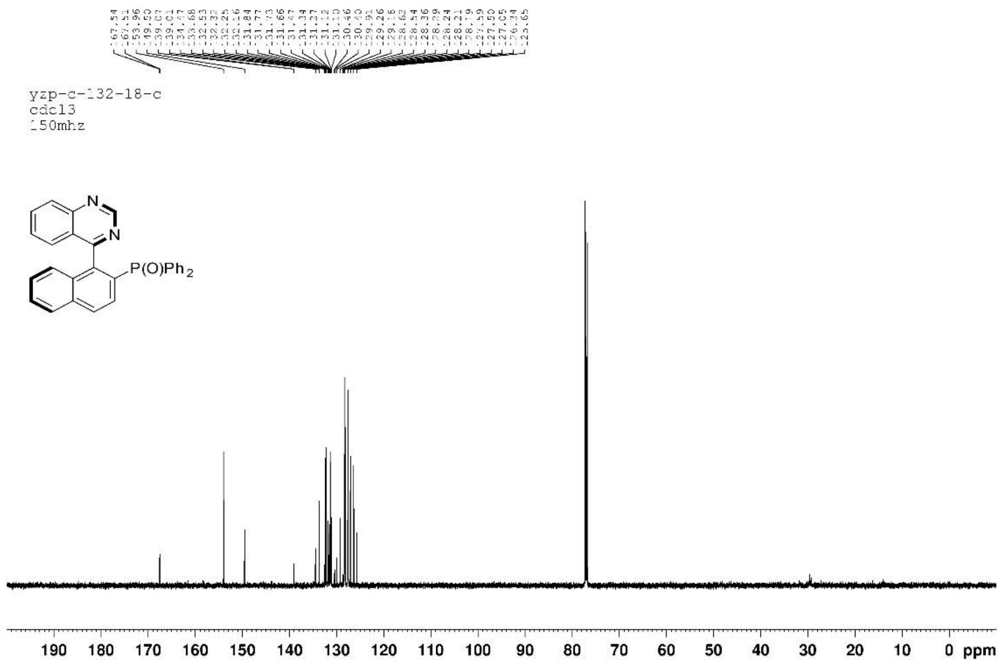

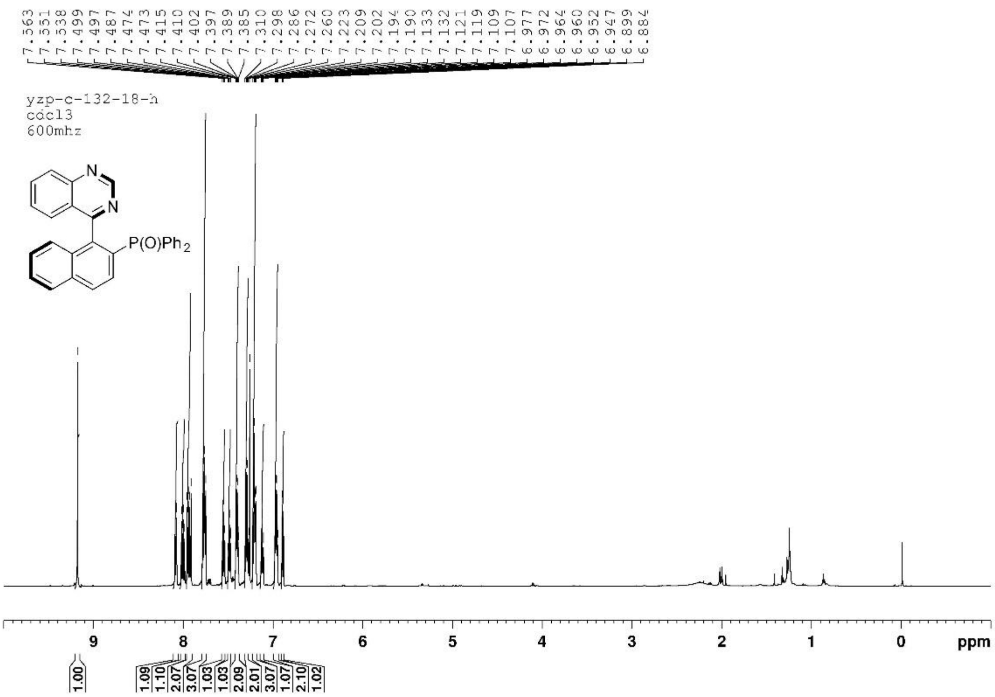

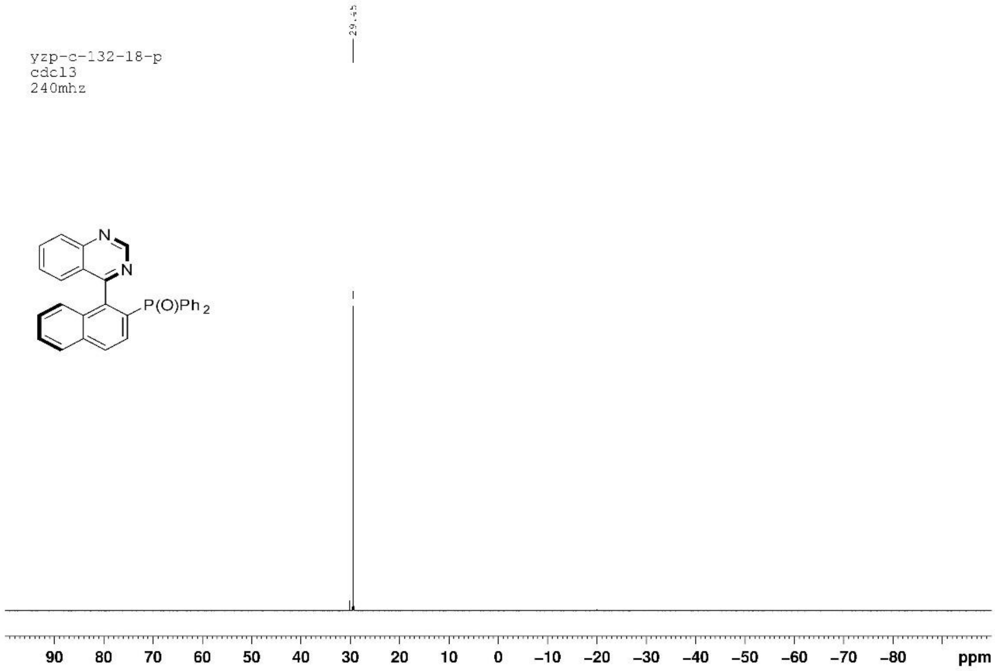

(*R*)- (1-(Isoquinolin-1-yl)naphthalen-2-yl)di-p-tolylphosphine oxide (3ab)

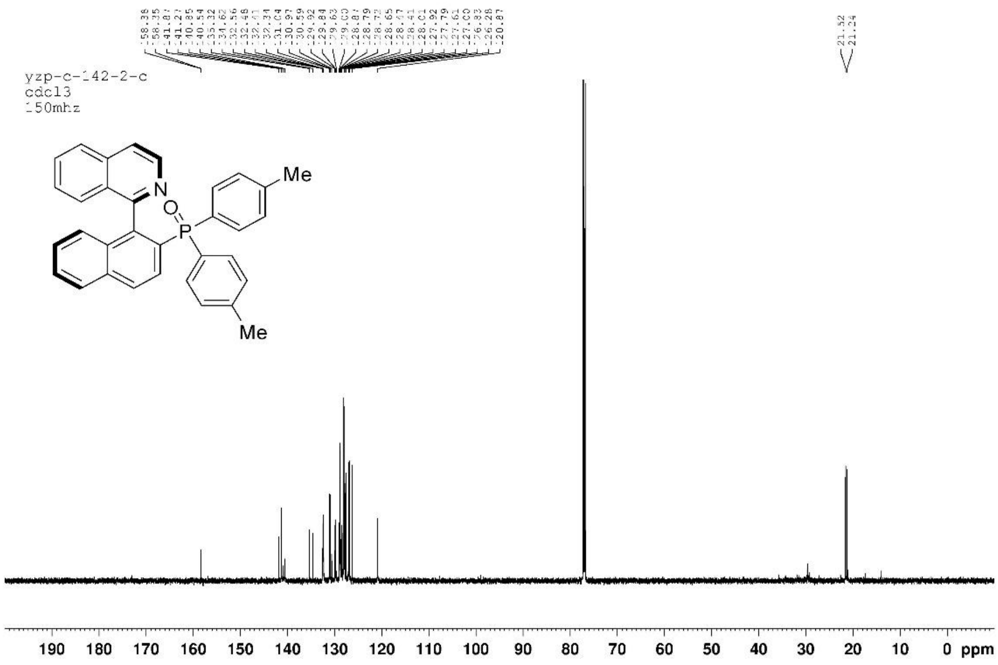

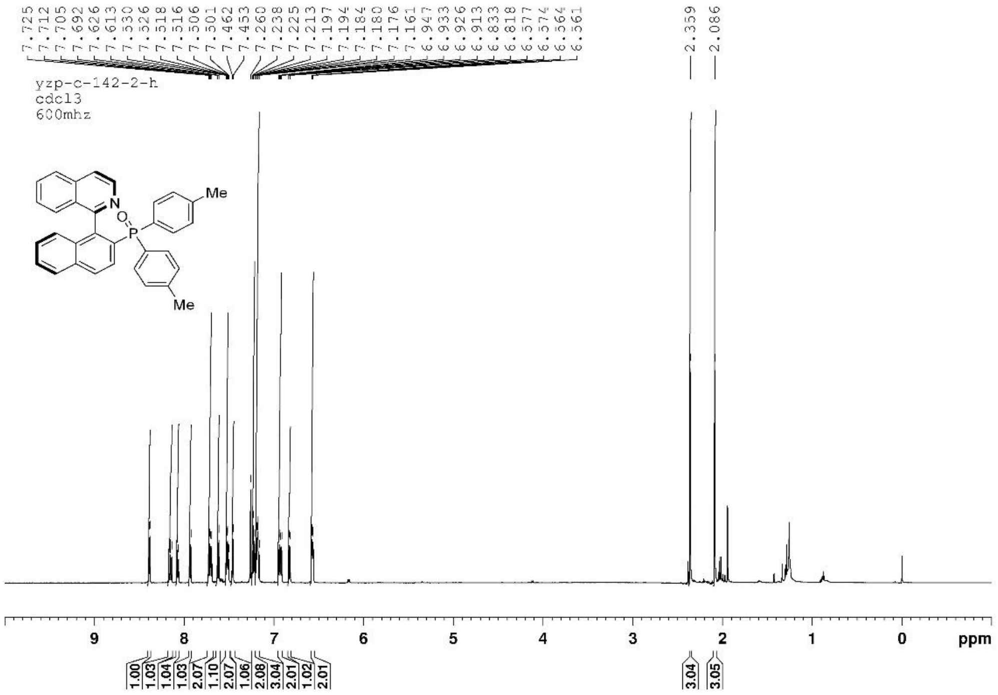

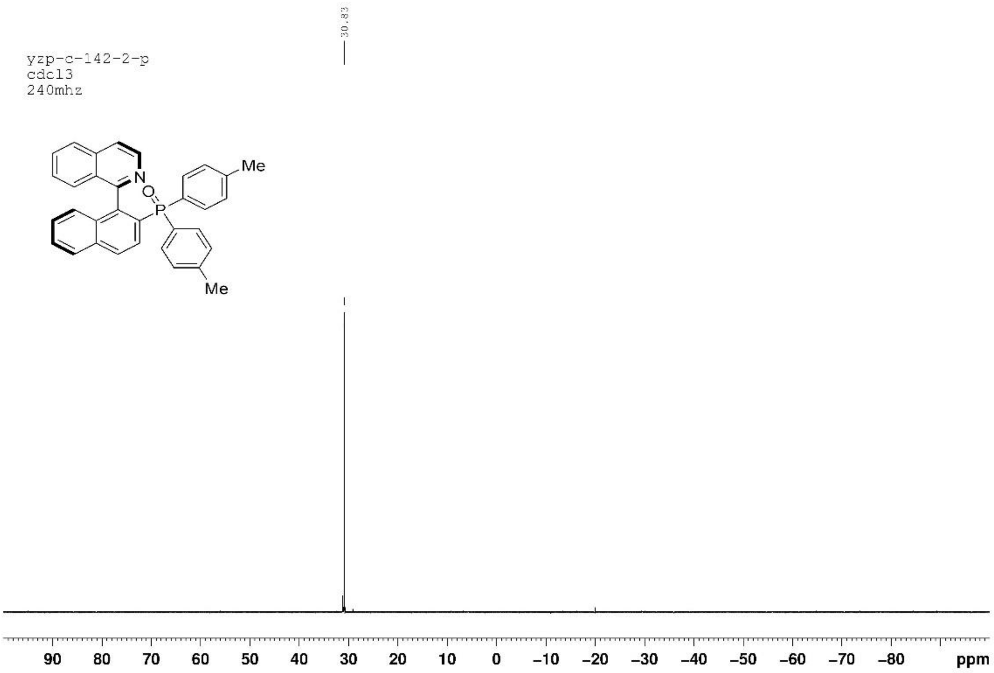

(*R*)-(1-(Isoquinolin-1-yl)naphthalen-2-yl)di-m-tolylphosphine oxide (3ac)

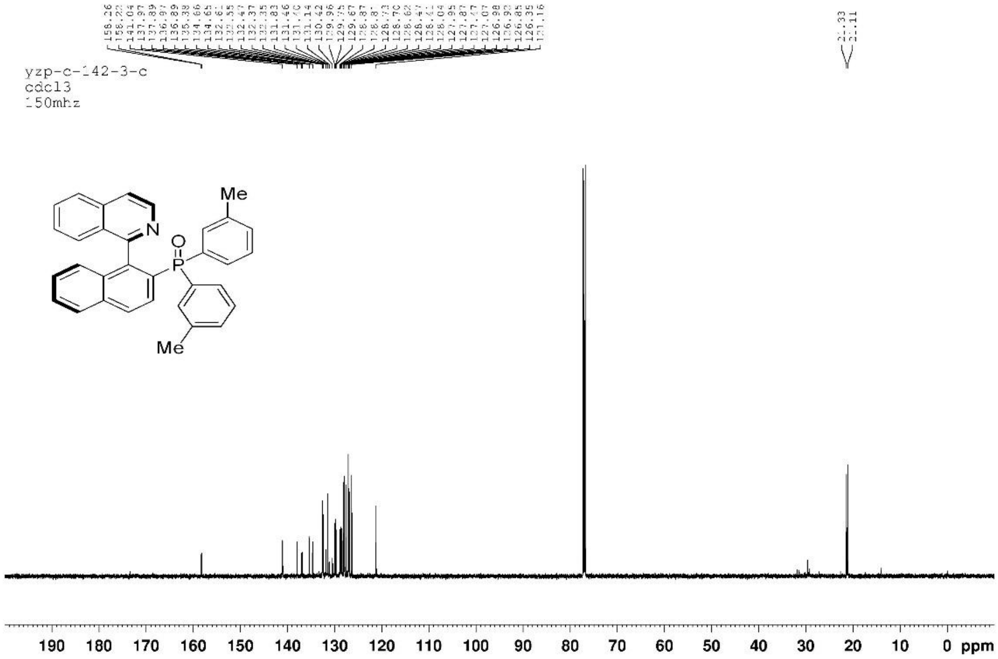

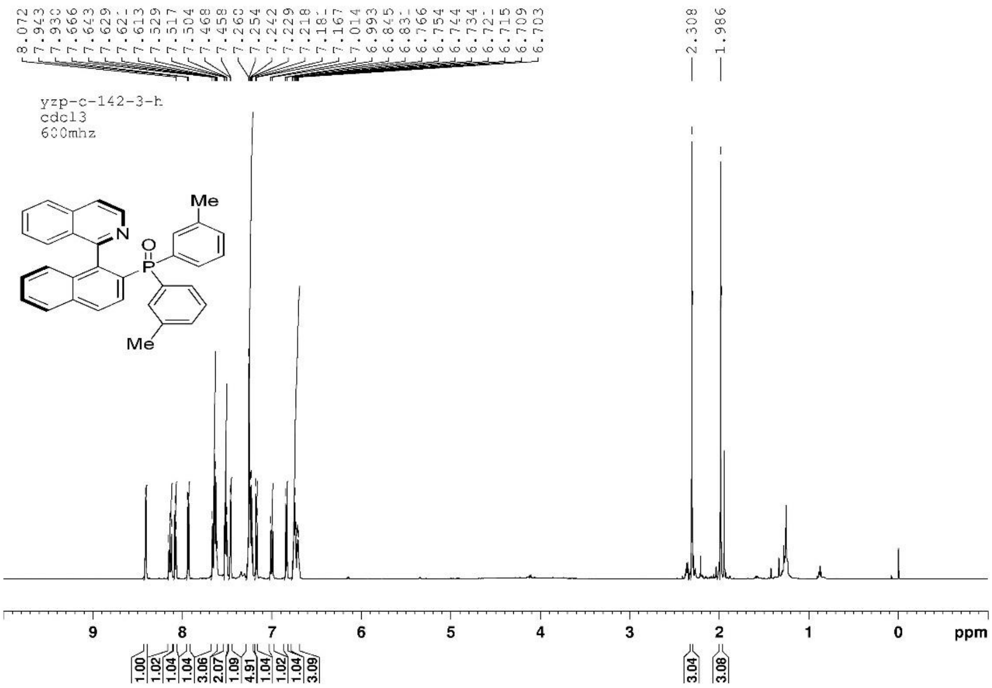
8.072
7.943
7.930
7.666
7.643
7.629
7.613
7.529
7.517
7.504
7.468
7.458
7.260
7.254
7.242
7.229
7.218
7.167
7.014
6.993
6.845
6.766
6.754
6.744
6.734
6.715
6.709
6.703
2.308
1.986
yzp-c-142-3-h
cdcl3
600mhz
Me
N
O
P
Me
9
8
7
6
5
4
3
2
1
0
ppm
1.00
1.02
1.04
1.04
3.06
2.07
1.09
4.91
1.04
1.02
1.04
3.09
3.04
3.08

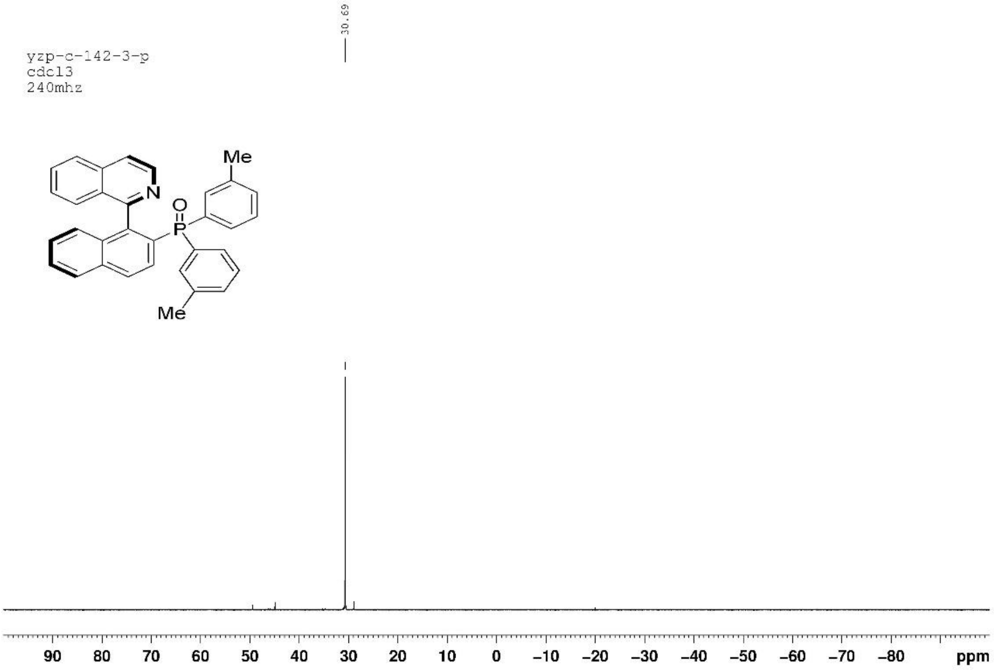
30.69
yzp-c-142-3-p
cdcl3
240mhz
Me
N
O
P
Me
90
80
70
60
50
40
30
20
10
0
−10
−20
−30
−40
−50
−60
−70
−80
ppm

(*R*)-bis(4-(*tert*-Butyl)phenyl)(1-(isoquinolin-1-yl)naphthalen-2-yl)phosphine oxide (3ae)

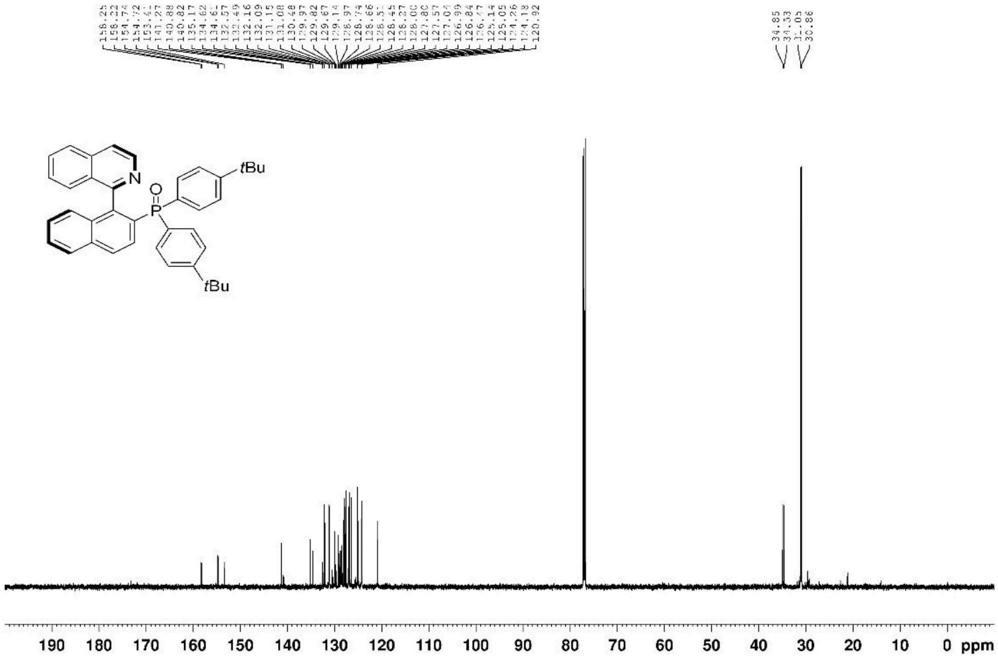

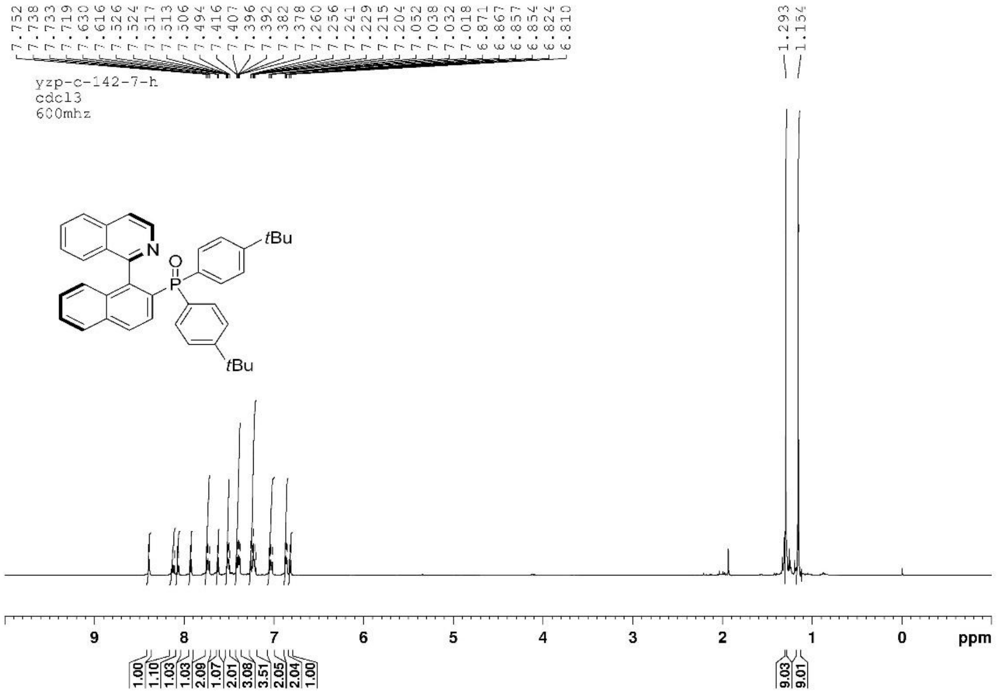

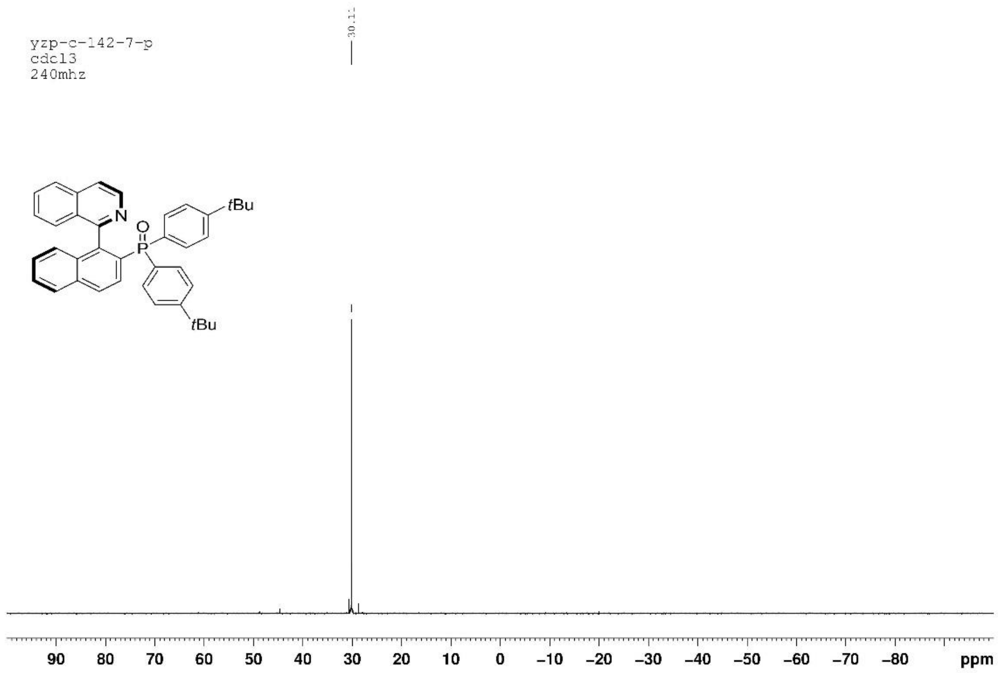

(*R*)-(1-(Isoquinolin-1-yl)naphthalen-2-yl)bis(4-methoxyphenyl)phosphine oxide (3af)

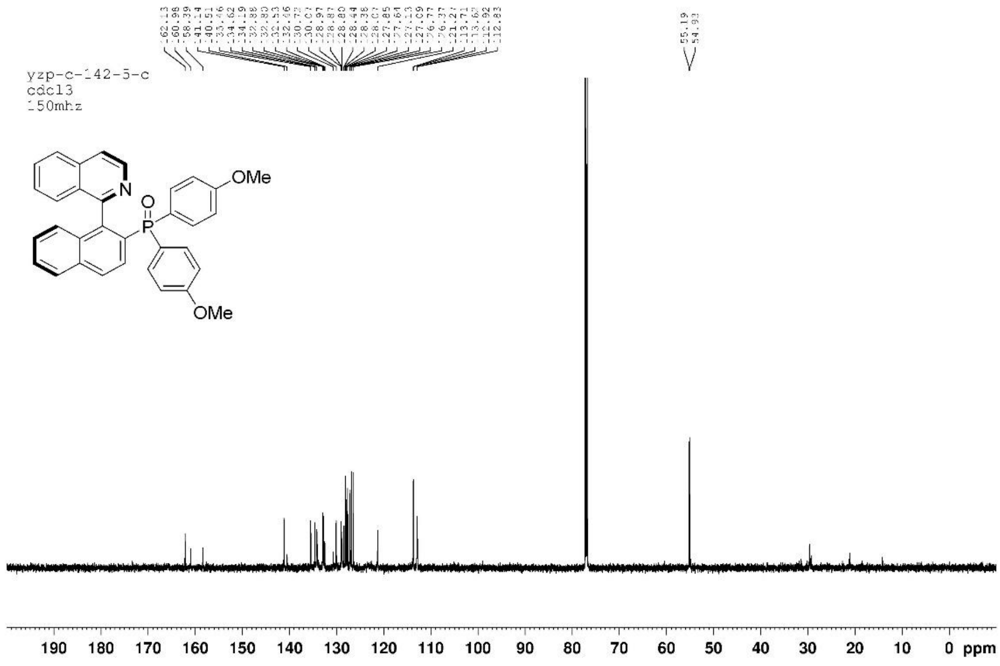

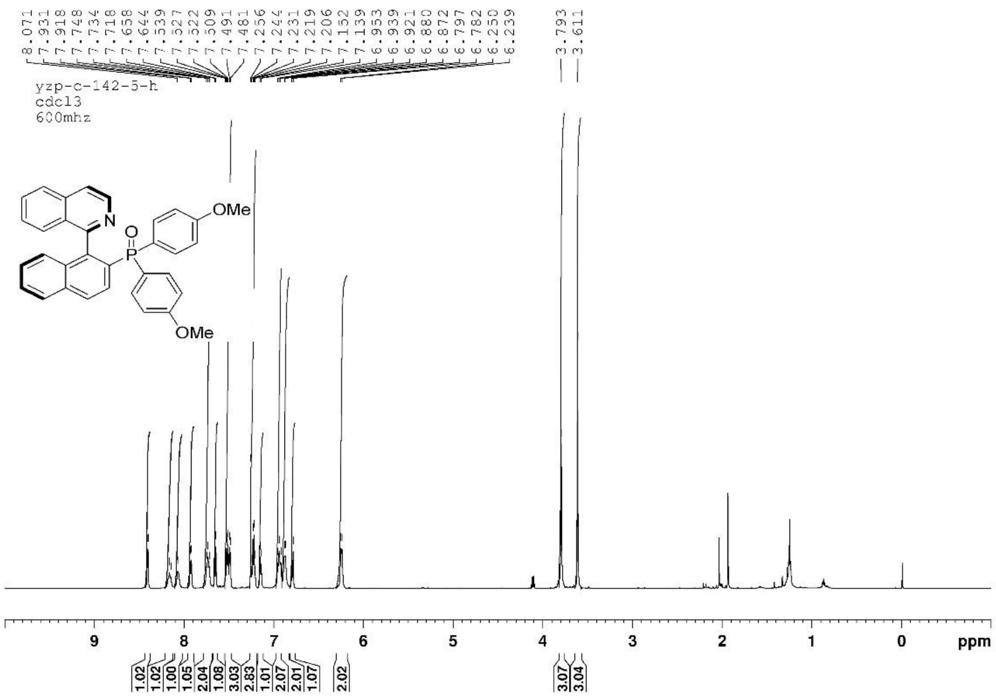
yzp-c-142-5-h
cdcl3
600mhz
OMe
N
O
P
OMe
ppm

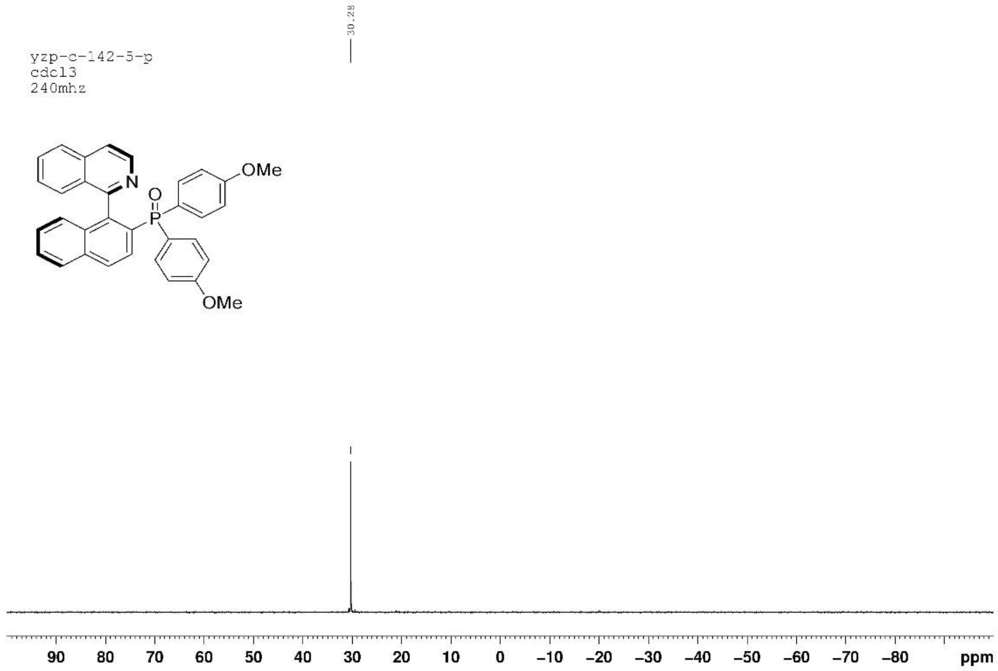
30.28
yzp-c-142-5-p
cdcl3
240mhz
OMe
N
O
P
OMe
ppm

(*R*)-di([1,1'-Biphenyl]-4-yl)(1-(isoquinolin-1-yl)naphthalen-2-yl)phosphine oxide (3ag)

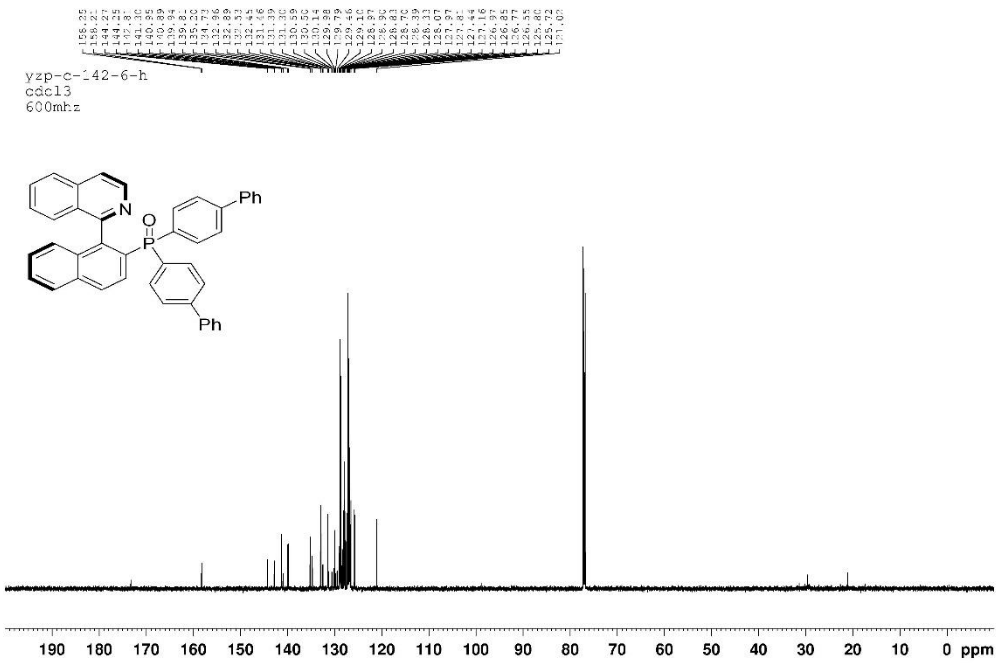

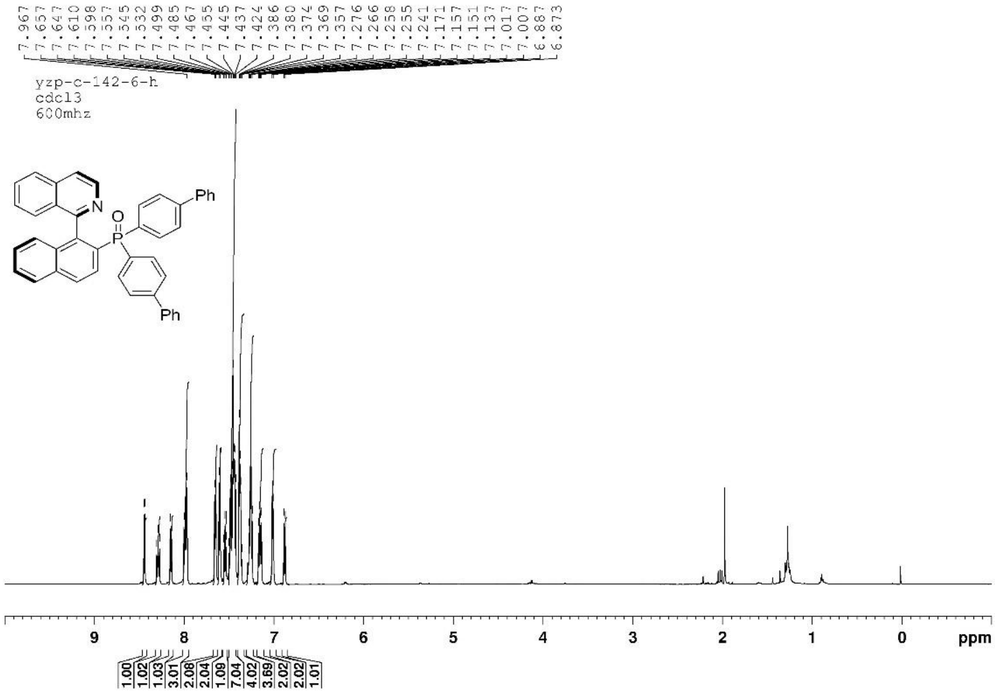

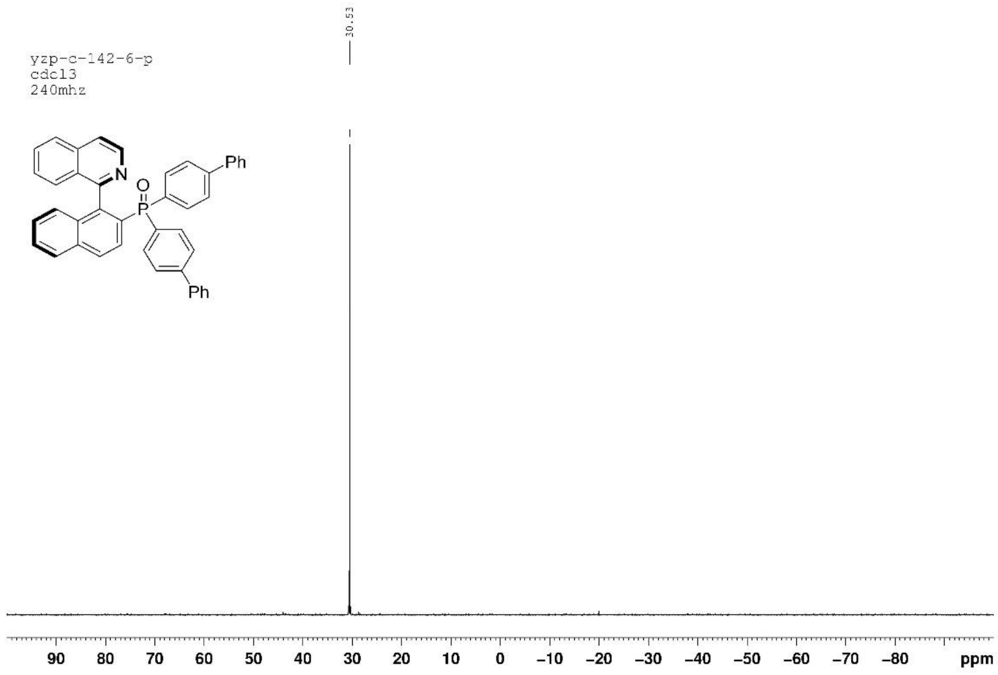

(*R*)-bis(4-Fluorophenyl)(1-(isoquinolin-1-yl)naphthalen-2-yl)phosphine oxide (3ah)

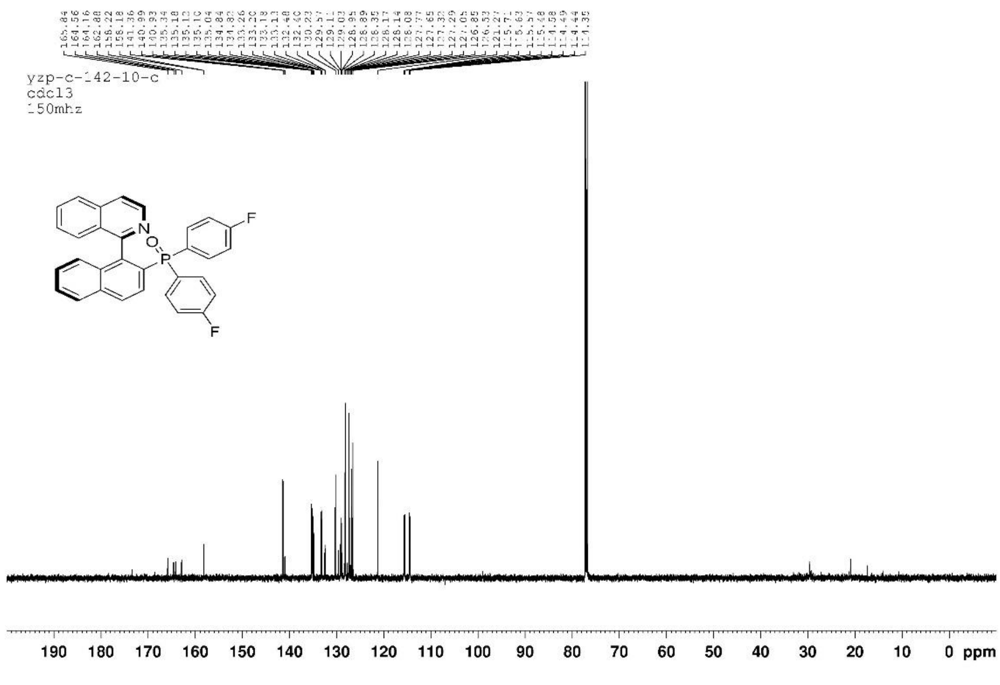

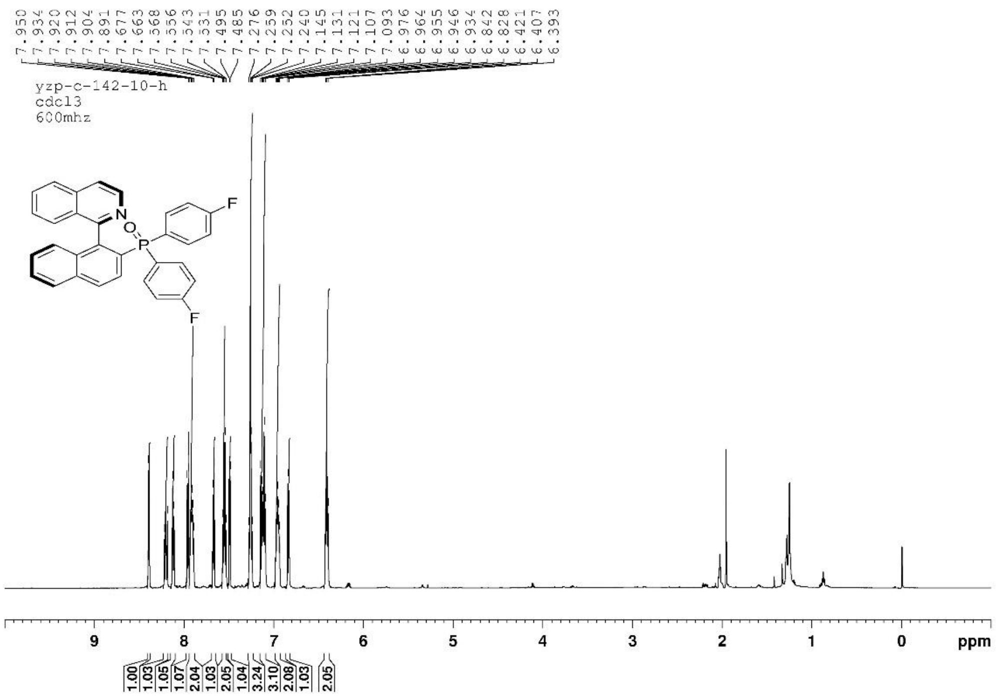
7.950
7.934
7.920
7.912
7.904
7.891
7.677
7.663
7.568
7.556
7.543
7.531
7.495
7.485
7.276
7.259
7.252
7.240
7.145
7.131
7.121
7.107
7.093
6.976
6.964
6.955
6.946
6.934
6.842
6.828
6.421
6.407
6.393
yzp-c-142-10-h
cdcl3
600mhz
N
O
P
F
F
9
8
7
6
5
4
3
2
1
0
ppm
1.00
1.03
1.05
1.07
2.04
1.03
2.05
1.04
3.24
3.10
2.08
1.03
2.05

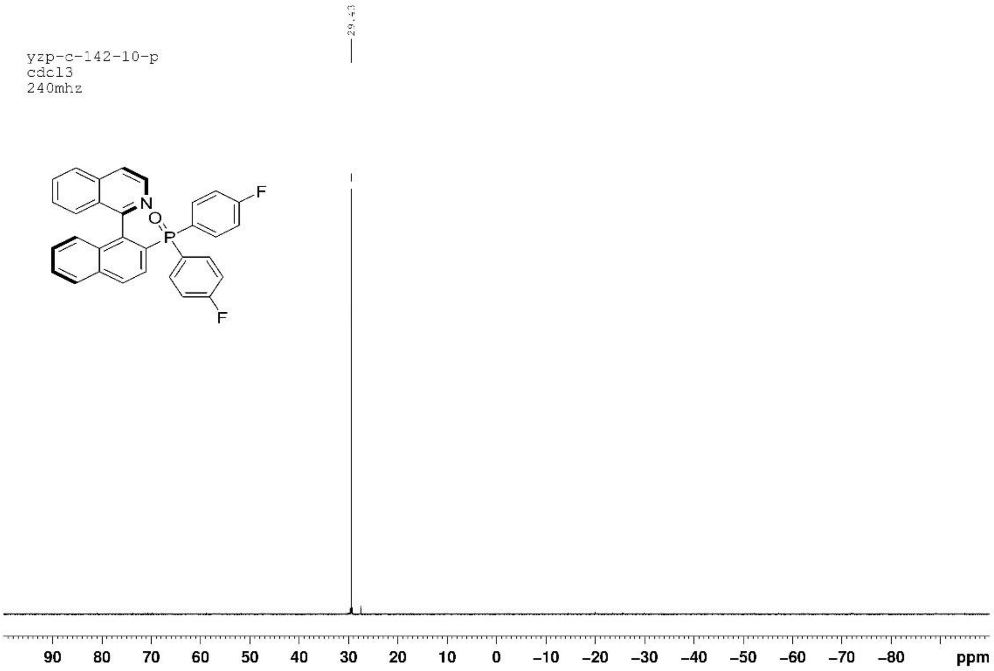
29.43
yzp-c-142-10-p
cdcl3
240mhz
N
O
P
F
F
90
80
70
60
50
40
30
20
10
0
−10
−20
−30
−40
−50
−60
−70
−80
ppm

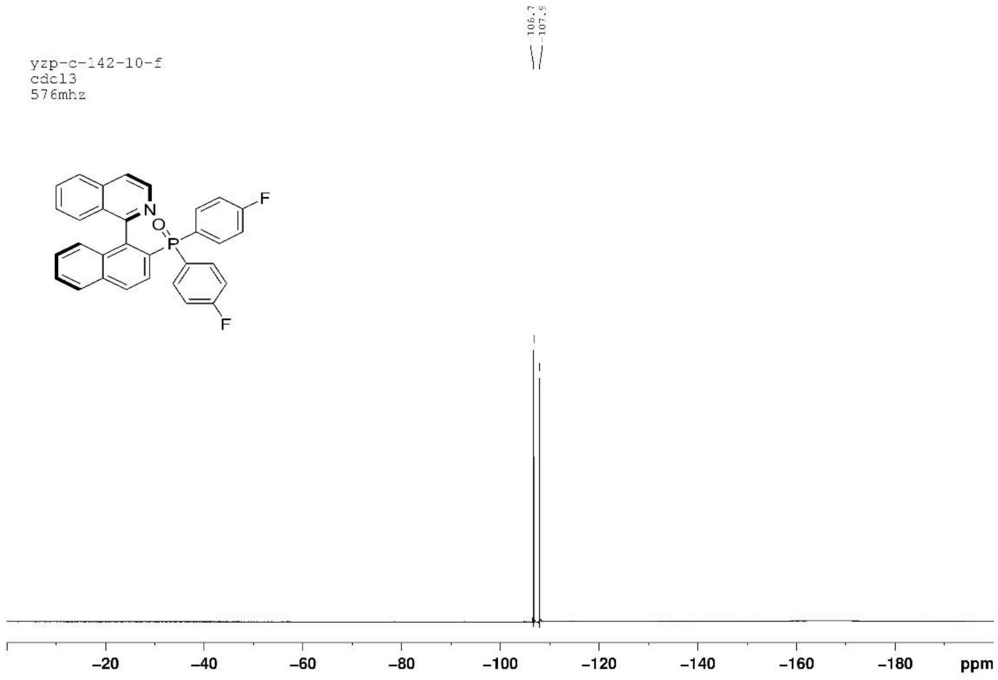

(*R*)-bis(4-Chlorophenyl)(1-(isoquinolin-1-yl)naphthalen-2-yl)phosphine oxide (3ai)

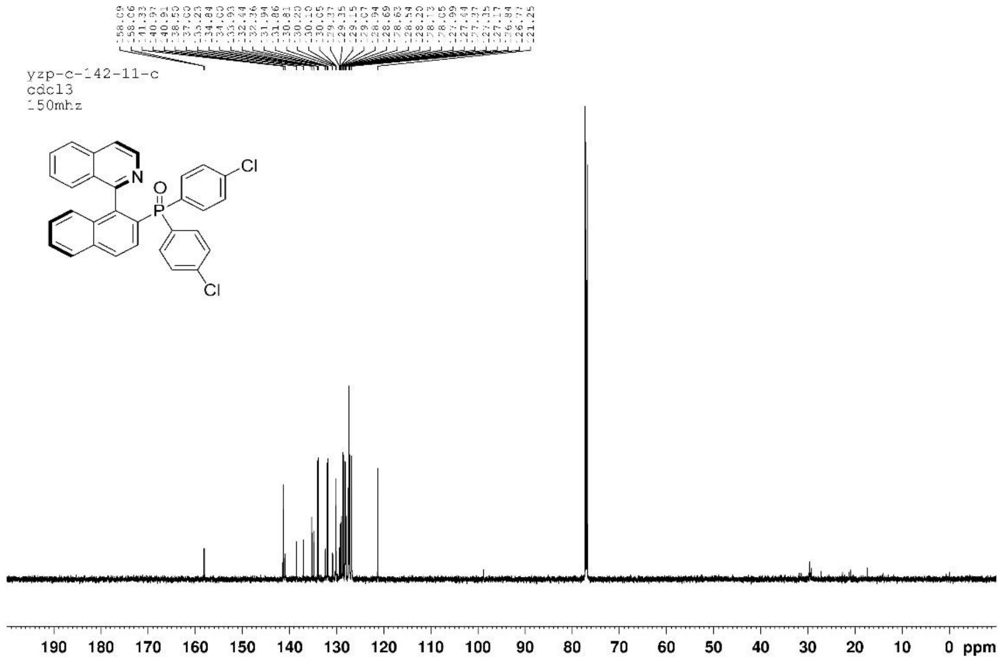

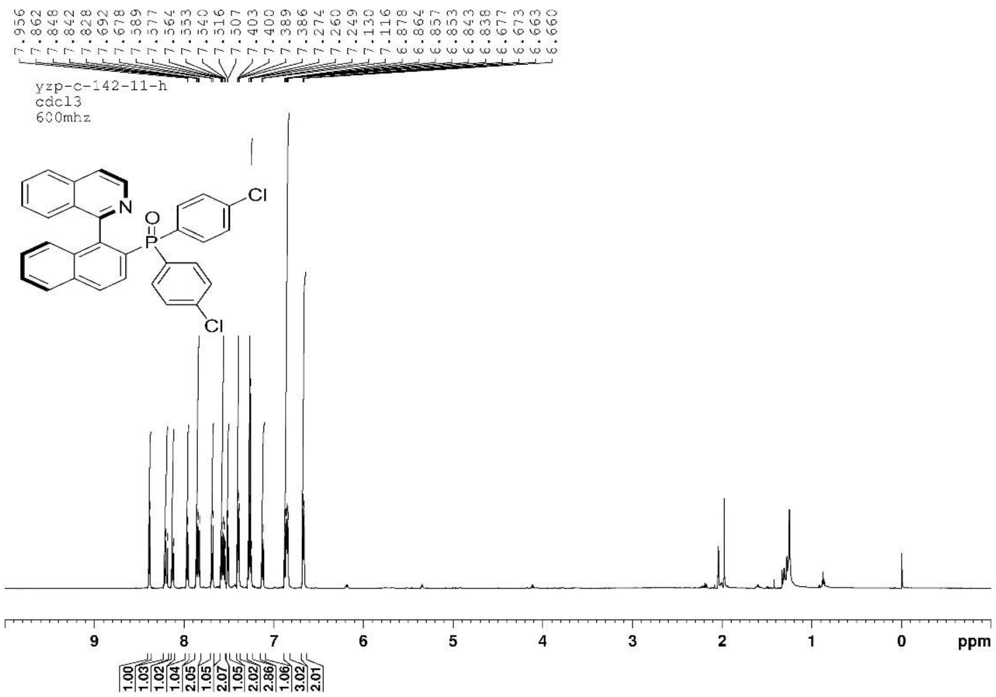

7.956
7.862
7.848
7.842
7.828
7.692
7.678
7.589
7.577
7.564
7.553
7.540
7.516
7.507
7.403
7.400
7.389
7.386
7.274
7.260
7.249
7.130
7.116
6.878
6.864
6.857
6.853
6.843
6.838
6.677
6.673
6.663
6.660
yzp-c-142-11-h
cdcl3
600mhz
Cl
N
O
P
Cl
9
8
7
6
5
4
3
2
1
0
ppm
1.00
1.03
1.02
1.04
2.05
1.05
2.07
1.05
2.02
2.86
1.06
3.02
2.01

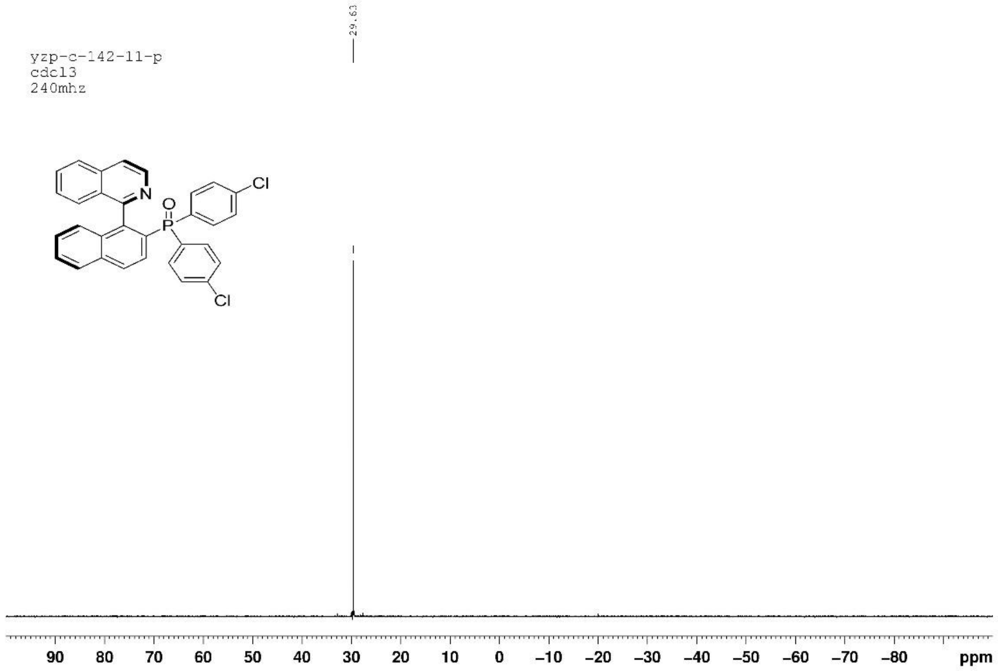

29.63
yzp-c-142-11-p
cdcl3
240mhz
Cl
N
O
P
Cl
90
80
70
60
50
40
30
20
10
0
−10
−20
−30
−40
−50
−60
−70
−80
ppm

(*R*)-(1-(Isoquinolin-1-yl)naphthalen-2-yl)bis(4-(trifluoromethyl)phenyl)phosphine oxide (3aj)

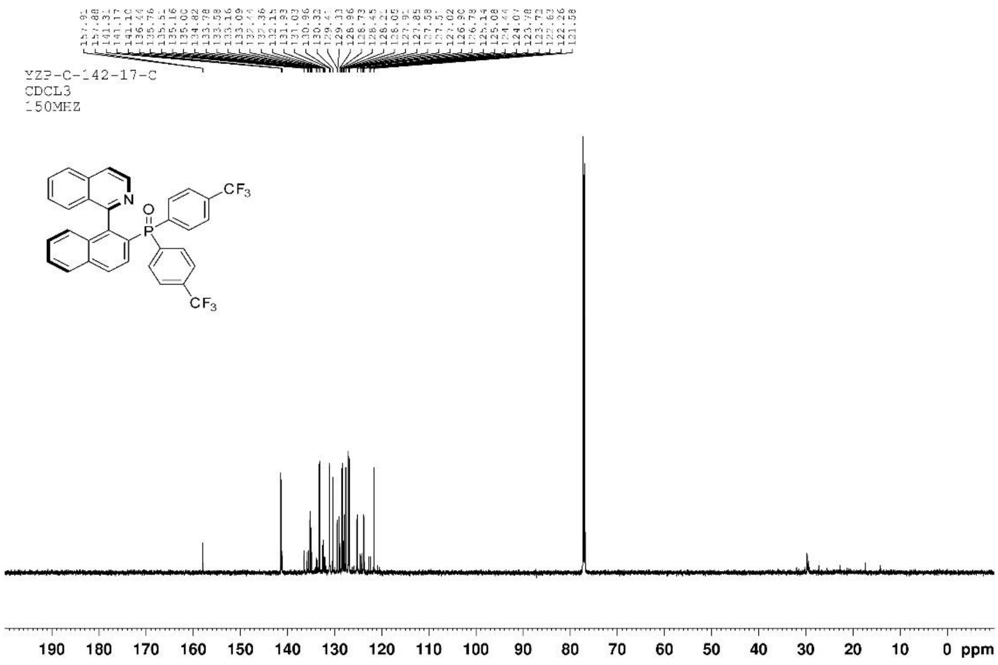

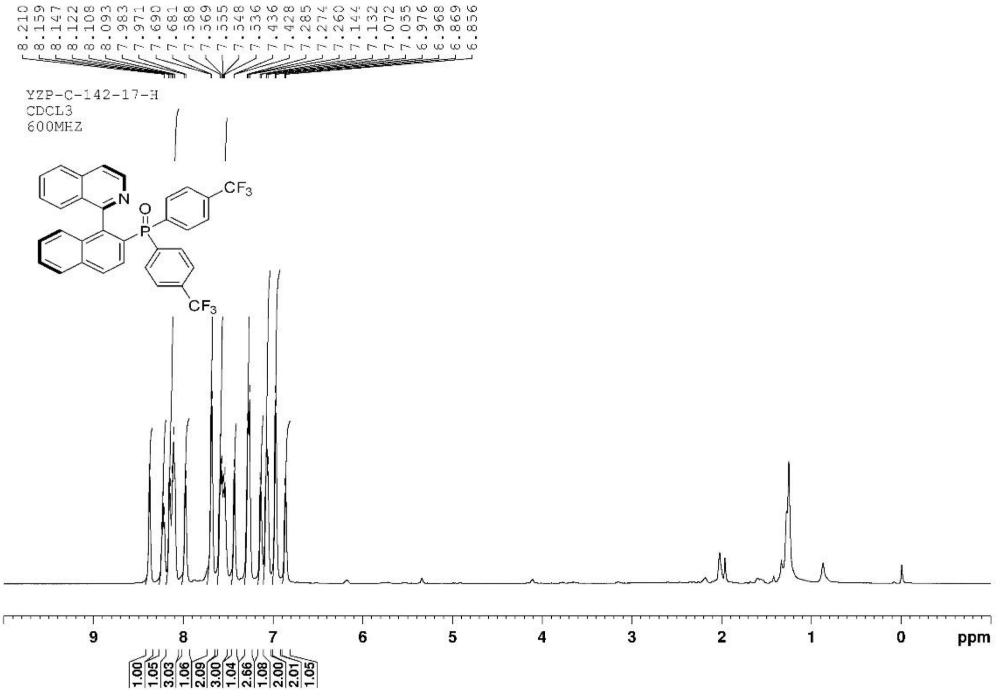

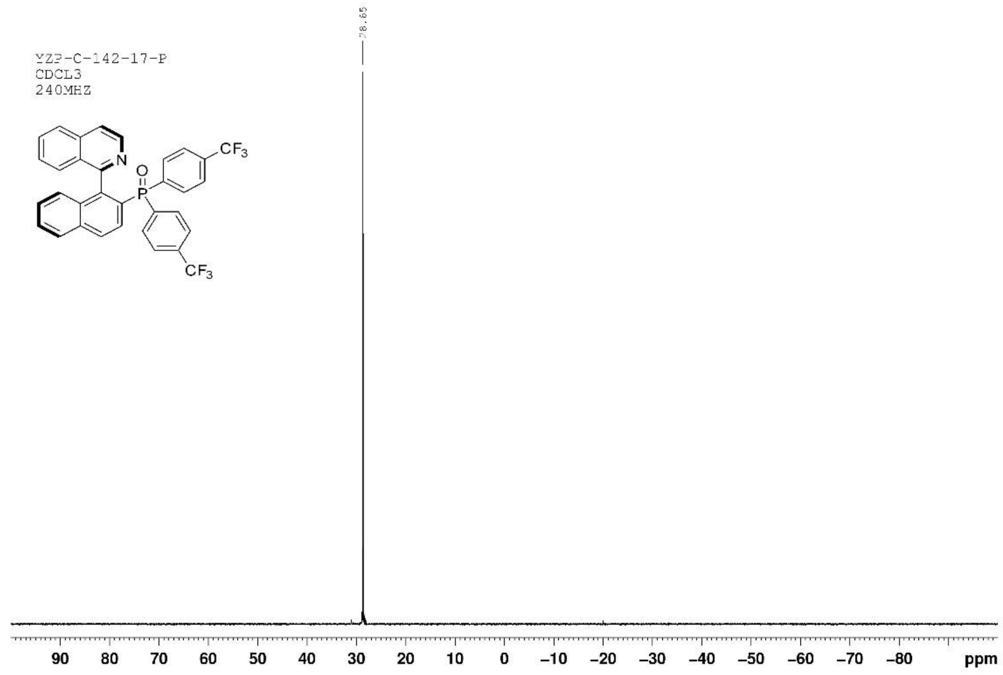
YZP-C-142-17-P
CDCL3
240MHZ
28.65
N
O
P
CF3
CF3
90 80 70 60 50 40 30 20 10 0 –10 –20 –30 –40 –50 –60 –70 –80 ppm

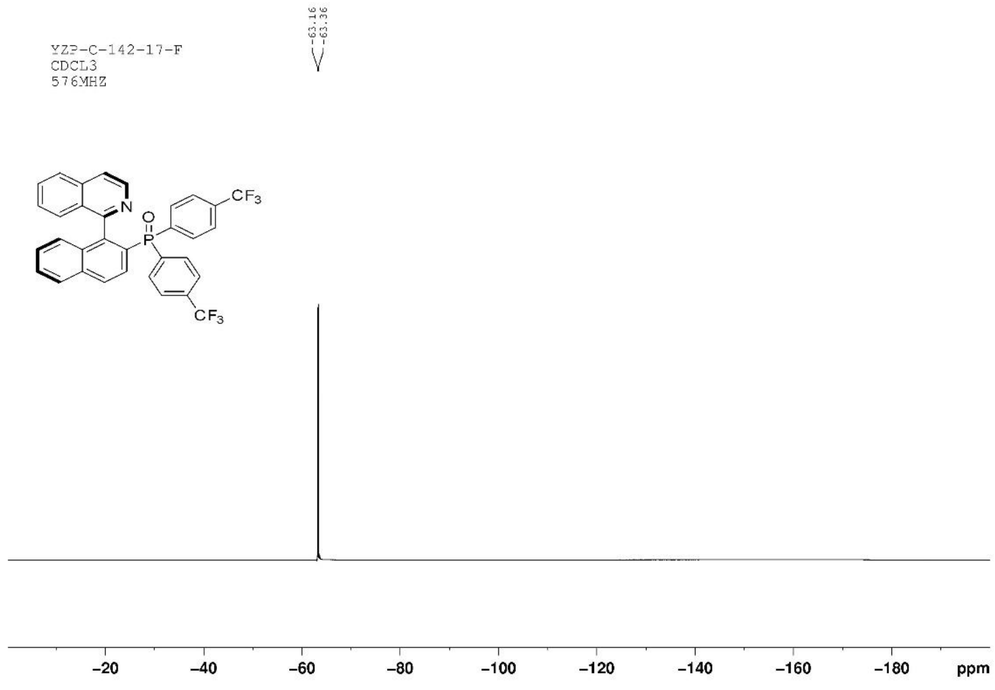
YZP-C-142-17-F
CDCL3
576MHZ
-63.16
-63.36
N
O
P
CF3
CF3
–20 –40 –60 –80 –100 –120 –140 –160 –180 ppm

(*R*)-bis(3,5-Dimethylphenyl)(1-(isoquinolin-1-yl)naphthalen-2-yl)phosphine oxide (3ak)

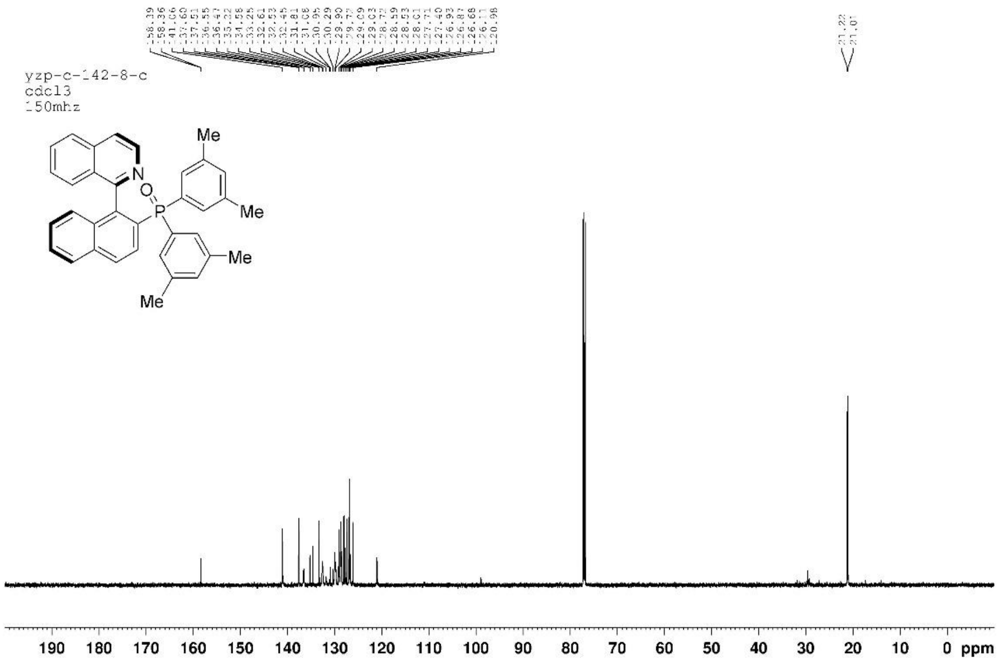

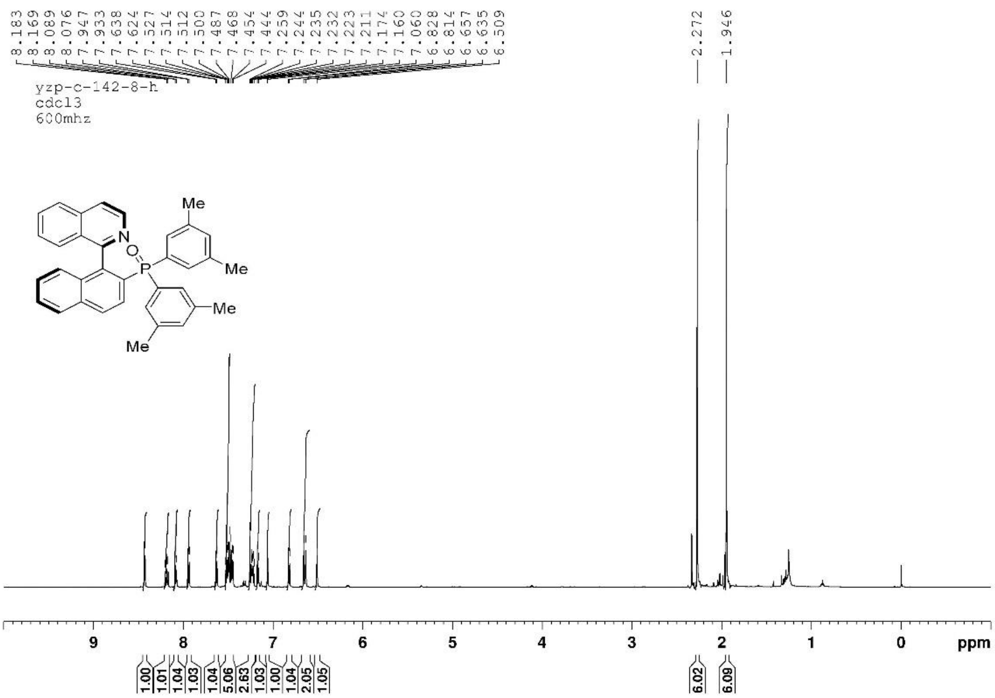

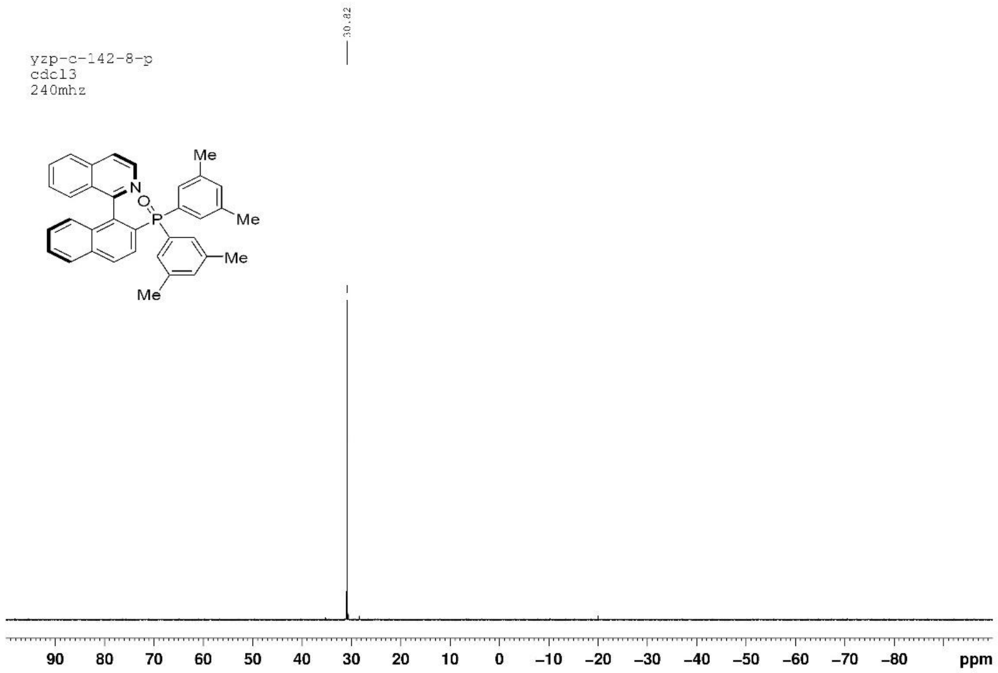

(*R*)-(1-(Isoquinolin-1-yl)naphthalen-2-yl)bis(4-methoxy-3,5-dimethylphenyl)phosphine oxide (3al)

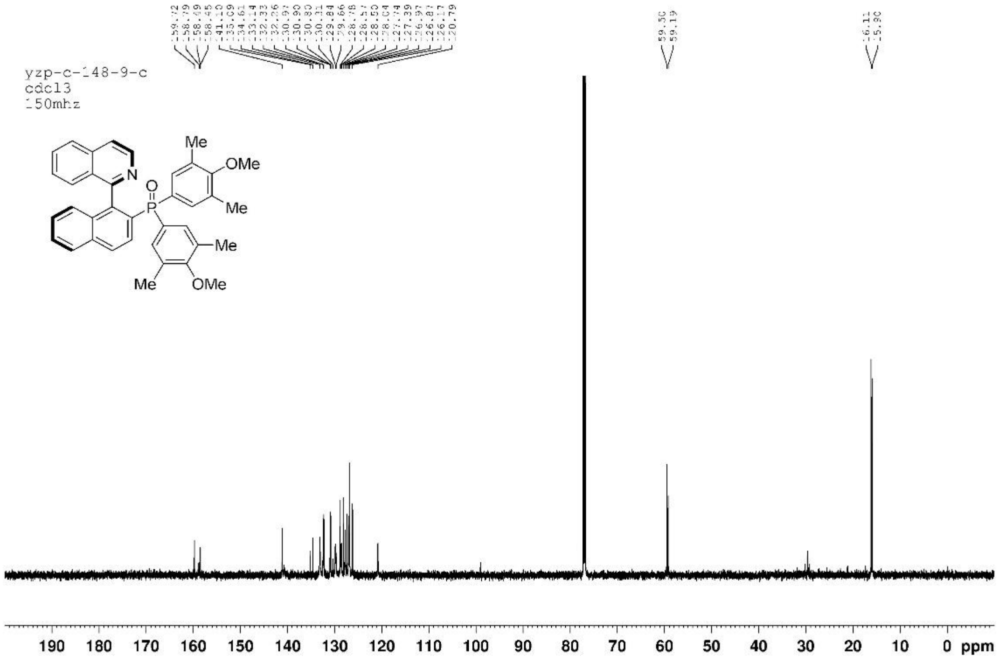

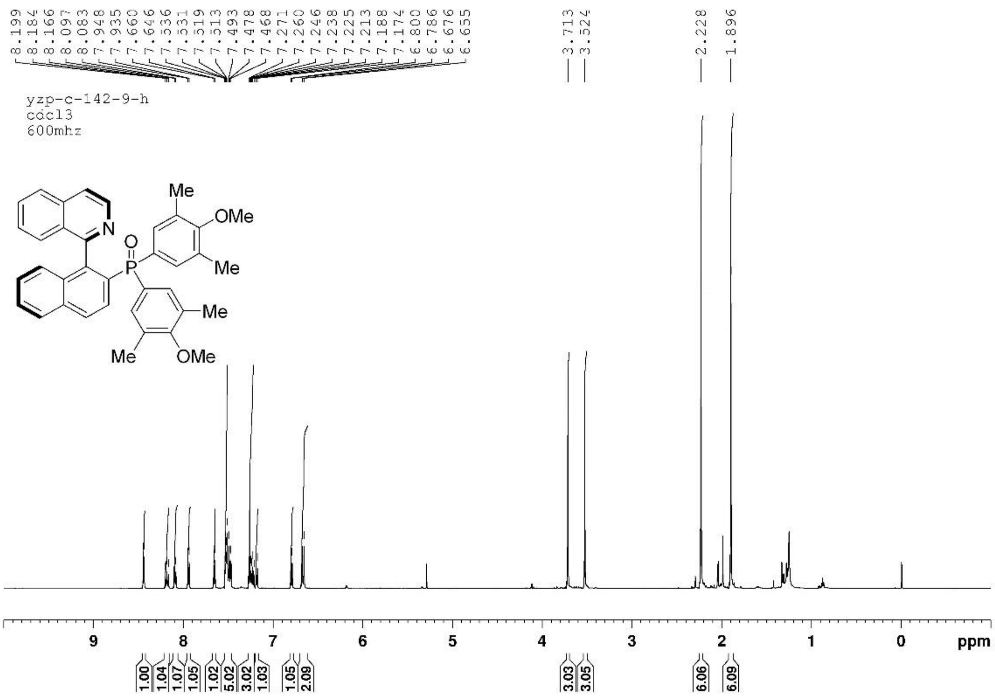
yzp-c-142-9-h
cdcl3
600mhz
ppm

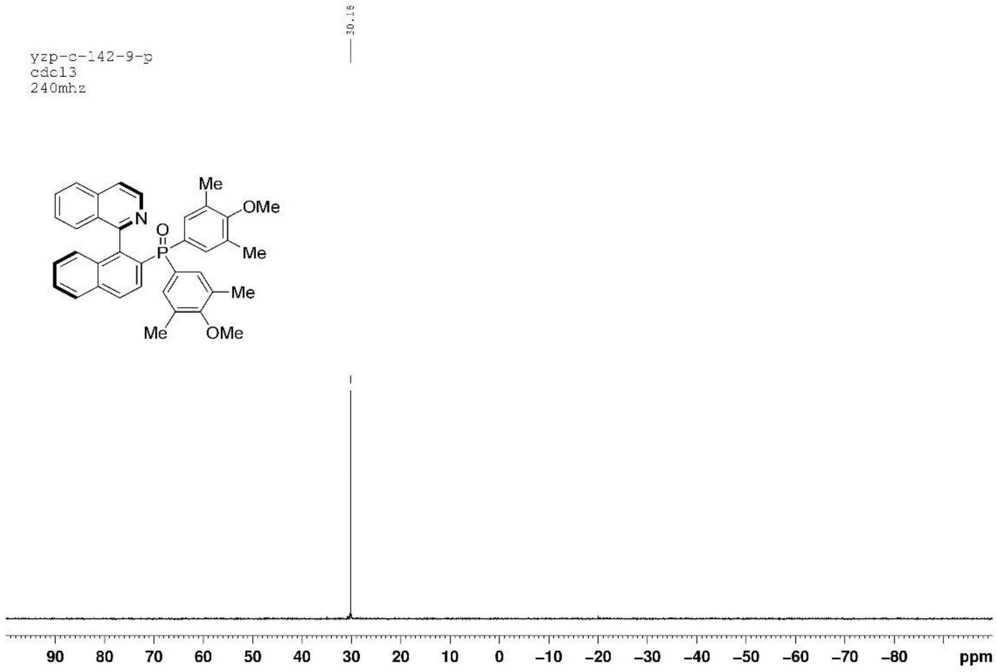
yzp-c-142-9-p
cdcl3
240mhz
ppm

(*R*)-(1-(Isoquinolin-1-yl)naphthalen-2-yl)di(naphthalen-2-yl)phosphine oxide (3am)

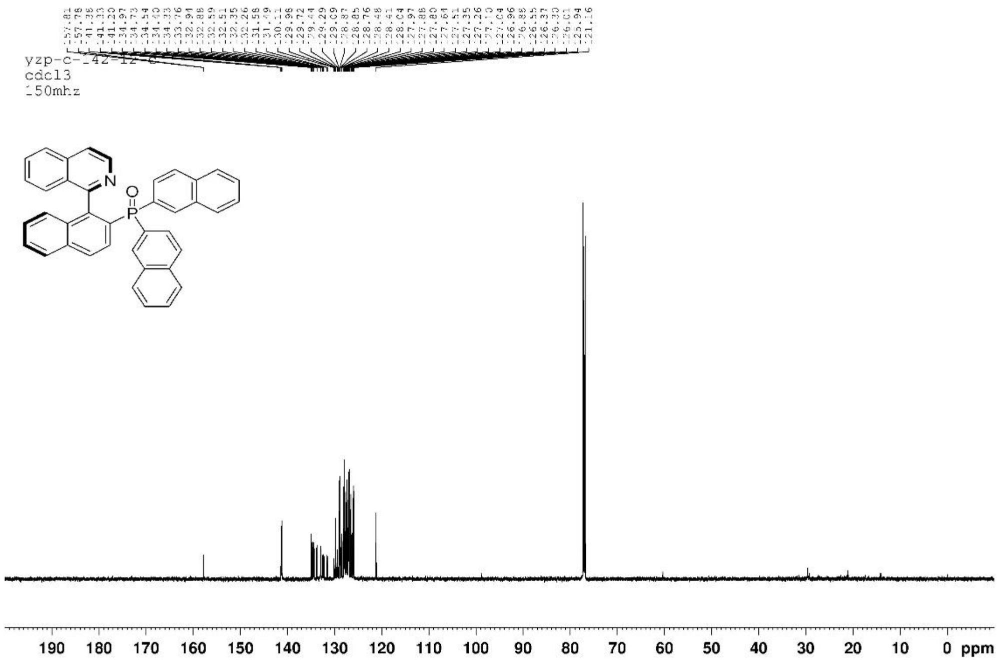

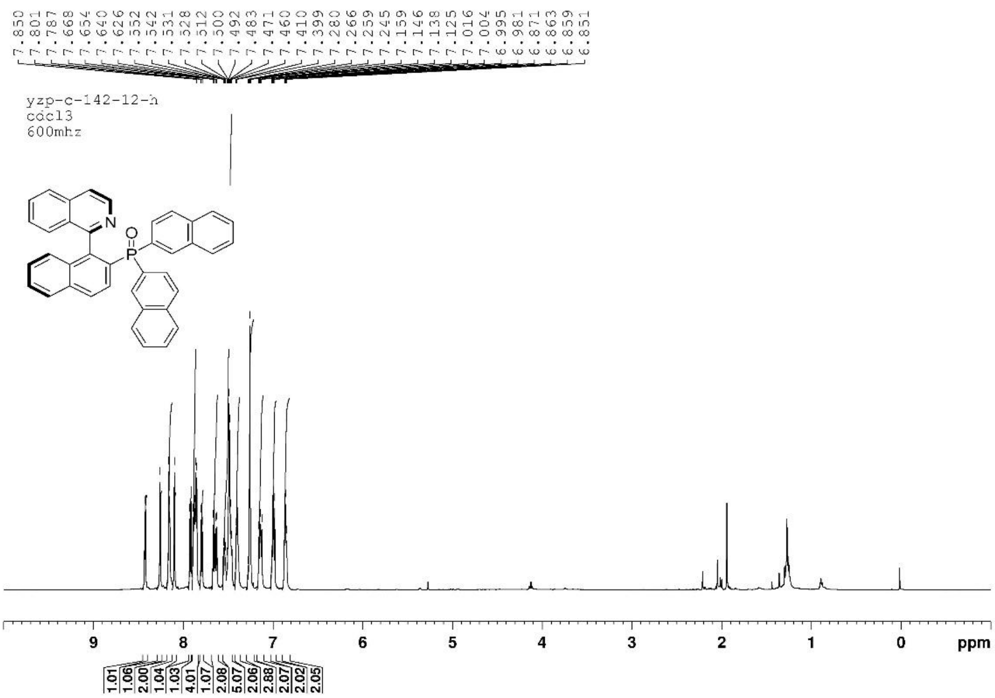

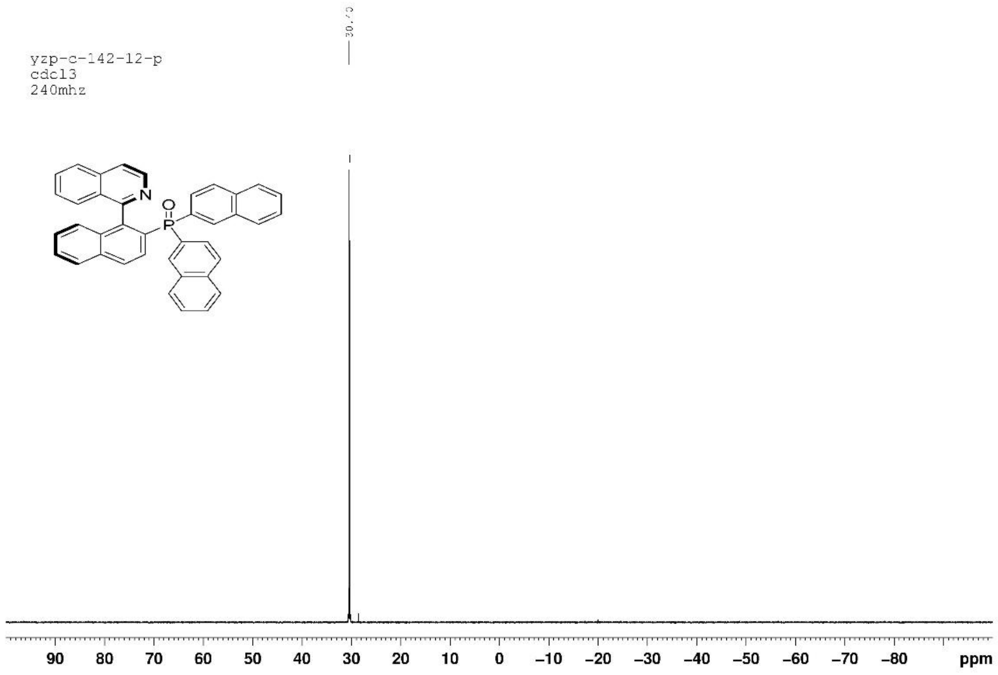

(*R*)-Dibenzyl(1-(isoquinolin-1-yl)naphthalen-2-yl)phosphine oxide (3an)

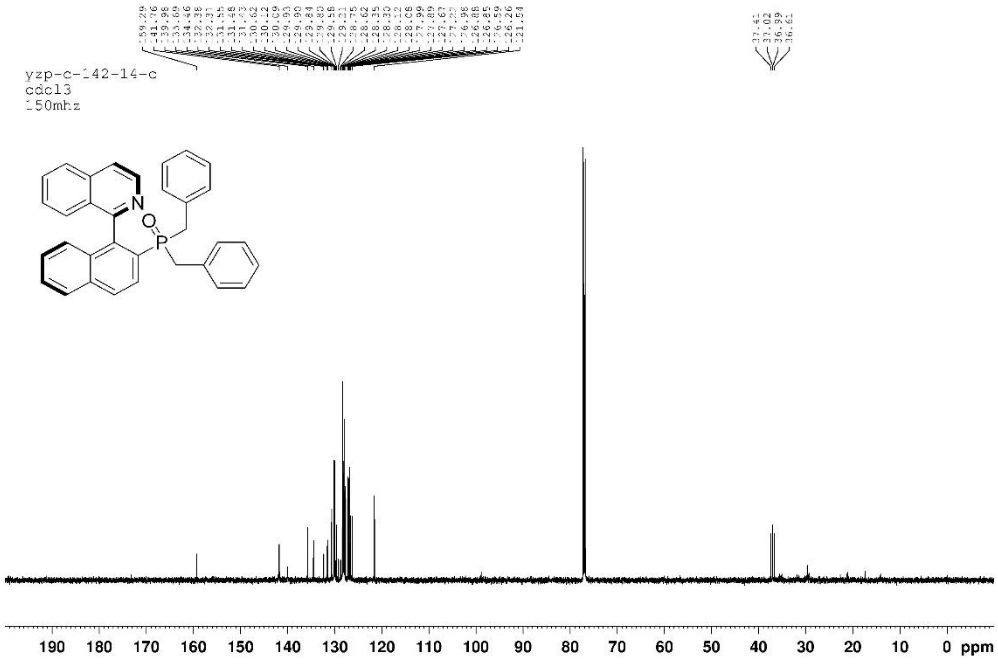

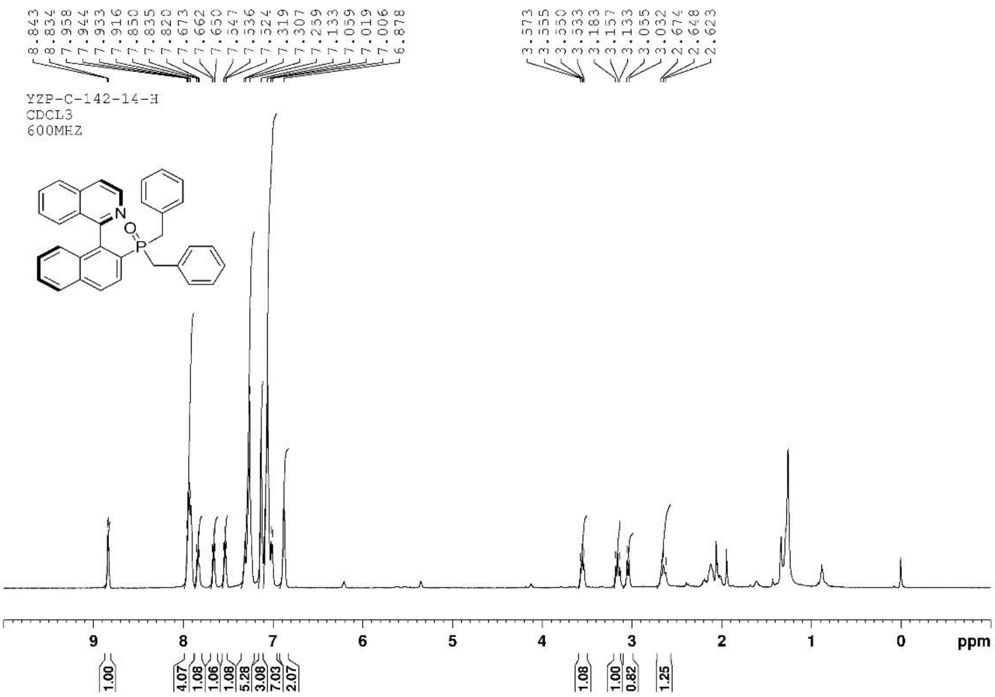
8.843
8.834
7.958
7.944
7.933
7.916
7.850
7.835
7.820
7.673
7.662
7.650
7.547
7.536
7.524
7.319
7.307
7.259
7.133
7.059
7.019
7.006
6.878
3.573
3.555
3.550
3.533
3.183
3.157
3.133
3.055
3.032
2.674
2.648
2.623
YZP-C-142-14-H
CDCL3
600MHZ
N
O
P
9
8
7
6
5
4
3
2
1
0
ppm
1.00
4.07
1.08
1.06
1.08
5.28
3.08
7.03
2.07
1.08
1.00
0.82
1.25

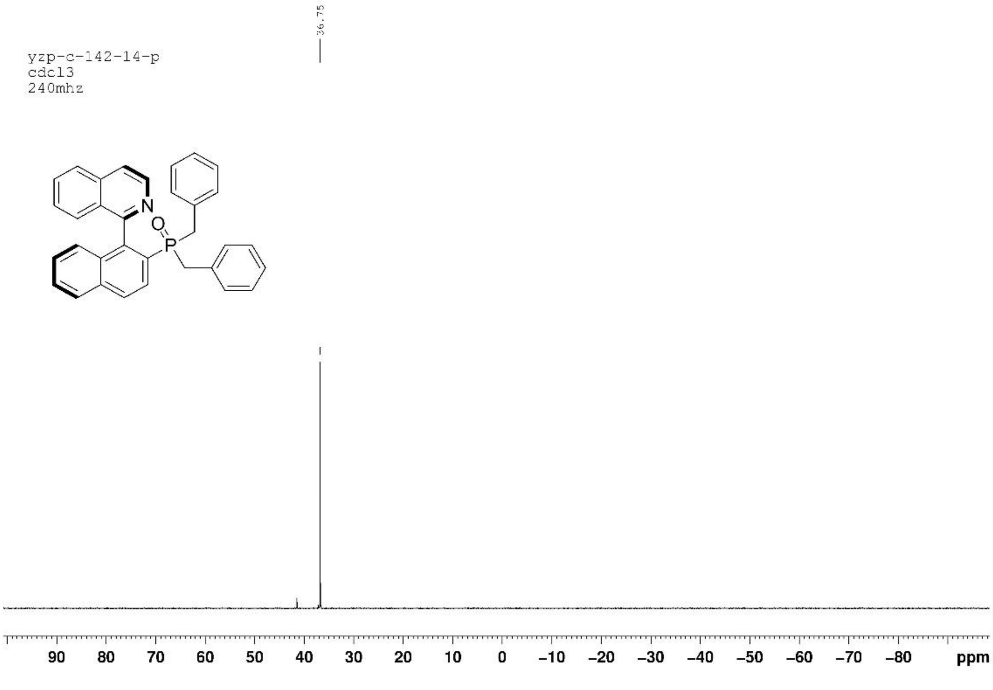
36.75
yzp-c-142-14-p
cdcl3
240mhz
N
O
P
90
80
70
60
50
40
30
20
10
0
−10
−20
−30
−40
−50
−60
−70
−80
ppm

(*R*)-Ethyl(1-(isoquinolin-1-yl)naphthalen-2-yl)(phenyl)phosphine oxide (3ao)

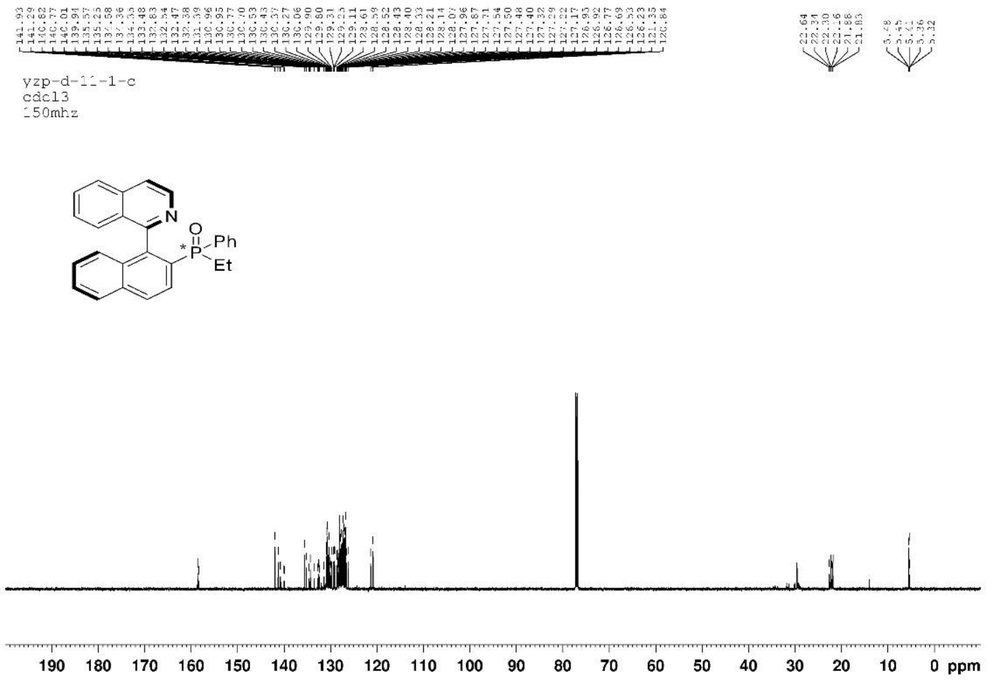

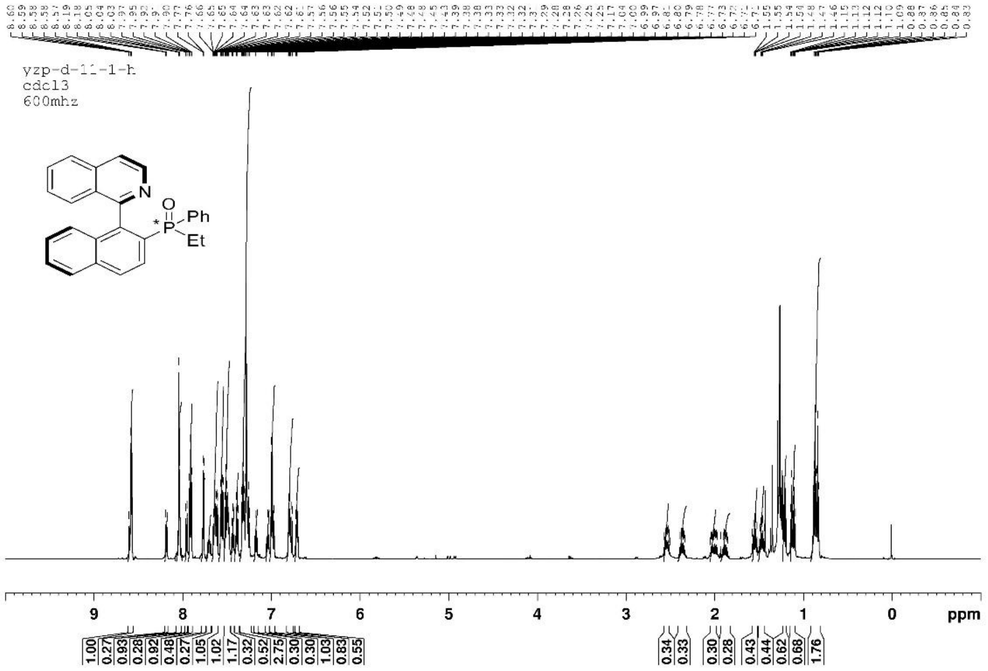

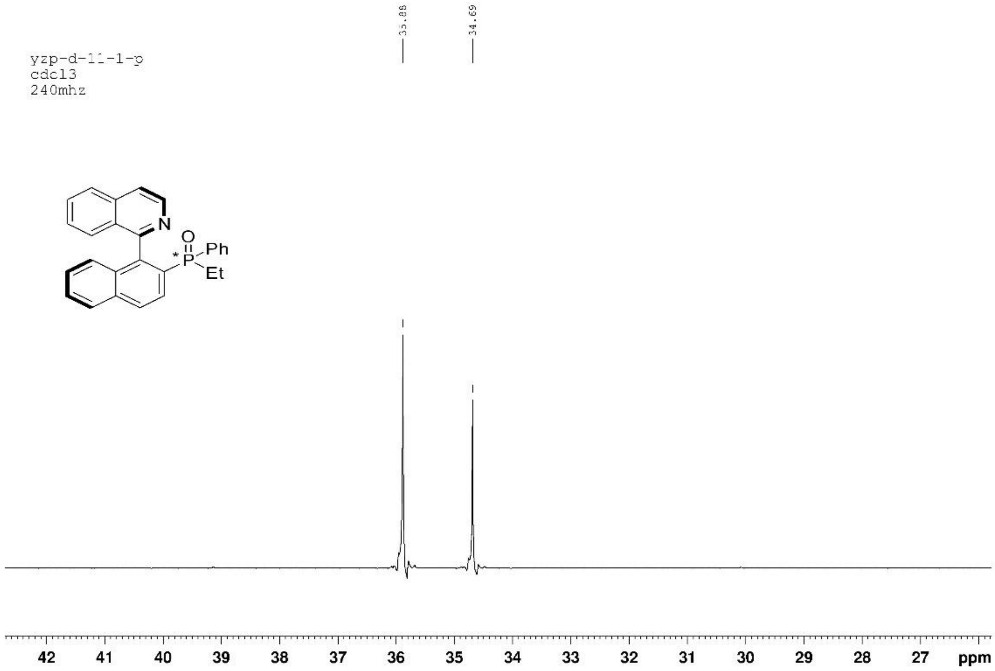

(*R*)-Ethyl (1-(isoquinolin-1-yl)naphthalen-2-yl)(phenyl)phosphinate (3ap)

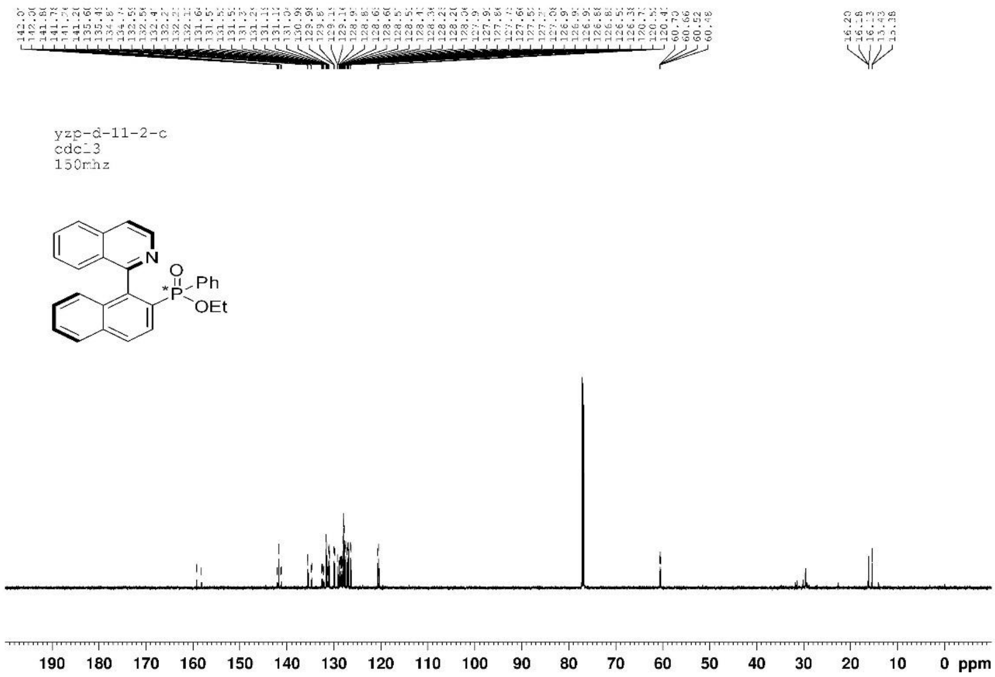

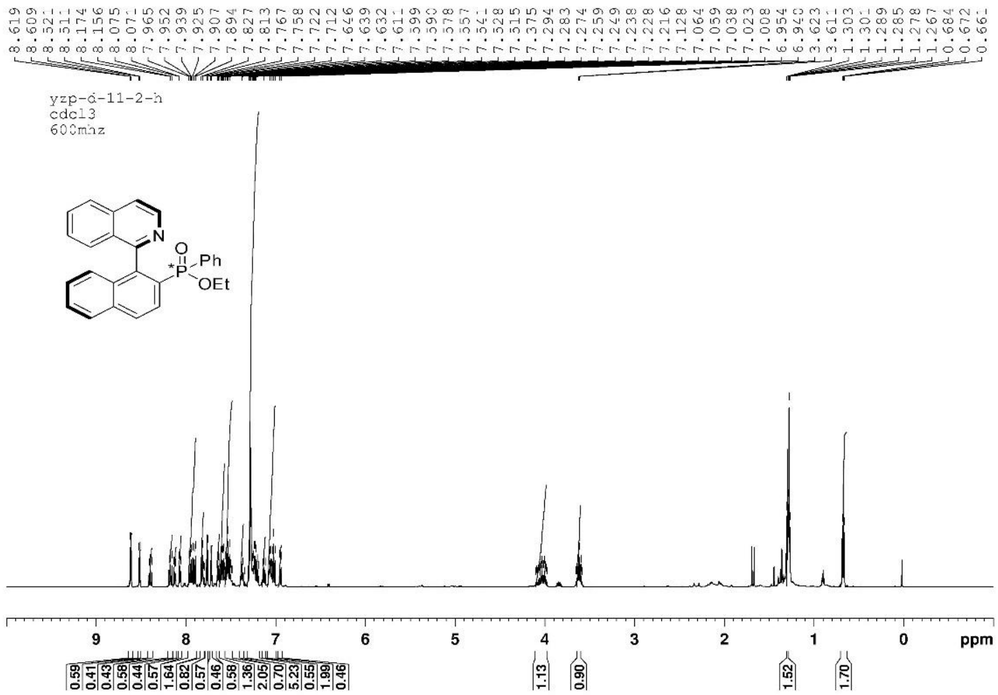

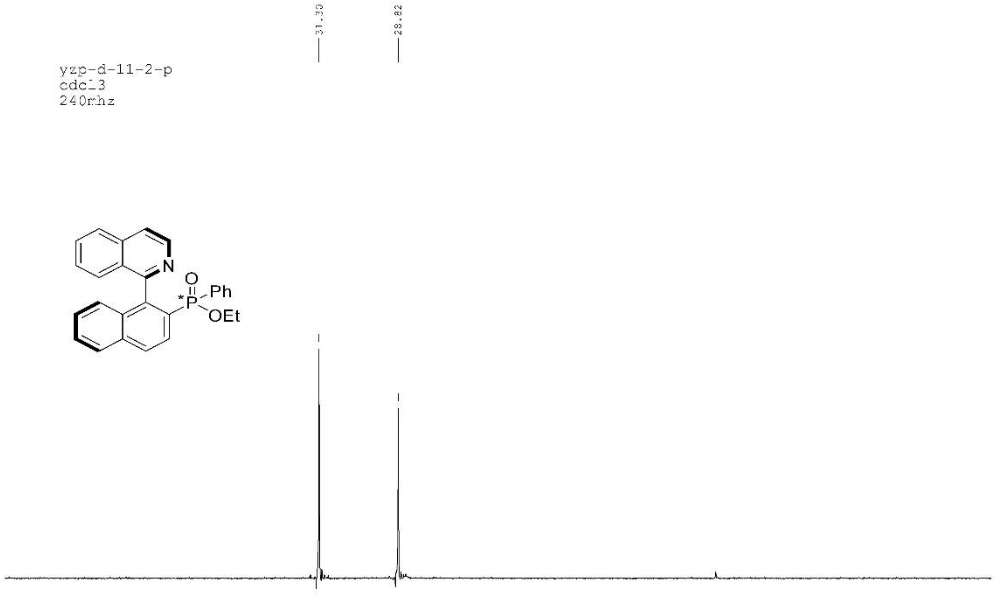

(*R*)-1-(2-(Diphenylphosphaneyl)naphthalen-1-yl)isoquinoline (3aa’)

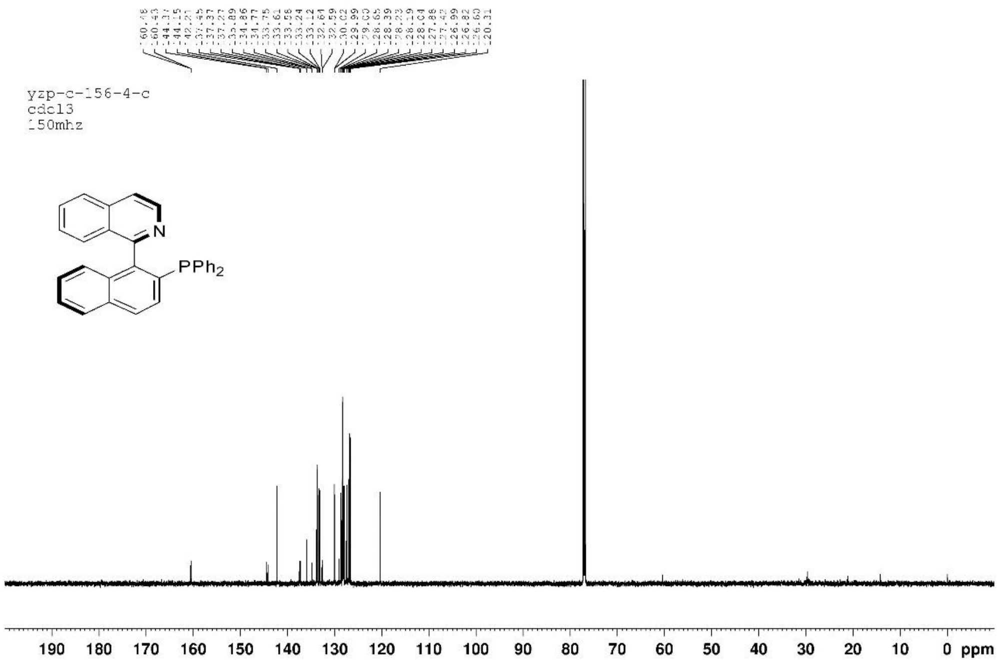

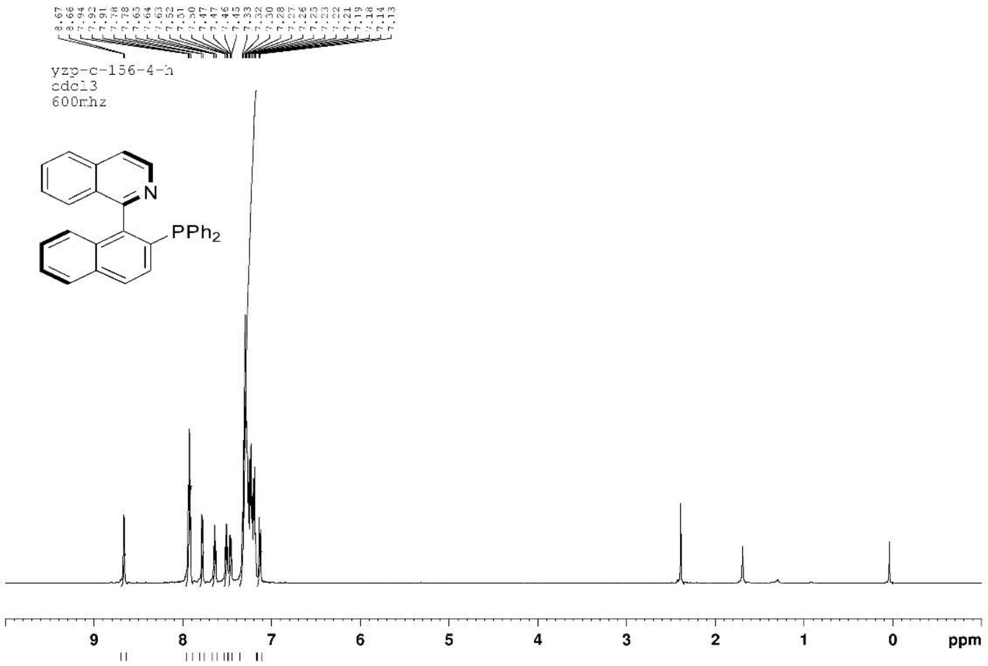

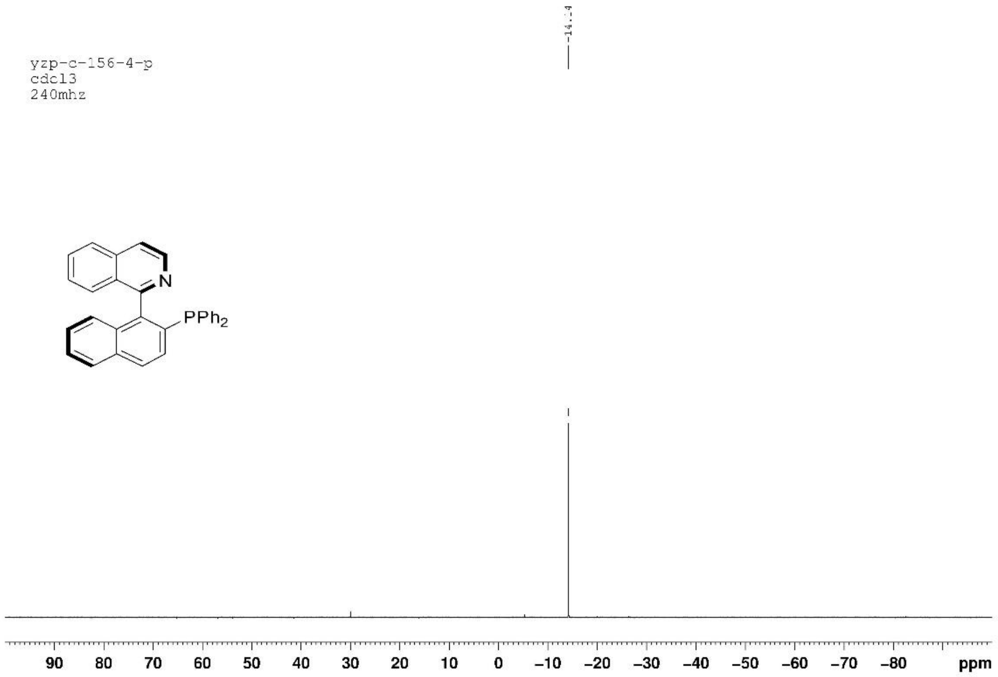

(*R*)-1-(2-(Diphenylphosphaneyl)naphthalen-1-yl)-4-phenylisoquinoline (3ha’)

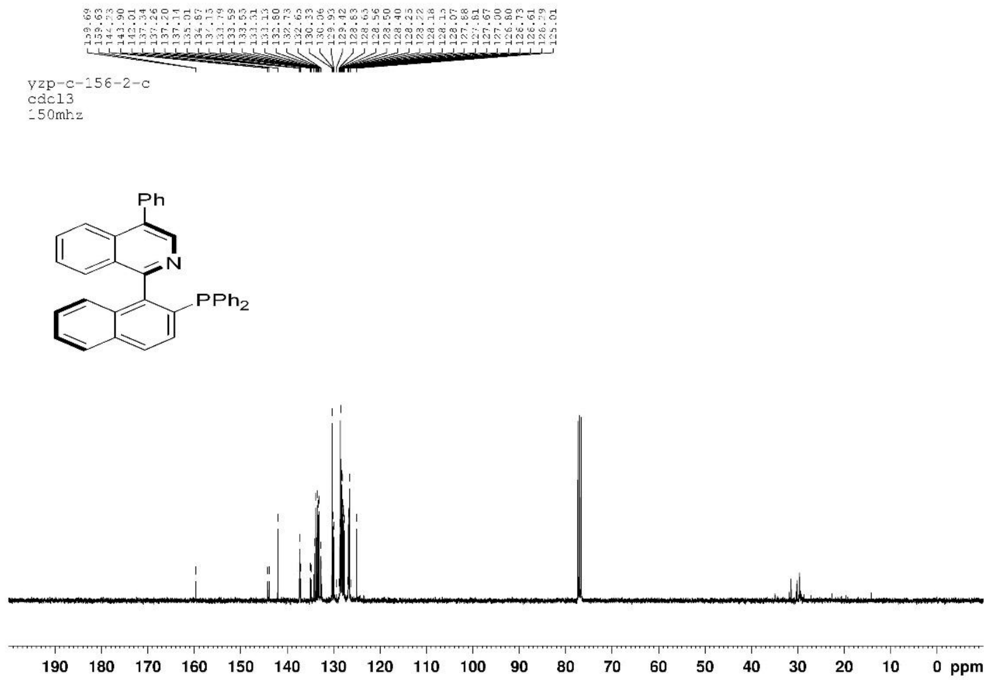

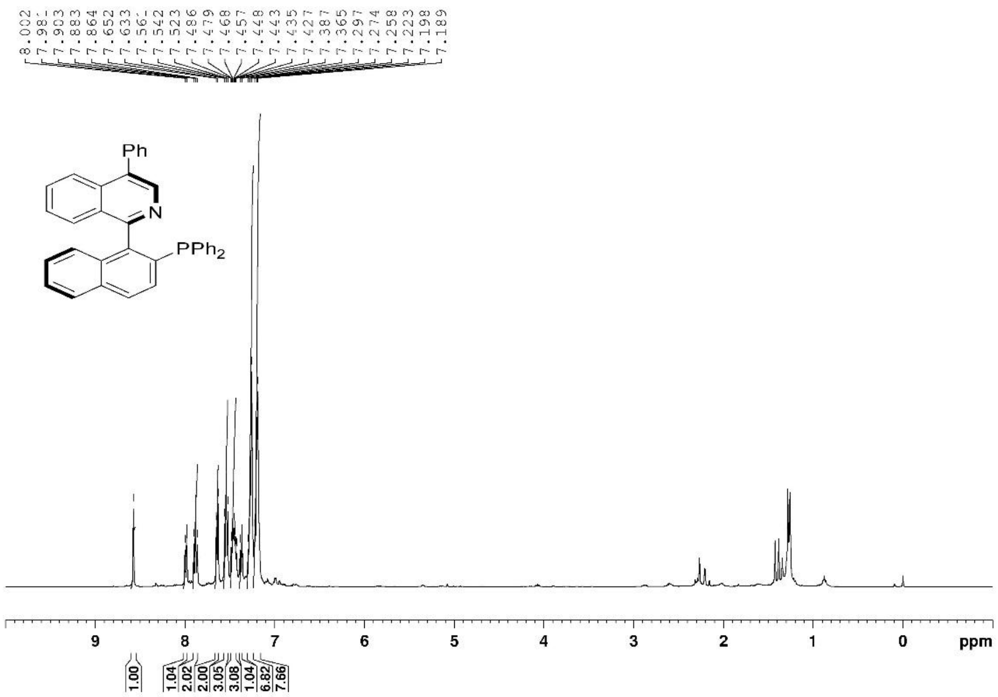

8.002
7.98-
7.903
7.883
7.864
7.652
7.633
7.56-
7.542
7.523
7.486
7.479
7.468
7.457
7.448
7.443
7.435
7.427
7.387
7.365
7.297
7.274
7.258
7.223
7.198
7.189
Ph
N
PPh2
9
8
7
6
5
4
3
2
1
0
ppm
1.00
1.04
2.02
2.00
3.05
3.08
1.04
6.82
7.66

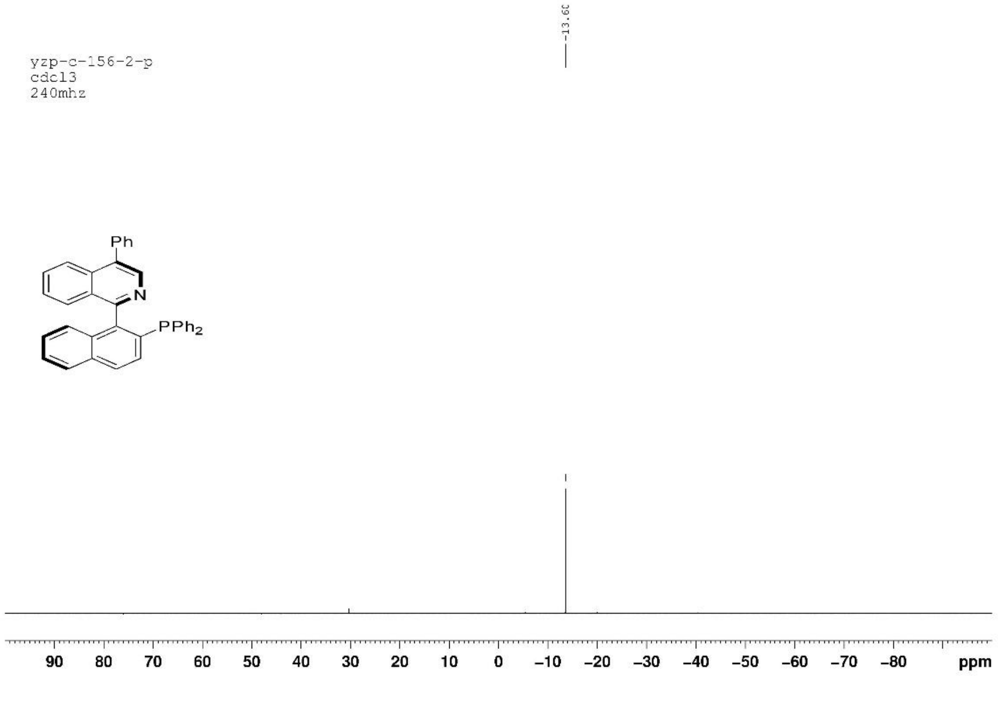

-13.60
yzp-c-156-2-p
cdcl3
240mhz
Ph
N
PPh2
90
80
70
60
50
40
30
20
10
0
−10
−20
−30
−40
−50
−60
−70
−80
ppm

(*R*)-1-(2-(Diphenylphosphaneyl)-6-phenylnaphthalen-1-yl)-6-phenylisoquinoline (3za')

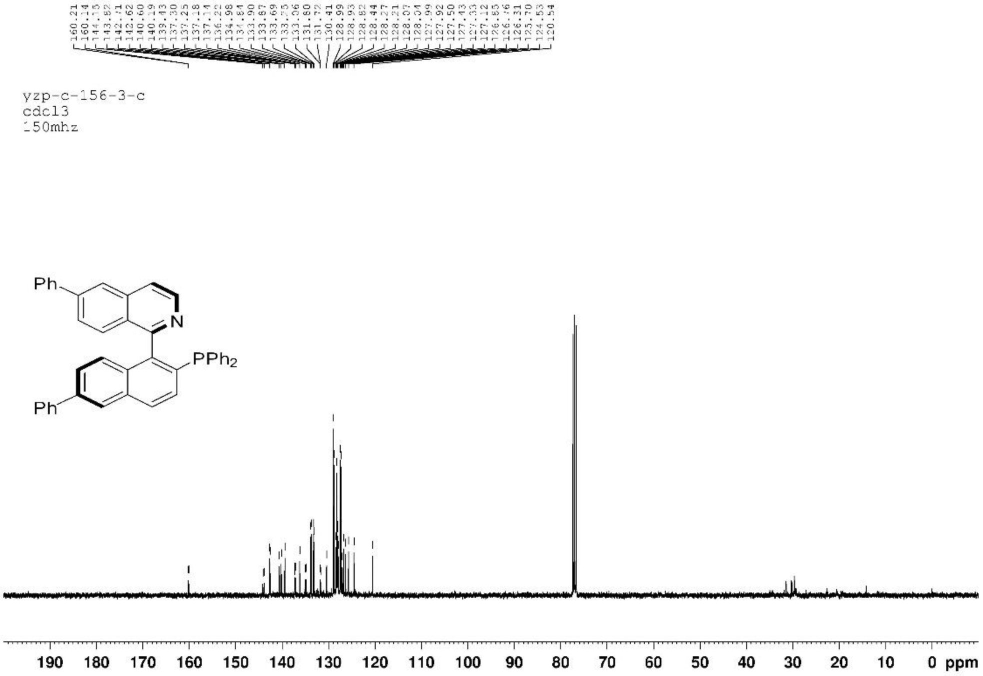

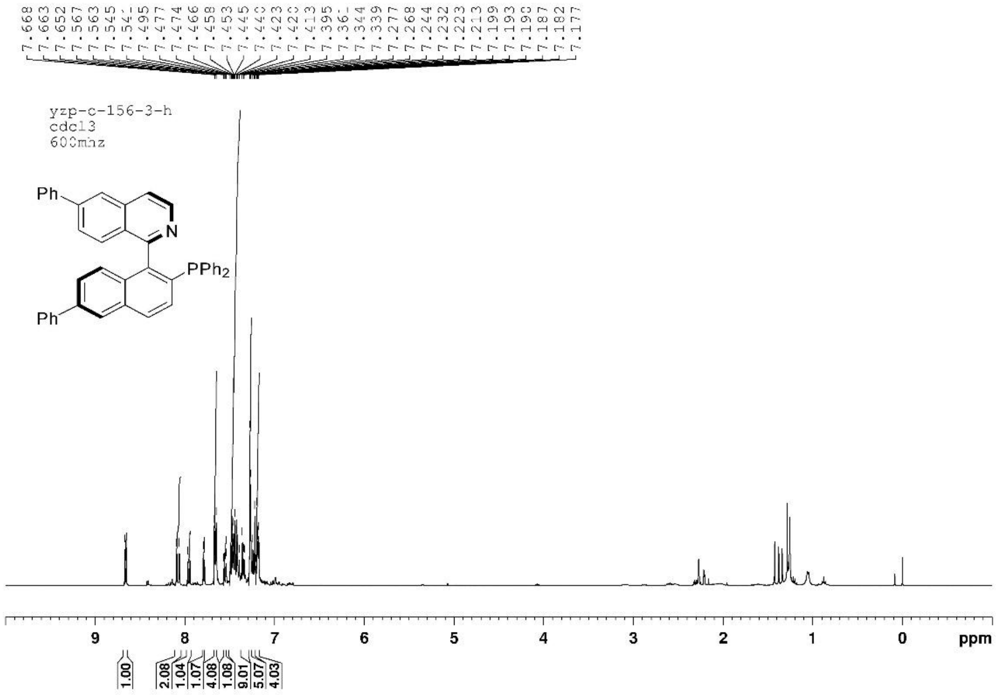

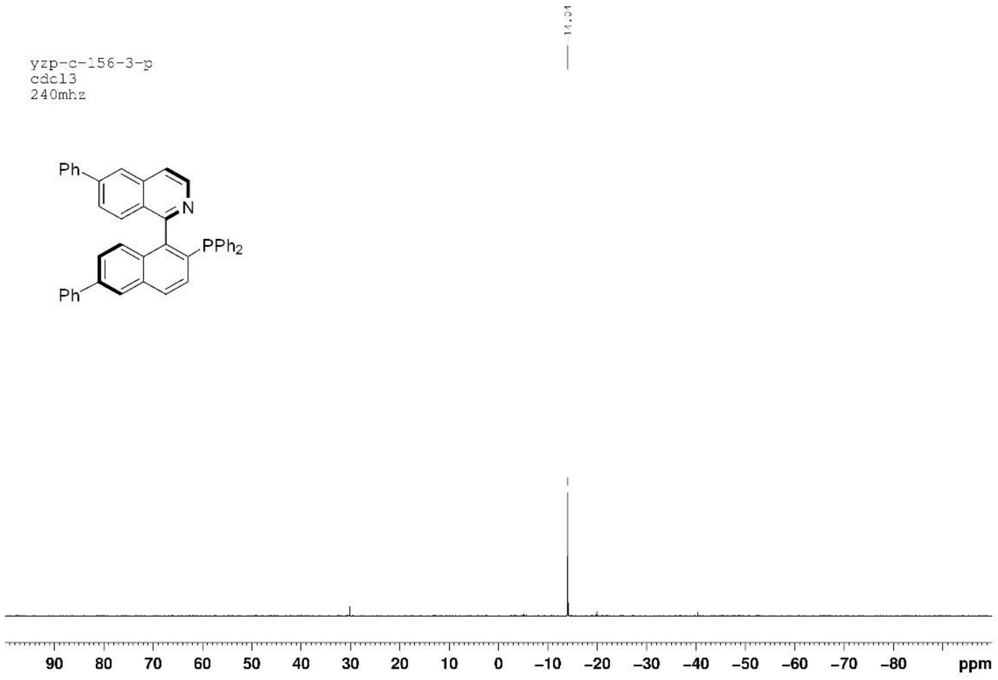

(*R*)-1-(2-(Diphenylphosphaneyl)-7-phenylnaphthalen-1-yl)-7-phenylisoquinoline (3aaa')

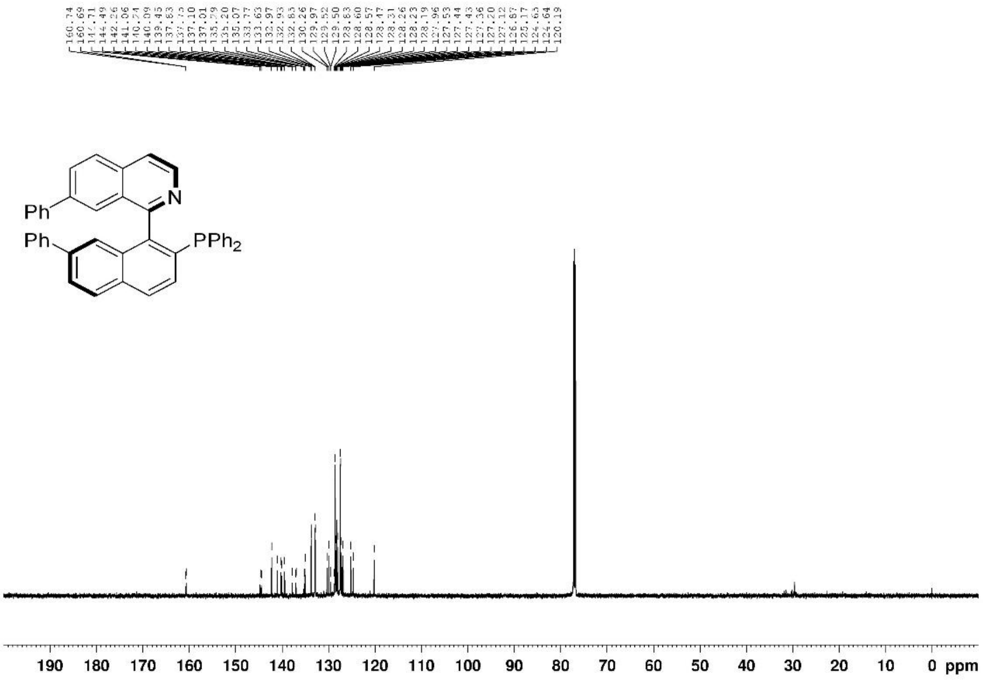

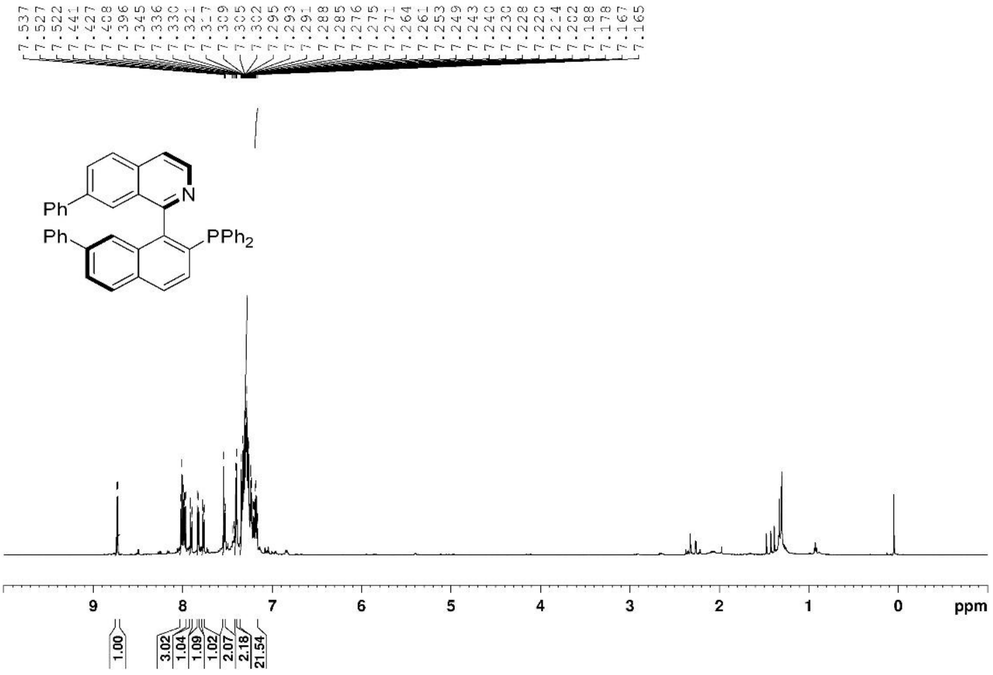
Ph
Ph
N
PPh2
9
8
7
6
5
4
3
2
1
0
ppm
1.00
3.02
1.04
1.09
1.02
2.07
2.18
21.54

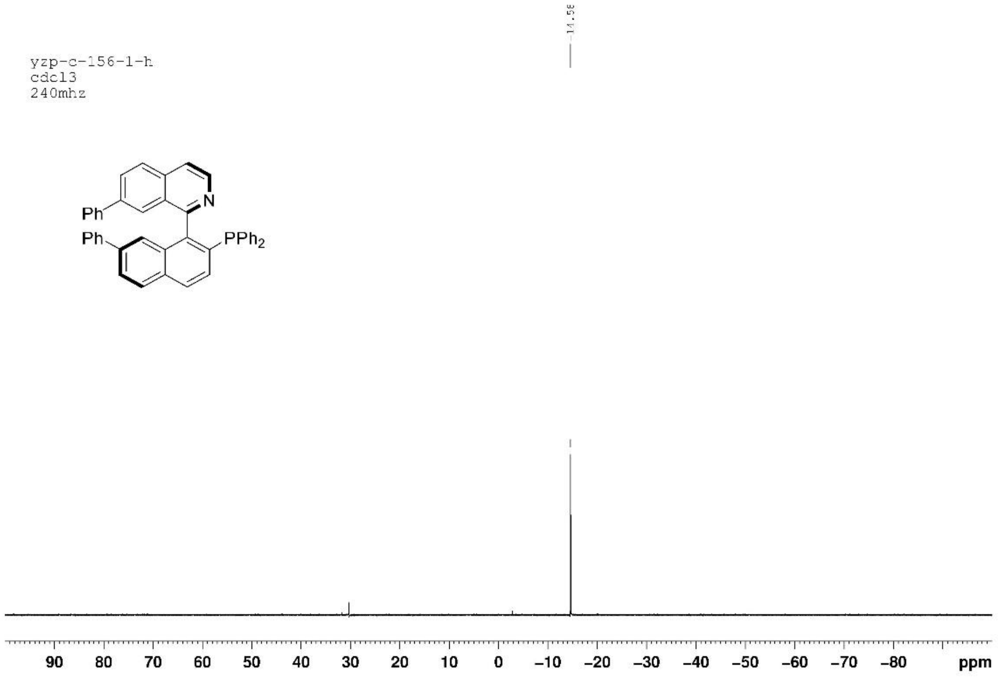
yzp-c-156-1-h
cdcl3
240mhz
Ph
Ph
N
PPh2
90
80
70
60
50
40
30
20
10
0
−10
−20
−30
−40
−50
−60
−70
−80
ppm

Dimethyl (*S*,*E*)-2-(1,3-diphenylallyl)malonate (6)

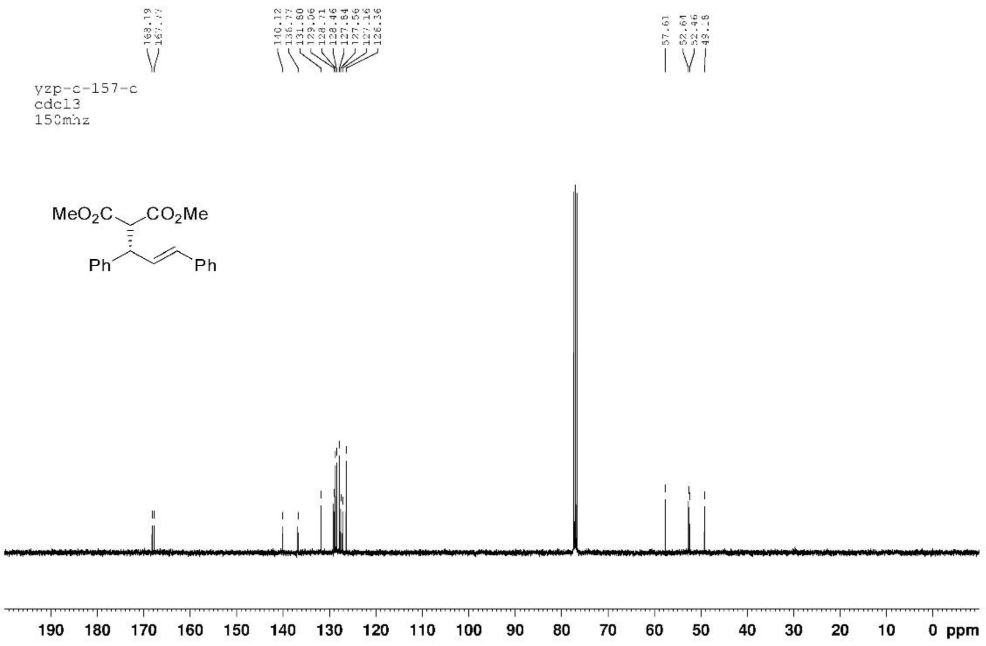

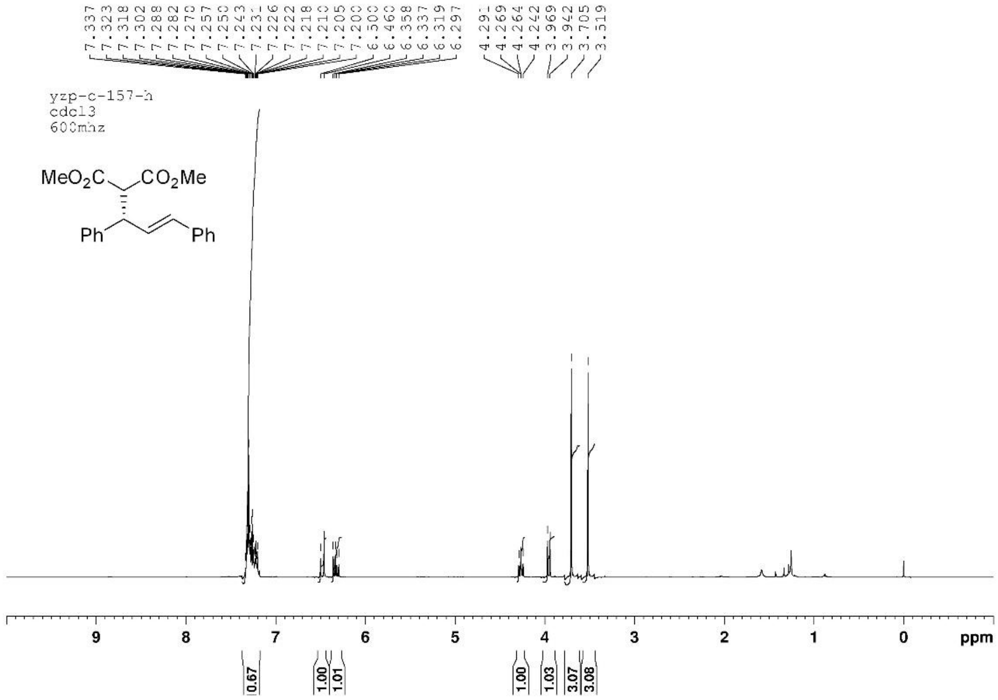

(*R*)-2-(Phenylethynyl)chroman-4-one (9)

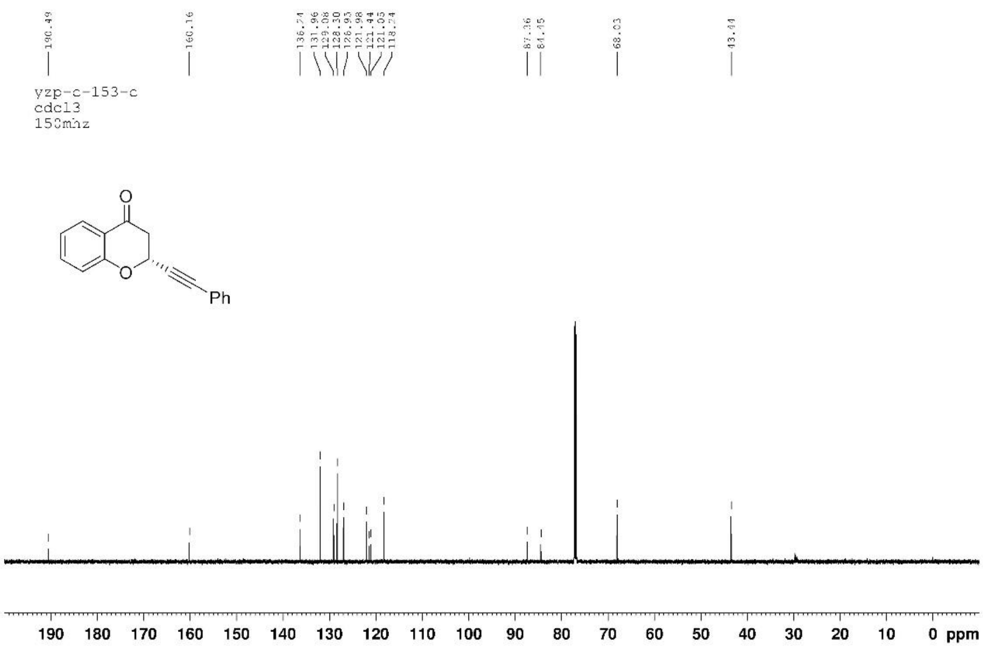

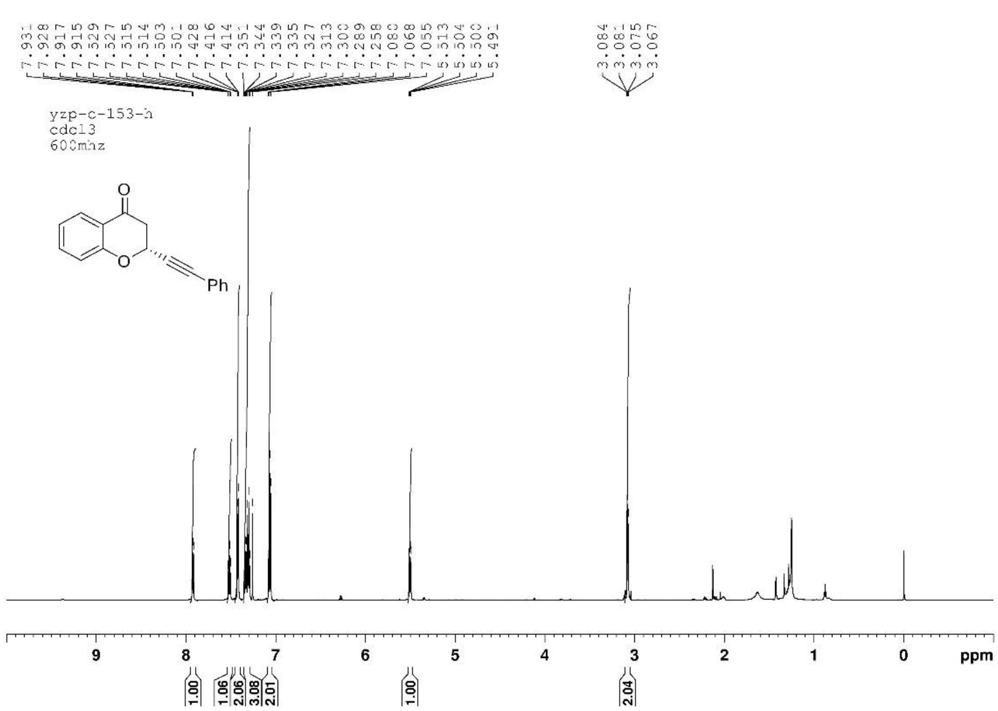

(*R*)-1,1'-Binaphthalen]-2-yldiphenylphosphine oxide (12)

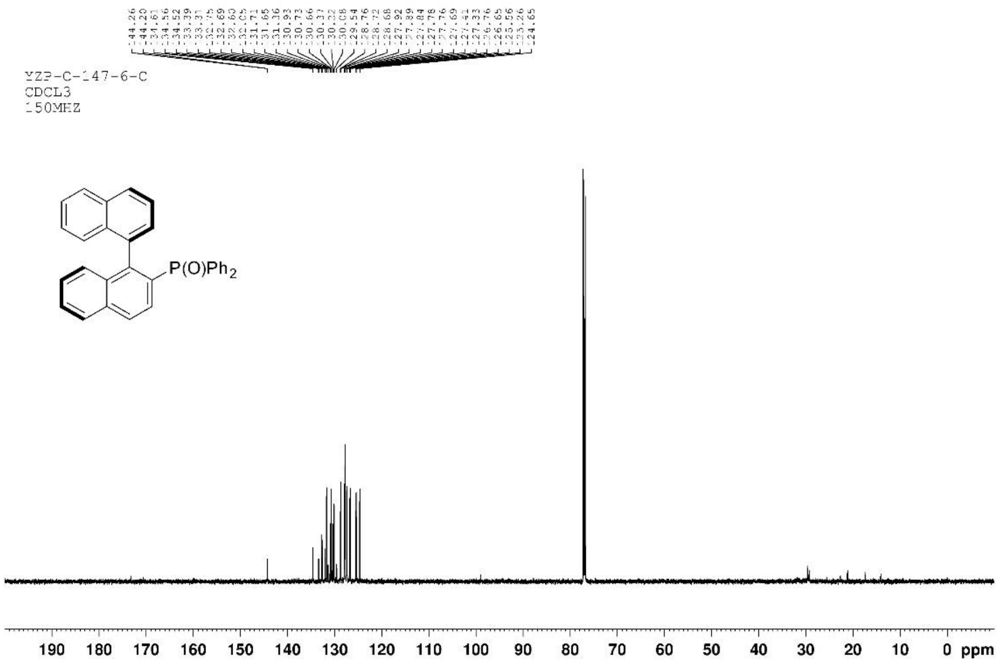

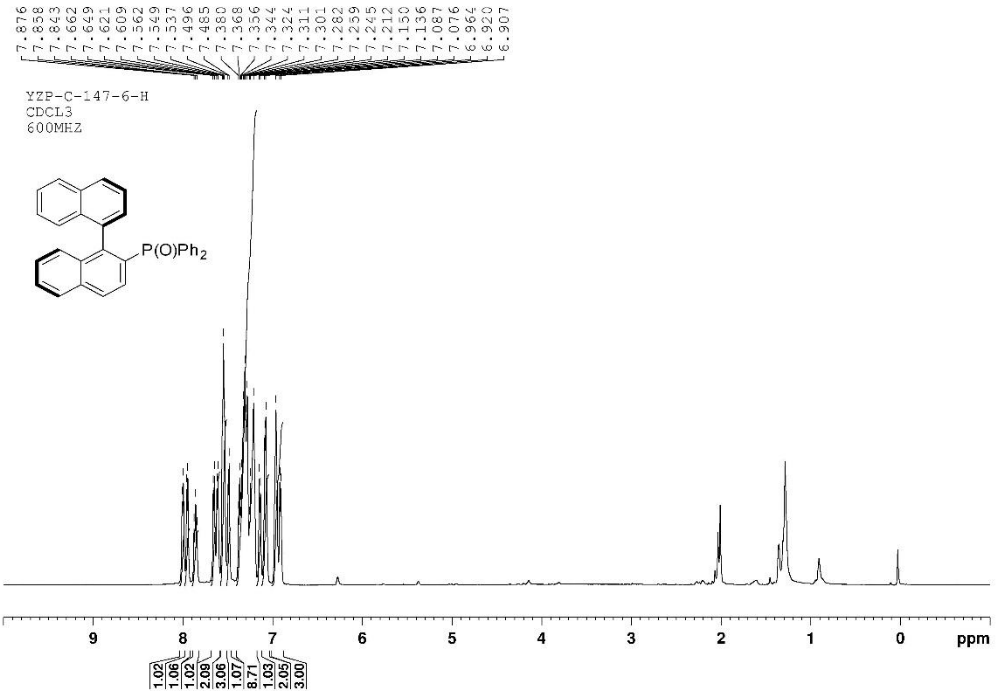

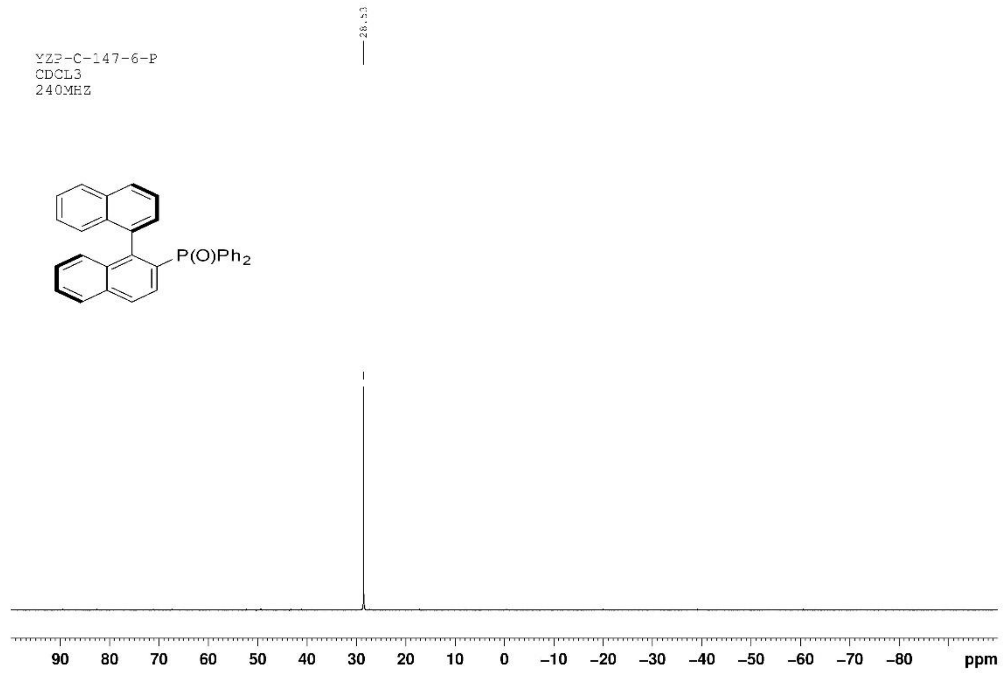

(*R*)-(1-(3-Methylpyridin-2-yl)naphthalen-2-yl)diphenylphosphine oxide (13)

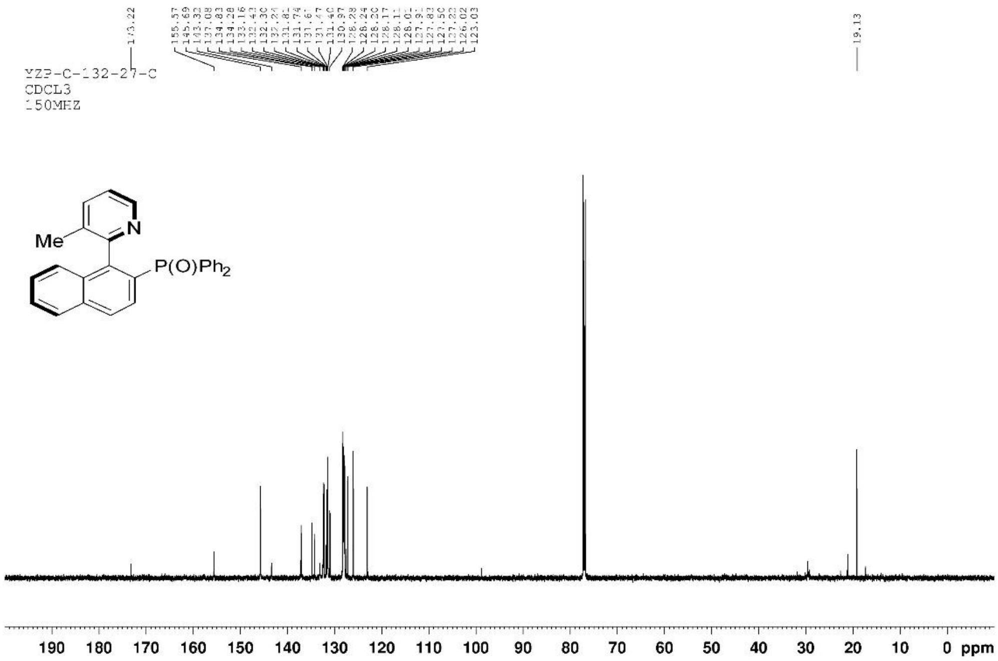

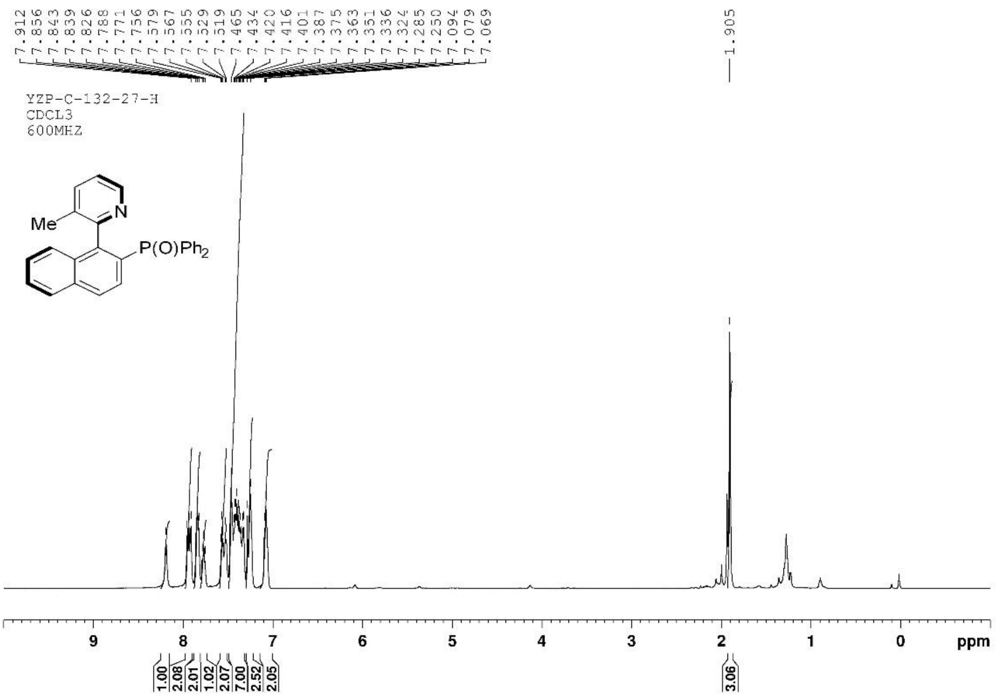
7.912
7.856
7.843
7.839
7.826
7.788
7.771
7.756
7.579
7.567
7.555
7.529
7.519
7.465
7.434
7.420
7.416
7.401
7.387
7.375
7.363
7.351
7.336
7.324
7.285
7.250
7.094
7.079
7.069
1.905
YZP-C-132-27-H
CDCL3
600MHZ
Me
N
P(O)Ph2
9
8
7
6
5
4
3
2
1
0
ppm
1.00
2.08
2.01
1.02
2.07
7.00
2.52
2.05
3.06

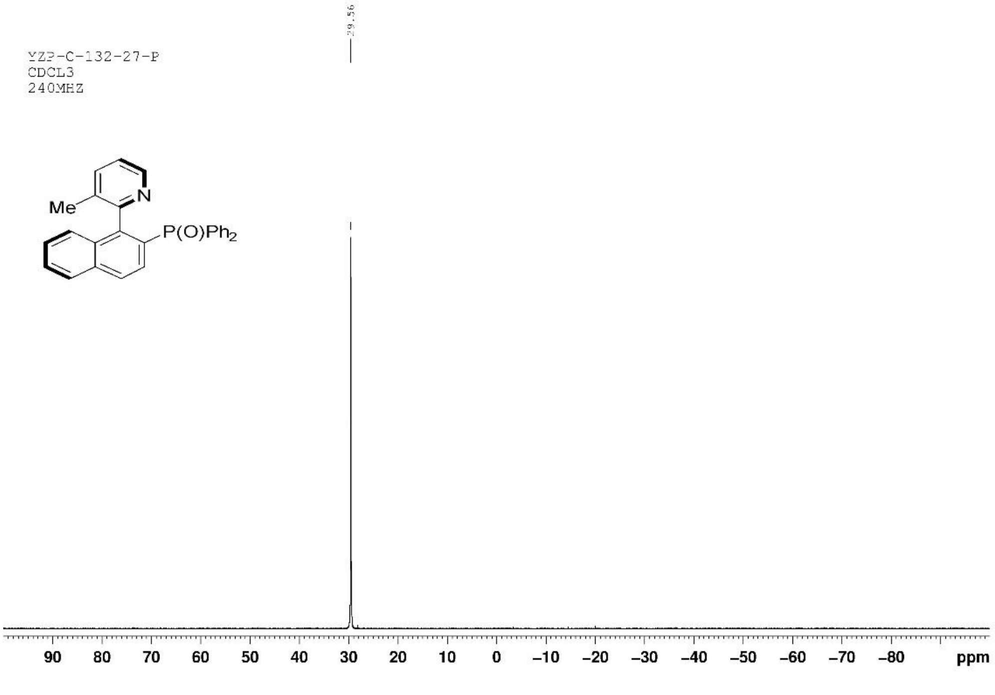
29.56
YZP-C-132-27-P
CDCL3
240MHZ
Me
N
P(O)Ph2
90
80
70
60
50
40
30
20
10
0
–10
–20
–30
–40
–50
–60
–70
–80
ppm

1-(Naphthalen-1-yl)isoquinoline (14)

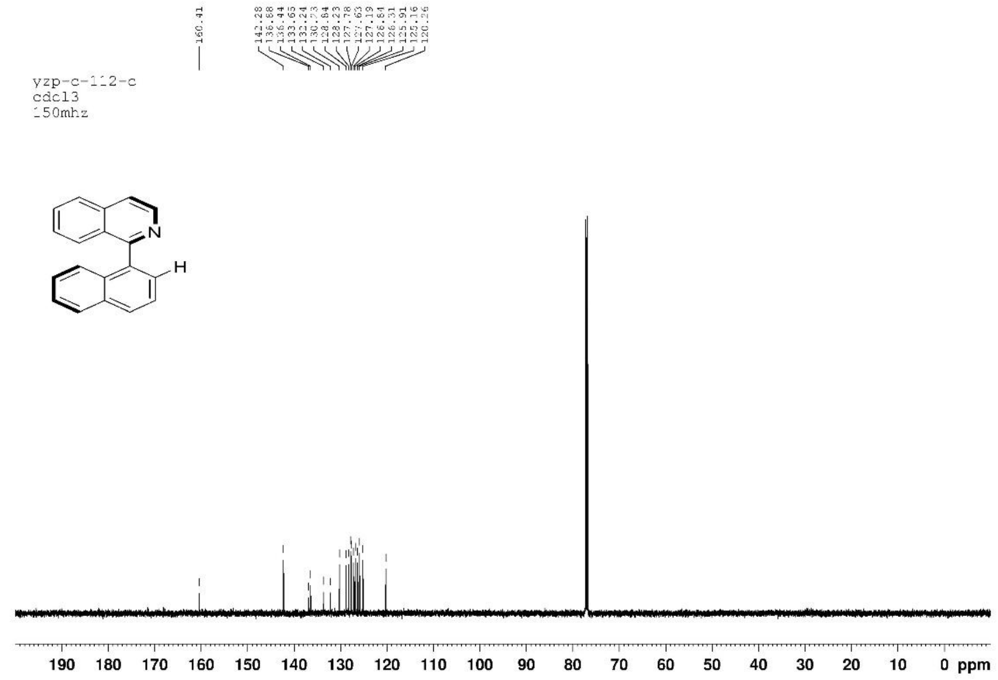

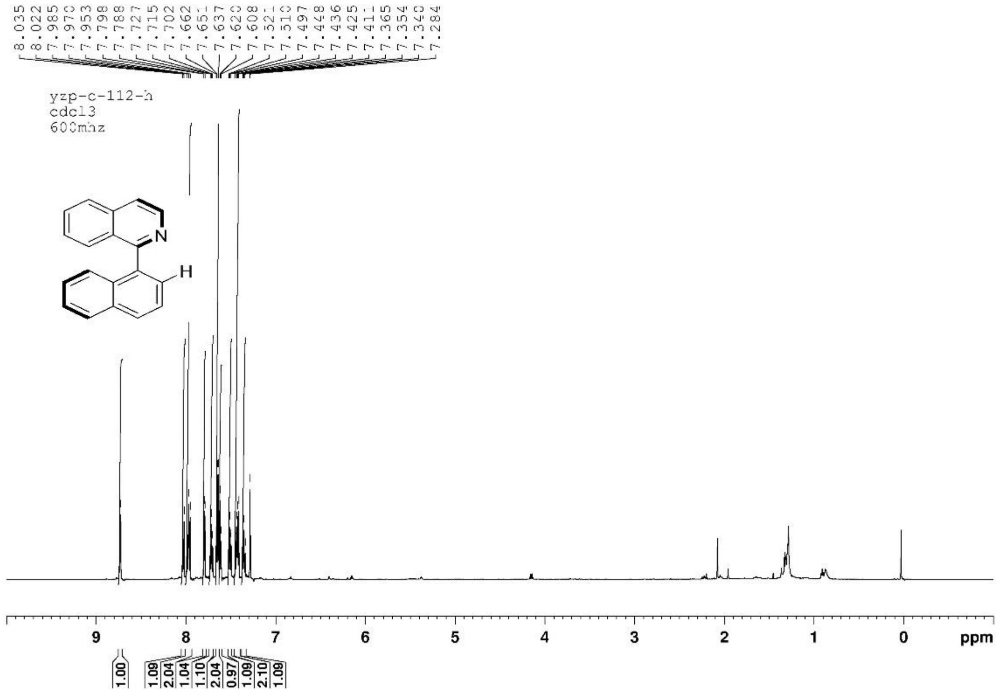

9. HPLC spectrum

(*R*)-(1-(Isoquinolin-1-yl)naphthalen-2-yl)diphenylphosphine oxide (3aa)

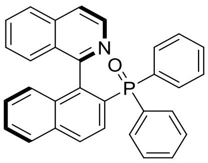

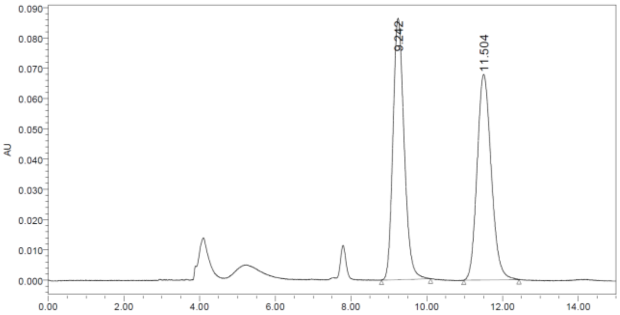

	RT	Area	% Area	Height
1	9.242	1756342	50.05	86300
2	11.504	1752637	49.95	67892

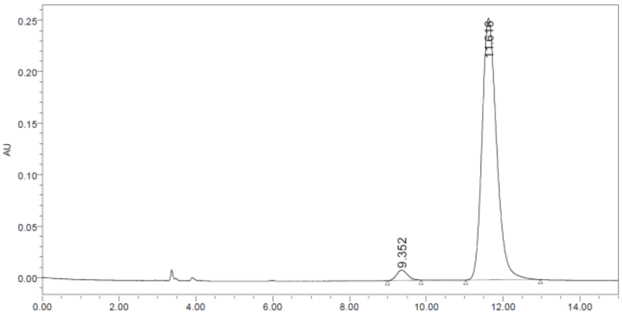

	RT	Area	% Area	Height
1	9.352	200895	2.91	9992
2	11.616	6707736	97.09	254230

(*R*)-(1-(Isoquinolin-1-yl)-6-methylnaphthalen-2-yl)diphenylphosphine oxide (3ba)

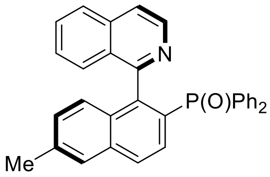

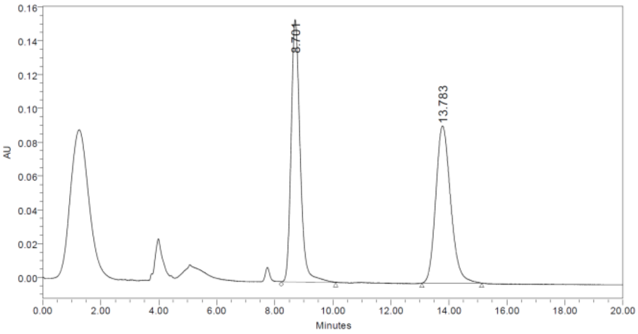

	RT	Area	% Area	Height
1	8.701	3346669	50.23	155026
2	13.783	3316533	49.77	92908

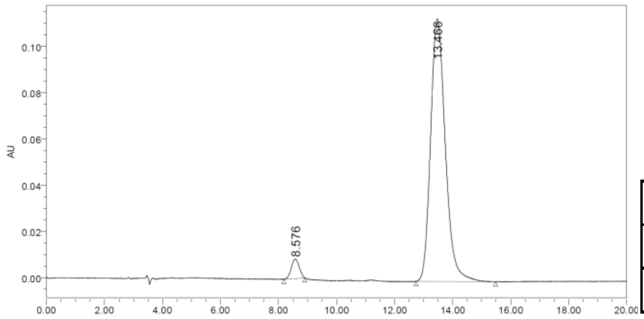

	RT	Area	% Area	Height
1	8.576	159496	3.83	8434
2	13.466	4010344	96.17	113686

(*R*)- (1-(Isoquinolin-1-yl)-7-methylnaphthalen-2-yl)diphenylphosphine oxide (3ca)

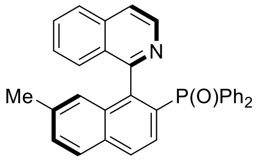

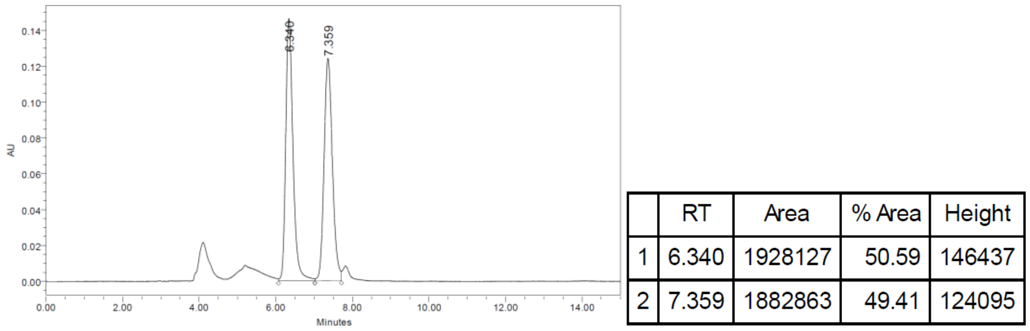

	RT	Area	% Area	Height
1	6.340	1928127	50.59	146437
2	7.359	1882863	49.41	124095

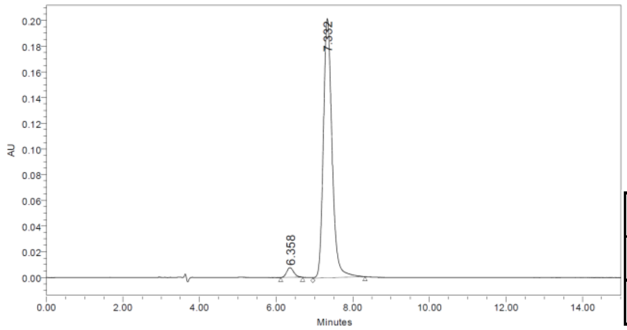

	RT	Area	% Area	Height
1	6.358	99729	3.04	7638
2	7.332	3176781	96.96	201884

(*R*)-(1-(Isoquinolin-1-yl)-7-methoxynaphthalen-2-yl)diphenylphosphine oxide (3da)

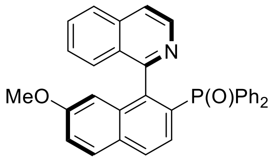

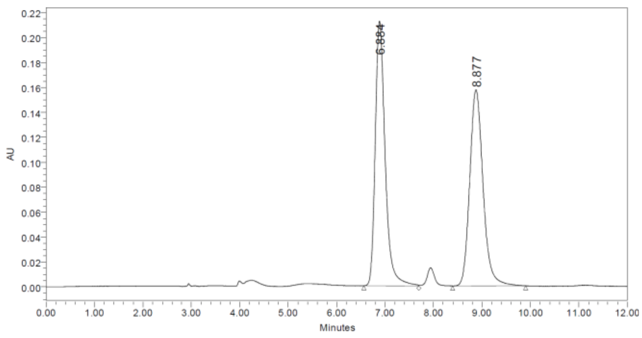

	RT	Area	% Area	Height
1	6.884	3008747	50.04	212280
2	8.877	3004187	49.96	157309

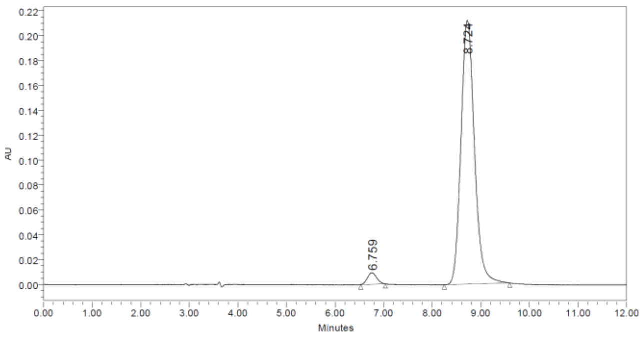

	RT	Area	% Area	Height
1	6.759	122843	2.92	9224
2	8.724	4086458	97.08	212099

(*R*)-(1-(Isoquinolin-1-yl)-6-methoxynaphthalen-2-yl)diphenylphosphine oxide (3ea)

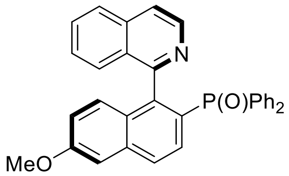

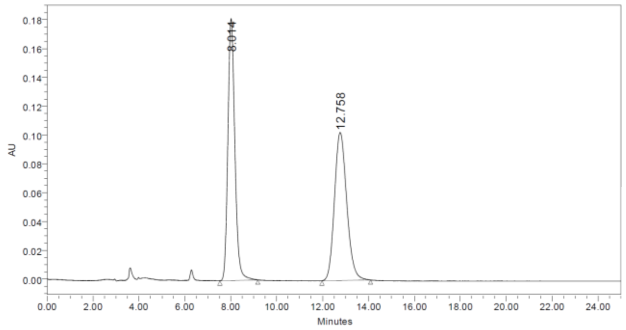

	RT	Area	% Area	Height
1	8.014	3830444	50.11	181891
2	12.758	3813672	49.89	102894

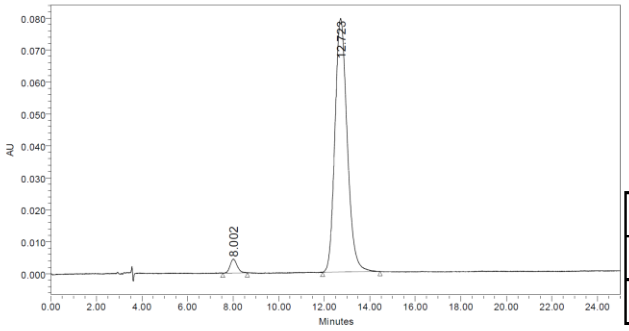

	RT	Area	% Area	Height
1	8.002	94207	3.08	4381
2	12.723	2960049	96.92	79340

(*R*)-Methyl 6-(diphenylphosphoryl)-5-(isoquinolin-1-yl)-2-naphthoate (3fa)

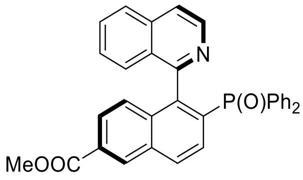

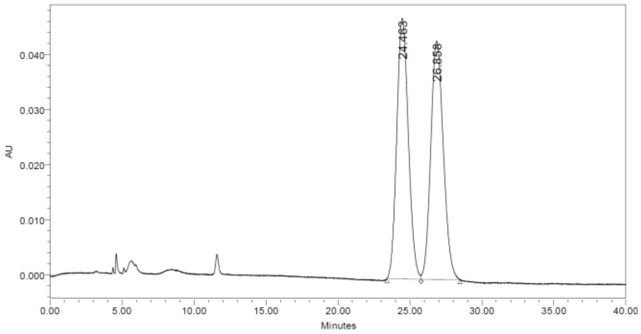

	RT	Area	% Area	Height
1	24.463	2633734	50.13	47303
2	26.858	2620258	49.87	43335

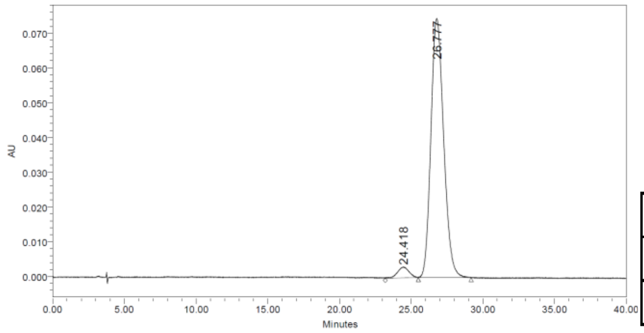

	RT	Area	% Area	Height
1	24.418	174293	3.67	3157
2	26.777	4575404	96.33	74554

(*R*)-(1-(6-Isopropylisoquinolin-1-yl)naphthalen-2-yl)diphenylphosphine oxide (3ga)

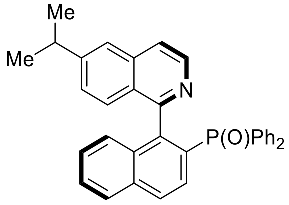

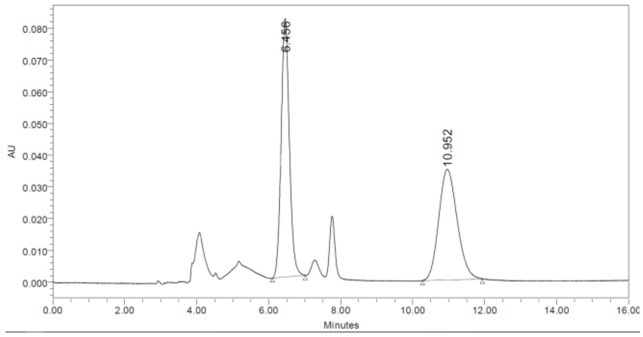

	RT	Area	% Area	Height
1	6.456	1288170	50.94	81356
2	10.952	1240394	49.06	34820

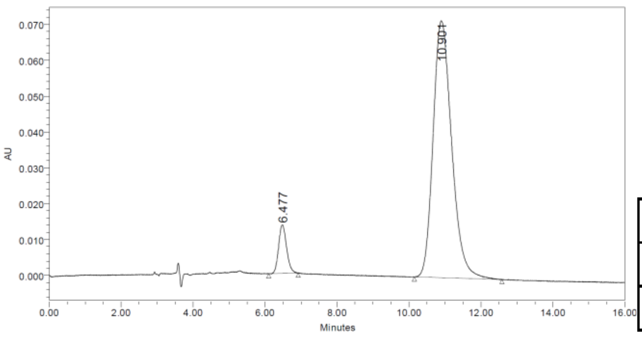

	RT	Area	% Area	Height
1	6.477	209565	7.53	13514
2	10.901	2572292	92.47	71671

(*R*)-Diphenyl(1-(4-phenylisoquinolin-1-yl)naphthalen-2-yl)phosphine oxide (3ha)

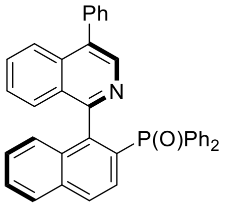

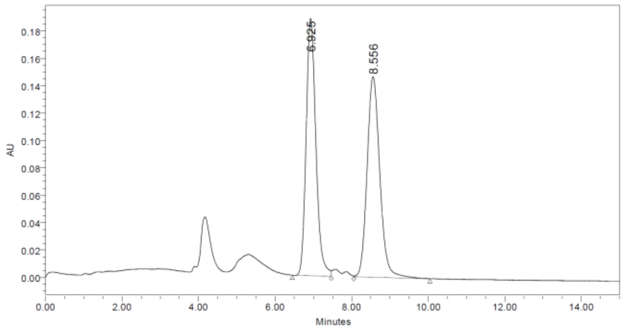

	RT	Area	% Area	Height
1	6.925	3309382	49.43	187712
2	8.556	3385797	50.57	146463

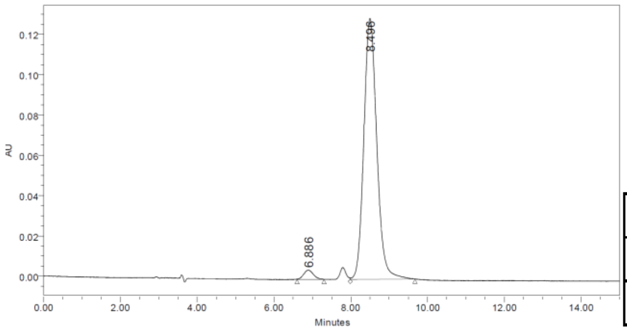

	RT	Area	% Area	Height
1	6.886	78285	2.52	4580
2	8.496	3034305	97.48	129376

(*R*)-Diphenyl(1-(5-phenylisoquinolin-1-yl)naphthalen-2-yl)phosphine oxide (3ia)

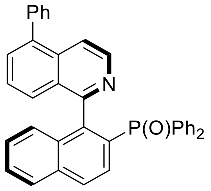

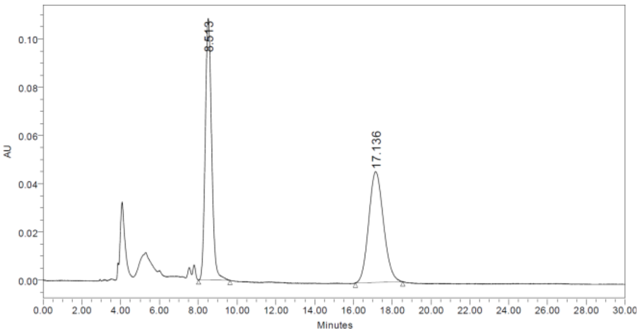

	RT	Area	% Area	Height
1	8.513	2477272	50.44	108448
2	17.136	2434228	49.56	46004

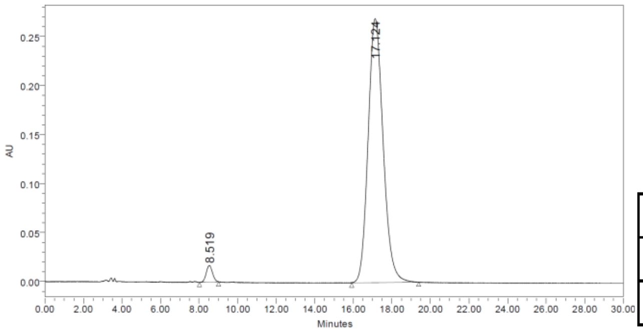

	RT	Area	% Area	Height
1	8.519	377376	2.52	16923
2	17.124	14571325	97.48	269439

(*R*)-Diphenyl(1-(6-phenylisoquinolin-1-yl)naphthalen-2-yl)phosphine oxide (3ja)

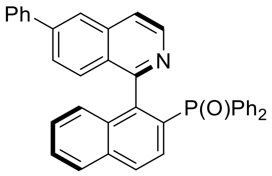

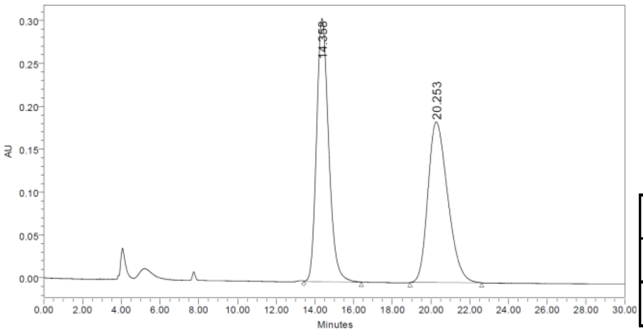

	RT	Area	% Area	Height
1	14.358	13389877	50.11	306907
2	20.253	13333074	49.89	187354

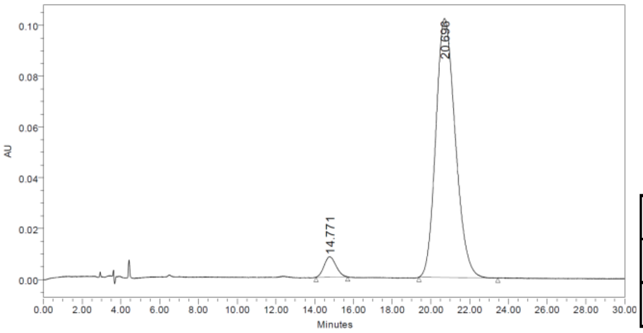

	RT	Area	% Area	Height
1	14.771	341878	4.58	8050
2	20.696	7130815	95.42	101984

(*R*)-Diphenyl(1-(7-phenylisoquinolin-1-yl)naphthalen-2-yl)phosphine oxide (3ka)

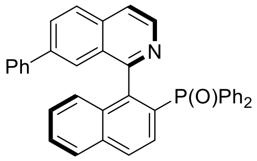

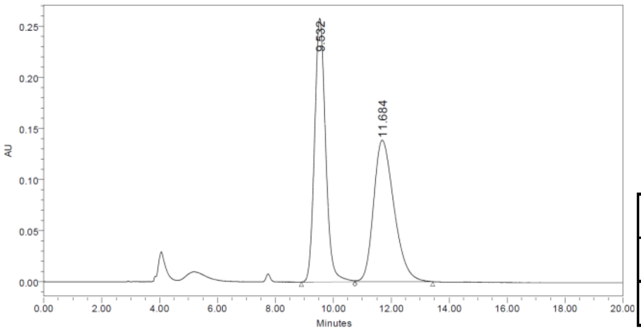

	RT	Area	% Area	Height
1	9.532	6833148	50.05	258064
2	11.684	6820680	49.95	138657

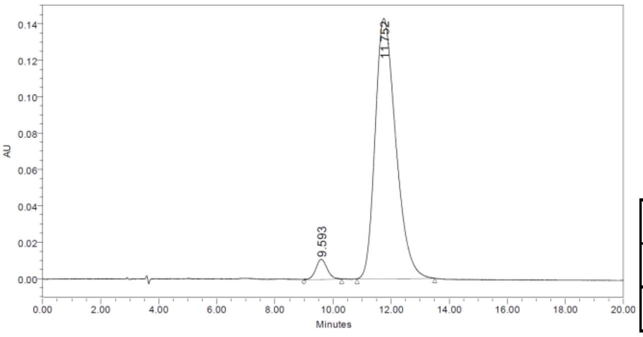

	RT	Area	% Area	Height
1	9.593	300180	4.06	11034
2	11.752	7091639	95.94	143032

(*R*)-(1-(Isoquinolin-1-yl)-7-phenylnaphthalen-2-yl)diphenylphosphine oxide (3la)

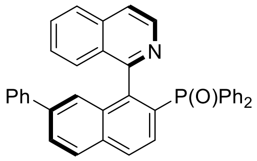

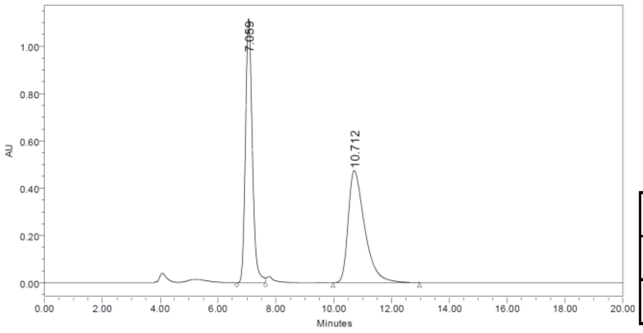

	RT	Area	% Area	Height
1	7.059	18409015	49.51	1117219
2	10.712	18776223	50.49	474557

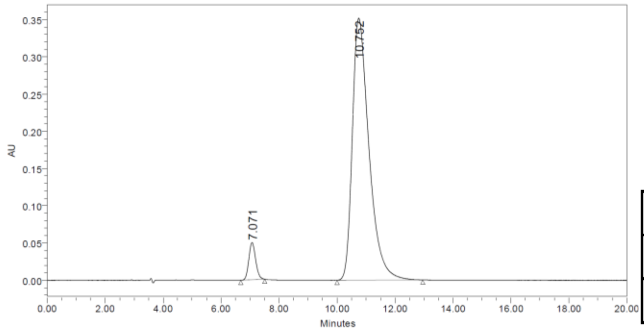

	RT	Area	% Area	Height
1	7.071	818150	5.51	49972
2	10.752	14027769	94.49	351823

(*R*)-(1-(Isoquinolin-1-yl)-6-phenylnaphthalen-2-yl)diphenylphosphine oxide (3ma)

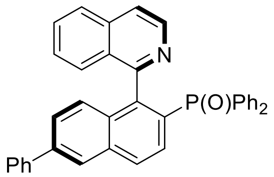

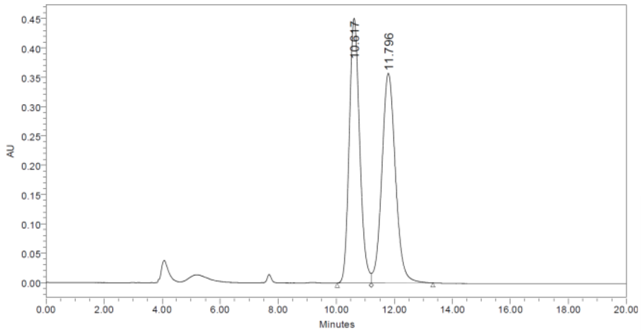

	RT	Area	% Area	Height
1	10.617	11270645	49.18	451627
2	11.796	11647257	50.82	358030

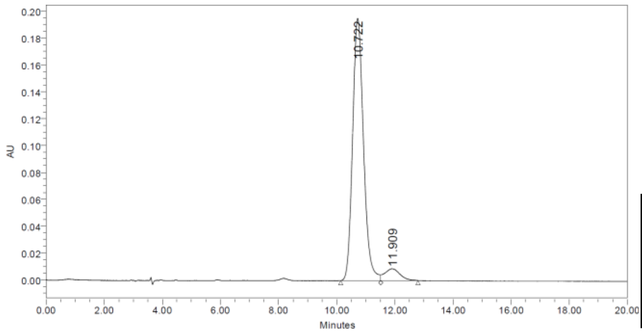

	RT	Area	% Area	Height
1	10.722	5072197	94.08	195120
2	11.909	318895	5.92	8805

(*R*)-(1-(Isoquinolin-1-yl)-4-phenylnaphthalen-2-yl)diphenylphosphine oxide (3na)

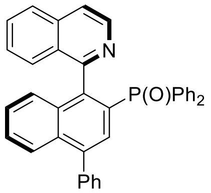

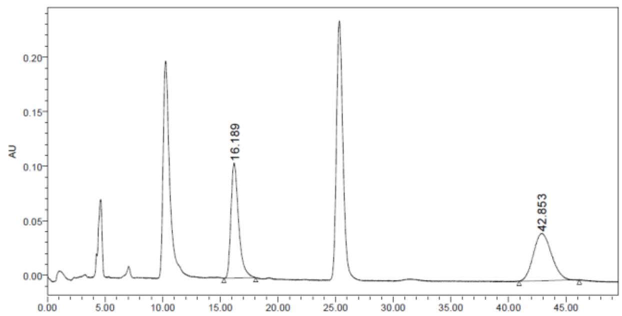

	RT	Area	% Area	Height
1	16.189	4890993	50.74	105523
2	42.853	4749229	49.26	43307

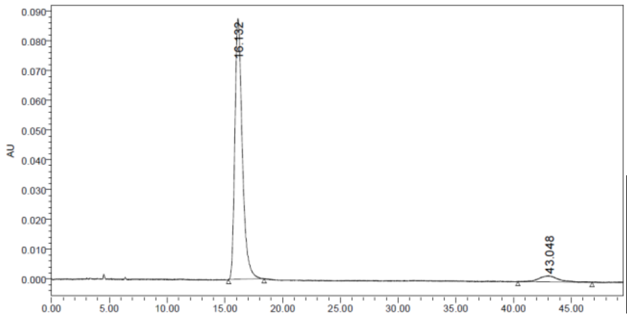

	RT	Area	% Area	Height
1	16.132	3886864	94.53	87545
2	43.048	224775	5.47	2005

(*R*)-(1-(Isoquinolin-1-yl)-6-(p-tolyl)naphthalen-2-yl)diphenylphosphine oxide (3oa)

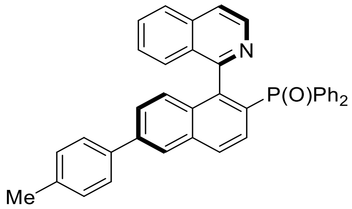

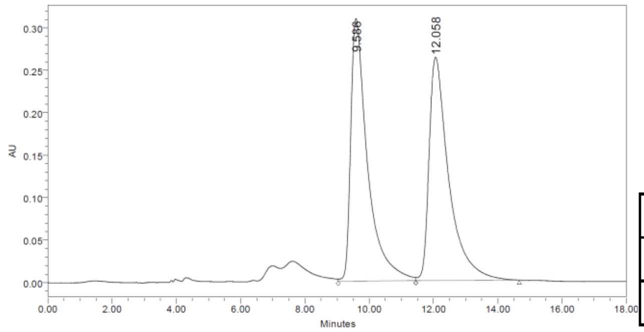

	RT	Area	% Area	Height
1	9.586	11258132	50.02	309181
2	12.058	11249987	49.98	263309

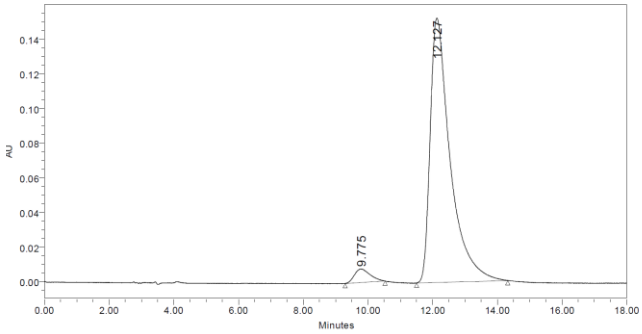

	RT	Area	% Area	Height
1	9.775	262592	3.85	7813
2	12.127	6555027	96.15	152671

(*R*)-(6-(3,5-Dimethylphenyl)-1-(isoquinolin-1-yl)naphthalen-2-yl)diphenylphosphine oxide (3pa)

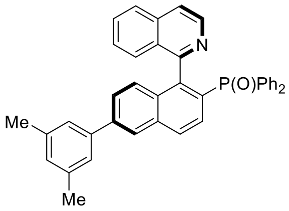

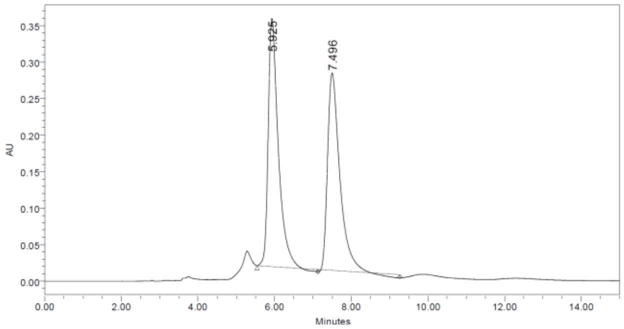

	RT	Area	% Area	Height
1	5.925	6288725	50.50	339789
2	7.496	6165356	49.50	270496

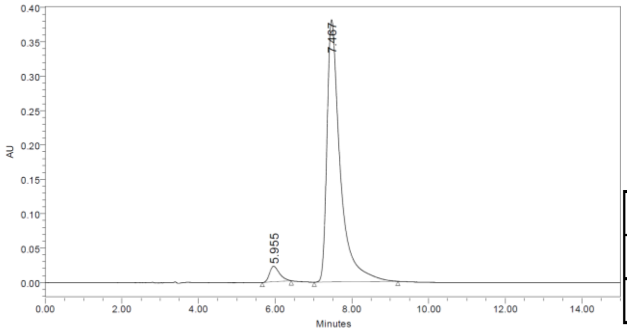

	RT	Area	% Area	Height
1	5.955	411093	4.30	22699
2	7.467	9140939	95.70	381446

(*R*)-(1-(Isoquinolin-1-yl)-6-(4-methoxyphenyl)naphthalen-2-yl)diphenylphosphine oxide (3qa)

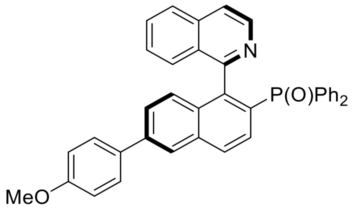

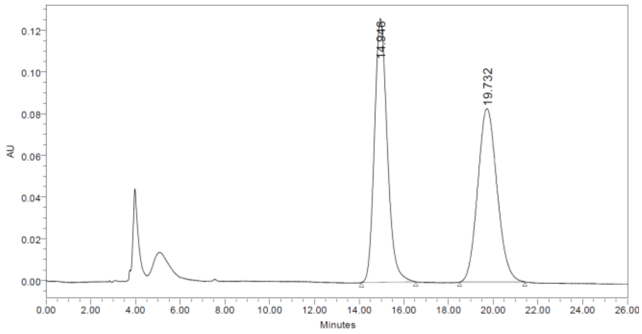

	RT	Area	% Area	Height
1	14.946	5054811	50.24	126554
2	19.732	5007420	49.76	83196

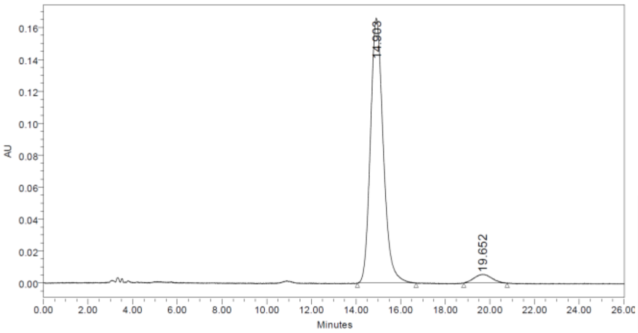

	RT	Area	% Area	Height
1	14.903	6627308	95.67	165905
2	19.652	299886	4.33	5427

(*R*)-(6-(4-Fluorophenyl)-1-(isoquinolin-1-yl)naphthalen-2-yl)diphenylphosphine oxide (3ra)

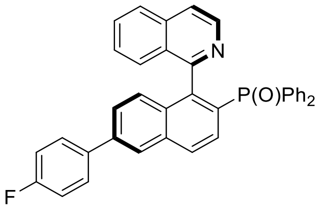

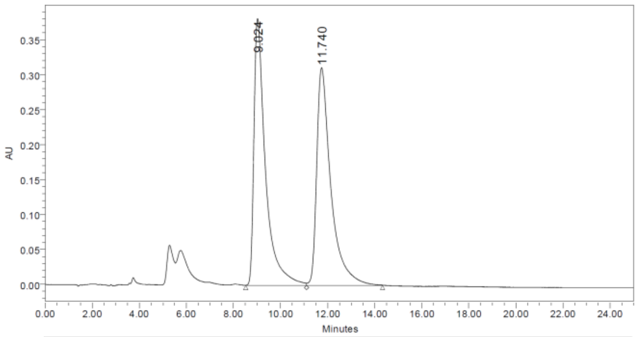

	RT	Area	% Area	Height
1	9.024	12961228	49.95	381516
2	11.740	12989242	50.05	311314

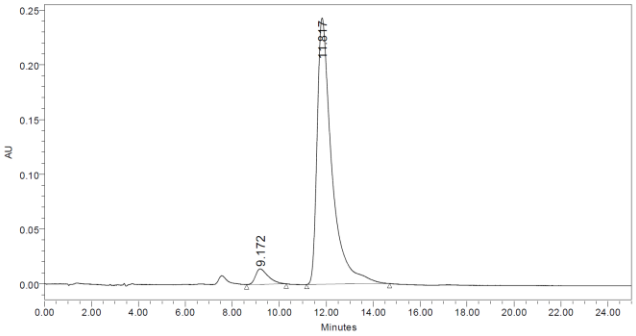

	RT	Area	% Area	Height
1	9.172	534341	4.83	14162
2	11.817	10531330	95.17	243239

(*R*)-(5-(Isoquinolin-1-yl)-[2,2'-binaphthalen]-6-yl)diphenylphosphine oxide (3sa)

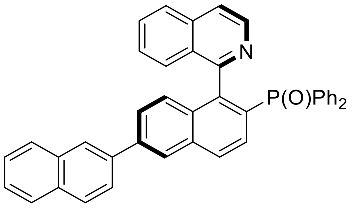

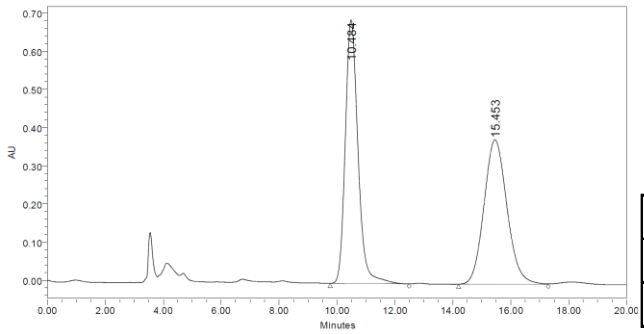

	RT	Area	% Area	Height
1	10.484	21043349	50.13	690388
2	15.453	20934790	49.87	378561

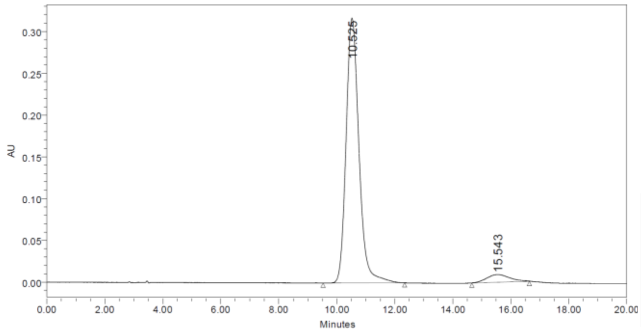

	RT	Area	% Area	Height
1	10.525	9778750	95.20	317186
2	15.543	492704	4.80	9154

(*R*)-(1-(Isoquinolin-1-yl)-6-(thiophen-2-yl)naphthalen-2-yl)diphenylphosphine oxide (3ta)

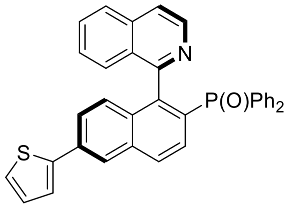

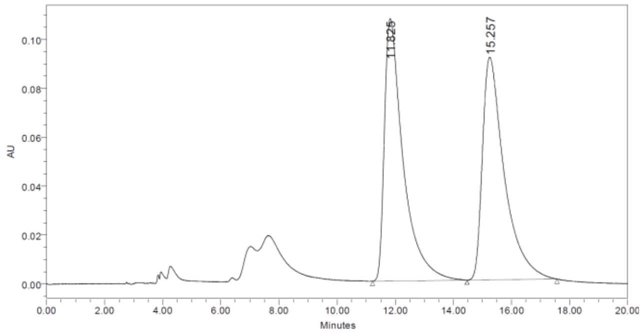

	RT	Area	% Area	Height
1	11.825	4825235	50.23	107469
2	15.257	4781557	49.77	91178

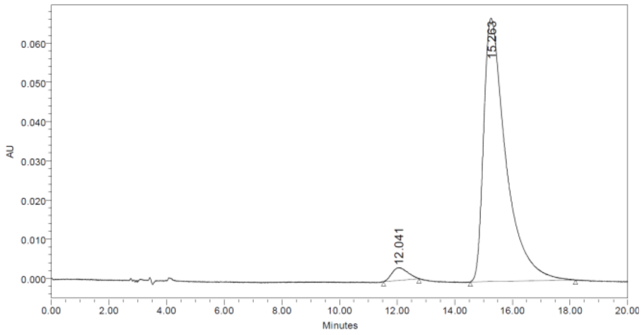

	RT	Area	% Area	Height
1	12.041	123796	3.34	3293
2	15.263	3583917	96.66	67042

(*R*)-Diphenyl(1-(6-(p-tolyl)isoquinolin-1-yl)naphthalen-2-yl)phosphine oxide (3ua)

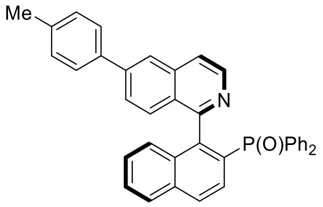

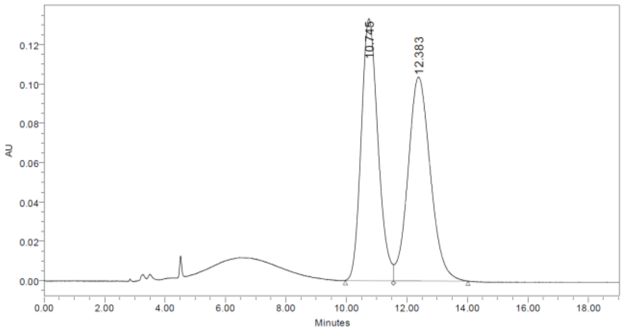

	RT	Area	% Area	Height
1	10.745	5116764	49.01	133214
2	12.383	5323313	50.99	103659

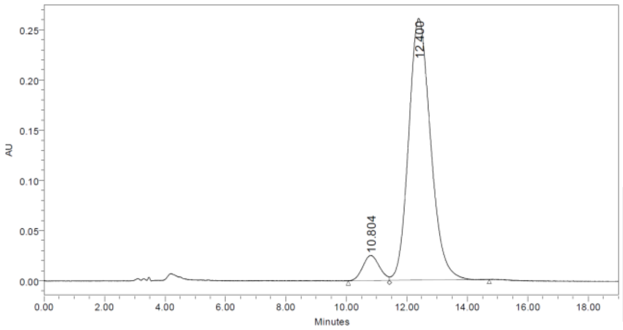

	RT	Area	% Area	Height
1	10.804	953889	6.76	25119
2	12.400	13161456	93.24	260656

(*R*)-(1-(6-(3,5-Dimethylphenyl)isoquinolin-1-yl)naphthalen-2-yl)diphenylphosphine oxide (3va)

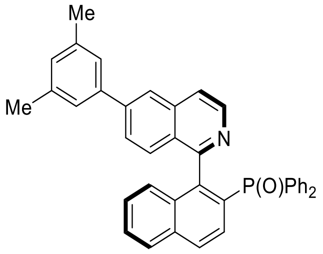

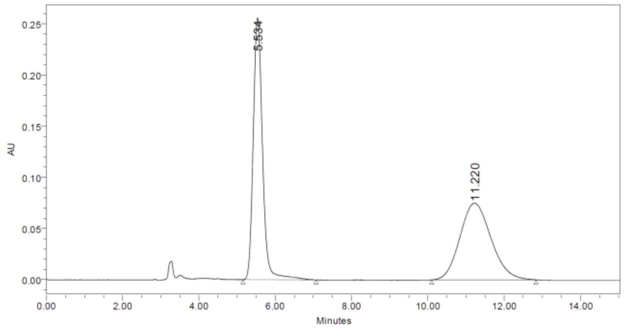

	RT	Area	% Area	Height
1	5.534	4328713	50.45	255802
2	11.220	4250727	49.55	75035

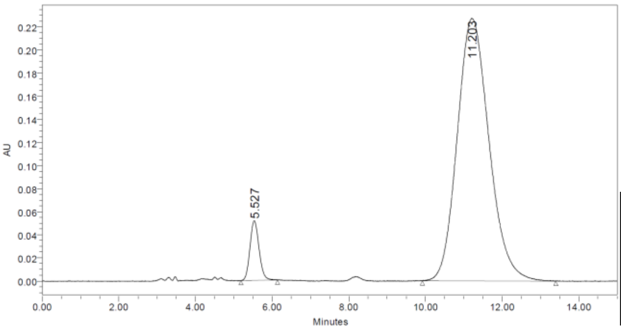

	RT	Area	% Area	Height
1	5.527	850031	6.08	51645
2	11.203	13122229	93.92	227352

(*R*)-(1-(6-(4-Methoxyphenyl)isoquinolin-1-yl)naphthalen-2-yl)diphenylphosphine oxide (3wa)

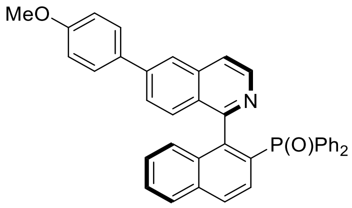

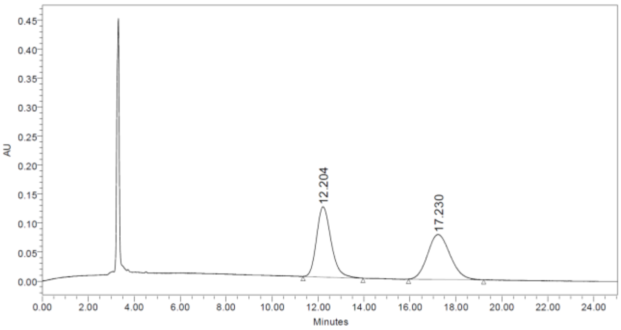

	RT	Area	% Area	Height
1	12.204	5339312	50.11	121305
2	17.230	5316714	49.89	77608

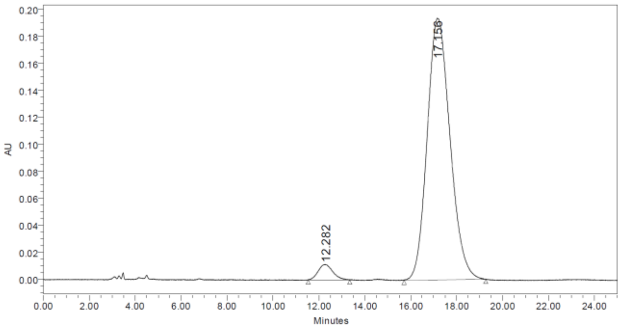

	RT	Area	% Area	Height
1	12.282	504480	3.63	11469
2	17.156	13410389	96.37	193779

(*R*)-(1-(6-(4-Fluorophenyl)isoquinolin-1-yl)naphthalen-2-yl)diphenylphosphine oxide (3xa)

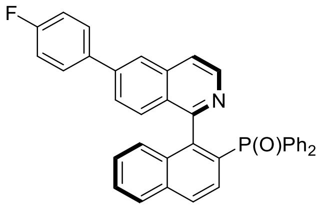

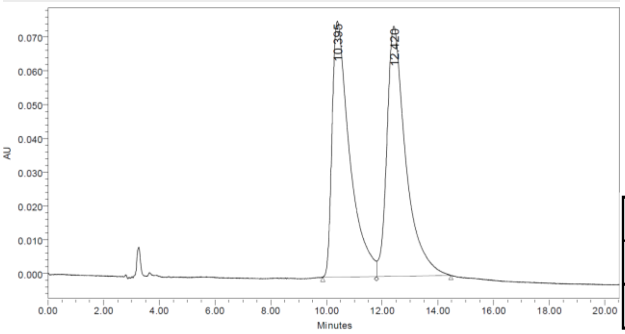

	RT	Area	% Area	Height
1	10.395	3326122	48.73	75805
2	12.420	3498998	51.27	74133

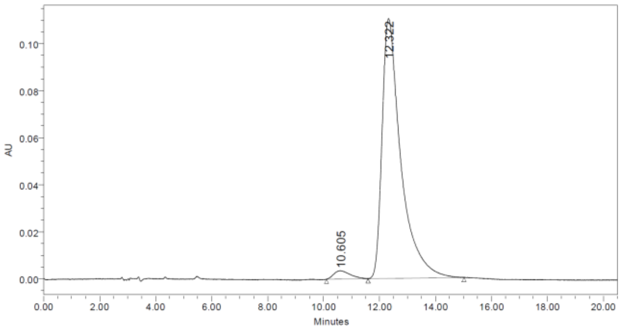

	RT	Area	% Area	Height
1	10.605	145749	2.76	3625
2	12.322	5143691	97.24	110519

(*R*)-Diphenyl(1-(6-(thiophen-2-yl)isoquinolin-1-yl)naphthalen-2-yl)phosphine oxide (3ya)

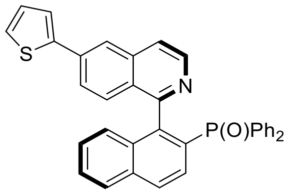

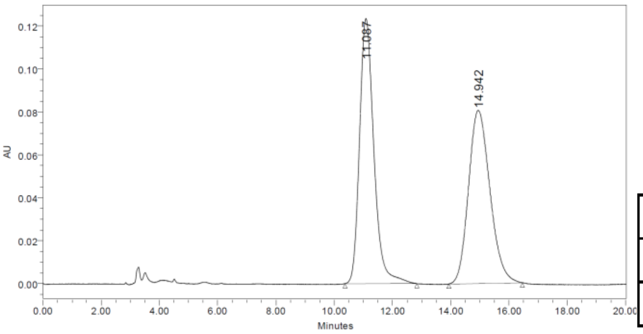

	RT	Area	% Area	Height
1	11.087	4232038	50.57	123612
2	14.942	4136986	49.43	80757

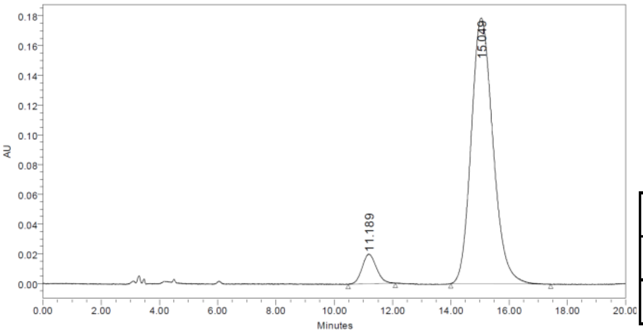

	RT	Area	% Area	Height
1	11.189	681177	6.80	20012
2	15.049	9332964	93.20	178830

(*R*)-Diphenyl(6-phenyl-1-(6-phenylisoquinolin-1-yl)naphthalen-2-yl)phosphine oxide (3za)

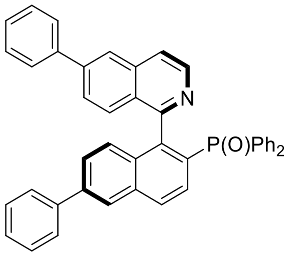

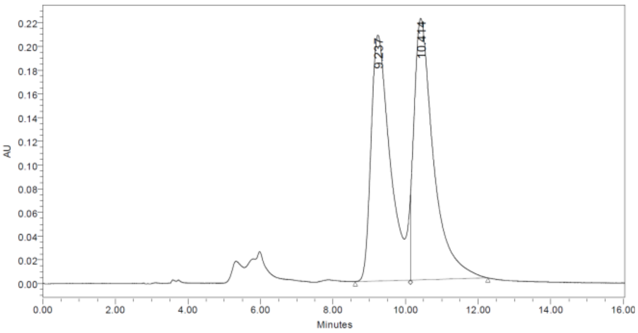

	RT	Area	% Area	Height
1	9.237	7758485	48.48	207328
2	10.414	8244836	51.52	220465

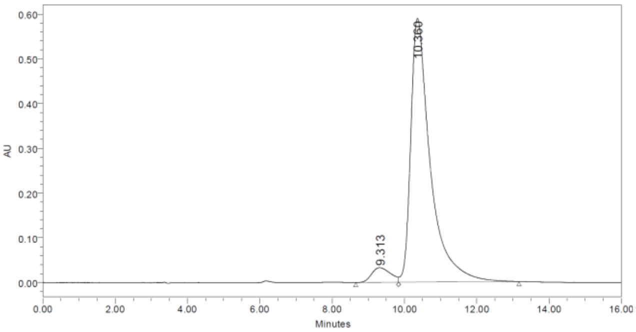

	RT	Area	% Area	Height
1	9.313	1180681	5.06	32644
2	10.360	22153146	94.94	588908

(*R*)-Diphenyl(7-phenyl-1-(7-phenylisoquinolin-1-yl)naphthalen-2-yl)phosphine oxide (3aaa)

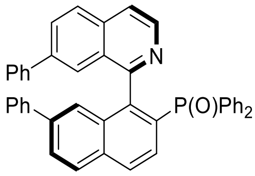

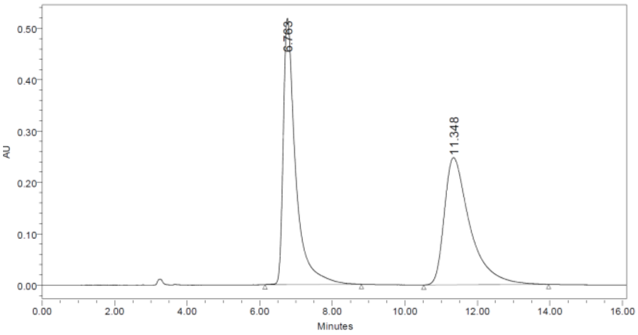

	RT	Area	% Area	Height
1	6.763	12291372	50.28	518262
2	11.348	12155262	49.72	247581

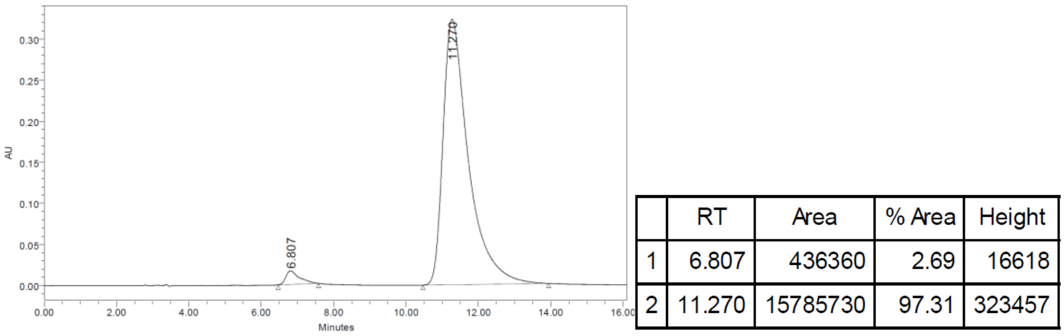

	RT	Area	% Area	Height
1	6.807	436360	2.69	16618
2	11.270	15785730	97.31	323457

(*R*)-Diphenyl(7-phenyl-1-(6-phenylisoquinolin-1-yl)naphthalen-2-yl)phosphine oxide (3aba)

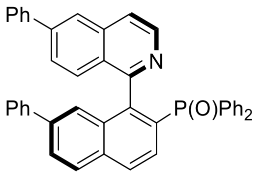

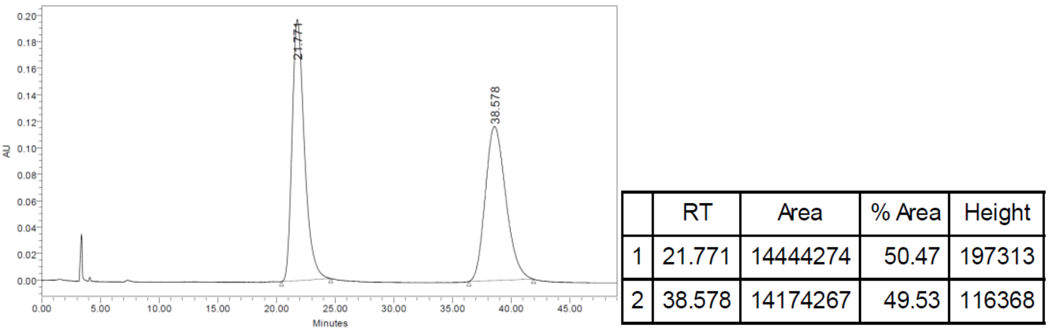

	RT	Area	% Area	Height
1	21.771	14444274	50.47	197313
2	38.578	14174267	49.53	116368

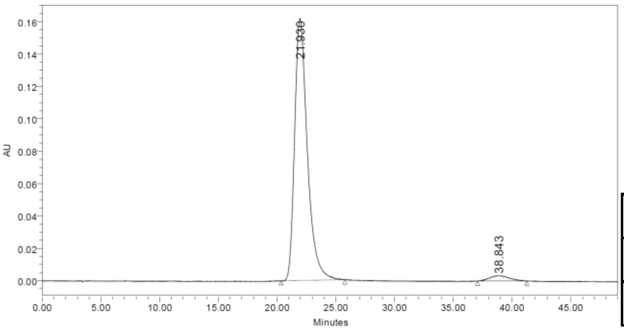

	RT	Area	% Area	Height
1	21.930	12195297	96.96	161901
2	38.843	381949	3.04	3313

(*R*)-Diphenyl(6-phenyl-1-(7-phenyl-8,8a-dihydroisoquinolin-1-yl)naphthalen-2-yl)phosphine oxide (3aca)

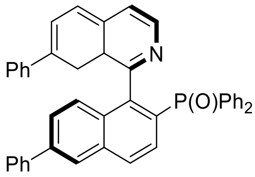

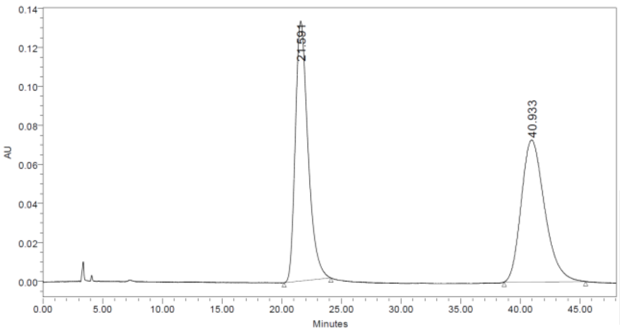

	RT	Area	% Area	Height
1	21.591	9837145	49.79	133312
2	40.933	9920627	50.21	72780

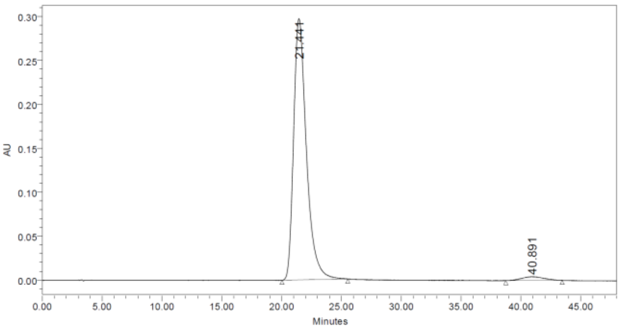

	RT	Area	% Area	Height
1	21.441	22079910	97.66	297559
2	40.891	529139	2.34	4199

(*R*)-(6-Methoxy-1-(6-phenylisoquinolin-1-yl)naphthalen-2-yl)diphenylphosphine oxide (3ada)

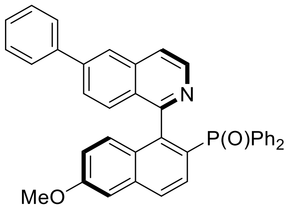

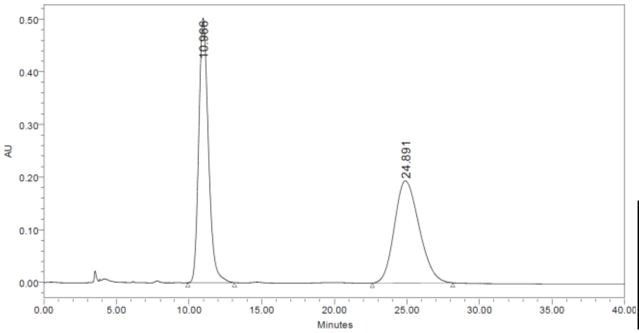

	RT	Area	% Area	Height
1	10.966	23173024	50.22	502056
2	24.891	22972718	49.78	193878

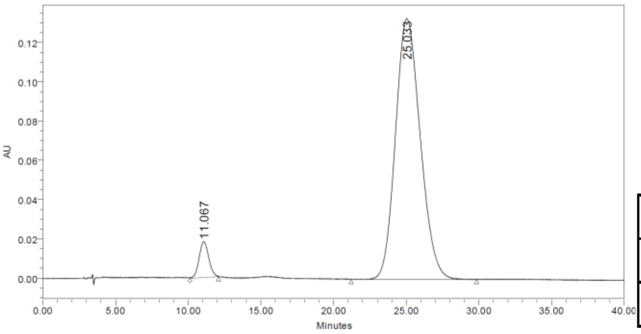

	RT	Area	% Area	Height
1	11.067	841614	5.07	18340
2	25.033	15744257	94.93	132868

(*R*)-(7-Methoxy-1-(7-phenylisoquinolin-1-yl)naphthalen-2-yl)diphenylphosphine oxide (3aea)

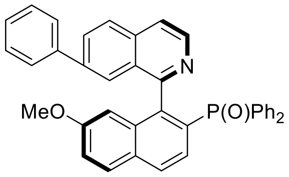

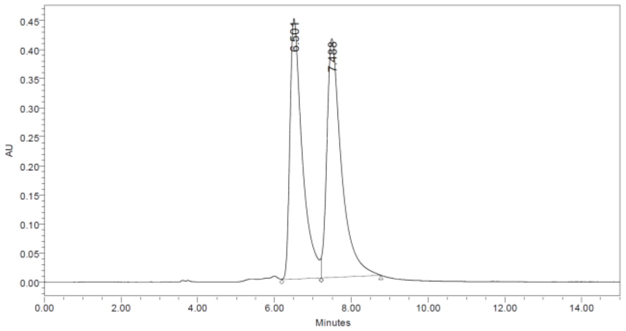

	RT	Area	% Area	Height
1	6.501	10056024	48.16	448806
2	7.488	10825692	51.84	411061

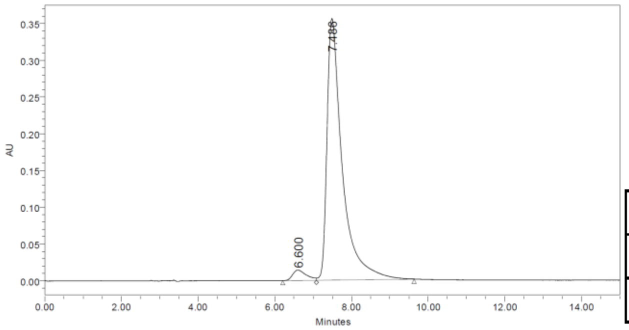

	RT	Area	% Area	Height
1	6.600	350823	3.45	14144
2	7.486	9827642	96.55	355607

(*R*)-(7-Methoxy-1-(6-phenylisoquinolin-1-yl)naphthalen-2-yl)diphenylphosphine oxide (3afa)

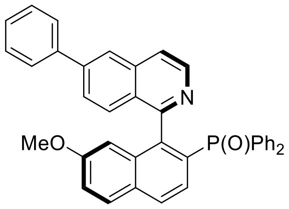

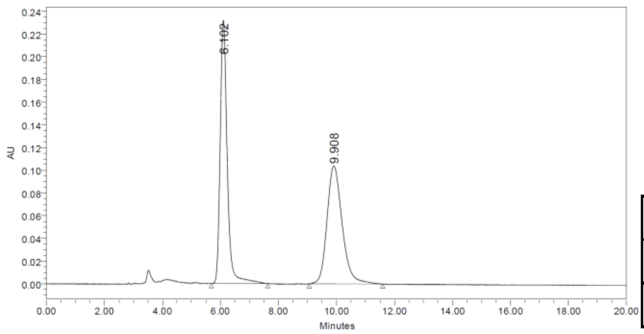

	RT	Area	% Area	Height
1	6.102	3917337	50.83	231793
2	9.908	3788793	49.17	104045

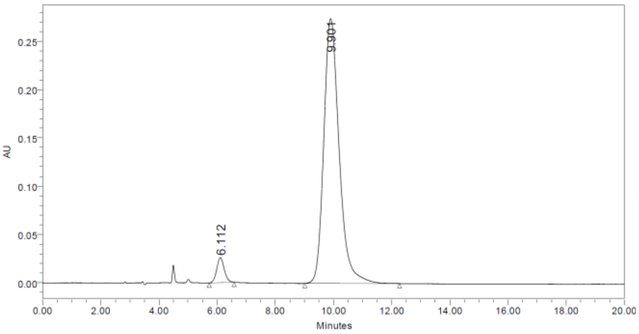

	RT	Area	% Area	Height
1	6.112	464906	4.42	25941
2	9.901	10050279	95.58	274212

(*R*)-(6-Methoxy-1-(7-phenylisoquinolin-1-yl)naphthalen-2-yl)diphenylphosphine oxide (3aga)

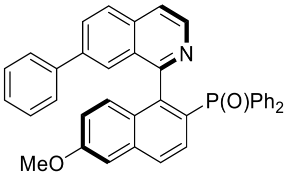

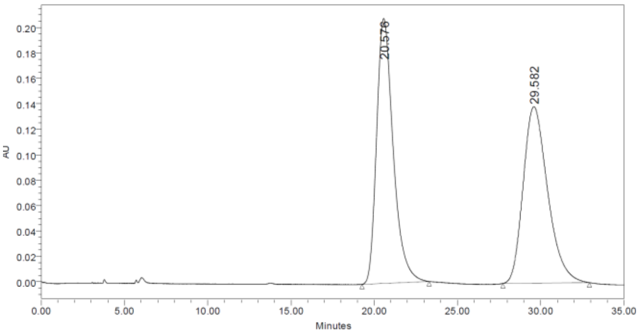

	RT	Area	% Area	Height
1	20.576	14438468	50.33	208327
2	29.582	14247660	49.67	138885

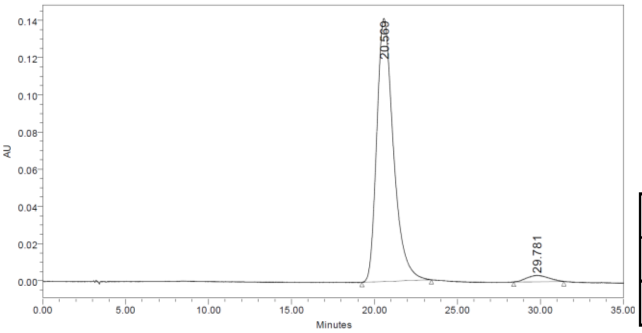

	RT	Area	% Area	Height
1	20.569	9881270	96.98	141508
2	29.781	308140	3.02	3398

(*R*)-Diphenyl(1-(quinazolin-4-yl)naphthalen-2-yl)phosphine oxide (3aha)

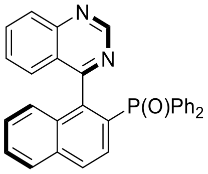

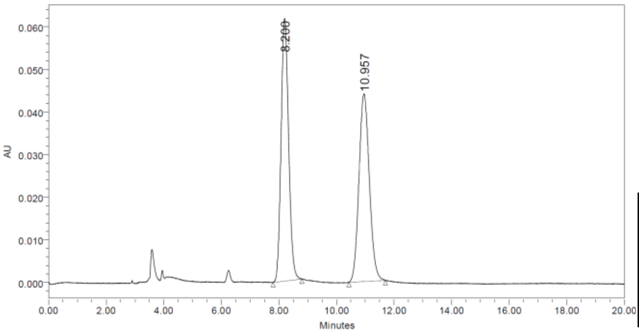

	RT	Area	% Area	Height
1	8.200	1109937	49.93	61603
2	10.957	1113177	50.07	44000

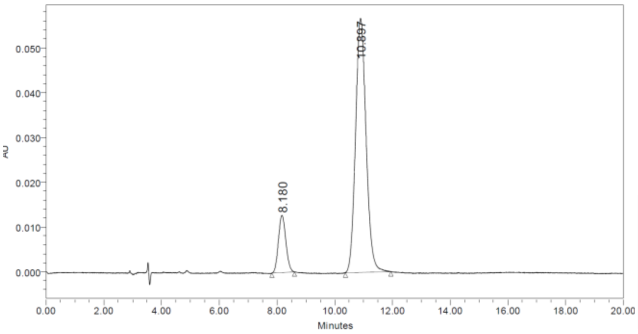

	RT	Area	% Area	Height
1	8.180	227844	13.58	12795
2	10.897	1450166	86.42	56722

(*R*)- (1-(Isoquinolin-1-yl)naphthalen-2-yl)di-p-tolylphosphine oxide (3ab)

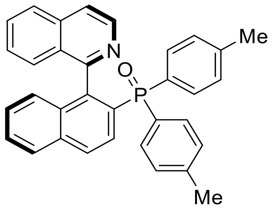

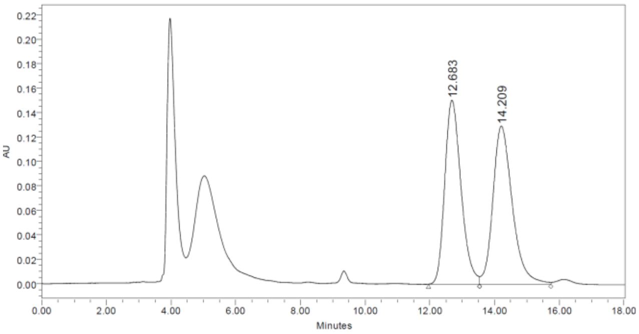

	RT	Area	% Area	Height
1	12.683	5273813	49.35	150566
2	14.209	5413521	50.65	129454

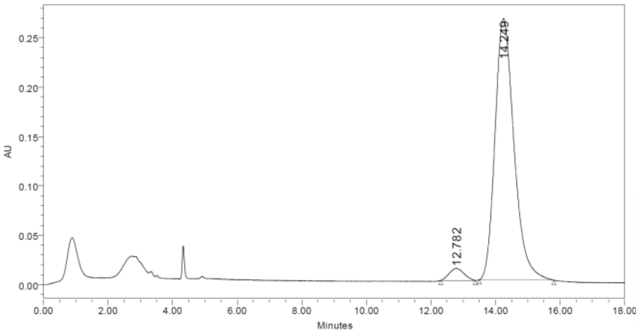

	RT	Area	% Area	Height
1	12.782	379898	3.39	12290
2	14.249	10833483	96.61	264747

(*R*)-(1-(Isoquinolin-1-yl)naphthalen-2-yl)di-m-tolylphosphine oxide (3ac)

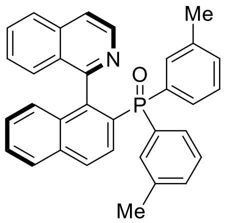

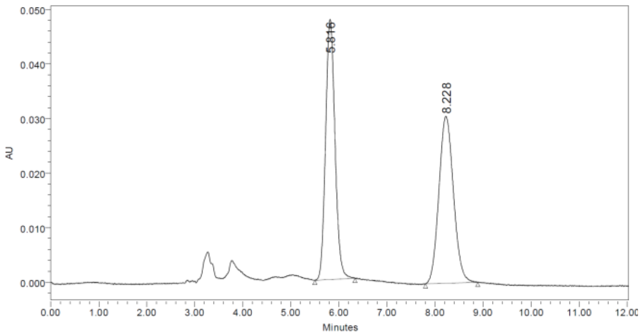

	RT	Area	% Area	Height
1	5.816	647375	50.04	47660
2	8.228	646353	49.96	30629

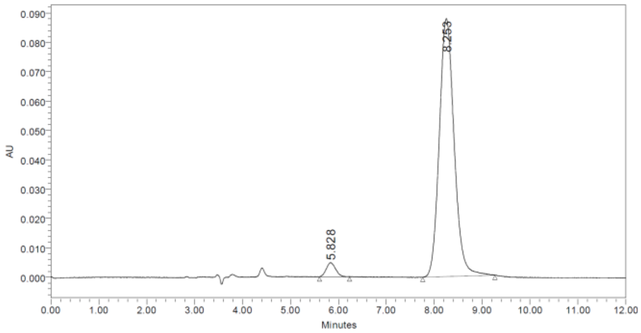

	RT	Area	% Area	Height
1	5.828	65976	3.35	4863
2	8.253	1900580	96.65	87907

(*R*)-bis(4-(*tert*-Butyl)phenyl)(1-(isoquinolin-1-yl)naphthalen-2-yl)phosphine oxide (3ae)

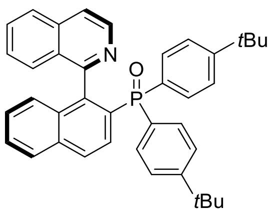

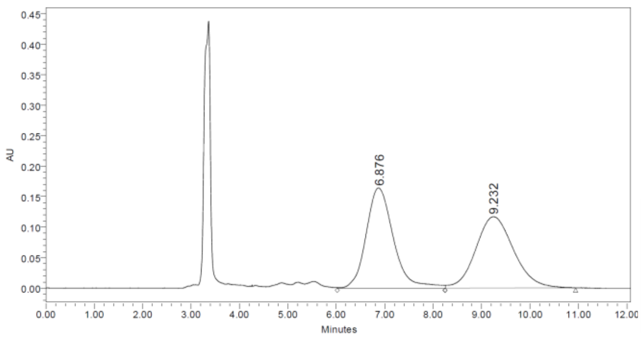

	RT	Area	% Area	Height
1	6.876	6411280	50.21	164284
2	9.232	6356694	49.79	117024

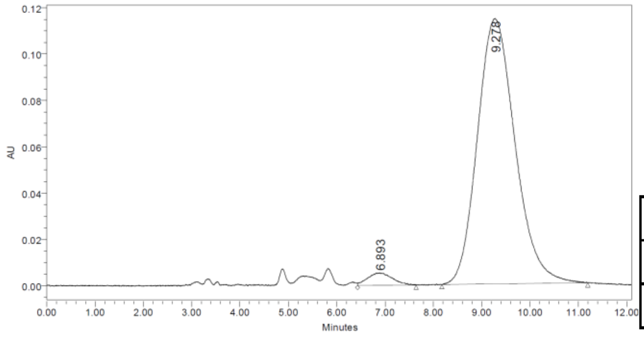

	RT	Area	% Area	Height
1	6.893	188278	2.94	5343
2	9.278	6222026	97.06	114590

(*R*)-(1-(Isoquinolin-1-yl)naphthalen-2-yl)bis(4-methoxyphenyl)phosphine oxide (3af)

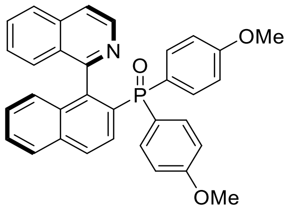

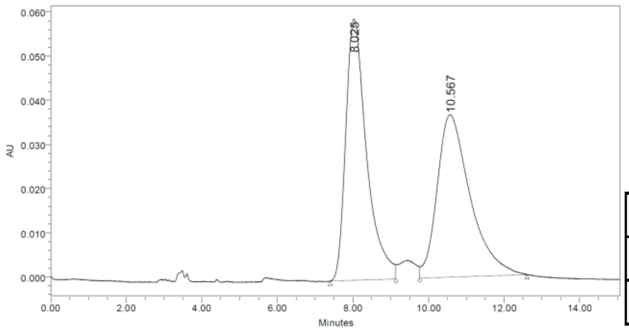

	RT	Area	% Area	Height
1	8.025	2270868	50.36	59122
2	10.567	2238434	49.64	36762

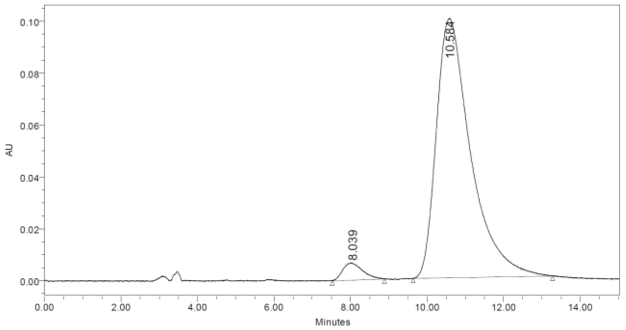

	RT	Area	% Area	Height
1	8.039	241360	3.72	6745
2	10.584	6242366	96.28	100103

(*R*)-Di([1,1'-biphenyl]-4-yl)(1-(isoquinolin-1-yl)naphthalen-2-yl)phosphine oxide (3ag)

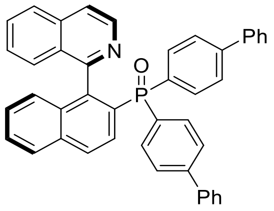

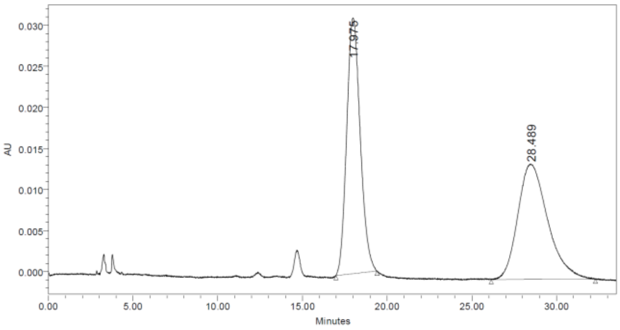

	RT	Area	% Area	Height
1	17.975	1738146	50.19	31170
2	28.489	1725078	49.81	14042

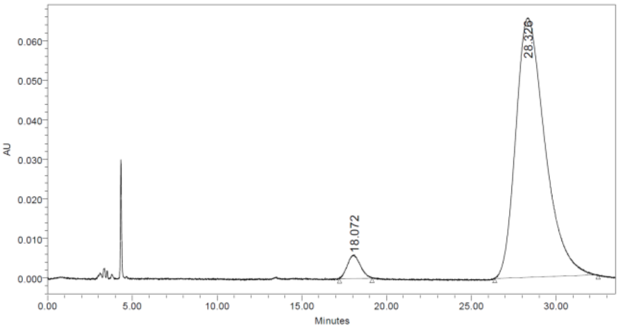

	RT	Area	% Area	Height
1	18.072	314977	3.86	5962
2	28.326	7850856	96.14	65555

(*R*)-bis(4-Fluorophenyl)(1-(isoquinolin-1-yl)naphthalen-2-yl)phosphine oxide (3ah)

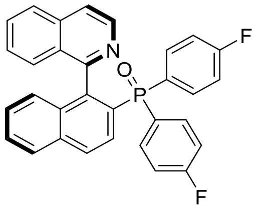

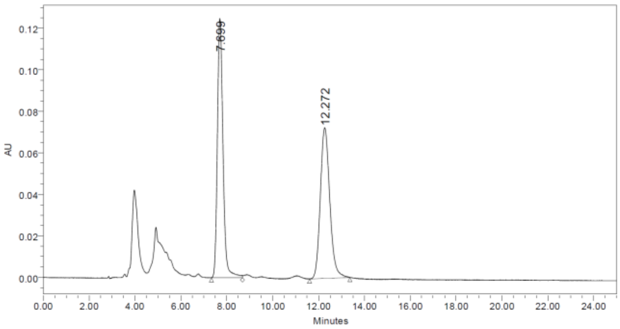

	RT	Area	% Area	Height
1	7.699	2120042	50.50	124832
2	12.272	2078034	49.50	72565

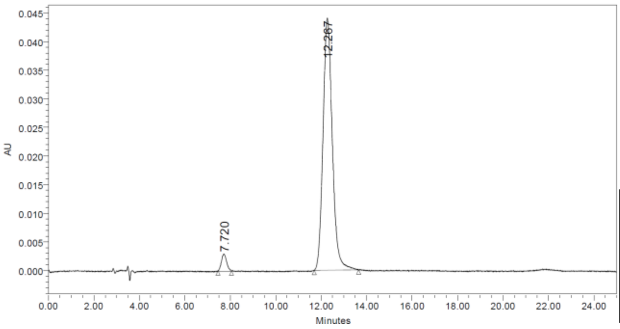

	RT	Area	% Area	Height
1	7.720	46260	3.52	2946
2	12.267	1266984	96.48	44054

(*R*)-bis(4-Chlorophenyl)(1-(isoquinolin-1-yl)naphthalen-2-yl)phosphine oxide (3ai)

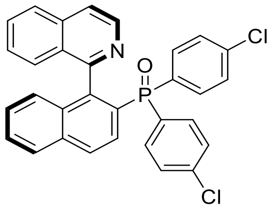

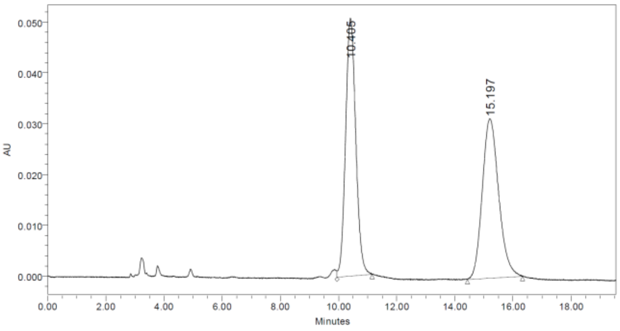

	RT	Area	% Area	Height
1	10.405	1231254	50.03	50757
2	15.197	1229936	49.97	31406

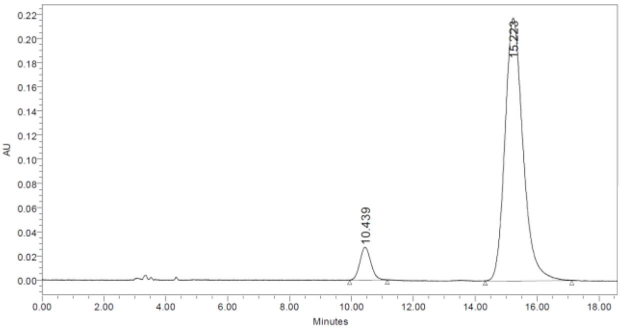

	RT	Area	% Area	Height
1	10.439	671377	7.05	27293
2	15.223	8846330	92.95	217707

(*R*)-(1-(Isoquinolin-1-yl)naphthalen-2-yl)bis(4-(trifluoromethyl)phenyl)phosphine oxide (3aj)

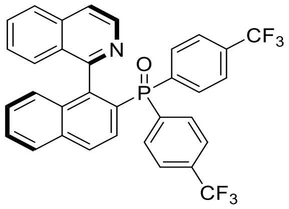

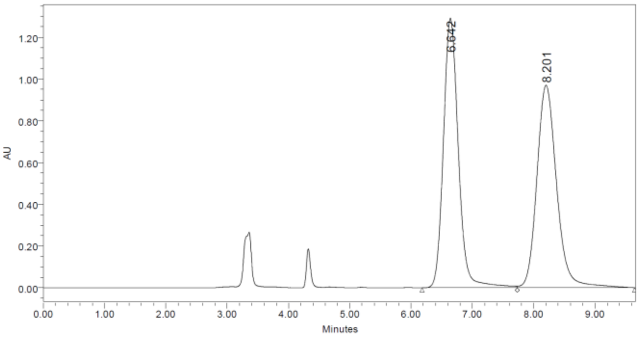

	RT	Area	% Area	Height
1	6.642	21970353	50.25	1291812
2	8.201	21753906	49.75	970692

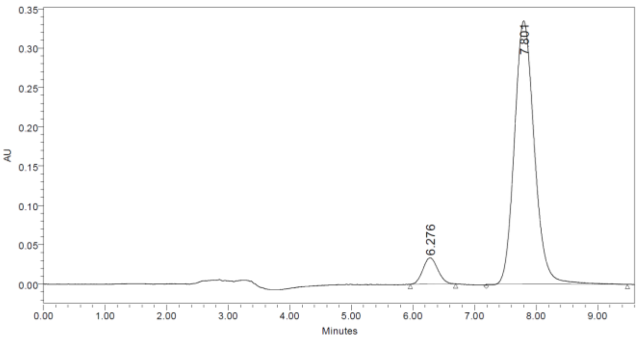

	RT	Area	% Area	Height
1	6.276	564327	6.96	33500
2	7.801	7545961	93.04	334752

(*R*)-bis(3,5-Dimethylphenyl)(1-(isoquinolin-1-yl)naphthalen-2-yl)phosphine oxide (3ak)

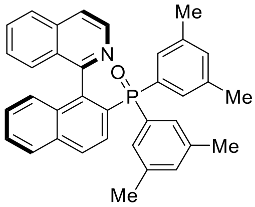

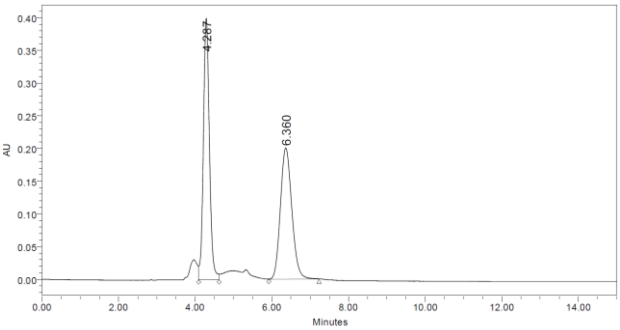

	RT	Area	% Area	Height
1	4.287	4363228	51.29	399775
2	6.360	4144193	48.71	200674

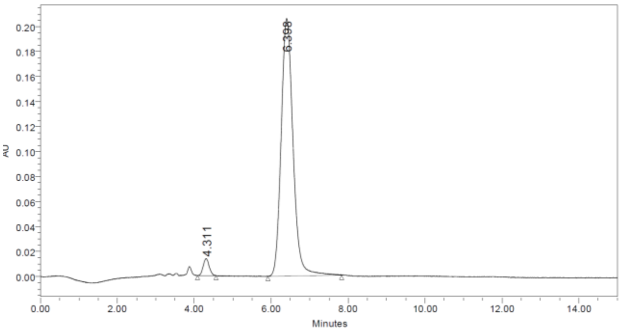

	RT	Area	% Area	Height
1	4.311	145234	3.18	13384
2	6.398	4420644	96.82	206152

(*R*)-(1-(Isoquinolin-1-yl)naphthalen-2-yl)bis(4-methoxy-3,5-dimethylphenyl)phosphine oxide (3al)

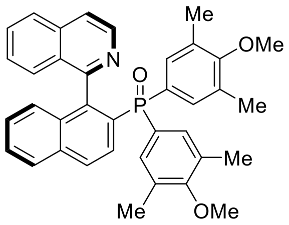

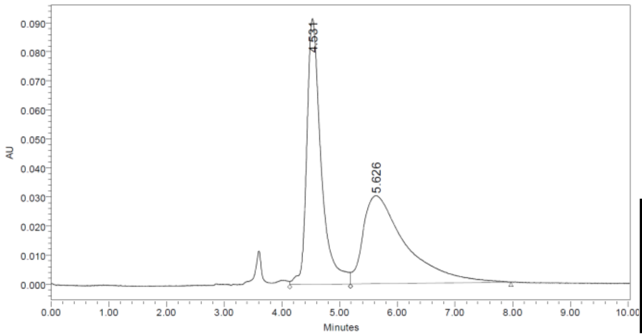

	RT	Area	% Area	Height
1	4.531	1577266	50.30	91583
2	5.626	1558409	49.70	30354

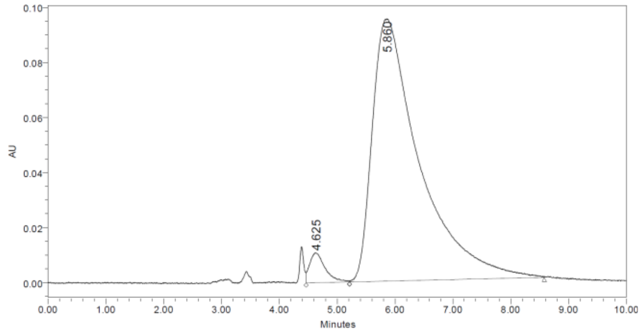

	RT	Area	% Area	Height
1	4.625	202469	3.62	10858
2	5.860	5386322	96.38	95200

(*R*)-(1-(Isoquinolin-1-yl)naphthalen-2-yl)di(naphthalen-2-yl)phosphine oxide (3am)

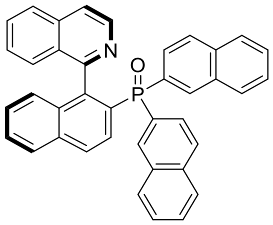

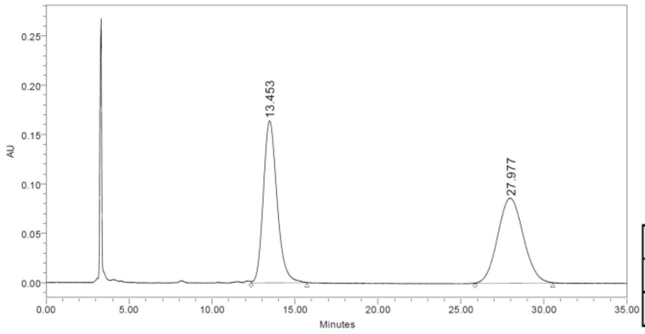

	RT	Area	% Area	Height
1	13.453	9386162	50.34	164301
2	27.977	9259880	49.66	86296

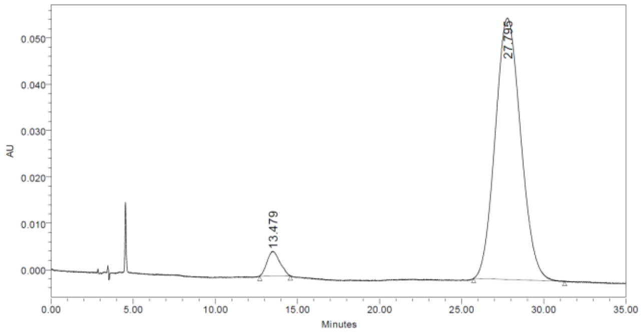

	RT	Area	% Area	Height
1	13.479	291656	4.56	5371
2	27.795	6110977	95.44	56455

(*R*)-Dibenzyl(1-(isoquinolin-1-yl)naphthalen-2-yl)phosphine oxide (3an)

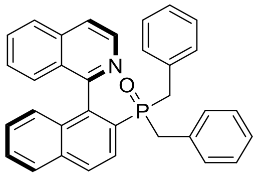

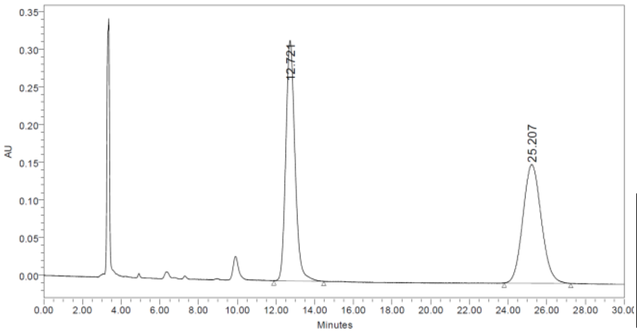

	RT	Area	% Area	Height
1	12.721	10502918	50.25	319225
2	25.207	10396332	49.75	157572

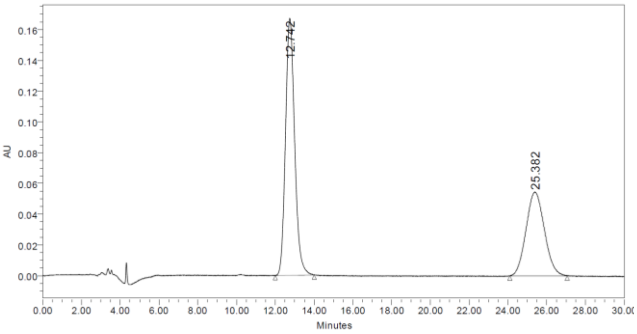

	RT	Area	% Area	Height
1	12.742	5469034	60.51	167263
2	25.382	3569039	39.49	54530

(*R*)-Ethyl(1-(isoquinolin-1-yl)naphthalen-2-yl)(phenyl)phosphine oxide (3ao)

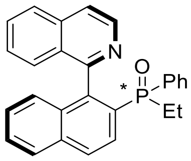

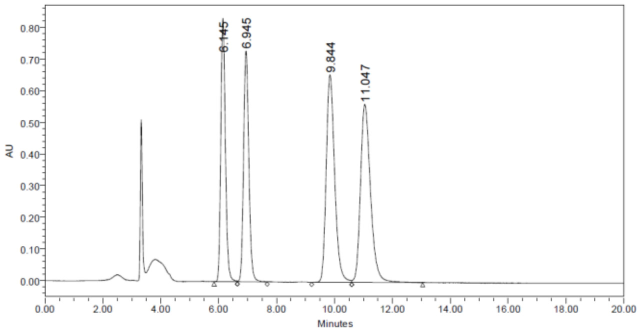

	RT	Area	% Area	Height
1	6.145	9119452	20.19	831353
2	6.945	9178237	20.32	729658
3	9.844	13321383	29.49	655599
4	11.047	13557520	30.01	562571

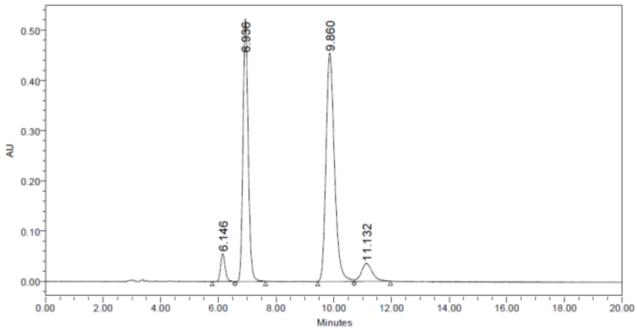

	RT	Area	% Area	Height
1	6.146	622162	3.55	55545
2	6.936	6598310	37.68	522781
3	9.860	9344925	53.37	454676
4	11.132	945510	5.40	35825

(*R*)-Ethyl (1-(isoquinolin-1-yl)naphthalen-2-yl)(phenyl)phosphinate (3ap)

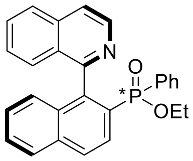

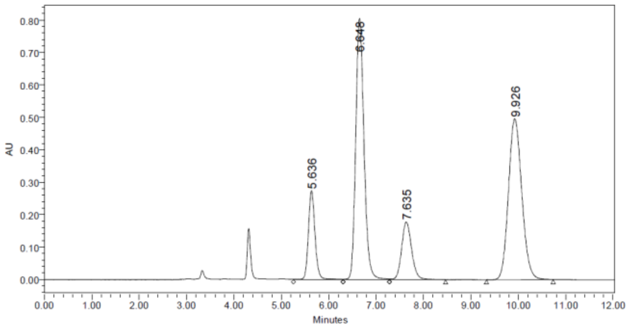

	RT	Area	% Area	Height
1	5.636	2679540	10.82	273879
2	6.648	9751755	39.36	804607
3	7.635	2618761	10.57	177758
4	9.926	9725843	39.26	495977

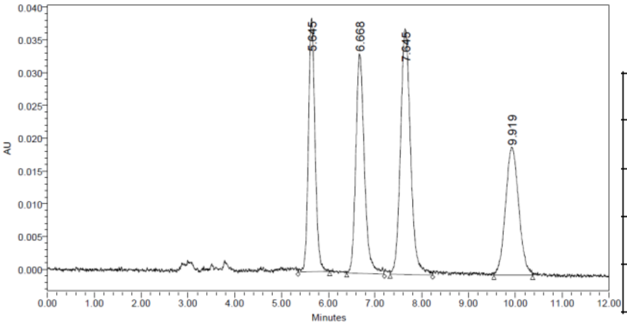

	RT	Area	% Area	Height
1	5.645	375787	21.54	38657
2	6.668	426314	24.44	33528
3	7.645	559219	32.06	37447
4	9.919	383021	21.96	19532

(*R*)-1-(2-(Diphenylphosphaneyl)naphthalen-1-yl)isoquinoline (3aa')

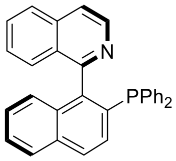

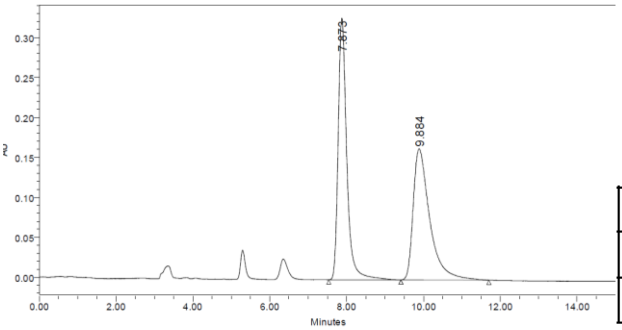

	RT	Area	% Area	Height
1	7.873	5080113	50.81	327240
2	9.884	4919100	49.19	164148

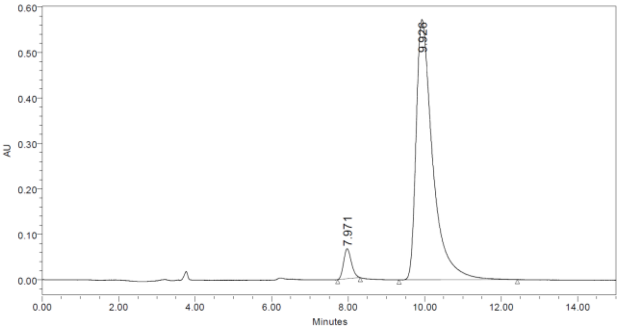

	RT	Area	% Area	Height
1	7.971	936333	5.15	66005
2	9.926	17262260	94.85	572982

(*R*)-1-(2-(Diphenylphosphaneyl)naphthalen-1-yl)-4-phenylisoquinoline (3ha')

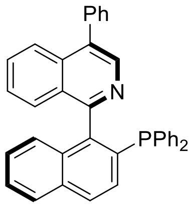

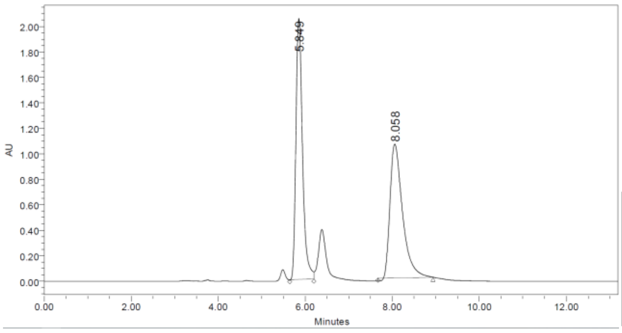

	RT	Area	% Area	Height
1	5.849	21403872	50.33	2047134
2	8.058	21127252	49.67	1049240

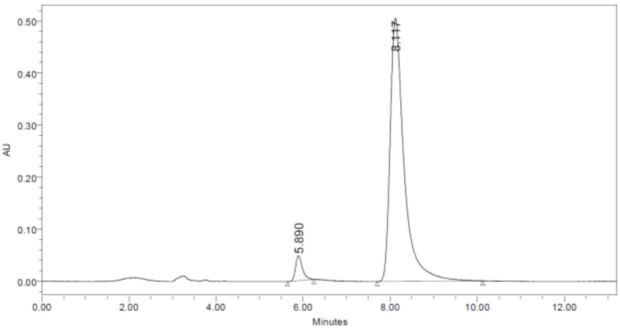

	RT	Area	% Area	Height
1	5.890	522912	4.46	47641
2	8.117	11209649	95.54	505388

(*R*)-1-(2-(Diphenylphosphaneyl)-6-phenylnaphthalen-1-yl)-6-phenylisoquinoline (3za')

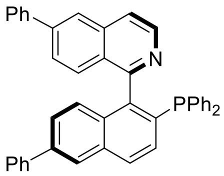

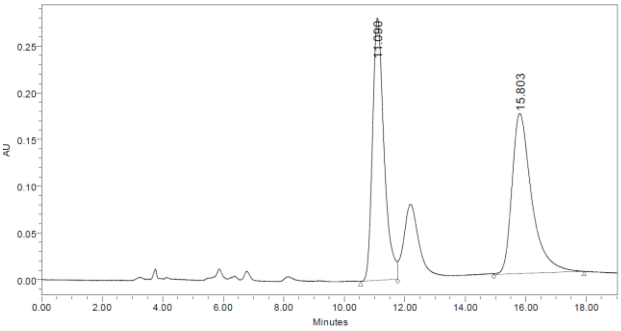

	RT	Area	% Area	Height
1	11.090	7397763	48.82	280524
2	15.803	7754796	51.18	171466

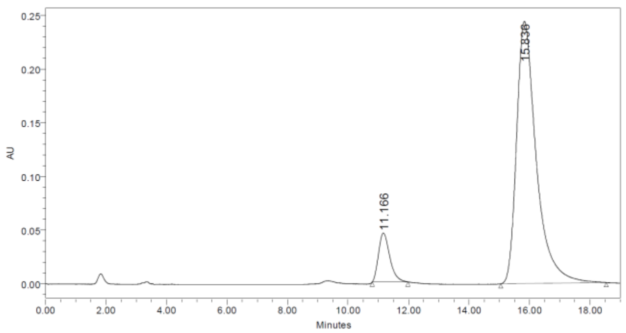

	RT	Area	% Area	Height
1	11.166	1175490	9.60	45618
2	15.836	11074224	90.40	244240

(*R*)-1-(2-(Diphenylphosphaneyl)-7-phenylnaphthalen-1-yl)-7-phenylisoquinoline (3aaa’)

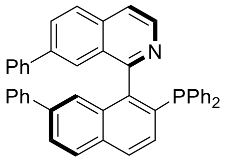

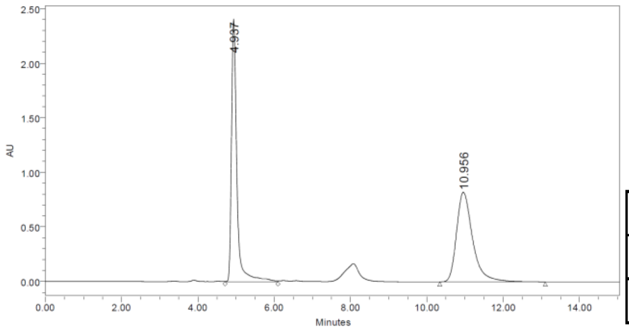

	RT	Area	% Area	Height
1	4.937	23571874	49.52	2404409
2	10.956	24027907	50.48	821245

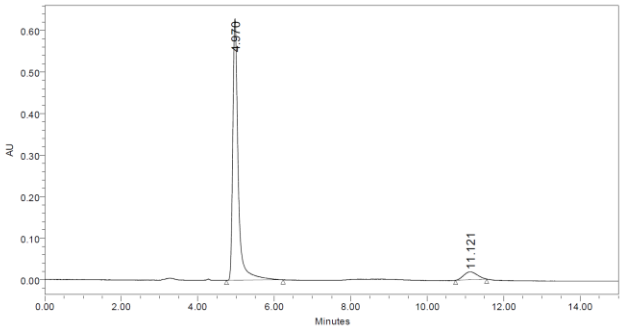

	RT	Area	% Area	Height
1	4.970	6185229	92.96	629991
2	11.121	468750	7.04	19076

(*R*)-1,1'-Binaphthalen]-2-yldiphenylphosphine oxide (12)

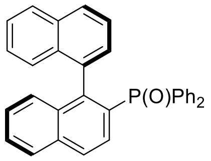

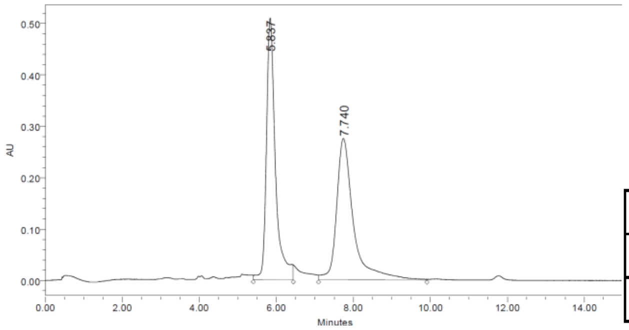

	RT	Area	% Area	Height
1	5.837	8250898	49.65	508820
2	7.740	8366645	50.35	275043

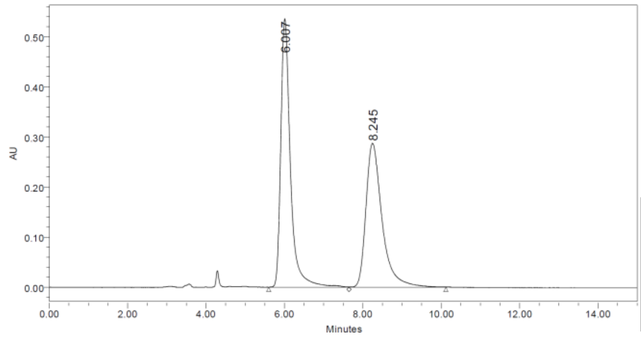

	RT	Area	% Area	Height
1	6.007	9136920	52.66	535812
2	8.245	8212423	47.34	287364

(*R*)-(1-(3-Methylpyridin-2-yl)naphthalen-2-yl)diphenylphosphine oxide (13)

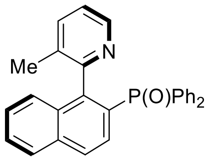

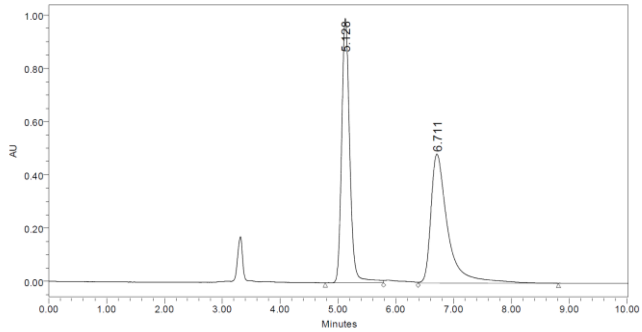

	RT	Area	% Area	Height
1	5.128	9635999	49.61	992300
2	6.711	9788206	50.39	484614

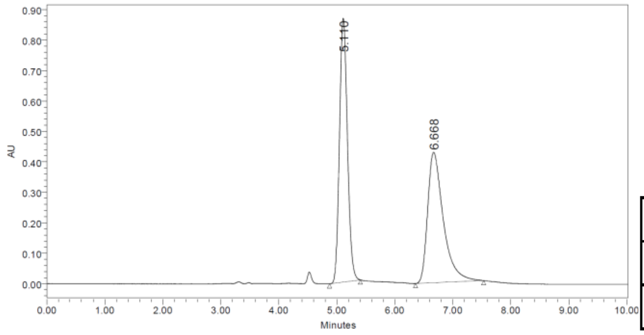

	RT	Area	% Area	Height
1	5.110	8197480	50.42	866389
2	6.668	8059939	49.58	428734

Dimethyl (*S,E*)-2-(1,3-diphenylallyl)malonate (6)

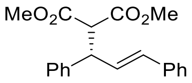

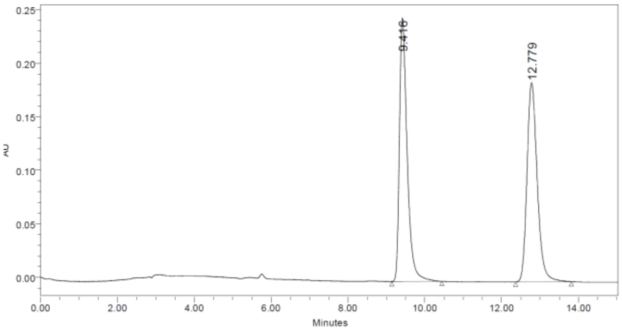

	RT	Area	% Area	Height
1	9.416	3440689	50.03	245991
2	12.779	3436385	49.97	186040

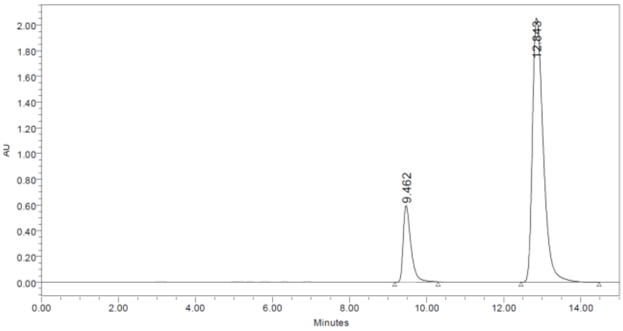

	RT	Area	% Area	Height
1	9.462	8266623	17.01	596349
2	12.843	40329601	82.99	2052592

(*R*)-2-(Phenylethynyl)chroman-4-one (9)

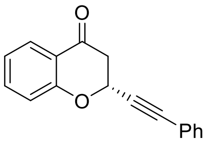

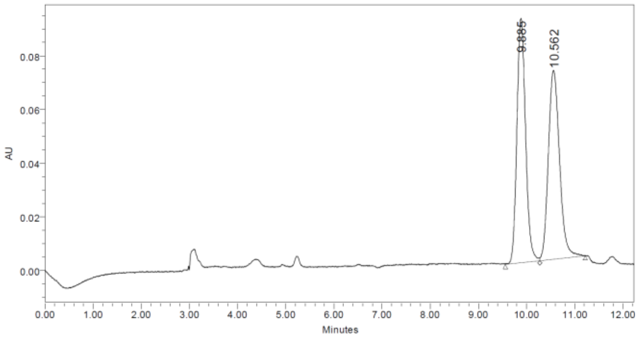

	RT	Area	% Area	Height
1	9.885	1131082	49.84	91019
2	10.562	1138319	50.16	70362

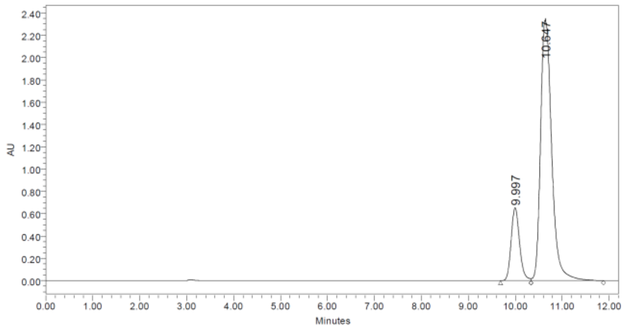

	RT	Area	% Area	Height
1	9.997	8142889	17.45	653850
2	10.647	38514064	82.55	2348626
